# Efficacy and Safety of Three Antiretroviral Regimens for Initial Treatment of HIV-1: A Randomized Clinical Trial in Diverse Multinational Settings

**DOI:** 10.1371/journal.pmed.1001290

**Published:** 2012-08-14

**Authors:** Thomas B. Campbell, Laura M. Smeaton, N. Kumarasamy, Timothy Flanigan, Karin L. Klingman, Cynthia Firnhaber, Beatriz Grinsztejn, Mina C. Hosseinipour, Johnstone Kumwenda, Umesh Lalloo, Cynthia Riviere, Jorge Sanchez, Marineide Melo, Khuanchai Supparatpinyo, Srikanth Tripathy, Ana I. Martinez, Apsara Nair, Ann Walawander, Laura Moran, Yun Chen, Wendy Snowden, James F. Rooney, Jonathan Uy, Robert T. Schooley, Victor De Gruttola, James Gita Hakim, PEARLS study team of the ACTG, Edith Swann, Ronald L. Barnett, Barbara Brizz, Yvette Delph, Nikki Gettinger, Ronald T. Mitsuyasu, Susan Eshleman, Steven Safren, Susan A. Fiscus, Adriana Andrade, David W. Haas, Farida Amod, Vladimir Berthaud, Robert C. Bollinger, Yvonne Bryson, David Celentano, David Chilongozi, Myron Cohen, Ann C. Collier, Judith Silverstein Currier, Susan Cu-Uvin, Joseph Eron, Charles Flexner, Joel E. Gallant, Roy M. Gulick, Scott M. Hammer, Irving Hoffman, Peter Kazembe, Newton Kumwenda, Javier R. Lama, Jody Lawrence, Chiedza Maponga, Francis Martinson, Kenneth Mayer, Karin Nielsen, Richard B. Pendame, Bharat Ramratnam, Ian Sanne, Patrice Severe, Thira Sirisanthana, Suniti Solomon, Steve Tabet, Taha Taha, Charles van der Horst, Christine Wanke, Joan Gormley, Cheryl J. Marcus, Beverly Putnam, Edde Loeliger, Keith A. Pappa, Nancy Webb, David L. Shugarts, Mark A. Winters, Renard S. Descallar, Joseph Steele, Michael Wulfsohn, Farideh Said, Yue Chen, John C Martin, Norbert Bischofberger, Andrew Cheng, Howard Jaffe, Jabin Sharma, S. Poongulali, Sandra Wagner Cardoso, Deise Lucia Faria, Sima Berendes, Kelly Burke, Rosie Mngqibisa, Cecelia Kanyama, Virginia Kayoyo, Wadzanai P. Samaneka, Anthony Chisada, Sharla Faesen, Suwat Chariyalertsak, Breno Santos, Rita Alves Lira, Anjali A. Joglekar, Alberto La Rosa, Rosa Infante, Mamta Jain, Tianna Petersen, Sheela Godbole, Sampada Dhayarkar, Judith Feinberg, Jenifer Baer, Richard B. Pollard, David Asmuth, Raman R Gangakhedkar, Asmita Gaikwad, M. Graham Ray, Cathi Basler, Michael F. Para, Kathy J. Watson, Babafemi Taiwo, Donna McGregor, Henry H. Balfour, Beth Mullan, Ge-Youl Kim, Michael K. Klebert, Gary Matthew Cox, Martha Silberman, Donna Mildvan, Manuel Revuelta, Karen T. Tashima, Helen Patterson, P. Jan Geiseler, Bartolo Santos, Eric S Daar, Ruben Lopez, Laurie Frarey, David Currin, David H. Haas, Vicki L. Bailey, Pablo Tebas, Larisa Zifchak, Jolene Noel-Connor, Madeline Torres, Beverly E. Sha, Janice M. Fritsche, Michelle Cespedes, Janet Forcht, William A. O'Brien, Cheryl Mogridge, Christine Hurley, Roberto Corales, Maria Palmer, Mary Adams, Amneris Luque, Luis Lopez-Detres, Todd Stroberg

**Affiliations:** HIV Research Branch, TRP, DAIDS, NIAD, NIH, Bethesda, Maryland; ACTG Operations Centre, Social & Scientific Systems, Inc. Silver Spring, Maryland; ACTG Operations Center, Social & Scientific Systems, Inc. Silver Spring, Maryland; ACTG Operations Center, Social & Scientific Systems, Inc. Silver Spring, Maryland; ACTG Operations Center, Social & Scientific Systems Inc. Silver Spring, Maryland; UCLA CARE Center, Los Angeles, California; Johns Hopkins University, Baltimore, Maryland; Harvard Medical School, Boston, Massachusetts; Department of Microbiology & Immunology, University of North Carolina, School of Medicine, Chapel Hill, North Carolina; Division of Infectious Diseases, John Hopkins University, Baltimore; Infectious Diseases, Vanderbilt University, Nashville, Tennessee; FCPath, FCP, Department of Medicine, Nelson R Mandela School of Medicine, Durban, South Africa; Infectious Disease, Vanderbilt University Medical Centre, Nashville, Tennessee; Division of Infectious Diseases, John Hopkins University, Baltimore, Maryland; Pediatric Infectious Disease Dept., UCLA School of Medicine, Los Angeles, California; Department of Epidemiology, Johns Hopkins School of Hygiene and Public Health, Baltimore, Maryland; UNC HIVNET, UNC Project, Lilongwe, Malawi; University of North Carolina, Chapel Hill, North Carolina; University of Washington, ACTU, Harborview Medical Centre, Seattle, Washington; University of California, Los Angeles, California; The Miriam Hospital, Brown University, Immunology Centre, Providence, Rhode Island; Division of Infectious Diseases, Dept. of Medicine, University of North Carolina, Chapel Hill, North Carolina; Johns Hopkins University Hospital, Baltimore, Maryland; Division of Infectious Diseases, Johns Hopkins University School of Medicine, Baltimore, Maryland; The Cornell Clinical Trials Unit, New York, New York; Division of Infectious Diseases, Columbia Presbyterian Medical Centre, New York, New York; University of North Carolina, Chapel Hill, North Carolina; Baylor College of Medicine-Abbott Fund Children's Clinical Centre of Excellence, Lilongwe, Malawi; Johns Hopkins Project, Malawi; Investigaciones Medicas en Salud (INMENSA), Lima, Peru; University of California, San Francisco, Adult AIDS Clinical Trials Unit, San Francisco, California; Medical University of Zimbabwe, Zimbabwe; UNC Project, Lilongwe; Division of Infectious Diseases, Brown University School of Medicine, Memorial Hospital of Rhode Island, Pawtucket, Rhode Island; UCLA School of Medicine, Los Angeles, California; Malawi; Laboratory of Retrovirology, Division of Infectious Diseases, Brown University Medical School, Providence, Rhode Island; University of Witwatersrand, Johannesburg, South Africa; Internal Medical, Infectious Diseases, Institute de Laboratories et de Recherches, Port-au-Prince, Haiti; Research Institute for Health Sciences, Chiang Mai University, Chiang Mai, Thailand; YRG Centre for AIDS Research and Education, Chennai, India; University of Washington, Harborview Medical Centre, Seattle, Washington; Johns Hopkins University, School of Hygiene & Public Health, Baltimore, Maryland; Department of Medicine, University of North Carolina, Chapel Hill, North Carolina; Tufts University School of Medicine, Boston, Massachusetts; The Miriam Hospital, Immunology Centre, Providence, Rhode Island; University of North Carolina, Chapel Hill, North Carolina; University of Colorado Health Sciences, Denver, Colorado; Smanga Ntshele, Community Advisory Board Member, Durban, South Africa; Clinical Development & Medical Affairs, Greenford, Middlesex; GlaxoSmithKline, Infectious Diseases Medicine, Triangle Park, North Carolina; Frontier Science & Technology Research Foundation, Inc., Amherst, Massachusetts; University of Colorado Health Sciences, Denver, Colorado; Stanford University Medical Center, Division of Infectious Disease, Stanford, California; Gilead Sciences, Foster City, California; YRG Centre for AIDS Research and Education, India; YRG Centre for AIDS Research and Education, Chennai, India; Instituto de Pesquisa Clinica Evandro Chagas-Fiocruz, Brazil; Instituto de Pesquisa Clinica Evandro Chagas-Fiocruz, Brazil; College of Medicine, Blantyre, Malawi; Blantyre, Malawi; Nelson Mandela School of Medicine, Durban, South Africa; Kamuzu Central Hospital, Lilongwe, Malawi; Kamuzu Central Hospital, Lilongwe, Malawi; University of Zimbabwe College of Health Sciences, Harare, Zimbabwe; University of Zimbabwe College of Health Sciences, Harare, Zimbabwe; University of the Witwatersrand, Johannesburg, South Africa; Chiang Mai University, Chiang Mai, Thailand; Hospital Conceicao, Porto Alegre, Brazil; Hospital Conceicao, Porto Alegre, Brazil; National AIDS Research Institute, Pune, India; Asociacion Civil Impacta Salud y Educacion - Miraflores, Lima, Peru; Investigaciones Medicas en Salud – INMENSA, Lima, Peru; UT Southwestern Medical Center at Dallas, Dallas, Texas; UT Southwestern Medical Center at Dallas, Dallas, Texas; NARI Clinic at NIV, Pune, India; NARI Clinic at NIV, Pune, India; University of Cincinnati, Cincinnati, Ohio; University of Cincinnati, Cincinnati, Ohio; UC Davis School of Medicine, Davis, California; UC Davis School of Medicine, Davis, California; NARI Clinic at Gadikhana Dr. Kotnis Municipal Dispensary, Pune, India; NARI Clinic at Gadikhana Dr. Kotnis Municipal Dispensary, Pune, India; University of Colorado Hospital, Aurora, Colorado; University of Colorado Hospital, Aurora, Colorado; The Ohio State University, Ohio; The Ohio State University, Ohio; Northwestern University, Chicago, Illinois; Northwestern University, Chicago, Illinois; University of Minnesota, Minneapolis, Minnesota; University of Minnesota, Minneapolis, Minnesota; Washington University, Saint Louis, Missouri; Washington University, Saint Louis, Missouri; Duke University Medical Center, Durham, North Carolina; Duke University Medical Center, Durham, North Carolina; Beth Israel Medical Center, New York, New York; Beth Israel Medical Center, New York, New York; The Miriam Hospital, Providence, Rhode Island; The Miriam Hospital, Providence, Rhode Island; University of Southern California; Los Angeles, California; University of Southern California, Los Angeles, California; Harbor-UCLA, Los Angeles, California; Harbor-UCLA, Los Angeles, California; University of North Carolina, Chapel Hill, North Carolina; University of NorthCarolina, Chapel Hill, North Carolina; Vanderbilt University, Nashville, Tennessee; Vanderbilt University, Nashville, Tennessee; Hospital of the University of Pennsylvania, Philadelphia, Pennsylvania; Hospital of the University of Pennsylvania, Philadelphia, Pennsylvania; Columbia University, New York, New York; Columbia University, New York, New York; Rush University Medical Center, Chicago, Illinois; Rush University Medical Center, Chicago, Illinois; New York University/NYC HHC at Bellevue Hospital Center, New York, New York; New York University/NYC HHC at Bellevue Hospital Center, New York, New York; The University of Texas Medical Branch, Galveston, Texas; The University of Texas Medical Branch, Galveston, Texas; AIDS Care, Georgetown University, Washington (D.C.); AIDS Care, Georgetown University, Washington (D.C.); UCLA CARE Center, Los Angeles, California; University of Rochester, Rochester, New York; University of Rochester, Rochester, New York; Cornell University, New York, New York; Cornell University, New York, New York; 1 Division of Infectious Diseases, Department of Medicine, University of Colorado School of Medicine, Aurora, United States of America; 2 Center for Biostatistics in AIDS Research, Harvard School of Public Health, Boston, Massachusetts, United States of America; 3 YRG Centre for AIDS Research & Education, Chennai, India; 4 Brown Medical School, Providence, Rhode Island, United States of America; 5 National Institutes of Health, Bethesda, Maryland, United States of America; 6 Clinical HIV Research Unit, Department of Medicine, Faculty of Health Sciences, University of the Witwatersrand, Johannesburg, South Africa; 7 Evandro Chagas Clinical Research Institute, Fiocruz, Rio de Janeiro, Brazil; 8 Kamuzu Central Hospital, Lilongwe, Malawi; 9 Department of Medicine, College of Medicine, Blantyre, Malawi; 10 Nelson R. Mandela School of Medicine, Durban, South Africa; 11 Institut Nacional de laboratoire et de Recherches, Port-au-Prince, Haiti; 12 Asociación Civil Impacta Salud y Educación, Lima, Peru; 13 Servico de Infectology, Hospital Nossa Senhora da Conceicao-GHC, Porto Alegre, Brazil; 14 Department of Medicine and Research Institute for Health Sciences, Chiang Mai University, Chiang Mai, Thailand; 15 National AIDS Research Institute, Pune, India; 16 Frontier Science & Technology Research Foundation, Amherst, Massachusetts, United States of America; 17 Social & Scientific Systems, Inc, Silver Spring, Maryland, United States of America; 18 GlaxoSmithKline, Research Triangle Park, North Carolina, United States of America; 19 Gilead Sciences, Inc., Foster City, California, United States of America; 20 Bristol-Myers Squibb Company, Plainsboro, New Jersey, United States of America; 21 University of California San Diego, San Diego, United States of America; 22 University of Zimbabwe College of Health Sciences, Harare, Zimbabwe; San Francisco General Hospital, United States of America

## Abstract

**Background:**

Antiretroviral regimens with simplified dosing and better safety are needed to maximize the efficiency of antiretroviral delivery in resource-limited settings. We investigated the efficacy and safety of antiretroviral regimens with once-daily compared to twice-daily dosing in diverse areas of the world.

**Methods and Findings:**

1,571 HIV-1-infected persons (47% women) from nine countries in four continents were assigned with equal probability to open-label antiretroviral therapy with efavirenz plus lamivudine-zidovudine (EFV+3TC-ZDV), atazanavir plus didanosine-EC plus emtricitabine (ATV+DDI+FTC), or efavirenz plus emtricitabine-tenofovir-disoproxil fumarate (DF) (EFV+FTC-TDF). ATV+DDI+FTC and EFV+FTC-TDF were hypothesized to be non-inferior to EFV+3TC-ZDV if the upper one-sided 95% confidence bound for the hazard ratio (HR) was ≤1.35 when 30% of participants had treatment failure.

An independent monitoring board recommended stopping study follow-up prior to accumulation of 472 treatment failures. Comparing EFV+FTC-TDF to EFV+3TC-ZDV, during a median 184 wk of follow-up there were 95 treatment failures (18%) among 526 participants versus 98 failures among 519 participants (19%; HR 0.95, 95% CI 0.72–1.27; *p* = 0.74). Safety endpoints occurred in 243 (46%) participants assigned to EFV+FTC-TDF versus 313 (60%) assigned to EFV+3TC-ZDV (HR 0.64, CI 0.54–0.76; *p*<0.001) and there was a significant interaction between sex and regimen safety (HR 0.50, CI 0.39–0.64 for women; HR 0.79, CI 0.62–1.00 for men; *p* = 0.01). Comparing ATV+DDI+FTC to EFV+3TC-ZDV, during a median follow-up of 81 wk there were 108 failures (21%) among 526 participants assigned to ATV+DDI+FTC and 76 (15%) among 519 participants assigned to EFV+3TC-ZDV (HR 1.51, CI 1.12–2.04; *p* = 0.007).

**Conclusion:**

EFV+FTC-TDF had similar high efficacy compared to EFV+3TC-ZDV in this trial population, recruited in diverse multinational settings. Superior safety, especially in HIV-1-infected women, and once-daily dosing of EFV+FTC-TDF are advantageous for use of this regimen for initial treatment of HIV-1 infection in resource-limited countries. ATV+DDI+FTC had inferior efficacy and is not recommended as an initial antiretroviral regimen.

**Trial Registration:**

www.ClinicalTrials.gov NCT00084136

*Please see later in the article for the Editors' Summary.*

## Abstract 2

Thomas Campbell and colleagues report findings of a randomized trial conducted in multiple countries regarding the efficacy of antiretroviral regimens with simplified dosing.

## Editors' Summary

**Background:**

Despite the enormous gains in reducing HIV-related illness and death over the past decade, there are still considerable challenges to meeting the global goal of universal access to highly active antiretroviral treatment—a combination of effective drugs that attack the HIV virus in various ways—to everyone living with HIV/AIDS who could benefit from treatment. In recognition of the related financial, technical, and system obstacles to providing universal access to HIV treatment, in 2010 the UN agency responsible for HIV/AIDS—UNAIDS—launched an ambitious plan called Treatment 2.0, which aims to simplify the way HIV treatment is currently provided. One of the main focuses of Treatment 2.0 is to simplify drug regimes for the treatment of HIV and to make treatment regimes less toxic. In line with Treatment 2.0, the World Health Organization currently recommends that antiretroviral regimens for the initial treatment of HIV should include two nucleoside reverse transcriptase inhibitors (zidovudine or tenofovir disoproxil fumarate [DF] with lamivudine or emtricitabine) and a non-nucleoside reverse transcriptase inhibitor (efavirenz or nevirapine.)

**Why Was This Study Done?:**

Most of the evidence about the safety and effectiveness of clinical trials come from clinical trials in high-income countries and thus is not generally representative of the majority of people with HIV. So in this study, the researchers conducted a randomized controlled trial in diverse populations in many different settings to investigate whether antiretroviral regimens administered once daily were as effective as twice-daily regimens and also whether a regimen containing the drug atazanavir administered once daily was as safe and effective as a regimen containing efavirenz—data from previous studies have suggested that atazanavir has characteristics, such as its side effect profile, which may make it more suitable for low income settings.

**What Did the Researchers Do and Find?:**

The researchers recruited eligible patients from centers in Brazil, Haiti, India, Malawi, Peru, South Africa, Thailand, the United States, and Zimbabwe—almost half (47%) were women. Then the researchers randomly assigned participants to one of three regimens: efavirenz 600 mg daily plus co-formulated lamivudine-zidovudine 150 mg/300 mg twice daily (EFV+3TC-ZDV); or atazanavir 400 mg once daily, plus didanosine-EC 400 mg once daily, plus emtricitabine 200 mg once daily (ATV+DDI+FTC); or efavirenz 600 mg once daily plus coformulated emtricitabine-tenofovir-DF 200 mg/300 mg once daily (EFV+FTC-TDF). During the study period ATV+DDI+FTC was found to be inferior to EFV+3TC-ZDV, so the Multinational Data Safety Monitoring Board ordered this arm of the trial to stop. Then a year later, due to the low number of treatment failures (deaths, severe HIV disease, or serious opportunistic infections) in the remaining two arms, the board advised the trial to stop early. So the researchers analyzed the data obtained up to this point and pooled the results from all of the centers.

The researchers found that during an average of 184 weeks of follow-up, there were 95 treatment failures (18%) among 526 participants taking EFV+FTC-TDF compared to 98 failures among 519 participants taking EFV+3TC-ZDV. During an average 81 weeks follow-up, there were 108 failures (21%) among 526 participants assigned to ATV+DDI+FTC and 76 (15%) among 519 participants assigned to EFV+3TC-ZDV. As for safety, 243 (46%) participants assigned to EFV+FTC-TDF reached a safety endpoint (grade 3 disease, abnormal lab measurement, or the need to change drug) compared to 313 (60%) in the EFV+3TC-ZDV group. Importantly, the researchers found that there was greater risk of safety events for women assigned to EFV+3TC-ZDV and also that the atazanavir-based regimen had a higher relative efficacy in women compared to men.

**What Do These Findings Mean?:**

These findings suggest that in diverse populations, EFV+FTC-TDF is as effective as EFV+3TC-ZDV but importantly, the once-daily dosing of EFV+FTC-TDF makes this regimen useful for the initial treatment of HIV, especially in low-income countries. Therefore, as per the guidance in Treatment 2.0, EFV+FTC-TDF in a single combination tablet that can be taken once a day is an attractive option. These findings also indicate that as ATV+DDI+FTC was found to be inferior to the other regimens, this combination should not be used in the initial treatment of HIV. These findings also add to the evidence that antiretroviral efficacy and safety can differ between women and men and support further development of sex-specific recommendations for antiretroviral regimen options.

**Additional Information:**

Please access these Web sites via the online version of this summary at http://dx.doi.org/10.1371/journal.pmed.1001290.

- The UNAIDS website has more information about Treatment 2.0; and the WHO website provides technical information
- For an introduction to the treatment of HIV/AIDS see http://www.avert.org/treatment.htm; the AVERT site also has personal stories from women living with HIV/AIDS
- AIDSmap provides information for individuals and communities affected by HIV/AIDS
- The ACTG website provides information about research to improve treatment of HIV and related complications

## Introduction

Increased effectiveness of HIV-1 treatment through optimizing antiretroviral regimens for simplification and reduced toxicity is a priority in the recent UNAIDS Treatment 2.0 initiative [1],[2]. Treatment 2.0 emphasizes that effective antiretroviral regimens with simplified dosing, fewer side effects, and lower long-term toxicity are needed to minimize requirements for laboratory monitoring and maximize the efficiency of antiretroviral delivery. However, most existing knowledge of antiretroviral safety and efficacy comes from clinical trials in high-income countries with study populations not representative of the global diversity of people infected with HIV-1. Prospective comparisons of antiretroviral efficacy and safety in diverse multinational settings with representative proportions of women are needed to better inform the choice of antiretroviral drugs for initial HIV-1 treatment.

World Health Organization (WHO, 2010 revision) guidelines recommend initiation of antiretroviral therapy with two nucleoside reverse transcriptase inhibitors (NRTI) (zidovudine [ZDV] or tenofovir disoproxil fumarate [DF] with lamivudine [3TC] or emtricitabine [FTC) and a non-nucleoside reverse transcriptase inhibitor (NNRTI) (efavirenz [EFV] or nevirapine) [3]. Randomized clinical trials conducted in developed countries provide evidence that these regimens are safe and effective [4]–[6]. Although a regimen of FTC, tenofovir-DF (TDF), and EFV meets criteria outlined in Treatment 2.0 including low toxicity and simplified once-daily dosing, the comparative safety and efficacy of this regimen in low-resource settings is unknown.

Compared to EFV, the HIV-1 protease inhibitor atazanavir (ATV) lacks known teratogenicity and is active against NNRTI-resistant virus. These features are potentially advantageous for use of ATV in resource-limited settings where use of single dose nevirapine for prevention of mother-to-child transmission of HIV-1 could increase the risk of NNRTI-resistant virus in women and their sexual partners. Previous studies of antiretroviral naïve persons provide evidence that ATV without ritonavir boosting is safe and efficacious: Unboosted ATV had similar efficacy compared to EFV when given with co-formulated 3TC-ZDV [7], similar efficacy compared to ritonavir-boosted ATV when given with extended release stavudine and 3TC [8], and comparable activity to nelfinavir when given with didanosine and stavudine [9]. Previous studies also provide evidence that, when given with EFV, the NRTI combination of didanosine and FTC (or 3TC) is safe and efficacious and has comparable activity to 3TC-ZDV and stavudine plus 3TC [10]–[14]. Thus, taken together available data predict that a regimen of ATV, didanosine, and FTC would have antiviral efficacy comparable to 3TC-ZDV and EFV; however, direct comparisons of these two regimens have not been performed previously.

## Methods

### Study Design and Participants

The Prospective Evaluation of Antiretrovirals in Resource Limited Settings (PEARLS) study of the AIDS Clinical Trials Group (ACTG) evaluated two hypotheses: (1) Antiretroviral regimens administered once daily are non-inferior to twice-daily regimens; (2) A regimen containing ATV administered once daily without ritonavir boosting is non-inferior to an EFV-based regimen. Study design details are available at ClinicalTrials.gov NCT00084136 and in the study protocol provided in Text S1. The CONSORT checklist used for preparation of this manuscript is provided in Text S2.

Enrollment was limited to the following ACTG international sites: Instituto de Pesquisa Clinica Evandro Chagas, Rio de Janeiro, Brazil; Hospital Nossa Senhora da Conceicao-GHC, Porto Alegre, Brazil; Les Centres GHESKIO, Port-au-Prince, Haiti; YRG Centre for AIDS Research & Education, Chennai, India; National AIDS Research Institute, Pune, India; College of Medicine Clinical Research Site, Blantyre, Malawi; Kamuzu Central Hospital, Lilongwe, Malawi; Asociacion Civil Impacta Salud y Educacion - Miraflores and San Miguel Clinical Research Site, Lima, Peru; Durban Adult HIV Clinical Research Site, Durban, South Africa; University of Witwatersrand Clinical HIV Research Unit, Johannesburg, South Africa; Research Institute for Health Sciences, Chiang Mai, Thailand; and Parirenyatwa Hospital Clinical Research Center, Harare, Zimbabwe. All ACTG sites in the United States were also eligible to enroll participants. Enrollment in the US was limited to no more than 18% of total; the remaining enrollment was distributed equally across the international sites with an option for international sites to request additional enrollment once their initial quota of 100 participants was filled.

Eligible participants were ≥18 y, had documented HIV-1 infection, CD4+ lymphocytes <300 cells/µl, and ≤7 d of cumulative antiretroviral therapy prior to study entry. Persons with absolute neutrophils <750/µl, hemoglobin <7.5 g/dl, calculated creatinine clearance <60 ml/min, or aspartate transaminase or alanine transaminase greater than 5-fold above the upper limit of normal were excluded. Women of reproductive potential were non-pregnant and, if participating in sexual activity that could lead to pregnancy, agreed to use contraception (two forms if taking EFV). Persons with serious chronic, acute, or recurrent infections had completed ≥14 d of therapy and were clinically stable.

### Randomization

Sites enrolled participants through a centralized web-based system. The ACTG Data Management Center (Frontier Science & Technology Research Foundation) randomly assigned participants 1∶1∶1 to an open-label regimen of EFV 600 mg daily plus co-formulated 3TC-ZDV 150 mg/300 mg twice daily (EFV+3TC-ZDV); or ATV 400 mg once daily with food, plus didanosine-EC (DDI) 400 mg once daily taken on an empty stomach 1 h before or 2 h after the ATV dose, plus FTC 200 mg once daily (ATV+DDI+FTC); or EFV 600 mg once daily plus co-formulated FTC-TDF 200 mg/300 mg once daily (EFV+FTC-TDF). Permuted block randomization was stratified by country (nine levels) and screening plasma HIV-1 RNA (<100,000 versus ≥100,000 copies/ml). Treatment assignment was revealed after successful enrolment at the local site on the web-based system.

### Procedures

A targeted physical exam, medication review, adherence interview and pill counts, serum chemistries, liver function tests, pregnancy test, CD4+ lymphocyte count, and plasma HIV-1 RNA were scheduled at least every 8 wk. All study drug modifications including initial doses, participant-initiated and/or protocol-mandated interruptions, substitutions, and permanent discontinuation and reasons for modification were assessed at each visit. Adverse events (signs, symptoms, and laboratory results) used US Division of AIDS (DAIDS) scale for severity grading [15]. Diagnosis criteria were standardized across sites using ACTG Appendix 60 (see Text S3). Plasma HIV-1 RNA was measured in real time by the Roche Amplicor Monitor assay (v1.5) at laboratories participating in the DAIDS Virology Quality Assurance program.

### Outcomes

The primary efficacy endpoint (treatment failure) was time from randomization to first occurrence of any of the following: (1) death; (2) HIV-1 disease progression defined as new or recurrent WHO stage 4 diagnosis (excluding HIV-1-associated nephropathy or cardiomyopathy) [3], Chagas' diseases, or chronic microsporidiosis or cyclosporidiosis occurring at least 12 wk following randomization and not part of immune reconstitution inflammatory syndrome (IRIS); or (3) virologic failure defined as two successive measurements of plasma HIV-1 RNA≥1,000 copies/ml, with the first measurement at the week 16 visit or later (≥14 wk after randomization), regardless of study treatment history or status (intention-to-treat). Participants who did not meet primary endpoint criteria at any time were censored at the last study visit at which plasma HIV-1 RNA was measured. Disease progression and IRIS events were reviewed and adjudicated by a panel of five physician team members who were blinded to participant identity, clinic site, demographic characteristics, and study treatment. Study treatment information was also ignored for the mortality and HIV-1 disease progression components, so the analysis for the primary efficacy endpoint was fully intent-to-treat. A post hoc sensitivity analysis that explored whether crossover could explain observed results was also performed.

The primary safety endpoint was the earliest of the following times: date of onset of grade ≥3 (at least one grade higher than entry) sign/symptom, date of specimen collection of a grade ≥3 (at least one grade higher than entry) laboratory abnormality, or date of last dose of randomized study treatment before any modification to that treatment (change in drug dosage, addition of another antiretroviral drug, or discontinuation of any component of the randomized antiretroviral regimen). Elevated serum bilirubin concentration was excluded from the laboratory abnormality component of this endpoint only, because it is usually asymptomatic and not associated with known adverse outcomes. Any signs, symptoms, or changes in antiretroviral therapy that resulted from elevated bilirubin were captured in the other components of the composite safety endpoint. Participants who did not meet the safety endpoint definition were censored at the earlier of the last study visit or final medication dose. Because study treatment modification was part of the composite primary safety endpoint, this analysis was necessary as-treated.

First antiretroviral regimen discontinuation was time to premature discontinuation of study participation, failure to take antiretrovirals for ≥8 consecutive wk, or switch to another antiretroviral regimen. Prespecified antiretroviral substitutions not included in the definition of regimen discontinuation were as follows: Substitutions of stavudine for ZDV were not counted as endpoints in this analysis because WHO guidelines (2003 revision) when PEARLS was implemented listed 3TC-ZDV as the initial recommendation for the nucleoside analog component of an antiretroviral regimen with substitution of other nucleosides, including stavudine, if needed. In 2006 the protocol was modified to include TDF for ZDV as a prespecified non-endpoint in response to the 2006 revision of the WHO guidelines that listed ZDV and TDF as the preferred initial nucleoside analog reverse transcriptase inhibitors to be combined with 3TC or FTC. Substitutions of DDI for TDF and TDF for DDI were not counted as endpoints because both drugs are once daily nucleoside analogs. Likewise, substitutions of nevirapine for EFV were prespecified as non-endpoints because both drugs are in the NNRTI class and can be dosed once daily.

Plasma HIV-1 RNA below lower quantitation limit (<400 copies/ml) was a secondary endpoint that used the closest value to the scheduled visit. Another secondary endpoint, time to loss of virologic response (TLOVR) included an analysis as specified in US Food and Drug Administration (FDA) guidelines where all antiretroviral substitutions were counted as endpoints [16], and an analysis where the prespecified antiretroviral substitutions did not count as endpoints. Immunologic failure was defined as CD4+ lymphocytes <100 cells/µl at 48 wk or later. Those not meeting the immunologic failure secondary endpoint were censored at the study visit week of last CD4+ lymphocyte count.

### Sample Size

Assumptions included a non-inferiority threshold hazard (relative risk ratio) of 1.35, overall 30% treatment failure rate within the two arms compared under the alternative of equivalence (hazard ratio [HR] = 1.0), and one-sided significance ≤0.05. The estimated statistical power for the primary efficacy comparison by a one-sided log-rank test comparison of ATV and EFV+FTC-TDF arms to EFV+3TC-ZDV was 80% for a sample size of 456 per arm with inflation by 10% to account for losses to follow-up. The study did not have a fixed follow-up duration, but was planned to continue until 30% of participants experienced a primary efficacy endpoint.

### Study Oversight and Monitoring

The study was approved by the institutional review boards and ethics committees at each participating institution. Written, informed consent was obtained from study participants, and human experimentation guidelines of the US Department of Health and Human Services were followed.

The US National Institute of Allergy and Infectious Diseases (NIAID) Multinational Data Safety Monitoring Board (DSMB) reviewed safety and efficacy at least yearly. The prespecified stopping guidelines were only for early evidence of inferiority of an experimental arm, based upon Haybittle-Peto bounds. On 6 May 2008, ATV+DDI+FTC was found to be inferior to EFV+3TC-ZDV for the primary efficacy endpoint at median follow-up of 72 wk. The HR for time to regimen failure was 1.67 (99.98% CI 1.0–2.75; *p* = 0.001), reflecting 104 failures in the ATV+DDI+FTC arm compared to 67 failures in the EFV+3TC-ZDV arm. These CIs reflect those data available at interim review by the DSMB, which prespecified 99.98% intervals to correspond to *p* = 0.001 Haybittle-Peto bounds for superiority (and likewise, inferiority). Study participants, investigators, institutional review boards, and ethics committees were informed of the DSMB findings on 23 May 2008, and participants still taking ATV+DDI+FTC were switched to alternative antiretroviral regimens. The DSMB did not report any findings related to the comparison of the EFV+FTC-TDF and EFV+3TC-ZDV arms so these arms were not modified and the investigators remained blinded to outcomes in those arms until completion of all study follow-up.

On 3 November 2009, the DSMB concluded that the remarkably low rate of new endpoints in the EFV+FTC-TDF and EFV+3TC-ZDV arms made it unlikely that the study would reach the planned 30% rate of primary endpoint within 2 y (or even considerably longer) and continuation of study follow-up for two more years would likely improve precision of the comparison by only a small amount. The DSMB recommended that “it was simply not practical to continue until 274 events (30%) and that no statistical penalty needed to be paid for stopping before then.” The ACTG followed the DSMB recommendations and closeout visits were conducted between 1 April and 31 May 2010.

### Statistical Methods

Analyses for the comparison of ATV+DDI+FTC to EFV+3TC-ZDV used data collected from 1 May 2005 through 22 May 2008. Analyses for comparison of EFV+FTC-TDF to EFV+3TC-ZDV used data collected through 31 May 2010. The study was designed to test the primary efficacy hypothesis of non-inferiority using an upper bound of a one-sided, 0.05 level interval. Early study closure prompted a revised analysis plan for pairwise comparison. Since comparison of the ATV+DDI+FTC and EFV+3TC-ZDV arms used additional data collected between 4 March to 22 May 2008, two-sided 95% CIs and associated *p*-values are presented here. As the closure of the EFV+FTC-TDF and EFV+3TC-ZDV arms was not due to the prespecified stopping guidelines, we followed the DSMB recommendations on significance level and focused inferential procedure for the primary efficacy outcome on estimating treatment difference effect size and its related range of plausible values, rather than hypothesis testing. Specifically, two-sided 95% CIs for treatment difference (parameterized as the relative difference by HR) were provided. Parallel methods were used for secondary efficacy outcomes (for consistency) and safety outcomes (by original plan). *p*-Values based upon stratified log-rank test with a null hypothesis of no difference between randomized arms were provided only for secondary efficacy and safety outcomes. Time-to-event outcome distributions were summarized by the method of Kaplan and Meier, and compared between randomized groups by log-rank test stratified by randomized allocation. HRs were estimated from Cox proportional hazards regression; HR variation over time was based on a test for interaction between treatment group and time. Cumulative probabilities of time-to-event endpoints used Greenwood estimates of variation for CI formulation. For each of the primary efficacy and safety time-to-event outcomes, the proportional hazards assumption was tested through introducing an interaction term between treatment group and time, and was not rejected in any case (all *p*>0.18). Interactions between study treatment and pretreatment covariates were tested individually by Cox regression. Estimated binomial proportions were compared between arms using Fisher exact test and 95% exact CIs. Comparisons of CD4+ cells over time used a one-sided, 0.025-level Wei-Johnson test [17].

## Results

### Study Participants

**Figure 1:**
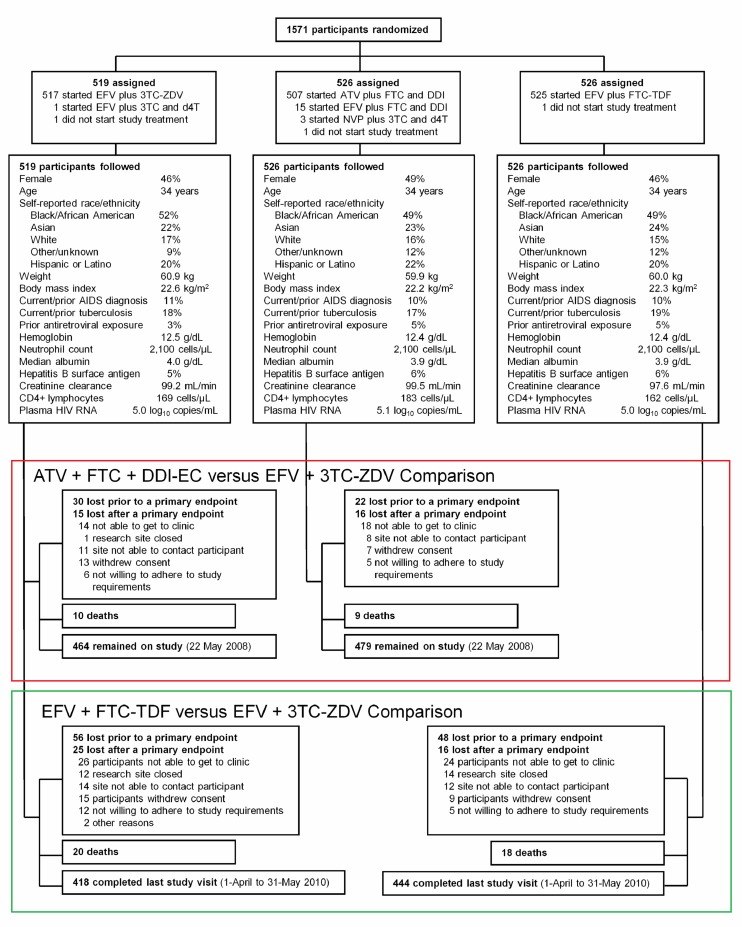
Flow diagram for participant outcomes. The outcomes of all participants randomized to the three arms are provided. The most common prior antiretroviral exposure was for prevention of mother-to-child transmission of HIV-1 with either ZDV monotherapy (19 women; median duration 32 d; intraquartile range 30–60 d) or a single dose of nevirapine in the peripartum period (16 women). Continuous variable values are the median for the treatment arm. Creatinine clearance was calculated by Crockoft-Gault equation. Follow-up visits were conducted for 1,571 participants. ATV plus FTC and DDI follow-up was terminated on 22 May 2008 in response to the DSMB recommendation and comparison of ATV plus FTC and DDI to EFV plus 3TC-ZDV used data available up to the time of the ATV arm closure (red box); median follow-up 81 wk. Comparison of EFV plus FTC-TDF to EFV plus 3TC-ZDV used all follow-up data for participants in these arms through 31 May 2010 (green box); median follow-up 184 wk.

Between May 2005 and July 2007, 1,571 participants were randomized to one of the three treatment arms (Figure 1): Brazil (*n* = 31), Haiti (*n* = 100), India (*n* = 255), Malawi (*n* = 221), Peru (*n* = 134), South Africa (*n* = 210), Thailand (*n* = 100), US (*n* = 210), and Zimbabwe (*n* = 110). There were 739 women (47%) and 787 (50%) participants were Black or African American. There were 434 ongoing infections at study entry (Table S1) that were treated according to local standard of care. The five most common ongoing infections were oropharyngeal candidiasis (77 cases), pulmonary tuberculosis (73 cases), mucocutaneous herpes simplex virus (65 cases), anogenital warts (56 cases), and other or vulvovaginal candidiasis (44 and 32 cases, respectively). Together, these six diagnoses accounted for 80% of the ongoing infections at study entry.

### Follow-up

Outcomes are summarized in Figure 1; 99% of expected study visits were completed. Median follow-up was 81 wk for comparison of ATV+DDI+FTC to EFV+3TC-ZDV and 184 wk for the comparison of EFV+FTC-TDF to EFV+3TC-ZDV. There were 47 deaths (3%) and 183 (12%) participants did not complete study follow-up; 126 (8%) left prior to regimen failure. The risk ratio of any premature study discontinuation for participants allocated to ATV+DDI+FTC versus EFV+3TC-ZDV was 0.80 (CI 0.54–1.18; *p* = 0.26) and for EFV+FTC-TDF versus EFV+3TC-ZDV was 0.79 (CI 0.59–1.06; *p* = 0.12). The primary endpoint analyses included all 1,571 participants according to randomized treatment assignment.

### Efficacy of ATV Plus DDI and FTC

**Figure 2:**
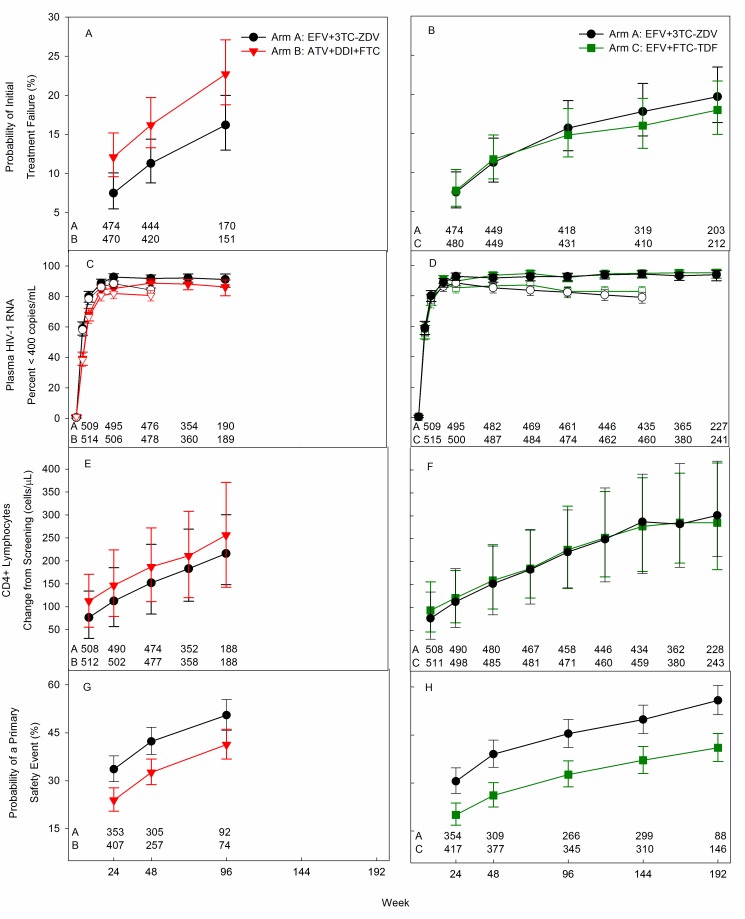
Efficacy and safety of randomized study treatment over time. (A–H) black circles, EFV plus 3TC-ZDV; red triangles, ATV plus DDI-EC and FTC; green squares, EFV plus FTC-TDF. (A–B) Estimated cumulative probability of antiretroviral regimen failure defined by the protocol-specified primary efficacy endpoint: comparison of EFV plus 3TC-ZDV to ATV plus FTC and DDI (A) and EFV plus FTC-TDF (B). (C–D) Proportion of participants with plasma HIV-1 RNA less than 400 copies/ml for comparison of EFV plus 3TC-ZDV to ATV plus FTC and DDI (C) and EFV plus FTC-TDF (D). These comparisons included all randomized study participants according to assigned study treatment. The analysis that counted missing values as greater than 400 copies/ml (open symbols) is truncated at the maximum potential duration of study follow-up for participants who entered the study at the end of the enrollment period (144 wk). (E–F) Median change in CD4+ lymphocyte count from screening value over time for comparison of EFV plus 3TC-ZDV to ATV plus FTC and DDI (E) and EFV plus FTC-TDF (F). (G–H) Estimated cumulative probability a safety endpoint over time for comparison of EFV plus 3TC-ZDV to ATV plus FTC and DDI (G) and EFV plus FTC-TDF (H). For (A–D, G and H), bars represent the 95% CI for the estimate. For (E–F), bars represent the interquartile range. (A–H) The number of evaluable participants at each time point is provided for each randomized treatment assignment.

**Figure 3:**
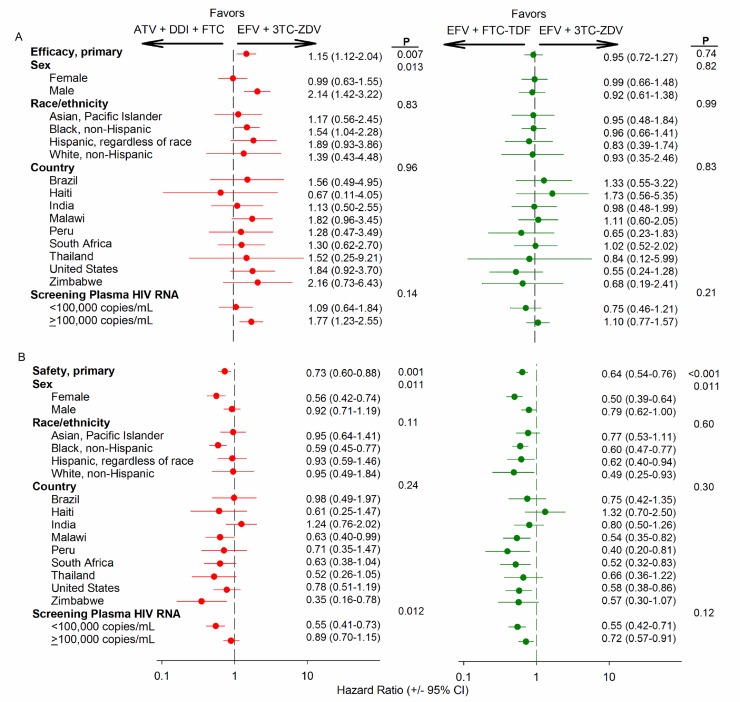
Subgroup analysis for primary efficacy and safety endpoints by randomly assigned antiretroviral treatment. Subgroup analyses were conducted for the baseline covariates self-reported sex and race/ethnicity and the countries in which the participating research sites were located. The relative risk and 95% CIs are provided for all participants (overall) and for each subgroup. *p*-Value represents interaction test between baseline covariate and randomized treatment group. Comparisons between ATV plus DDI and FTC and EFV plus 3TC-ZDV are in red. Comparisons between EFV plus FTC-TDF and EFV plus 3TC-ZDV are in green. (A) Treatment failure (efficacy) composite endpoint. (B) Safety events composite endpoint.

Risk of treatment failure primary endpoint was greater for participants assigned to ATV+DDI+FTC compared to EFV+3TC-ZDV with 108 (20.5%) versus 76 (14.6%) failures, respectively (Table 1). The between-arm difference in primary endpoint failure rates persisted over time (Figure 2A). The most common cause of treatment failure was confirmed plasma HIV-1 RNA ≥1,000 copies/ml (82% of primary endpoints). The lower bounds of the 95% CIs for the relative hazard of both treatment and virologic failure, but not disease progression and death, for comparison of the ATV and EFV+3TC-ZDV arms excluded 1.0 (Table 1). 30 disease progression events (15 in the ATV+DDI+FTC arm and 15 in the EFV+3TC/ZDV arm) did not meet the definition of treatment failure because of either being diagnosed within the first 12 wk of study follow-up (15 events) and/or being part of an IRIS event (15 events). Men randomized to ATV+DDI+FTC had higher risk of treatment failure compared to men randomized to EFV+3TC-ZDV (HR 2.14, CI 1.42–3.42) but a difference in regimen efficacy was not detected in women (Figure 3A, left side). No significant statistical interactions between treatment effect and race and ethnicity, country, or viral load stratum were observed.

**Table 1:** Primary and secondary time-to-event outcomes for the comparison of atazanavir plus didanosine-EC and emtricitabine to efavirenz plus lamivudine-zidovudine using data collected through 22 May 2008.

Study Endpoint	*n* Events		HR (95% CI)^a^	*p*-Value^b^	Events per 100 Person-Years (95% CI)	
	ATV+DDI+FTC	EFV+3TC-ZDV			ATV+DDI+FTC	EFV+3TC-ZDV
Treatment failure (composite endpoint)	108	76	1.51 (1.12–2.04)	0.007	13.3 (11.0–16.1)	8.9 (7.1–11.2)
All death^c^	9	10	0.88 (0.36–2.17)	0.78	1.0 (0.5–1.9)	1.1 (0.6–2.0)
All initial HIV-1 disease progression^d^^,^ e	18	10	1.80 (0.83–3.90)	0.14	2.0 (1.3–3.2)	1.1 (0.6–2.1)
All initial confirmed virologic failure^e^^,^ f	92	63	1.56 (1.12–2.16)	0.008	11.2 (9.1–13.7)	7.3 (5.7–9.4)
Safety events (composite endpoint)^e^^,^ g	210	252	0.73 (0.60–0.88)	0.001	30.8 (26.9–35.2)	43.0 (38.0–48.6)
All initial antiretroviral dose modifications^e^^,^ h	149	172	0.80 (0.65–1.00)	0.05	9.9 (8.4–11.6)	12.2 (10.5–14.2)
All initial grade 3 or 4 signs or symptoms^d^^,^ e	69	98	0.66 (0.48–0.90)	0.008	8.2 (6.5–10.4)	12.6 (10.3–15.3)
All initial grade 3 or 4 laboratory abnormalities^d^^,^ e ^,^ g	76	119	0.58 (0.43–0.78)	0.0003	9.2 (7.3–11.5)	16.1 (13.4–19.3)
First antiretroviral regimen discontinuation^i^	149	103	1.57 (1.22–2.01)	0.0005	9.9 (8.4–11.6)	6.2–(5.1–7.5)
Immunologic failure^j^	19	23	0.82 (0.44–1.52)	0.53	2.1 (1.3–3.3)	2.5 (1.7–3.8)

a Also known as relative risk. Estimated from Cox regression model stratified by both country and RNA stratum and including randomized treatment group as sole covariate.

b *p*-Value calculated from stratified log-rank test between arms.

c The five most common causes of death were infection (six deaths), liver disease (three deaths), malignancy (two deaths), suicide (two deaths), and unknown cause (two deaths).

d Disease progression diagnoses are in Table S2; grade 3 and 4 laboratory events in Table S3; and signs and symptoms in Table S4.

e All events meeting these criteria are reported; some participants met criteria for multiple endpoints.

f Confirmed plasma HIV RNA≥1,000 copies/ml at study week 16 or later.

g Elevated bilirubin concentration not included.

h Change in any component of initial randomized antiretroviral regimen.

i The following antiretroviral substitutions were prespecified and were not included in this endpoint: TDF for DDI, stavudine or TDF for ZDV, or nevirapine for EFV.

j CD4+ lymphocytes <100/µl at week 48 or later.

Plasma HIV-1 RNA was below 400 copies/ml in 82% of participants randomized to ATV+DDI+FTC versus 88% randomized to EFV+3TC-ZDV at 24 wk (*p* = 0.004) (Figure 2C). In the FDA TLOVR analysis disallowing any antiretroviral substitution, there was no difference between treatment arms at 48 wk (135 versus 149; *p* = 0.3). In the TLOVR analysis that did not penalize for prespecified antiretroviral drug substitutions, the number of endpoints was greater for ATV+DDI+FTC compared to EFV+3TC-ZDV at 48 wk (135 versus 85; *p*<0.001).

Risk of immunologic failure was low and did not differ between arms (Table 1). CD4+ lymphocyte increases from baseline were 187/µl and 152/µl in the ATV+DDI+FTC and EFV+3TC-ZDV arms, respectively, at 48 wk and were significantly greater in ATV+DDI+FTC at all time points evaluated (all individual *p*-values<0.05; one-sided test over 96 wk, *p*<0.001) (Figure 2E).

### Regimen Discontinuation for ATV Plus DDI and FTC

Initial antiretroviral regimen discontinuation was due to non-prespecified drug substitutions (61% of all observed discontinuations), premature discontinuation of study follow-up (30%), permanent discontinuation of all antiretroviral therapy (8%), and temporary discontinuation of all antiretroviral therapy for more than 8 wk (1%). Risk of this endpoint, when protocol-specified drug substitutions were not counted, was significantly greater among participants randomized to ATV+DDI+FTC (Table 1). The most common reasons for non-prespecified drug substitutions among persons randomized to ATV+DDI+FTC were virologic failure (40 cases), tuberculosis treatment (28 cases), clinical adverse events (23 cases), and laboratory abnormalities (10 cases).

### Safety of ATV Plus DDI and FTC

Excluding hyperbilirubinemia, which is an expected effect of ATV treatment, there were fewer safety endpoints among participants randomized to ATV+DDI+FTC compared to EFV+3TC-ZDV (Figure 2G; Table 1). Estimated probability of a safety endpoint by week 48 was 32.6% (CI 28.8%–36.8%) versus 42.3% (CI 38.2–46.7%). There was a significant interaction between study treatment and both sex and plasma HIV-1 RNA strata for the primary safety endpoint (*p* = 0.01 for both) (Figure 3B, left side). Women randomized to ATV+DDI+FTC had lower risk of a safety endpoint compared to women randomized to EFV+3TC-ZDV (HR 0.56, CI 0.42–0.74). Among men, risk difference for the primary safety endpoint between arms was attenuated (HR 0.92, CI 0.71–1.19). The risk of a safety endpoint for the lower versus the upper plasma HIV-1 RNA strata were 0.55 (CI 0.41–0.73) and 0.89 (CI 0.70–1.15), respectively. There were no significant interactions between race or country and assigned treatment for the primary safety endpoint.

There were fewer initial dose modifications among participants randomized to ATV+DDI+FTC compared to EFV+3TC-ZDV (Table 1). The estimated cumulative probability of any dose modification of the assigned antiretroviral regimen at 48 wk was 20.7% (CI 17.5%–24.4%) compared to 25.7% (CI 22.1%–29.7%), respectively. Excluding hyperbilirubinemia, there were fewer severe or potentially life-threatening laboratory abnormalities in ATV+DDI+FTC (Tables 1 and S3); the estimated cumulative probability at 48 wk was 12.4% (CI 9.9%–15.6%) compared to 21.0% (CI 17.7%–24.8%). There was a lower risk of a severe or life-threatening sign or symptom in the ATV+DDI+FTC arm (Tables 1 and S4). At 48 wk the cumulative probabilities of a new severe or life-threatening sign or symptom were 10.5% (CI 8.1%–13.4%) and 16.5% (CI 13.5%–20.0%) for the ATV+DDI+FTC and EFV+3TC-ZDV arms, respectively.

Neurological symptoms, cachexia/weight loss, and dermatologic symptoms occurred in 13 (2%), 5 (1%), and 8 (2%) participants assigned to ATV+DDI+FTC, respectively, compared to 22 (4%), 17 (3%), and 15 (3%) participants assigned to EFV+3TC-ZDV (Table S3). Participants assigned to ATV+DDI+FTC were more likely to have a new diagnosis of serious renal disease (19 [4%] versus 5 [1%] participants; nominal *p* = 0.006) (Tables S5 and S6).

### Efficacy of EFV+FTC-TDF

There were 95 (18.0%) and 98 (18.8%) treatment failures in the EFV+FTC-TDF and EFV+3TC-ZDV arms, respectively, and the range of the relative risk difference was 0.72 to 1.27 (Table 2). Treatment failure relative risk did not change significantly over time (*p* = 0.9) (Figure 2B). There were no significant statistical interactions between antiretroviral regimen treatment effect and sex, race and ethnicity, country, or viral load stratum (Figure 3A, right side). The most common cause of regimen failure was confirmed plasma HIV-1 RNA≥1,000 copies/ml (81% of all primary endpoints). The range of the relative risk difference for virologic failure was 0.72 to 1.36 (Table 2). Of 156 initial virologic failures, 64 (41%) and 125 (80%) occurred within the first 24 wk and 96 wk of follow-up, respectively. There were no significant differences in the risk of HIV-1 disease progression or death between arms. 25 disease progression events (15 in the EFV+FTC/TDF arm and ten in the EFV+3TC/ZDV arm) did not meet the definition of the treatment failure endpoint due to either being diagnosed within the first 12 wk of study follow-up (12 events) and/or being part of an IRIS event (13 events).

**Table 2:** Primary and secondary time-to-event outcomes for comparison of efavirenz plus emtricitabine-tenofovir-DF to efavirenz plus lamivudine-zidovudine using data collected through 31-May-2010.

Study Endpoint	Number of Events		Hazard Ratio (95% CI)^a^	*p*-Value^b^	Events per 100 Person-Years (95% CI)	
	EFV+FTC-TDF	EFV+3TC-ZDV			EFV+FTC-TDF	EFV+3TC-ZDV
Treatment failure (composite endpoint)	95	98	0.95 (0.72–1.27)	NA	5.4 (4.4–6.7)	5.8 (4.7–7.0)
All death^c^	18	20	0.90 (0.48–1.70)	0.74	0.9 (0.6–1.5)	1.1 (0.7–1.7)
All initial HIV-1 disease progression^d^^,^ e	11	12	0.89 (0.39–2.01)	0.77	0.6 (0.3–1.0)	0.7 (0.4–1.1)
All initial confirmed virologic failure^e^^,^ f	78	78	0.99 (0.72–1.36)	0.95	4.4 (3.6–5.5)	4.5 (3.6–5.7)
Safety events (composite endpoint)	243	313	0.64 (0.54–0.76)	<0.0001	17.6 (15.5–19.9)	28.7 (25.7–32.0)
All initial antiretroviral dose modifications^e^^,^ g	140	222	0.54 (0.44–0.67)	<0.0001	8.1 (6.9–9.6)	15.6 (13.7–17.8)
All initial grade 3 or 4 signs or symptoms^d^^,^ e	115	116	0.96 (0.74–1.24)	0.73	6.9 (5.8–8.3)	7.4 (6.1–8.8)
All initial grade 3 or 4 laboratory abnormalities^d^^,^ e	98	154	0.55 (0.43–0.71)	<0.0001	5.8 (4.8–7.1)	10.8 (9.2–12.6)
First antiretroviral regimen discontinuation^h^	125	147	0.83 (0.65–1.05)	0.12	7.1 (5.9–8.4)	8.6 (7.4–10.2)
Immunologic failure^i^	33	30	1.08 (0.66–1.79)	0.75	1.8 (1.3–2.5)	1.6 (1.1–2.3)

a Also known as relative risk. Estimated from Cox regression model stratified by both country and RNA stratum and including randomized treatment group as sole covariate.

b *p*-Value calculated from stratified log-rank test between arms. Not applicable (NA) because no formal hypothesis testing was performed based on DSMB recommendations.

c The five most common causes of death were infection (17 deaths) and unknown cause (five deaths) followed by suicide, trauma, and stroke (three deaths each).

d Disease progression diagnoses are in Table S7; grade 3 and 4 laboratory adverse events in Table S8; and signs and symptoms in Table S9.

e All events meeting these criteria are reported; some participants met criteria for multiple endpoints.

f Confirmed plasma HIV RNA≥1,000 copies/ml at study week 16 or later.

g Change in any component of initial randomized antiretroviral regimen.

h The following antiretroviral substitutions were prespecified and were not included in this endpoint: stavudine or TDF for ZDV, nevirapine for EFV, or didansoine for TDF.

i CD4+ lymphocytes <100/µl at week 48 or later.

There were no differences between EFV+FTC-TDF and EFV+3TC-ZDV for plasma HIV-1 RNA<400 copies/ml at 24 and 48 wk (*p* = 0.12 and 0.60, respectively; missing imputed as ≥400 copies/ml) and the kinetics of attaining and maintaining plasma HIV-1 RNA suppression were similar in both arms over time (Figure 2D). In the FDA TLOVR analysis disallowing any antiretroviral substitution there were fewer events in EFV+FTC-TDF arm at 48 wk (99 versus 153; *p*<0.001) and 96 wk (124 versus 186; *p*<0.001). In the TLOVR analysis that did not penalize for prespecified antiretroviral drug substitutions, there was no difference between treatment arms at 48 or 96 wk (86 events in each arm, *p* = 0.99 and 111 versus 116 events; *p* = 0.74, respectively).

Risk of immunologic failure was low (Table 2). Median absolute CD4+ lymphocytes increased from 167 cells/µl at screening to 452 cells/µl at 192 wk and there was no significant difference between the EFV+FTC-TDF and EFV+3TC-ZDV arms over time (one-sided *p* = 0.06) (Figure 2F).

### Regimen Discontinuation for EFV+FTC-TDF

Antiretroviral regimen discontinuation was due to non-prespecified drug substitutions (44% of all observed discontinuations), premature discontinuation of study follow-up (44%), temporary discontinuation of all antiretroviral therapy for more than 8 wk (8%), and permanent discontinuation of all antiretroviral therapy (4%). Risk of this endpoint, when protocol-specified drug substitutions were not counted, did not differ significantly between EFV+FTC-TDF and EFV+3TC-ZDV (Table 2). The most common reasons for non-prespecified drug substitutions among persons randomized to EFV+FTC-TDF and EFV+3TC-ZDV were virologic failure (44 versus 40 cases), clinician/participant decision (seven cases each), and pregnancy (seven cases each).

### Safety of EFV+FTC-TDF

There were fewer safety endpoints among participants assigned to EFV+FTC-TDF compared to EFV+3TC-ZDV (Table 2). Estimated probability of a safety endpoint by week 192 was 45.5% (CI 41.3%–50.0%) versus 61.5% (CI 57.1%–65.9%). Relative risk of a safety endpoint between arms did not vary over time (*p* = 0.8) (Figure 2H). There was a significant interaction between sex and study treatment for the primary safety endpoint (Figure 3B, right side). Women randomized to EFV+FTC-TDF had lower risk of a safety endpoint compared to women randomized to EFV+3TC-ZDV (HR 0.50, CI 0.39–0.64). Among men, risk difference for the primary safety endpoint between arms was attenuated (HR 0.79, CI 0.62–1.00). There were no significant interactions between race, country, or entry plasma HIV-1 RNA stratum and assigned treatment arm for the primary safety endpoint (Figure 3B, right side).

Among the individual safety endpoint components, there were significantly fewer initial dose modifications among participants randomized to EFV+FTC-TDF compared to EFV+3TC-ZDV (Table 2). The estimated cumulative probability of any dose modification of the assigned treatment arms at 192 wk was 25.9% (CI 22.3%–30.0%) compared to 43.9% (CI 39.6%–48.5%), respectively. At any time prior to meeting the primary efficacy endpoint, six (1%) participants assigned to EFV+FTC-TDF switched to ZDV (not prespecified) and 46 (8.9%) participants assigned to EFV+3TC-ZDV switched to TDF (prespecified). Adjustment for effects of crossover from FTC-TDF to 3TC-ZDV and 3TC-ZDV to FTC-TDF, including risk time and events during crossover, did not significantly affect the risk ratio estimate for the primary efficacy comparison (adjusted HR = 0.94).

There were fewer severe or potentially life-threatening laboratory abnormalities in the EFV+FTC-TDF arm compared to the EFV+3TC-ZDV arm (Tables 2 and S8). The estimated cumulative probability of a severe or potentially life-threatening laboratory abnormality at 192 wk was 19.7% (CI 16.4%–23.7%) compared to 30.9% (CI 27.0%–35.2%). Neutropenia was the most common adverse laboratory abnormality among persons assigned to EFV+3TC-ZDV, but the risk of laboratory abnormalities between arms remained significant when neutropenia events were excluded (HR 0.71, CI 0.52–0.96; *p* = 0.03). There were five severe or potentially life-threatening elevations of serum creatinine among participants assigned to EFV+FTC-TDF and two among participants assigned to EFV+3TC-ZDV. The risk of a severe or life-threatening sign or symptom was not significantly different between arms (Tables 2 and S9). Participants assigned to EFV+FTC-TDF had fewer serious metabolic diagnoses compared to participants assigned to EFV+3TC-ZDV (three versus 19 cases; *p*<0.001) (Tables S10 and S11) with seven diagnoses of lipoatrophy and two diagnoses of lipoaccumulation in the EFV+3TC-ZDV arm compared to none in the EFV+FTC-TDF arm.

### Co-infection with *Mycobacterium tuberculosis*

A total of 172 (10.9%) participants were diagnosed with tuberculosis: 91 participants had active tuberculosis at the time of study entry and continued tuberculosis treatment during study follow-up. 81 participants had a new diagnosis of active tuberculosis after study entry. During study follow-up 28 participants randomized to ATV+DDI+FTC had an initial antiretroviral drug substitution because of need for anti-tuberculosis therapy triggering the antiretroviral regimen switch outcome. No participants randomized to EFV plus 3TC-ZDV or EFV plus FTC-TDF had an antiretroviral drug substitution because of anti-tuberculosis treatment.

### Pregnancy

There were 62 pregnancies among 58 women in the trial population. For the comparison of ATV+DDI+FTC to EFV+3TC-ZDV there were 20 and eight pregnancies, respectively, and the incidence of pregnancy among women of childbearing potential was 4.8 per 100 person-years (95% CI 3.1–7.4) versus 1.9 per 100 person-years (95% CI 1.0–3.9). Of these 28 pregnancies, there were 12 live births, nine spontaneous abortions, five induced abortions, one intrauterine fetal demise, and one ectopic pregnancy. For the comparison of EFV+FTC/TDF to EFV+3TC-ZDV there were 20 and 22 pregnancies, respectively, and the incidence of pregnancy among women of childbearing potential was 2.3 per 100 person-years (95% CI 1.5–3.6) versus 2.6 per 100 person-years (95% CI 1.7–3.9). Of these 42 pregnancies, there were 14 live births, 11 spontaneous abortions, seven induced abortions, two intrauterine fetal demise, and eight women remained pregnant at the time of study closure.

## Discussion

A unique feature of PEARLS is the prospective enrollment of a study population from low-, intermediate-, and high-income countries on four continents with near equal proportions of men and women. The distribution of enrollment by country, uniform entry criteria, and quality assurance measures across study sites allowed direct and highly powered comparisons of antiretroviral regimen efficacy in HIV-1-infected persons with diverse racial, cultural, and demographic characteristics. In this context, ATV+DDI+FTC had inferior efficacy compared to EFV+3TC-ZDV and is not recommend as an initial antiretroviral regimen. The regimen of EFV+FTC-TDF had similar high and durable efficacy with a significant safety advantage compared to EFV+3TC-ZDV.

PEARLS was the first clinical trial to prospectively evaluate ATV+DDI+FTC. This regimen had significantly inferior virological efficacy as demonstrated by highly significant greater rates of protocol defined virologic failure (plasma HIV-1 RNA≥1,000 copies/ml at week 16 or later) and significantly less viral suppression (plasma HIV-1 RNA <400 copies/ml) at 24 wk, which was a prespecified secondary endpoint. PEARLS was not designed to directly compare individual antiretroviral agents within regimens, so the reason for inferiority of this antiretroviral combination is uncertain. Participants were instructed to take DDI on an empty stomach at a separate time from when ATV was taken with food. The possibility that this inconvenient dosing schedule could have affected adherence to the ATV+DDI+FTC regimen is being investigated. There was no significant interaction between treatment effect and baseline viral load stratum for the comparison of ATV+DDI+FTC to EFV+3TC/ZDV. However, there was a significant difference in treatment effect between women and men for the efficacy of ATV+DDI+FTC and the inferior efficacy of this regimen compared to EFV+3TC-ZDV was most pronounced among men. Several previous studies demonstrated higher serum protease inhibitor concentrations among women [18]–[20], so we are currently investigating whether inadequate ATV exposure in men in the ATV+DDI+FTC arm explains the interaction between sex and treatment efficacy in PEARLS. Given that women had significantly better relative efficacy with this regimen than men, whether or not this regimen should be used for initial treatment of HIV-1-infected women remains an unanswered question.

Overall, the ATV+DDI+FTC arm had superior safety compared to EFV+3TC/ZDV. Although absolute number of events was small, participants assigned to ATV+DDI+FTC had greater frequency of serious renal disease. Previous large studies in which ATV, didanosine, and FTC were components of other multidrug antiretroviral regimens did not report this toxicity [7]–[14]. Participants assigned to ATV+DDI+FTC had greater CD4+ lymphocyte increases than participants in the EFV+3TC/ZDV arm. Since there was a trend toward more new AIDS endpoints in the ATV arm compared to the EFV arm (18 versus 10; *p* = 0.14) (see Table 1), there was no evidence that the statistically significant difference in CD4+ cell count increase was associated with a clinical benefit.

PEARLS is the second randomized clinical trial to compare EFV+FTC-TDF and EFV+3TC-ZDV in an initial antiretroviral regimen. The study populations of the previous study (GS-01-934) [6] and PEARLS are different. Compared to GS-01-934, PEARLS had a larger sample size for the comparison of EFV+FTC-TDF to EFV+3TC-ZDV (1,045 versus 515) and a larger proportion of women (46% versus 14%), African race (50% versus 23%), Asian race (23% versus ≤4%), Hispanic ethnicity (20% versus 16%), and greater geographic diversity (North America, Caribbean, South America, Africa and Asia versus North America and Europe). These differences in study populations, and their potential effects on study outcomes, should be considered when comparing the results of GS-01-934 and PEARLS.

A second key finding of PEARLS is that EFV+FTC-TDF and EFV+3TC-ZDV had very similar treatment failure rates and both regimens suppressed plasma HIV-1 RNA below 400 copies/ml for greater than 80% of participants for up to 144 wk. Given the precision of the confidence bounds on the efficacy comparisons, we conclude that these regimens had similar efficacy for initial treatment of HIV-1. GS-01-934 reached different conclusions about the relative efficacy of these two antiretroviral regimens [6]. It is possible that differences in the study populations between PEARLS and GS-01-934 contributed to the different efficacy conclusions, but the finding that sex, race, ethnicity, or geography did not affect relative regimen efficacy in PEARLS (Figure 2A, right panel) does not support this explanation. The conclusion of the GS-01-934 study that EFV+FTC-TDF had superior efficacy to EFV+3TC-ZDV was based on an FDA TLOVR primary endpoint that assigned equal consequence to changes in the randomized drug assignments regardless of the reason for change. When the FDA TLOVR algorithm was evaluated as secondary endpoint in PEARLS we also found significant superiority of EFV+FTC-TDF, but a TLOVR algorithm that did not count protocol-prespecified drug substitutions as endpoints did not detect a difference between arms. The discordant findings of the two TLOVR analyses in PEARLS suggest that the different conclusions of GS-934 and PEARLS about relative regimen efficacy could be due to whether or not drug substitutions for toxicity management were included in the primary efficacy endpoint. Inclusion of drug discontinuation for toxicity management in an efficacy endpoint has the potential to lead to inaccurate conclusions about regimen efficacy. This point is illustrated in the efficacy comparison of ATV+DDI+FTC to EFV+3TC-ZDV in PEARLS. In the FDA TLOVR the inferior efficacy of ATV+DDI+FTC was masked by the higher rate of drug substitutions in the EFV+3TC-ZDV arm, whereas in PEARLS the primary endpoint comparison and the TLOVR that did not count protocol-specified drug substitutions ATV+DDI+FTC had clearly inferior efficacy.

Another important finding of PEARLS is that EFV+FTC-TDF had superior safety with significantly less laboratory adverse events compared to EFV+3TC-ZDV. This finding also differs from GS-01-934, which had overall higher rates of primary safety events than PEARLS [6], but did not detect a significant difference in clinical or laboratory adverse events between arms. Since the better safety of EFV+FTC-TDF in PEARLS was most pronounced in women, we speculate that the larger number of women in PEARLS allowed us to detect this safety difference.

The between-arm differences in the PEARLS primary safety analyses were driven largely by higher rates of neutropenia and anemia resulting in protocol-recommended drug substitutions in the EFV+3TC-ZDV arm. Neutropenia and anemia are well-described toxicities of ZDV [21], and neutropenia has been associated with increased risk of serious bacterial infections in HIV-infected people [22]–[25]. Thus the risk, potential consequences and the laboratory monitoring required to detect and manage neutropenia and anemia are important considerations when deciding whether to initiate antiretroviral treatment with EFV+3TC-ZDV. Although no apparent differences in the occurrence of clinical events as a complication of neutropenia or anemia were observed in PEARLS, potential consequences could have been attenuated by frequent laboratory monitoring and standard procedures for clinical management of adverse events specified in the protocol. The finding that the EFV+FTC-TDF arm had significantly fewer serious metabolic diagnoses (which included lipoatrophy and lipodystrophy) is an important safety advantage of this regimen that was also observed in GS-01-934 [26]. Although renal impairment has been associated with TDF, renal adverse events were uncommon in the EFV+FTC-TDF arm of PEARLS.

All HIV-1 protease inhibitors, even with ritonavir boosting, have a significant pharmacokinetic interaction with rifampin that decreases protease inhibitor concentrations and potentially reduces anti-HIV-1 efficacy [3]. Thus use of HIV-1 protease inhibitors in persons with active tuberculosis is not recommended if other options are available. Although active tuberculosis was relatively uncommon in PEARLS participants overall, treatment of tuberculosis was the third most common cause of the antiretroviral regimen discontinuation endpoint in the ATV plus DDI and FTC arm largely because of the requirement to substitute for ATV if there was concomitant use of rifampin. There was no prescribed substitution for EFV if rifampin was used and discontinuation due to tuberculosis treatment did not occur in either EFV containing arm.

Half of HIV-1 infections worldwide occur in women [27], but historically women are underrepresented in clinical trials of antiretroviral therapy [28]. Almost half of PEARLS participants were women so we were able to evaluate potential interactions between sex and treatment effect and safety. Our findings of greater risk of safety events for women assigned to EFV+3TC-ZDV and higher relative efficacy of an ATV-based regimen in women compared to men add to a growing body of evidence that antiretroviral efficacy and safety can differ in women and men [29]–[31], and support further development of sex-specific recommendations for both antiretroviral regimen choice and toxicity monitoring. When treating HIV-1-infected women with EFV it is important to recognize the teratogenic risk during the first trimester of pregnancy. In PEARLS, women of reproductive potential were treated with EFV only if they agreed to use effective birth control methods and close monitoring was performed to detect pregnancies early to ensure that EFV was used safely. Despite these requirements, there were 62 pregnancies during study follow-up, including 42 in the two EFV-containing arms. It is also notable that the incidence of pregnancy of women of child-bearing potential was higher in the ATV+DDI+FTC arm, which had less stringent contraception requirements, compared to the EFV-containing arms. The outcomes of the live births to women in the PEARLS study are reported elsewhere [32].

There are limitations to consider in the application of the study findings to resource-limited settings. PEARLS was conducted at clinical research sites affiliated with academic medical centers in large cities and these environments are undoubtedly different from community clinics or rural health care facilities. The entry criteria resulted in recruitment of a relatively health study population with a low prevalence of co-morbidities. Although most participants had a pretreatment CD4+ lymphocyte count that put them at risk for AIDS-related complications, relatively few reported prior or current AIDS-related infection or malignancy at baseline. Data concerning potential participants who were screened and found not to meet entry criteria were not collected so it is unclear how representative our study population was of other HIV-1-infected persons in care at the study sites. Intense clinical and laboratory monitoring required by the study design could also have affected safety and efficacy outcomes through improved adherence and retention in care. To date, we have only investigated the influence of factors such as race/ethnicity, sex, and geography on treatment effect (e.g., the performance of one regimen relative to another regimen). Although we did not detect interactions between race/ethnicity and geography and treatment effect, this finding does not mean that these factors do not affect antiretroviral efficacy or safety and further analyses to explore these possibilities are ongoing.

To our knowledge, PEARLS is the first study to recruit a study population with this racial, geographic, and sex diversity for a prospective randomized clinic trial of antiretroviral therapy. This unique feature of PEARLS likely contributed to the identification of previously unrecognized sex-related differences in antiretroviral efficacy and safety, and provides an evidence base to better guide the choice of an initial antiretroviral regimen in multinational settings. The efficacy and safety of EFV+FTC-TDF in this diverse study population, especially in HIV-1-infected women, combined with the availability of these three drugs in a single co-formulated tablet with once-daily dosing make this an attractive regimen for initiation of antiretroviral therapy in resource-constrained settings by the criteria outlined in UNAIDS Treatment 2.0 [1].

## Supporting Information

Alternative Language Abstract S1 **Spanish translation of the abstract by Jorge Sanchez.**
(DOC)

Alternative Language Abstract S2 **Portuguese translation of the abstract by Beatriz Grinszteyn.**
(DOC)

Alternative Language Abstract S3 **Thai translation of the abstract by Khuanchai Supparatpinyo.**
(DOCX)

Alternative Language Abstract S4 **Creole translation of the abstract by Cynthia Reverie.**
(DOCX)

Alternative Language Abstract S5 **French translation of the abstract by Cynthia Reverie.**
(DOCX)

Table S1 **Type of infectious diagnoses at study entry.**
(DOC)

Table S2 **Type of opportunistic infections observed for comparison of ATV+DDI-EC+FTC to EFV+3TC-ZDV.**
(DOC)

Table S3 **All new laboratory events of grade 3 or higher for comparison of ATV+DDI-EC+FTC to EFV+3TC-ZDV.**
(DOC)

Table S4 **All new signs and symptoms of grade 3 or higher for comparison of ATV+DDI-EC+FTC to EFV+3TC-ZDV.**
(DOC)

Table S5 **All new serious non-AIDS diagnoses for comparison of ATV+DDI-EC+FTC to EFV+3TC-ZDV.**
(DOC)

Table S6 **New serious non-AIDS diagnosis categories compared for ATV+DDI-EC+FTC versus EFV+3TC-ZDV.**
(DOC)

Table S7 **Type of opportunistic infections observed for comparison of EFV+FTC-TDF to EFV+3TC-ZDV.**
(DOC)

Table S8 **All new laboratory events of grade 3 or higher for comparison of EFV+FTC-TDF to EFV+3TC-ZDV.**
(DOC)

Table S9 **All new signs and symptoms of grade 3 or higher for comparison of EFV+FTC-TDF to EFV+3TC-ZDV.**
(DOC)

Table S10 **All new serious non-AIDS diagnoses for comparison of EFV+FTC-TDF to EFV+3TC-ZDV.**
(DOC)

Table S11 **New serious non-AIDS diagnosis categories compared for EFV+FTC-TDF versus EFV+3TC-ZDV.**
(DOC)

Text S1 **Study protocol.**
(DOC)

Text S2 **CONSORT checklist.**
(DOC)

Text S3 **Appendix 60.** Diagnoses appendix.
(PDF)

## Abbreviations

3TC: lamivudine
ACTG: AIDS Clinical Trials Group
ATV: atazanavir
DDI: didanosine-EC
DF: disoproxil fumarate
DSMB: Data Safety Monitoring Board
EFV: efavirenz
FDA: US Food and Drug Administration
FTC: emtricitabine
HR: hazard ratio
IRIS: immune reconstitution inflammatory syndrome
NNRTI: non-nucleoside reverse transcriptase inhibitor
PEARLS: Prospective Evaluation of Antiretrovirals in Resource Limited Settings
TDF: tenofovir-DF
TLOVR: time to loss of virologic response
ZDV: zidovudine
