# Supplementary material for: Efficacy and Safety of Three Antiretroviral Regimens for Initial Treatment of HIV-1: A Randomized Clinical Trial in Diverse Multinational Settings
Source: PLoS Med. 2012 Aug 14;9(8):e1001290. doi: 10.1371/journal.pmed.1001290 (PMC3419182; doi:10.1371/journal.pmed.1001290)
Supplement: Alternative Language Abstract S1 — Spanish translation of the abstract by Jorge Sanchez. (DOC) [file pmed.1001290.s001.doc]

**Eficacia y Seguridad de tres Esquemas Terapéuticos Antiretrovirales para el Tratamiento Inicial del VIH-1: Un Ensayo Clínico Aleatorizado Multinacional**

**ABSTRACTO**

***Antecedentes*:** Para maximizar la eficiencia de los programas de tratamiento antirretroviral en países con recursos limitados se requiere de regímenes de antirretrovirales con dosificación simplificada y seguridad mejorada. En este estudio investigamos la eficacia y seguridad de regímenes antirretrovirales con dosificación una vez al día versus dos veces al día en diversas áreas del mundo.

***Métodos*:** 1571 personas infectadas con el virus de inmunodeficiencia humana tipo 1 (HIV-1) (47% fueron mujeres) de nueve países en cuatro continentes, fueron asignados con igual probabilidad a terapia de etiqueta abierta con efavirenz mas lamivudina-zidovudina (EFV+3TC-ZDV), atazanavir mas didanosina-EC mas emtricitabina (ATV+DDI+FTC), o efavirenz mas emtricitabina-tenofovir-DF (EFV+FTC-TDF). Se postuló como hipótesis que los esquemas ATV+DDI+FTC y EFV+FTC-TDF serían no-inferiores a EFV+3TC-ZDV si el límite superior de un solo lado del intervalo de confianza al 95% para el hazard ratio (HR) fuera ≤1.35 cuando 30% de los participantes tuvieran falla terapéutica.

***Resultados*:** Un comité revisor independiente recomendó detener el seguimiento antes de la acumulación de 472 fallas terapéuticas. Comparando EFV+FTC-TDF con EFV+3TC-ZDV, con una mediana de 184 semanas de seguimiento, hubieron 95 fallas terapéuticas (18%) en 526 participantes versus 98 fallas en 519 participantes (19%; HR 0.95, intervalo de confianza al 95% [CI] 0.72-1.27; p=0.74). Eventos de seguridad ocurrieron en 243 (46%) participantes asignados a EFV+FTC-TDF versus 313 (60%) asignados a EFV+3TC-ZDV (HR 0.64, CI 0.54-0.76; p<0.001) y se observó una significativa interacción entre sexo y seguridad del régimen (HR 0.50, CI 0.39-0.64 para mujeres; HR 0.79, CI 0.62-1.00 para hombres; p=0.01). Comparando ATV+DDI+FTC con EFV+3TC-ZDV, durante una mediana de seguimiento de 81 semanas hubieron 108 fallas (21%) en 526 participantes asignados a ATV+DDI+FTC y 76 (15%) en 519 participantes asignados a EFV+3TC-ZDV (HR 1.51, CI 1.12-2.04; p=0.007).

***Conclusión*:** EFV+FTC-TDF tuvo similar eficacia en comparación con EFV+3TC-ZDV en esta población de estudio, reclutada en centros multinacionales diversos. La seguridad superior, especialmente en mujeres infectadas con HIV-1, y la dosificación una vez al día de EFV+FTC-TDF son ventajas de este régimen para el tratamiento inicial de la infección por HIV-1 en países con recursos limitados. ATV+DDI+FTC tuvo eficacia inferior y no es recomendable como esquema de tratamiento inicial.
