# Supplementary material for: Efficacy and Safety of Three Antiretroviral Regimens for Initial Treatment of HIV-1: A Randomized Clinical Trial in Diverse Multinational Settings
Source: PLoS Med. 2012 Aug 14;9(8):e1001290. doi: 10.1371/journal.pmed.1001290 (PMC3419182; doi:10.1371/journal.pmed.1001290)
Supplement: Alternative Language Abstract S2 — Portuguese translation of the abstract by Beatriz Grinszteyn. (DOC) [file pmed.1001290.s002.doc]

**Segurança e Eficácia de 3 esquemas antirretrovirais para tratamento inicial do HIV-1: um ensaio clinico randomizado multinacional**

**ABSTRACT**

***Contexto*:** Esquemas antirretrovirais de posologia simplificada e maior segurança são necessários a fim de maximizar a eficiencia da disponibilização de antirretrovirais em locais com recursos limitados. Nós investigamos a eficácia e a segurança de esquemas antirretrovirais de administração uma vez ao dia, comparados à administração duas vezes ao dia em várias regiões do mundo.

***Métodos*:** 1.571 pessoas infectadas pelo vírus da imunodeficiência tipo 1 (*human immunodeficiency virus type* 1 - HIV-1) (47% de mulheres) de nove países em quatro continentes foram designadas com igual probabilidade para terapia antirretroviral aberta com efavirenz e zidovudina/lamivudina (EFV + AZT/3TC), atazanavir e didanosina-EC e entricitabina (ATV + ddI + FTC ), ou efavirenz e entricitabina/tenofovir-DF (EFV+FTC/TDF). A hipótese é que ATV + ddI + FTC e EFV + FTC/TDF sejam não inferiores a EFV + AZT/3TC, se o limite superior de confiança de 95% determinado para o coeficiente de risco (*hazard ratio* - HR) for ≤ 1,35 quando 30% dos participantes tiverem falha terapêutica.

***Achados*:** O comite independente de monitoramento (DSMB) recomendou interromper o estudo antes do acúmulo de 472 falhas terapêuticas. Comparando EFV + FTC/TDF a EFV + AZT/3TC, durante um número mediano de 184 semanas de acompanhamento, houve 95 falhas terapêuticas (18%) entre 526 participantes contra 98 falhas entre 519 participantes (19%; HR 0,95, intervalo da confiança [IC] de 95% 0,72-1,27; *P* = 0,74). Ocorreram desfechos de segurança em 243 participantes (46%) no braço de EFV + FTC/TDF *vs*. 313 (60%) no braço de EFV+AZT/3TC (HR 0,64, IC 0,54-0,76; *P* < 0,001) e houve interação significativa entre o sexo e a segurança do esquema (HR 0,50, IC 0,39-0,64 para mulheres; HR 0,79, IC 0,62-1,00 para homens; *P* = 0,01). Comparando ATV + ddI + FTC a EFV + AZT/3TC durante uma mediana de 81 semanas de acompanhamento, houve 108 falhas terapêuticas (21%) entre 526 participantes no braço de ATV + ddI + FTC e 76 (15%) entre 519 participantes no braço de EFV + AZT/3TC (HR 1,51, IC 1,12-2,04; *P* = 0,007).

***Conclusão*:** A combinação de EFV + FTC/TDF demonstrou alta eficácia em comparação a EFV + AZT/3TC na população do estudo, recrutada em contextos multinacionais diversos. A maior segurança, especialmente entre mulheres infectadas pelo HIV-1, e a administração uma vez ao dia de EFV + FTC/TDF é vantajosa para o uso deste esquema no tratamento inicial da infecção pelo HIV-1 em países com poucos recursos. ATV + ddI + FTC teve eficácia inferior e não é recomendado como esquema antirretroviral inicial.
