# Supplementary material for: Efficacy and Safety of Three Antiretroviral Regimens for Initial Treatment of HIV-1: A Randomized Clinical Trial in Diverse Multinational Settings
Source: PLoS Med. 2012 Aug 14;9(8):e1001290. doi: 10.1371/journal.pmed.1001290 (PMC3419182; doi:10.1371/journal.pmed.1001290)
Supplement: Alternative Language Abstract S3 — Thai translation of the abstract by Khuanchai Supparatpinyo. (DOCX) [file pmed.1001290.s003.docx]

**ประสิทธิผลและความปลอดภัยของการใช้ยาต้านเชื้อเอชไอวี 3 สูตรในการเริ่มรักษาผู้ติดเชื้อเอชไอวี-1: การศึกษาทางคลินิกแบบสุ่มในหลายประเทศ**

บทคัดย่อ

*บทนำ***:** สูตรยาต้านเชื้อเอชไอวีที่ง่ายและมีผลข้างเคียงน้อยมีความจำเป็นในการเพิ่มประสิทธิภาพในการรักษาผู้ติดเชื้อเอชไอวีในประเทศที่มีทรัพยากรจำกัด คณะผู้วิจัยได้ทำการศึกษาเพื่อประเมินประสิทธิผลและความปลอดภัยของการใช้ยาต้านเชื้อเอชไอวีสูตรรับประทานวันละครั้งเปรียบเทียบกับสูตรมาตรฐานที่ต้องรับประทานวันละสองครั้งในหลายประเทศทั่วโลก

**วิธีการศึกษา:** ผู้ติดเชื้อเอชไอวี-1 จำนวน 1571 ราย (47% เป็นหญิง) จาก 9 ประเทศใน 4 ทวีปถูกสุ่มแบบเปิดฉลากเป็น 3 กลุ่มเท่า ๆ กัน กลุ่มที่หนึ่งได้รับยา efavirenz ร่วมกับ lamivudine-zidovudine (EFV+3TC-ZDV) กลุ่มที่สองได้รับยา atazanavir ร่วมกับ didanosine-EC และ emtricitabine (ATV+DDI+FTC) และกลุ่มที่สามได้รับยา efavirenz ร่วมกับ emtricitabine-tenofovir-DF (EFV+FTC-TDF) สมมุติฐานของการศึกษาคือยาในกลุ่ม ATV+DDI+FTC และ EFV+FTC-TDF มีประสิทธิผลไม่ด้อยกว่ายาในกลุ่ม EFV+3TC-ZDV โดยที่ hazard ratio มีขอบเขตบนของค่าความเชื่อมั่นชนิดด้านเดียว 95% น้อยกว่าหรือเท่ากับ 1.35 เมื่อติดตามการรักษาไปจนกระทั่งผู้ป่วยร้อยละ 30 ประสบความล้มเหลวในการรักษา

ผลการศึกษา**:** คณะกรรมการติดตามการวิจัยอิสระแนะนำให้หยุดการศึกษาก่อนที่จะติดตามการรักษาครบตามกำหนดซึ่งจะต้องมีผู้ป่วยที่รักษาล้มเหลวจำนวน 472 ราย เมื่อเปรียบเทียบระหว่างกลุ่มที่หนึ่งและกลุ่มที่สองพบว่าเมื่อติดตามผู้ป่วยเป็นเวลามัธยฐาน 184 สัปดาห์ กลุ่มที่ได้รับยา EFV+FTC-TDF จำนวน 526 ราย มีการรักษาล้มเหลว 95 ราย (18%) เมื่อเทียบกับ 98 ราย (19%) ในกลุ่มที่ได้รับยา EFV+3TC-ZDV จำนวน 519 ราย (HR 0.95, 95% confidence interval [CI] 0.72-1.27; p=0.74) มีผู้ป่วยที่เกิดผลข้างเคียงจำนวน 243 ราย (46%) ในกลุ่มที่ได้รับยา EFV+FTC-TDF เทียบกับ 313 ราย (60%) ในกลุ่มที่ได้รับยา EFV+3TC-ZDV (HR 0.64, CI 0.54-0.76; p<0.001) และมีความสัมพันธ์อย่างมีนัยสำคัญระหว่างเพศและความปลอดภัยของยา (HR 0.50, CI 0.39-0.64 ในเพศหญิง; HR 0.79, CI 0.62-1.00 ในเพศชาย; p=0.01) เมื่อเปรียบเทียบระหว่างกลุ่มที่สองและกลุ่มที่สามพบว่าเมื่อติดตามผู้ป่วยเป็นเวลามัธยฐาน 81 สัปดาห์ กลุ่มที่ได้รับยา ATV+DDI+FTC จำนวน 526 รายมีการรักษาล้มเหลว 108 ราย (21%) เมื่อเทียบกับ 76 ราย (15%) ในกลุ่มที่ได้รับยา EFV+3TC-ZDV จำนวน 519 ราย (HR 1.51, CI 1.12-2.04; p=0.007).

*สรุป***:** ยาต้านเชื้อเอชไอวีในกลุ่ม EFV+FTC-TDF มีประสิทธิผลสูงใกล้เคียงกับยาสูตรมาตรฐานได้แก่ EFV+3TC-ZDV ในการศึกษาในประชากรจากหลายประเทศทั่วโลก การพบว่ายากลุ่มดังกล่าวมีความปลอดภัยสูงกว่าในผู้ป่วยหญิงและเป็นสูตรยาที่รับประทานวันละครั้งทำให้ยาสูตร EFV+FTC-TDF เป็นสูตรยาที่ควรเลือกใช้ในการเริ่มการรักษาผู้ติดเชื้อเอชไอวีในประเทศที่มีทรัพยากรจำกัด ส่วนยาในสูตร ATV+DDI+FTC มีประสิทธิผลด้อยกว่าและไม่ควรนำมาใช้ในการเริ่มรักษาผู้ติดเชื้อเอชไอวี
