# Supplementary material for: Efficacy and Safety of Three Antiretroviral Regimens for Initial Treatment of HIV-1: A Randomized Clinical Trial in Diverse Multinational Settings
Source: PLoS Med. 2012 Aug 14;9(8):e1001290. doi: 10.1371/journal.pmed.1001290 (PMC3419182; doi:10.1371/journal.pmed.1001290)
Supplement: Alternative Language Abstract S4 — Creole translation of the abstract by Cynthia Reverie. (DOCX) [file pmed.1001290.s004.docx]

**« Efikasite ak sekirite twa rejim medikaman antiretwoviwo yo pou premye tretman VIH-1: yon etid klinik soi tiraj oso nan diferan peyi sou latè”**

**REZIME:**

**ENTWODISKSYON**: Rejim medikaman antiretwoviwo yo ki fèt ak dòz senp epi ki prezante pi bon sekirite, yo se medikaman ki nesesè pou ogmante efikasite nan bay medikaman antiretwoviwo nan peyi sou devlope yo. Nou te fè rechèch sou konparezon ki te fèt sou efikasite ak sekirite ant rejim medikaman antiretwoviwo ke moun pran 2 fwa pa jou ak sila yo ke moun pran yon fwa pa jou nan diferan peyi sou latè.

**METOD KI TE ITILIZE** : te gen 1571 moun ki enfekte ak VIH -1 (ladan yo te gen 47% fanm) ki te fè pati 9 peyi ki tabli sou 4 nan kontinan yo. Moun sa yo te gen menm kantite chans pou yo te antre pa chans nan tretman antiretwoviwo ki fèt ak Efavirens plis Lamividin-Zidovidin (EFV + 3TC- ZDV), Atanazavir plis Didanosine ki vlope plis Emtricitabine (ATV +DDI + FTC), oubyen Efavirens plis Emtricitabine –Tenofovir (EFV+FTC +TDF). Yo panse ke ATV +DDI +FTC ak EFV + 3TC – ZDV pa te enferyè pa rapò ak EFV + FTC + TDF si piwo nivo konfyans lan rive nan 95% pou rasyo aza a (HR) ki te ≤ 1.35 lè ta gen echèk tretman an pou 30% nan patisipan yo.

**REZILTA** : Gen yon komite sipèvizyon ki pa fè pati de ekip ki te kondwi etid la ki rekòmande pou kanpe etid swivi ki t’ap fèt anvan an akòz te gen 472 ka echèk nan tretman an. Lè yo te konpare rejim EFV+FTC+-TDF ak rejim EFV+3TC-ZDV pandan yon tan swivi mwayen ki te dire 184 semenn, te gen 95 ka echèk nan tretman an (swa 18%) pami 526 patisipan kont 98 ka echèk pami 519 patisipan (19%; HR 0.95, 95% entèval konfyans [CI] 0.72- 1.27; p=0.74). Evenman sekirite ke etid la tap chèche te parèt kay 243 (46%) nan patisipan yo ki te plase sou rejim EFV+FTC-TDF kont 313 (60%) patisipan ki te plase sou EFV+3TC-ZDV (HR 0.64, CI 0.54-0.76; p<0.001) epi te gen yon entèraksyon enpòtan ant sèks ak sekirite rejim antiretwoviwo (HR 0.50, CI 0.39-0.64 pou fanm yo; HR 0.79. CI 0.62-1.00 pou gason yo : p=0.01). Lè yo te konpare rejim ATV+DDI+FTC ak rejim EFV+3TC-ZDV pandan yon tan swivi mwayen ki te dire 81 semenn, te gen 108 (21%) ka echèk pami 526 patisipan ki te plase sou ATV+DDI+FTC epi 76 (15%) ka echèk pami 519 patisipan ki te plase sou EFV+3TC-ZDV (HR 1.51, CI 1.12-2.04; P=0.007).

**KONKLIZYON** : Yo te jwenn rejim EFV+FTC-TDF gen menm gwo efikasite ak rejim EFV+3TC-ZDV nan etid sa ki te fèt sou popilasyon ke yo te rekrite nan diferan peyi ki nan rezo rechèch la. Sekirite siperyè, espesyalman kay fanm ki enfekte ak VIH-1, epi itilzasyon dòz inik pa jou rejim EFV+FTC-TDF, prezante avantaj nan itilizasyon rejim sa-a pou premye tretman kont enfeksyon VIH-1 nan peyi sou devlope. Rejim ATV+DDI+FTC prezante yon efikasite ki enferyè epi li pa rekòmande pou yo itilize l’ kòm premye rejim antiretwoviwo.
