# Supplementary material for: Efficacy and Safety of Three Antiretroviral Regimens for Initial Treatment of HIV-1: A Randomized Clinical Trial in Diverse Multinational Settings
Source: PLoS Med. 2012 Aug 14;9(8):e1001290. doi: 10.1371/journal.pmed.1001290 (PMC3419182; doi:10.1371/journal.pmed.1001290)
Supplement: Alternative Language Abstract S5 — French translation of the abstract by Cynthia Reverie. (DOCX) [file pmed.1001290.s005.docx]

**« L'efficacité et l'innocuité de trois schémas thérapeutiques antirétroviraux pour le traitement initial du VIH-1: un essai clinique randomisé dans divers contextes multinationales »**

**RÉSUMÉ**

**Mise en contexte**: Des régimes antirétroviraux avec dosage simplifié et une meilleure sécurité sont nécessaires pour maximiser l'efficacité de la prestation des antirétroviraux dans les pays à ressources limitées. Nous avons étudié l'efficacité et l'innocuité des traitements antirétroviraux à dosage uni quotidien  par rapport à ceux à dosage biquotidien dans divers régions à travers le monde.

**Méthodes**: 1571 personnes infectées par le virus de l'immunodéficience humaine de type 1 (VIH-1) dont 47% de sexe féminin provenant de neuf pays de quatre continents ont été assignés, avec une probabilité égale à une thérapie antirétrovirale connue contenant soit l'Efavirenz et la Lamivudine-Zidovudine (EFV +3 TC-ZDV ), soit l'Atazanavir plus Didanosine-EC (à enrobage entérique) ainsi que l'Emtricitabine (ATV + DDI + FTC), soit de l'Efavirenz, plus l'Emtricitabine-Ténofovir (EFV + FTC-TDF). L’hypothèse de l’étude était que ATV + DDI + EFV + FTC et FTC-TDF étaient non-inférieurs à l'EFV + 3TC-ZDV si le plus haut niveau de confiance serait de 95% pour un ratio hasard (HR) de ≤ 1,35, supposant 30% des participants auraient fait échec au traitement.

**Résultats**: Un conseil de surveillance indépendant a recommandé l'arrêt de l'étude avant l'accumulation des 472 échecs de traitement. En comparant l'EFV + FTC-TDF à l'EFV+ 3TC-ZDV, pendant une médiane de 184 semaines de suivi, il y avait 95 échecs au traitement (18%) parmi les 526 participants contre 98 échecs parmi les 519 participants (19%; HR 0,95, 95% d’intervalle de confiance [CI] 0.72 à 1.27, p = 0,74). Les événements de sécurité recherchés ont eu lieu dans 243 (46%) des participants affectés à l'EFV + FTC-TDF contre 313 (60%) affecté à l'EFV +3 TC-ZDV (HR 0,64, IC 0,54 à 0.76, p <0,001) et il y avait une interaction significative entre le sexe et la sécurité du régime (HR 0,50, IC de 0.39 à 0.64 pour les femmes; RR 0,79, IC 0.62 à 1.00 pour les hommes, p = 0,01). En comparant ATV + DDI + FTC à EFV+3 TC-ZDV, pendant une durée médiane de suivi de 81 semaines, il y a eu 108 échecs (21%) parmi les 526 participants affectés à ATV + DDI + FTC et 76 (15%) parmi les 519 participants affectés à EFV +3TC-ZDV (HR 1,51, IC 1.12 à 2.4, p = 0,007).

**Conclusion**: EFV + FTC-TDF a la même grande efficacité par rapport à EFV + 3TC-ZDV dans cette population de l'essai, recrutée dans des régions multinationales. Le régime à dosage uni quotidien : Une sécurité supérieure, en particulier chez les femmes infectées par le VIH-1, et un dosage uni quotidien de EFV + FTC-TDF constitue un avantage pour l'utilisation de ce régime pour le traitement initial du VIH-1 dans les pays à ressources limitées. ATV + DDI + FTC a une efficacité inférieure et n'est pas recommandée comme un régime antirétroviral thérapeutique initial.
