# Supplementary material for: Efficacy and Safety of Three Antiretroviral Regimens for Initial Treatment of HIV-1: A Randomized Clinical Trial in Diverse Multinational Settings
Source: PLoS Med. 2012 Aug 14;9(8):e1001290. doi: 10.1371/journal.pmed.1001290 (PMC3419182; doi:10.1371/journal.pmed.1001290)
Supplement: Table S1 — Type of infectious diagnoses at study entry. (DOC) [file pmed.1001290.s006.doc]

**Table S1: Type of infectious diagnoses at study entry**

| **Active Infections at Study Entry** | **EFV+** | | **ATV+** | | **EFV+**  **FTC-TDF** | | **Total** | |
| --- | --- | --- | --- | --- | --- | --- | --- | --- |
|  | **3TC-ZDV** | | **DDI+FTC** | |  |  |  |  |
| Any infection | 153 |  | 137 |  | 144 |  | 434 |  |
| Candidiasis, oropharyngeal | 22 |  | 26 |  | 29 |  | 77 |  |
| Tuberculosis, pulmonary | 28 |  | 20 |  | 25 |  | 73 |  |
| Mucocutaneous herpes simplex | 24 |  | 22 |  | 19 |  | 65 |  |
| Anogenital warts | 25 |  | 13 |  | 18 |  | 56 |  |
| Candidiasis, other | 17 |  | 14 |  | 13 |  | 44 |  |
| Candidiasis, vulvovaginal | 11 |  | 11 |  | 10 |  | 32 |  |
| Chronic hepatitis C | 12 |  | 6 |  | 7 |  | 25 |  |
| Oral hairy leukoplakia | 8 |  | 6 |  | 8 |  | 22 |  |
| Tuberculosis, extra pulmonary | 10 |  | 4 |  | 7 |  | 21 |  |
| Chronic hepatitis B | 3 |  | 7 |  | 4 |  | 14 |  |
| Syphilis | 5 |  | 4 |  | 3 |  | 12 |  |
| Bacterial sinusitis | 3 |  | 3 |  | 4 |  | 10 |  |
| Malaria | 3 |  | 4 |  | 3 |  | 10 |  |
| Fungal infection, other | 1 |  | 4 |  | 4 |  | 9 |  |
| Varicella zoster | 2 |  | 2 |  | 4 |  | 8 |  |
| Bacterial pneumonia | 2 |  | 4 |  | 1 |  | 7 |  |
| Candidiasis, esophageal | 3 |  | 1 |  | 2 |  | 6 |  |
| Histoplasmosis, disseminated | 1 |  | 2 |  | 2 |  | 5 |  |
| Acute gastrointestinal/diarrheal syndrome | 2 |  | 0 |  | 2 |  | 4 |  |
| Chronic diarrhea | 1 |  | 2 |  | 1 |  | 4 |  |
| Toxoplasma encephalitis | 0 |  | 1 |  | 3 |  | 4 |  |
| CMV retinitis | 1 |  | 1 |  | 0 |  | 2 |  |
| Mycobacterium avium complex (MAC) | 0 |  | 1 |  | 1 |  | 2 |  |
| Paracoccidioidomycosis | 0 |  | 1 |  | 1 |  | 2 |  |
| Pelvic inflammatory disease | 2 |  | 0 |  | 0 |  | 2 |  |
| *Penicilliosis marneffe*i, disseminated | 0 |  | 1 |  | 1 |  | 2 |  |
| Chronic diarrhea | 2 |  | 0 |  | 0 |  | 2 |  |
| *Pneumocystis carinii* pneumonia | 1 |  | 1 |  | 0 |  | 2 |  |
| Gonorrhea | 0 |  | 2 |  | 0 |  | 2 |  |
| Acute dysentery | 0 |  | 1 |  | 0 |  | 1 |  |
| Bacterial sepsis | 0 |  | 0 |  | 1 |  | 1 |  |
| CMV colitis | 0 |  | 0 |  | 1 |  | 1 |  |
| Cryptococcal meningitis | 0 |  | 0 |  | 1 |  | 1 |  |
| Fungal infection, nails | 0 |  | 0 |  | 1 |  | 1 |  |
| Latent tuberculosis | 0 |  | 0 |  | 1 |  | 1 |  |
| Mucocutaneous CMV ulcers | 1 |  | 0 |  | 0 |  | 1 |  |
| Mumps/parotitis | 1 |  | 0 |  | 0 |  | 1 |  |
| Non-tuberculosis, non-MAC mycobacteria | 1 |  | 0 |  | 0 |  | 1 |  |
| Osteomyelitis | 0 |  | 1 |  | 0 |  | 1 |  |
| Cryptococcosis, pulmonary | 1 |  | 0 |  | 0 |  | 1 |  |
| *Trichomonas vaginalis* | 1 |  | 0 |  | 0 |  | 1 |  |
| Venereal disease, other | 0 |  | 1 |  | 0 |  | 1 |  |
