# Supplementary material for: Efficacy and Safety of Three Antiretroviral Regimens for Initial Treatment of HIV-1: A Randomized Clinical Trial in Diverse Multinational Settings
Source: PLoS Med. 2012 Aug 14;9(8):e1001290. doi: 10.1371/journal.pmed.1001290 (PMC3419182; doi:10.1371/journal.pmed.1001290)
Supplement: Table S2 — Type of opportunistic infections observed for comparison of ATV+DDI-EC+FTC to EFV+3TC-ZDV. (DOC) [file pmed.1001290.s007.doc]

**Table S2:** Type of opportunistic infections observed through 22-May-2008 according to randomized treatment arm: atazanavir plus didanosine-EC and emtricitabine (ATV+DDI-EC+FTC) and efavirenz plus lamivudine-zidovudine (EFV+3TC-ZDV)

|  | | **Randomized Group** | |  |
| --- | --- | --- | --- | --- |
|  |  | **EFV+**  **3TC-ZDV** | **ATV+**  **DDI+FTC** | **Total** |
|  | Extra pulmonary tuberculosis | 9 (39%) | 10 (27%) | 19 (32%) |
|  | Bacterial pneumonia | 3 (13%) | 3 (8%) | 6 (10%) |
|  | *Pneumocystis jiroveci* pneumonia (PCP) | 3 (17%) | 3 (8%) | 6 (10%) |
|  | Mucocutaneous herpes simplex | 3 (13%) | 2 (5%) | 5 (8%) |
|  | Cryptococcal meningitis | 0 (0%) | 3 (8%) | 3 (5%) |
|  | Mycobacterium avium complex (MAC) | 2 (9%) | 1 (3%) | 3 (5%) |
|  | Cytomegalovirus retinitis | 0 (0%) | 2 (5%) | 2 (3%) |
|  | Invasive cervical carcinoma | 0 (0%) | 2 (5%) | 2 (3%) |
|  | Isosporiasis | 0 (0%) | 2 (5%) | 2 (3%) |
|  | Systemic non-Hodgkin lymphoma (NHL) | 1 (4%) | 1 (3%) | 2 (3%) |
|  | Toxoplasmic encephalitis | 1 (4%) | 1 (3%) | 2 (3%) |
|  | Cytomegalovirus colitis | 0 (0%) | 1 (3%) | 1 (2%) |
|  | Cytomegalovirus esophagitis | 0 (0%) | 1 (3%) | 1 (2%) |
|  | Coccidioidal meningitis | 0 (0%) | 1 (3%) | 1 (2%) |
|  | Cryptosporidiosis | 0 (0%) | 1 (3%) | 1 (2%) |
|  | Disseminated cryptococcus | 0 (0%) | 1 (3%) | 1 (2%) |
|  | Disseminated histoplasmosis | 0 (0%) | 1 (3%) | 1 (2%) |
|  | Kaposi sarcoma (KS) mucocutaneous and visceral | 0 (0%) | 1 (3%) | 1 (2%) |
|  | Progressive multifocal encephalopathy | 1 (4%) | 0 (0%) | 1 (2%) |
|  | | | | |
