# Supplementary material for: Efficacy and Safety of Three Antiretroviral Regimens for Initial Treatment of HIV-1: A Randomized Clinical Trial in Diverse Multinational Settings
Source: PLoS Med. 2012 Aug 14;9(8):e1001290. doi: 10.1371/journal.pmed.1001290 (PMC3419182; doi:10.1371/journal.pmed.1001290)
Supplement: Table S3 — All new laboratory events of grade 3 or higher for comparison of ATV+DDI-EC+FTC to EFV+3TC-ZDV. (DOC) [file pmed.1001290.s008.doc]

**Table S3:** All new laboratory events of grade 3 or higher through 22-May-2008 for the comparison of atazanavir plus didanosine-EC and emtricitabine (ATV+DDI-EC+FTC) to efavirenz plus lamivudine-zidovudine (EFV+3TC-ZDV)

|  | **Randomized Group** | | | | | | | | |
| --- | --- | --- | --- | --- | --- | --- | --- | --- | --- |
|  | **EFV+3TC-ZDV (N=519)** | | | **ATV+DDI-EC+FTC (N=526)** | | | **All (N=1045)** | | |
|  | **Grade** | |  | **Grade** | |  | **Grade** | |  |
| **Laboratory Event** | **3** | **4** | **Number subjects** | **3** | **4** | **Number subjects** | **3** | **4** | **Number subjects** |
| Any Chemistry | 24 (5%) | 6 (1%) | 30 (6%) | 31 (6%) | 3 (1%) | 34 (6%) | 55 (5%) | 9 (1%) | 64 (6%) |
| Any Chemistry, General | 24 | 6 | 30 | 31 | 3 | 34 | 55 | 9 | 64 |
| Albumin | 2 | 0 | 2 | 6 | 0 | 6 | 8 | 0 | 8 |
| Alkaline Phosphatase | 2 | 2 | 4 | 3 | 0 | 3 | 5 | 2 | 7 |
| Bicarbonate | 1 | 1 | 2 | 0 | 1 | 1 | 1 | 2 | 3 |
| Calcium | 0 | 1 | 1 | 0 | 0 | 0 | 0 | 1 | 1 |
| Creatine Kinase | 0 | 2 | 2 | 0 | 0 | 0 | 0 | 2 | 2 |
| Phosphorus | 18 | 0 | 18 | 25 | 0 | 25 | 43 | 0 | 43 |
| Potassium | 2 | 0 | 2 | 1 | 3 | 4 | 3 | 3 | 6 |
| Sodium | 0 | 0 | 0 | 3 | 1 | 4 | 3 | 1 | 4 |
| Any Endocrine | 0 (0%) | 0 (0%) | 0 (0%) | 3 (1%) | 1 (0%) | 4 (1%) | 3 (0%) | 1 (0%) | 4 (0%) |
| Any Endocrine, Metabolic | 0 | 0 | 0 | 3 | 1 | 4 | 3 | 1 | 4 |
| Fasting Blood Sugar | 0 | 0 | 0 | 3 | 1 | 4 | 3 | 1 | 4 |
| Any Hematology | 56 (11%) | 32 (6%) | 88 (17%) | 22 (4%) | 11 (2%) | 33 (6%) | 78 (7%) | 43 (4%) | 121 (12%) |
| Any Hematology, Coagulation | 3 | 3 | 6 | 5 | 1 | 6 | 8 | 4 | 12 |
| Partial Thromboplastin Time | 0 | 0 | 0 | 1 | 0 | 1 | 1 | 0 | 1 |
| Platelets | 3 | 3 | 6 | 4 | 1 | 5 | 7 | 4 | 11 |
| Any Hematology, RBC | 10 | 8 | 18 | 2 | 3 | 5 | 12 | 11 | 23 |
| Hemoglobin | 10 | 8 | 18 | 2 | 3 | 5 | 12 | 11 | 23 |
| Any Hematology, WBC/Differential | 48 | 21 | 69 | 16 | 8 | 24 | 64 | 29 | 93 |
| Absolute Neutrophil Count | 46 | 21 | 67 | 16 | 7 | 23 | 62 | 28 | 90 |
| White Blood Cells | 3 | 0 | 3 | 0 | 1 | 1 | 3 | 1 | 4 |
| Any Metabolic | 6 (1%) | 1 (0%) | 7 (1%) | 7 (1%) | 1 (0%) | 8 (2%) | 13 (1%) | 2 (0%) | 15 (1%) |
| Glucose (NON-FASTING) | 0 | 0 | 0 | 4 | 0 | 4 | 4 | 0 | 4 |
| Lactate | 3 | 0 | 3 | 0 | 1 | 1 | 3 | 1 | 4 |
| LDL (FASTING) | 0 | 0 | 0 | 1 | 0 | 1 | 1 | 0 | 1 |
| Total Cholesterol (FASTING) | 2 | 0 | 2 | 1 | 0 | 1 | 3 | 0 | 3 |
| Total Triglycerides (FASTING) | 1 | 1 | 2 | 1 | 0 | 1 | 2 | 1 | 3 |
| Any Liver/Hepatic | 11 (2%) | 10 (2%) | 21 (4%) | 136 (26%) | 38 (7%) | 174 (33%) | 147 (14%) | 48 (5%) | 195 (19%) |
| SGOT/AST | 7 | 10 | 17 | 10 | 5 | 15 | 17 | 15 | 32 |
| SGPT/ALT | 7 | 5 | 12 | 9 | 6 | 15 | 16 | 11 | 27 |
| Total Bilirubin | 5 | 1 | 6 | 132 | 31 | 163 | 137 | 32 | 169 |
| Any Renal | 0 (0%) | 0 (0%) | 0 (0%) | 0 (0%) | 2 (0%) | 2 (0%) | 0 (0%) | 2 (0%) | 2 (0%) |
| Creatinine | 0 | 0 | 0 | 0 | 2 | 2 | 0 | 2 | 2 |
| Any Pancreatic | 0 (0%) | 3 (1%) | 3 (1%) | 2 (0%) | 3 (1%) | 5 (1%) | 2 (0%) | 6 (1%) | 8 (1%) |
| Lipase | 0 | 3 | 3 | 2 | 3 | 5 | 2 | 6 | 8 |
| Pancreatic Serum Amylase | 0 | 1 | 1 | 0 | 0 | 0 | 0 | 1 | 1 |
| Any event | 83 (16%) | 46 (9%) | 129 (25%) | 171 (33%) | 53 (10%) | 224 (43%) | 254 (24%) | 99 (9%) | 353 (34%) |

Multiple episodes or adverse events on same row are counted only once. DAIDS Severity Grading: 3 = Severe, 4 = Life-Threatening. Worst grade for each AE category is presented and only follow-up during initial antiretroviral regimen included.
