# Supplementary material for: Efficacy and Safety of Three Antiretroviral Regimens for Initial Treatment of HIV-1: A Randomized Clinical Trial in Diverse Multinational Settings
Source: PLoS Med. 2012 Aug 14;9(8):e1001290. doi: 10.1371/journal.pmed.1001290 (PMC3419182; doi:10.1371/journal.pmed.1001290)
Supplement: Table S4 — All new signs and symptoms of grade 3 or higher for comparison of ATV+DDI-EC+FTC to EFV+3TC-ZDV. (DOC) [file pmed.1001290.s009.doc]

**Table S4:**

All new signs and symptoms of grade 3 or higher through 22-May-2008 for the comparison of atazanavir plus didanosine-EC and emtricitabine (ATV+DDI-EC+FTC) to efavirenz plus lamivudine-zidovudine (EFV+3TC-ZDV**)**

|  | **Randomized Group** | | | | | | | | |
| --- | --- | --- | --- | --- | --- | --- | --- | --- | --- |
|  | **EFV+3TC-ZDV (N=519)** | | | **ATV+DDI-EC+FTC (N=526)** | | | **All (N=1045)** | | |
|  | **Grade** | |  | **Grade** | |  | **Grade** | |  |
| **Sign/Symptom** | **3** | **4** | **Number subjects** | **3** | **4** | **Number subjects** | **3** | **4** | **Number subjects** |
| Any General Body | 48 (9%) | 4 (1%) | 52 (10%) | 26 (5%) | 6 (1%) | 32 (6%) | 74 (7%) | 10 (1%) | 84 (8%) |
| Abnormal Physical Appearance | 2 | 0 | 2 | 0 | 0 | 0 | 2 | 0 | 2 |
| Ache/Pain/discomfort | 24 | 3 | 27 | 19 | 3 | 22 | 43 | 6 | 49 |
| Asthenia/Fatigue/malaise | 6 | 0 | 6 | 0 | 0 | 0 | 6 | 0 | 6 |
| Cachexia/Wasting/weight Loss | 16 | 1 | 17 | 4 | 1 | 5 | 20 | 2 | 22 |
| Chills/Rigors/sweats/nightsweats | 1 | 0 | 1 | 0 | 0 | 0 | 1 | 0 | 1 |
| Fever | 10 | 0 | 10 | 10 | 2 | 12 | 20 | 2 | 22 |
| Any Respiratory | 8 (2%) | 1 (0%) | 9 (2%) | 7 (1%) | 0 (0%) | 7 (1%) | 15 (1%) | 1 (0%) | 16 (2%) |
| Cough | 3 | 0 | 3 | 5 | 0 | 5 | 8 | 0 | 8 |
| Difficulty Breathing/Dyspnea | 5 | 1 | 6 | 3 | 0 | 3 | 8 | 1 | 9 |
| Respiratory System Dysfunction | 1 | 0 | 1 | 1 | 0 | 1 | 2 | 0 | 2 |
| Any Circulatory/Cardiac | 10 (2%) | 0 (0%) | 10 (2%) | 6 (1%) | 1 (0%) | 7 (1%) | 16 (2%) | 1 (0%) | 17 (2%) |
| Cardiovascular Dysfunction | 1 | 0 | 1 | 2 | 0 | 2 | 3 | 0 | 3 |
| Edema/Enlarged/swollen | 6 | 0 | 6 | 4 | 1 | 5 | 10 | 1 | 11 |
| Heart Rate/Rhythm Abnormal | 3 | 0 | 3 | 0 | 0 | 0 | 3 | 0 | 3 |
| Any Hematology | 2 (0%) | 0 (0%) | 2 (0%) | 1 (0%) | 0 (0%) | 1 (0%) | 3 (0%) | 0 (0%) | 3 (0%) |
| Any Hematology, Signs and Symptoms | 2 | 0 | 2 | 1 | 0 | 1 | 3 | 0 | 3 |
| Bleeding/Bruising/petechiae | 0 | 0 | 0 | 1 | 0 | 1 | 1 | 0 | 1 |
| Lymphadenopathy | 2 | 0 | 2 | 0 | 0 | 0 | 2 | 0 | 2 |
| Any Liver/Hepatic | 0 (0%) | 0 (0%) | 0 (0%) | 4 (1%) | 0 (0%) | 4 (1%) | 4 (0%) | 0 (0%) | 4 (0%) |
| Hepatomegaly | 0 | 0 | 0 | 1 | 0 | 1 | 1 | 0 | 1 |
| Jaundice | 0 | 0 | 0 | 4 | 0 | 4 | 4 | 0 | 4 |
| Any Gastro-Intestinal | 16 (3%) | 0 (0%) | 16 (3%) | 11 (2%) | 2 (0%) | 13 (2%) | 27 (3%) | 2 (0%) | 29 (3%) |
| Appetite Loss/Decreased/anorexia | 4 | 0 | 4 | 2 | 0 | 2 | 6 | 0 | 6 |
| Constipation | 0 | 0 | 0 | 0 | 1 | 1 | 0 | 1 | 1 |
| Diarrhea/Loose Stools | 10 | 0 | 10 | 7 | 0 | 7 | 17 | 0 | 17 |
| Nausea | 3 | 0 | 3 | 2 | 1 | 3 | 5 | 1 | 6 |
| Nausea And Vomiting/Vomiting | 3 | 0 | 3 | 3 | 1 | 4 | 6 | 1 | 7 |
| Any Reproductive | 1 (0%) | 0 (0%) | 1 (0%) | 2 (0%) | 0 (0%) | 2 (0%) | 3 (0%) | 0 (0%) | 3 (0%) |
| Contraction/Cramp | 0 | 0 | 0 | 1 | 0 | 1 | 1 | 0 | 1 |
| Discharge/Drainage | 0 | 0 | 0 | 1 | 0 | 1 | 1 | 0 | 1 |
| Discharge/Exudate/pus | 1 | 0 | 1 | 0 | 0 | 0 | 1 | 0 | 1 |
| Any Skin | 15 (3%) | 0 (0%) | 15 (3%) | 8 (2%) | 0 (0%) | 8 (2%) | 23 (2%) | 0 (0%) | 23 (2%) |
| Allergic Rash/Urticaria/welts/hives | 1 | 0 | 1 | 1 | 0 | 1 | 2 | 0 | 2 |
| Blister/Ulcer/lesions | 4 | 0 | 4 | 0 | 0 | 0 | 4 | 0 | 4 |
| Erythema/Redness/inflammation | 2 | 0 | 2 | 2 | 0 | 2 | 4 | 0 | 4 |
| Induration | 2 | 0 | 2 | 0 | 0 | 0 | 2 | 0 | 2 |
| Itchy/Pruritus | 1 | 0 | 1 | 1 | 0 | 1 | 2 | 0 | 2 |
| Macules/Papules/rash | 10 | 0 | 10 | 5 | 0 | 5 | 15 | 0 | 15 |
| Patch/Plaque | 1 | 0 | 1 | 0 | 0 | 0 | 1 | 0 | 1 |
| Any Neurological | 21 (4%) | 1 (0%) | 22 (4%) | 10 (2%) | 3 (1%) | 13 (2%) | 31 (3%) | 4 (0%) | 35 (3%) |
| Agitation/Hyperactive | 0 | 0 | 0 | 1 | 0 | 1 | 1 | 0 | 1 |
| Burning Sensation | 0 | 0 | 0 | 1 | 1 | 2 | 1 | 1 | 2 |
| Confusion/Difficulty Concentrating | 3 | 0 | 3 | 0 | 0 | 0 | 3 | 0 | 3 |
| Consciousness Level Change/Lethargy | 1 | 0 | 1 | 2 | 0 | 2 | 3 | 0 | 3 |
| Convulsion/Seizure | 2 | 0 | 2 | 0 | 0 | 0 | 2 | 0 | 2 |
| Depression | 1 | 1 | 2 | 0 | 0 | 0 | 1 | 1 | 2 |
| Diplopia/Vision Blurred/Alteration | 2 | 0 | 2 | 1 | 0 | 1 | 3 | 0 | 3 |
| Dreams/Insomnia/sleeping Problems | 2 | 0 | 2 | 0 | 0 | 0 | 2 | 0 | 2 |
| Headache | 7 | 0 | 7 | 3 | 1 | 4 | 10 | 1 | 11 |
| Inappropriate/Changed Behavior | 3 | 0 | 3 | 0 | 0 | 0 | 3 | 0 | 3 |
| Memory Loss | 1 | 0 | 1 | 0 | 0 | 0 | 1 | 0 | 1 |
| Mental Status Changes | 4 | 0 | 4 | 0 | 0 | 0 | 4 | 0 | 4 |
| Neurologic Dysfunction | 1 | 0 | 1 | 1 | 0 | 1 | 2 | 0 | 2 |
| Numbness/Paresthesia/tingling | 2 | 0 | 2 | 1 | 0 | 1 | 3 | 0 | 3 |
| Rigid/Tight/stiff | 1 | 0 | 1 | 3 | 0 | 3 | 4 | 0 | 4 |
| Weakness | 1 | 0 | 1 | 0 | 1 | 1 | 1 | 1 | 2 |
| Any Other | 14 (3%) | 1 (0%) | 15 (3%) | 2 (0%) | 1 (0%) | 3 (1%) | 16 (2%) | 2 (0%) | 18 (2%) |
| Dizzy/Lightheaded/fainting | 14 | 0 | 14 | 0 | 0 | 0 | 14 | 0 | 14 |
| Incontinence/Loss Of Control | 0 | 0 | 0 | 0 | 1 | 1 | 0 | 1 | 1 |
| Lump/Mass/nodule | 0 | 0 | 0 | 1 | 0 | 1 | 1 | 0 | 1 |
| Other | 0 | 1 | 1 | 1 | 0 | 1 | 1 | 1 | 2 |
| Any sign/symptom | 89 (17%) | 7 (1%) | 96 (18%) | 51 (10%) | 9 (2%) | 60 (11%) | 140 (13%) | 16 (2%) | 156 (15%) |

Multiple episodes or adverse events on same row are counted only once. DAIDS Severity Grading: 3 = Severe, 4 = Life-Threatening. Worst grade for each AE category is presented and only follow-up during initial antiretroviral regimen included.
