# Supplementary material for: Efficacy and Safety of Three Antiretroviral Regimens for Initial Treatment of HIV-1: A Randomized Clinical Trial in Diverse Multinational Settings
Source: PLoS Med. 2012 Aug 14;9(8):e1001290. doi: 10.1371/journal.pmed.1001290 (PMC3419182; doi:10.1371/journal.pmed.1001290)
Supplement: Table S5 — All new serious non-AIDS diagnoses for comparison of ATV+DDI-EC+FTC to EFV+3TC-ZDV. (DOC) [file pmed.1001290.s010.doc]

**Table S5:** All New Serious Non-AIDS Diagnoses (SNADES) through 22-May-2008 for the comparison of atazanavir plus didanosine-EC and emtricitabine (ATV+DDI-EC+FTC) to efavirenz plus lamivudine-zidovudine (EFV+3TC-ZDV)

|  | **Randomized study group** | | | | | | | | | | | |  | | | | | |
| --- | --- | --- | --- | --- | --- | --- | --- | --- | --- | --- | --- | --- | --- | --- | --- | --- | --- | --- |
|  | **EFV+3TC-ZDV (N=106)** | | | | | | **ATV+DDI-EC+FTC (N=117)** | | | | | | **All participants (N=223)** | | | | | |
|  | **Gender** | | | |  | | **Gender** | | | |  | | **Gender** | | | |  | |
| **SNADES Dx** | **M** | | **F** | | **Subtotal** | | **M** | | **F** | | **Subtotal** | | **M** | | **F** | | **Subtotal** | |
| **Overall** | **50** | **(47%)** | **56** | **(53%)** | **106** | **(100%)** | **55** | **(47%)** | **62** | **(53%)** | **117** | **(100%)** | **105** | **(47%)** | **118** | **(53%)** | **223** | **(100%)** |
| **Serious Bacterial Infection** | **22** | **(21%)** | **26** | **(25%)** | **48** | **(45%)** | **27** | **(23%)** | **36** | **(31%)** | **63** | **(54%)** | **49** | **(22%)** | **62** | **(28%)** | **111** | **(50%)** |
| Bacterial pneumonia | 9 | (8%) | 7 | (7%) | 16 | (15%) | 8 | (7%) | 15 | (13%) | 23 | (20%) | 17 | (8%) | 22 | (10%) | 39 | (17%) |
| Pulmonary tuberculosis | 6 | (6%) | 6 | (6%) | 12 | (11%) | 13 | (11%) | 10 | (9%) | 23 | (20%) | 19 | (9%) | 16 | (7%) | 35 | (16%) |
| Acute gastrointestinal/diarrheal syndrome | 8 | (8%) | 7 | (7%) | 15 | (14%) | 6 | (5%) | 8 | (7%) | 14 | (12%) | 14 | (6%) | 15 | (7%) | 29 | (13%) |
| Pelvic inflammatory disease | 0 | (0%) | 3 | (3%) | 3 | (3%) | 0 | (0%) | 9 | (8%) | 9 | (8%) | 0 | (0%) | 12 | (5%) | 12 | (5%) |
| Gastrointestinal - gastrointestinal system disease/disorder, other | 0 | (0%) | 3 | (3%) | 3 | (3%) | 2 | (2%) | 1 | (1%) | 3 | (3%) | 2 | (1%) | 4 | (2%) | 6 | (3%) |
| Bacterial infection-deep tissue/other normally sterile site | 1 | (1%) | 1 | (1%) | 2 | (2%) | 1 | (1%) | 2 | (2%) | 3 | (3%) | 2 | (1%) | 3 | (1%) | 5 | (2%) |
| Bacterial sepsis/catheter related bacteremia/sepsis | 1 | (1%) | 2 | (2%) | 3 | (3%) | 2 | (2%) | 0 | (0%) | 2 | (2%) | 3 | (1%) | 2 | (1%) | 5 | (2%) |
| Catheter exit site and/or tunnel infection | 0 | (0%) | 0 | (0%) | 0 | (0%) | 1 | (1%) | 0 | (0%) | 1 | (1%) | 1 | (0%) | 0 | (0%) | 1 | (0%) |
| **Serious Cardiovascular Disease** | **7** | **(7%)** | **3** | **(3%)** | **10** | **(9%)** | **4** | **(3%)** | **1** | **(1%)** | **5** | **(4%)** | **11** | **(5%)** | **4** | **(2%)** | **15** | **(7%)** |
| Other cardiovascular diseases - specify | 3 | (3%) | 2 | (2%) | 5 | (5%) | 2 | (2%) | 1 | (1%) | 3 | (3%) | 5 | (2%) | 3 | (1%) | 8 | (4%) |
| Stroke, specify hemorrhagic, ischemic, or unknown | 0 | (0%) | 1 | (1%) | 1 | (1%) | 2 | (2%) | 0 | (0%) | 2 | (2%) | 2 | (1%) | 1 | (0%) | 3 | (1%) |
| Significant arrhythmia | 2 | (2%) | 0 | (0%) | 2 | (2%) | 0 | (0%) | 0 | (0%) | 0 | (0%) | 2 | (1%) | 0 | (0%) | 2 | (1%) |
| Other cardiovascular diseases - cardiomyopathy, etiology unknown | 2 | (2%) | 0 | (0%) | 2 | (2%) | 0 | (0%) | 0 | (0%) | 0 | (0%) | 2 | (1%) | 0 | (0%) | 2 | (1%) |
| Other cardiovascular diseases - shock | 1 | (1%) | 0 | (0%) | 1 | (1%) | 1 | (1%) | 0 | (0%) | 1 | (1%) | 2 | (1%) | 0 | (0%) | 2 | (1%) |
| **Serious Liver Disease** | **3** | **(3%)** | **6** | **(6%)** | **9** | **(8%)** | **7** | **(6%)** | **8** | **(7%)** | **15** | **(13%)** | **10** | **(4%)** | **14** | **(6%)** | **24** | **(11%)** |
| Liver disease | 3 | (3%) | 4 | (4%) | 7 | (7%) | 4 | (3%) | 8 | (7%) | 12 | (10%) | 7 | (3%) | 12 | (5%) | 19 | (9%) |
| Chronic hepatitis b | 0 | (0%) | 2 | (2%) | 2 | (2%) | 2 | (2%) | 1 | (1%) | 3 | (3%) | 2 | (1%) | 3 | (1%) | 5 | (2%) |
| Chronic hepatitis c | 0 | (0%) | 0 | (0%) | 0 | (0%) | 2 | (2%) | 0 | (0%) | 2 | (2%) | 2 | (1%) | 0 | (0%) | 2 | (1%) |
| **Serious Malignancy** | **1** | **(1%)** | **0** | **(0%)** | **1** | **(1%)** | **0** | **(0%)** | **0** | **(0%)** | **0** | **(0%)** | **1** | **(0%)** | **0** | **(0%)** | **1** | **(0%)** |
| Malignancy | 1 | (1%) | 0 | (0%) | 1 | (1%) | 0 | (0%) | 0 | (0%) | 0 | (0%) | 1 | (0%) | 0 | (0%) | 1 | (0%) |
| **Serious Metabolic Disease** | **3** | **(3%)** | **9** | **(8%)** | **12** | **(11%)** | **12** | **(10%)** | **6** | **(5%)** | **18** | **(15%)** | **15** | **(7%)** | **15** | **(7%)** | **30** | **(13%)** |
| Pancreatitis documentation, symptomatic | 0 | (0%) | 2 | (2%) | 2 | (2%) | 5 | (4%) | 4 | (3%) | 9 | (8%) | 5 | (2%) | 6 | (3%) | 11 | (5%) |
| Lipoatrophy/fat loss (lipodystrophy) | 1 | (1%) | 5 | (5%) | 6 | (6%) | 1 | (1%) | 0 | (0%) | 1 | (1%) | 2 | (1%) | 5 | (2%) | 7 | (3%) |
| Diabetes mellitus/impaired glucose - diabetes mellitus | 0 | (0%) | 0 | (0%) | 0 | (0%) | 3 | (3%) | 2 | (2%) | 5 | (4%) | 3 | (1%) | 2 | (1%) | 5 | (2%) |
| Lactic acidemia/lactic acidosis - lactic acidosis | 1 | (1%) | 2 | (2%) | 3 | (3%) | 1 | (1%) | 0 | (0%) | 1 | (1%) | 2 | (1%) | 2 | (1%) | 4 | (2%) |
| Pancreatitis documentation - chemical or asymptomatic | 0 | (0%) | 0 | (0%) | 0 | (0%) | 1 | (1%) | 1 | (1%) | 2 | (2%) | 1 | (0%) | 1 | (0%) | 2 | (1%) |
| Fat accumulation (lipodystrophy) | 1 | (1%) | 1 | (1%) | 2 | (2%) | 0 | (0%) | 0 | (0%) | 0 | (0%) | 1 | (0%) | 1 | (0%) | 2 | (1%) |
| Metabolic/endocrine - hypogonadism | 0 | (0%) | 0 | (0%) | 0 | (0%) | 1 | (1%) | 0 | (0%) | 1 | (1%) | 1 | (0%) | 0 | (0%) | 1 | (0%) |
| **Serious Musculosketal Disease** | **2** | **(2%)** | **2** | **(2%)** | **4** | **(4%)** | **7** | **(6%)** | **2** | **(2%)** | **9** | **(8%)** | **9** | **(4%)** | **4** | **(2%)** | **13** | **(6%)** |
| Musculoskeletal - fracture | 1 | (1%) | 0 | (0%) | 1 | (1%) | 5 | (4%) | 0 | (0%) | 5 | (4%) | 6 | (3%) | 0 | (0%) | 6 | (3%) |
| Musculoskeletal - arthritis | 1 | (1%) | 1 | (1%) | 2 | (2%) | 2 | (2%) | 2 | (2%) | 4 | (3%) | 3 | (1%) | 3 | (1%) | 6 | (3%) |
| Musculoskeletal - myositis | 0 | (0%) | 1 | (1%) | 1 | (1%) | 0 | (0%) | 0 | (0%) | 0 | (0%) | 0 | (0%) | 1 | (0%) | 1 | (0%) |
| **Serious Neuropsychiatric Disease** | **15** | **(14%)** | **20** | **(19%)** | **35** | **(33%)** | **12** | **(10%)** | **17** | **(15%)** | **29** | **(25%)** | **27** | **(12%)** | **37** | **(17%)** | **64** | **(29%)** |
| Sensory neuropathy | 8 | (8%) | 10 | (9%) | 18 | (17%) | 6 | (5%) | 13 | (11%) | 19 | (16%) | 14 | (6%) | 23 | (10%) | 37 | (17%) |
| Depression | 2 | (2%) | 4 | (4%) | 6 | (6%) | 2 | (2%) | 3 | (3%) | 5 | (4%) | 4 | (2%) | 7 | (3%) | 11 | (5%) |
| Suicidal ideation | 3 | (3%) | 2 | (2%) | 5 | (5%) | 0 | (0%) | 1 | (1%) | 1 | (1%) | 3 | (1%) | 3 | (1%) | 6 | (3%) |
| Seizure disorder (not epilepsy) | 0 | (0%) | 0 | (0%) | 0 | (0%) | 2 | (2%) | 1 | (1%) | 3 | (3%) | 2 | (1%) | 1 | (0%) | 3 | (1%) |
| Psychosis | 2 | (2%) | 1 | (1%) | 3 | (3%) | 0 | (0%) | 0 | (0%) | 0 | (0%) | 2 | (1%) | 1 | (0%) | 3 | (1%) |
| CNS disease/disorder, other | 0 | (0%) | 1 | (1%) | 1 | (1%) | 1 | (1%) | 1 | (1%) | 2 | (2%) | 1 | (0%) | 2 | (1%) | 3 | (1%) |
| Focal neurological deficit | 0 | (0%) | 2 | (2%) | 2 | (2%) | 0 | (0%) | 0 | (0%) | 0 | (0%) | 0 | (0%) | 2 | (1%) | 2 | (1%) |
| Epilepsy | 0 | (0%) | 0 | (0%) | 0 | (0%) | 0 | (0%) | 1 | (1%) | 1 | (1%) | 0 | (0%) | 1 | (0%) | 1 | (0%) |
| Anxiety | 0 | (0%) | 0 | (0%) | 0 | (0%) | 0 | (0%) | 1 | (1%) | 1 | (1%) | 0 | (0%) | 1 | (0%) | 1 | (0%) |
| Neurologic and/or psychiatric - mental status impairment | 1 | (1%) | 0 | (0%) | 1 | (1%) | 0 | (0%) | 0 | (0%) | 0 | (0%) | 1 | (0%) | 0 | (0%) | 1 | (0%) |
| Peripheral nerve disease/disorder, other | 0 | (0%) | 0 | (0%) | 0 | (0%) | 1 | (1%) | 0 | (0%) | 1 | (1%) | 1 | (0%) | 0 | (0%) | 1 | (0%) |
| **Serious Pulmonary Disease** | **2** | **(2%)** | **0** | **(0%)** | **2** | **(2%)** | **2** | **(2%)** | **1** | **(1%)** | **3** | **(3%)** | **4** | **(2%)** | **1** | **(0%)** | **5** | **(2%)** |
| Pulmonary - respiratory disease/disorder, other | 1 | (1%) | 0 | (0%) | 1 | (1%) | 1 | (1%) | 1 | (1%) | 2 | (2%) | 2 | (1%) | 1 | (0%) | 3 | (1%) |
| Pulmonary embolus | 0 | (0%) | 0 | (0%) | 0 | (0%) | 1 | (1%) | 0 | (0%) | 1 | (1%) | 1 | (0%) | 0 | (0%) | 1 | (0%) |
| Pulmonary - respiratory failure | 1 | (1%) | 0 | (0%) | 1 | (1%) | 0 | (0%) | 0 | (0%) | 0 | (0%) | 1 | (0%) | 0 | (0%) | 1 | (0%) |
| **Serious Renal Disease** | **4** | **(4%)** | **1** | **(1%)** | **5** | **(5%)** | **7** | **(6%)** | **12** | **(10%)** | **19** | **(16%)** | **11** | **(5%)** | **13** | **(6%)** | **24** | **(11%)** |
| Genitourinary - renal system disease/disorder, other | 3 | (3%) | 1 | (1%) | 4 | (4%) | 4 | (3%) | 12 | (10%) | 16 | (14%) | 7 | (3%) | 13 | (6%) | 20 | (9%) |
| Acute renal insufficiency | 1 | (1%) | 0 | (0%) | 1 | (1%) | 2 | (2%) | 0 | (0%) | 2 | (2%) | 3 | (1%) | 0 | (0%) | 3 | (1%) |
| Genitourinary - nephrolithiasis | 0 | (0%) | 0 | (0%) | 0 | (0%) | 1 | (1%) | 0 | (0%) | 1 | (1%) | 1 | (0%) | 0 | (0%) | 1 | (0%) |
