# Supplementary material for: Efficacy and Safety of Three Antiretroviral Regimens for Initial Treatment of HIV-1: A Randomized Clinical Trial in Diverse Multinational Settings
Source: PLoS Med. 2012 Aug 14;9(8):e1001290. doi: 10.1371/journal.pmed.1001290 (PMC3419182; doi:10.1371/journal.pmed.1001290)
Supplement: Table S6 — New serious non-AIDS diagnosis categories compared for ATV+DDI-EC+FTC versus EFV+3TC-ZDV. (DOC) [file pmed.1001290.s011.doc]

**Table S6:** Comparison of Serious Non-AIDS Diagnoses (SNADES) categories by randomized treatment arms: atazanavir plus didanosine-EC and emtricitabine (ATV+DDI-EC+FTC) versus efavirenz plus lamivudine-zidovudine (EFV+3TC-ZDV)

|  | | | **Randomized Group** | | | |  | | | | |
| --- | --- | --- | --- | --- | --- | --- | --- | --- | --- | --- | --- |
| **SNADES Category** | |  | **EFV+**  **3TC-ZDV** | | **ATV+DDI-EC+FTC** | | **Total** | | **P-Value*** | | |
| Serious Bacterial Infection | yes | | | 48 (9%) | | 63 (12%) | | 111 (11%) | | 0.161 |  |
|  | no | | | 471 (91%) | | 463 (88%) | | 934 (89%) | |  |  |
|  | | | | | | | | | | |  |
| Serious Cardiovascular Disease | yes | | | 10 (2%) | | 5 (1%) | | 15 (1%) | | 0.204 |  |
|  | no | | | 509 (98%) | | 521 (99%) | | 1,030 (99%) | |  |  |
|  | | | | | | | | | | |  |
| Serious Liver Disease | yes | | | 9 (2%) | | 15 (3%) | | 24 (2%) | | 0.302 |  |
|  | no | | | 510 (98%) | | 511 (97%) | | 1,021 (98%) | |  |  |
|  | | | | | | | | | | |  |
| Serious Malignancy | yes | | | 1 (0%) | | 0 (0%) | | 1 (0%) | | 0.497 |  |
|  | no | | | 518 (100%) | | 526 (100%) | | 1,044 (100%) | |  |  |
|  | | | | | | | | | | |  |
| Serious Metabolic Disease | yes | | | 12 (2%) | | 18 (3%) | | 30 (3%) | | 0.355 |  |
|  | no | | | 507 (98%) | | 508 (97%) | | 1,015 (97%) | |  |  |
|  | | | | | | | | | | |  |
| Serious Musculosketal Disease | yes | | | 4 (1%) | | 9 (2%) | | 13 (1%) | | 0.264 |  |
|  | no | | | 515 (99%) | | 517 (98%) | | 1,032 (99%) | |  |  |
|  | | | | | | | | | | |  |
| Serious Neuropsychiatric Disease | yes | | | 35 (7%) | | 29 (6%) | | 64 (6%) | | 0.440 |  |
|  | no | | | 484 (93%) | | 497 (94%) | | 981 (94%) | |  |  |
|  | | | | | | | | | | |  |
| Serious Pulmonary Disease | yes | | | 2 (0%) | | 3 (1%) | | 5 (0%) | | 1.000 |  |
|  | no | | | 517 (100%) | | 523 (99%) | | 1,040 (100%) | |  |  |
|  | | | | | | | | | | |  |
| Serious Renal Disease | yes | | | 5 (1%) | | 19 (4%) | | 24 (2%) | | 0.006 |  |
|  | no | | | 514 (99%) | | 507 (96%) | | 1,021 (98%) | |  |  |
|  | | | | | | | | | | | |
| ***Fisher's Exact Test | | | | | | | | | | | |
