# Supplementary material for: Efficacy and Safety of Three Antiretroviral Regimens for Initial Treatment of HIV-1: A Randomized Clinical Trial in Diverse Multinational Settings
Source: PLoS Med. 2012 Aug 14;9(8):e1001290. doi: 10.1371/journal.pmed.1001290 (PMC3419182; doi:10.1371/journal.pmed.1001290)
Supplement: Table S7 — Type of opportunistic infections observed for comparison of EFV+FTC-TDF to EFV+3TC-ZDV. (DOC) [file pmed.1001290.s012.doc]

**Table S7:** Type of opportunistic infections observed through 31-May-2010 according to randomized treatment arm: efavierenz plus emtricitabine-tenofovir-DF (EFV+FTC-TDF) and efavirenz plus lamivudine-zidovudine (EFV+3TC-ZDV)

|  | | **Randomized Group** | |  |
| --- | --- | --- | --- | --- |
|  |  | **EFV+**  **3TC-ZDV** | **EFV+**  **FTC-TDF** | **Total** |
|  | Extra pulmonary tuberculosis | 9 (36%) | 7 (32%) | 16 (34%) |
|  | Bacterial pneumonia | 4 (16%) | 5 (23%) | 9 (19%) |
|  | *Pneumocystis jiroveci* pneumonia (PCP) | 3 (12%) | 4 (18%) | 7 (15%) |
|  | Mucocutaneous herpes simplex | 4 (16%) | 1 (5%) | 5 (11%) |
|  | Kaposi sarcoma (KS) mucocutaneous and visceral | 0 (0%) | 2 (9%) | 2 (4%) |
|  | Mycobacterium avium complex (MAC) | 2 (8%) | 0 (0%) | 2 (4%) |
|  | Systemic non-Hodgkin lymphoma (NHL) | 1 (4%) | 1 (5%) | 2 (4%) |
|  | Toxoplasmic encephalitis | 1 (4%) | 1 (5%) | 2 (4%) |
|  | Cryptosporidiosis | 0 (0%) | 1 (5%) | 1 (2%) |
|  | Progressive multifocal encephalopathy | 1 (4%) | 0 (0%) | 1 (2%) |
|  | | | | |
