# Supplementary material for: Efficacy and Safety of Three Antiretroviral Regimens for Initial Treatment of HIV-1: A Randomized Clinical Trial in Diverse Multinational Settings
Source: PLoS Med. 2012 Aug 14;9(8):e1001290. doi: 10.1371/journal.pmed.1001290 (PMC3419182; doi:10.1371/journal.pmed.1001290)
Supplement: Table S8 — All new laboratory events of grade 3 or higher for comparison of EFV+FTC-TDF to EFV+3TC-ZDV. (DOC) [file pmed.1001290.s013.doc]

**Table S8:** All new laboratory events of grade 3 or higher through 31-May-2010 for the comparison of efavirenz plus emtricitabine-tenofovir-DF (EFV+FTC-TDF) to efavirenz plus lamivudine-zidovudine (EFV+3TC-ZDV)

|  | **Randomized Group** | | | | | | | | |
| --- | --- | --- | --- | --- | --- | --- | --- | --- | --- |
|  | **EFV+3TC-ZDV (N=519)** | | | **EFV+FTC-TDF (N=526)** | | | **All (N=1045)** | | |
|  | **Grade** | |  | **Grade** | |  | **Grade** | |  |
| **Laboratory Event** | **3** | **4** | **Number subjects** | **3** | **4** | **Number subjects** | **3** | **4** | **Number subjects** |
| Any Chemistry | 45 (9%) | 9 (2%) | 54 (10%) | 49 (9%) | 10 (2%) | 59 (11%) | 94 (9%) | 19 (2%) | 113 (11%) |
| Any Chemistry, General | 45 | 9 | 54 | 49 | 10 | 59 | 94 | 19 | 113 |
| Albumin | 7 | 0 | 7 | 5 | 0 | 5 | 12 | 0 | 12 |
| Alkaline Phosphatase | 2 | 2 | 4 | 1 | 0 | 1 | 3 | 2 | 5 |
| Bicarbonate | 2 | 1 | 3 | 1 | 2 | 3 | 3 | 3 | 6 |
| Calcium | 0 | 1 | 1 | 1 | 2 | 3 | 1 | 3 | 4 |
| Carbon Dioxide | 1 | 0 | 1 | 1 | 0 | 1 | 2 | 0 | 2 |
| Creatine Kinase | 0 | 2 | 2 | 0 | 1 | 1 | 0 | 3 | 3 |
| Phosphorus | 38 | 1 | 39 | 45 | 1 | 46 | 83 | 2 | 85 |
| Potassium | 2 | 1 | 3 | 1 | 4 | 5 | 3 | 5 | 8 |
| Sodium | 2 | 2 | 4 | 4 | 2 | 6 | 6 | 4 | 10 |
| Any Endocrine | 0 (0%) | 0 (0%) | 0 (0%) | 1 (0%) | 0 (0%) | 1 (0%) | 1 (0%) | 0 (0%) | 1 (0%) |
| Any Endocrine, Metabolic | 0 | 0 | 0 | 1 | 0 | 1 | 1 | 0 | 1 |
| Fasting Blood Sugar | 0 | 0 | 0 | 1 | 0 | 1 | 1 | 0 | 1 |
| Any Hematology | 64 (12%) | 41 (8%) | 105 (20%) | 37 (7%) | 19 (4%) | 56 (11%) | 101 (10%) | 60 (6%) | 161 (15%) |
| Any Hematology, Coagulation | 6 | 5 | 11 | 3 | 5 | 8 | 9 | 10 | 19 |
| Platelets | 6 | 5 | 11 | 3 | 5 | 8 | 9 | 10 | 19 |
| Any Hematology, RBC | 12 | 11 | 23 | 8 | 10 | 18 | 20 | 21 | 41 |
| Hemoglobin | 12 | 11 | 23 | 8 | 10 | 18 | 20 | 21 | 41 |
| Any Hematology, WBC/Differential | 53 | 26 | 79 | 32 | 8 | 40 | 85 | 34 | 119 |
| Absolute Neutrophil Count | 51 | 26 | 77 | 32 | 8 | 40 | 83 | 34 | 117 |
| White Blood Cells | 3 | 0 | 3 | 2 | 0 | 2 | 5 | 0 | 5 |
| Any Metabolic | 10 (2%) | 4 (1%) | 14 (3%) | 4 (1%) | 0 (0%) | 4 (1%) | 14 (1%) | 4 (0%) | 18 (2%) |
| Calculated LDL (FASTING) | 0 | 0 | 0 | 1 | 0 | 1 | 1 | 0 | 1 |
| Glucose (NON-FASTING) | 2 | 3 | 5 | 2 | 0 | 2 | 4 | 3 | 7 |
| Lactacte | 4 | 0 | 4 | 0 | 0 | 0 | 4 | 0 | 4 |
| LDL (FASTING) | 1 | 0 | 1 | 0 | 0 | 0 | 1 | 0 | 1 |
| Total Cholesterol (FASTING) | 3 | 0 | 3 | 1 | 0 | 1 | 4 | 0 | 4 |
| Total Triglycerides (FASTING) | 1 | 1 | 2 | 0 | 0 | 0 | 1 | 1 | 2 |
| Any Liver/Hepatic | 32 (6%) | 20 (4%) | 52 (10%) | 26 (5%) | 13 (2%) | 39 (7%) | 58 (6%) | 33 (3%) | 91 (9%) |
| SGOT/AST | 15 | 15 | 30 | 11 | 6 | 17 | 26 | 21 | 47 |
| SGPT/ALT | 11 | 10 | 21 | 11 | 6 | 17 | 22 | 16 | 38 |
| Total Bilirubin | 19 | 7 | 26 | 15 | 9 | 24 | 34 | 16 | 50 |
| Any Renal | 1 (0%) | 2 (0%) | 3 (1%) | 3 (1%) | 4 (1%) | 7 (1%) | 4 (0%) | 6 (1%) | 10 (1%) |
| Creatinine | 1 | 2 | 3 | 3 | 4 | 7 | 4 | 6 | 10 |
| Any Pancreatic | 0 (0%) | 4 (1%) | 4 (1%) | 1 (0%) | 1 (0%) | 2 (0%) | 1 (0%) | 5 (0%) | 6 (1%) |
| Lipase | 0 | 4 | 4 | 1 | 1 | 2 | 1 | 5 | 6 |
| Pancreatic Serum Amylase | 0 | 1 | 1 | 0 | 0 | 0 | 0 | 1 | 1 |
| Any Other | 0 (0%) | 1 (0%) | 1 (0%) | 0 (0%) | 0 (0%) | 0 (0%) | 0 (0%) | 1 (0%) | 1 (0%) |
| INR | 0 | 1 | 1 | 0 | 0 | 0 | 0 | 1 | 1 |
| Any event | 112 (22%) | 66 (13%) | 178 (34%) | 94 (18%) | 41 (8%) | 135 (26%) | 206 (20%) | 107 (10%) | 313 (30%) |

Multiple episodes or adverse events on same row are counted only once. DAIDS Severity Grading: 3 = Severe, 4 = Life-Threatening. Worst grade for each AE category is presented and only follow-up during initial antiretroviral regimen included.
