# Supplementary material for: Efficacy and Safety of Three Antiretroviral Regimens for Initial Treatment of HIV-1: A Randomized Clinical Trial in Diverse Multinational Settings
Source: PLoS Med. 2012 Aug 14;9(8):e1001290. doi: 10.1371/journal.pmed.1001290 (PMC3419182; doi:10.1371/journal.pmed.1001290)
Supplement: Table S9 — All new signs and symptoms of grade 3 or higher for comparison of EFV+FTC-TDF to EFV+3TC-ZDV. (DOC) [file pmed.1001290.s014.doc]

**Table S9:** All new signs and symptoms of grade 3 or higher through 31-May-2010 for the comparison of efavirenz plus emtricitabine-tenofovir-DF (EFV+FTC-TDF) to efavirenz plus lamivudine-zidovudine (EFV+3TC-ZDV)

|  | **Randomized Group** | | | | | | | | |
| --- | --- | --- | --- | --- | --- | --- | --- | --- | --- |
|  | **EFV+3TC-ZDV (N=519)** | | | **EFV+FTC-TDF (N=526)** | | | **All (N=1045)** | | |
|  | **Grade** | |  | **Grade** | |  | **Grade** | |  |
| **Sign/Symptom** | **3** | **4** | **Number subjects** | **3** | **4** | **Number subjects** | **3** | **4** | **Number subjects** |
| Any General Body | 60 (12%) | 5 (1%) | 65 (13%) | 51 (10%) | 9 (2%) | 60 (11%) | 111 (11%) | 14 (1%) | 125 (12%) |
| Abnormal Physical Appearance | 2 | 0 | 2 | 3 | 0 | 3 | 5 | 0 | 5 |
| Ache/Pain/discomfort | 32 | 3 | 35 | 24 | 5 | 29 | 56 | 8 | 64 |
| Asthenia/Fatigue/malaise | 9 | 0 | 9 | 9 | 0 | 9 | 18 | 0 | 18 |
| Cachexia/Wasting/weight Loss | 19 | 2 | 21 | 13 | 3 | 16 | 32 | 5 | 37 |
| Chills/Rigors/sweats/nightsweats | 1 | 0 | 1 | 0 | 0 | 0 | 1 | 0 | 1 |
| Fever | 12 | 0 | 12 | 10 | 1 | 11 | 22 | 1 | 23 |
| Any Respiratory | 15 (3%) | 1 (0%) | 16 (3%) | 15 (3%) | 1 (0%) | 16 (3%) | 30 (3%) | 2 (0%) | 32 (3%) |
| Cough | 4 | 0 | 4 | 8 | 1 | 9 | 12 | 1 | 13 |
| Difficulty Breathing/Dyspnea/sob | 11 | 1 | 12 | 7 | 1 | 8 | 18 | 2 | 20 |
| Respiratory System Dysfunction | 1 | 0 | 1 | 1 | 0 | 1 | 2 | 0 | 2 |
| Voice Abnormality/Alteration | 0 | 0 | 0 | 1 | 0 | 1 | 1 | 0 | 1 |
| Any Circulatory/Cardiac | 16 (3%) | 1 (0%) | 17 (3%) | 6 (1%) | 0 (0%) | 6 (1%) | 22 (2%) | 1 (0%) | 23 (2%) |
| Cardiovascular Dysfunction | 3 | 1 | 4 | 3 | 0 | 3 | 6 | 1 | 7 |
| Edema/Enlarged/swollen | 10 | 0 | 10 | 2 | 0 | 2 | 12 | 0 | 12 |
| Heart Rate/Rhythm Abnormal | 3 | 0 | 3 | 1 | 0 | 1 | 4 | 0 | 4 |
| Any Hematology | 3 (1%) | 0 (0%) | 3 (1%) | 6 (1%) | 0 (0%) | 6 (1%) | 9 (1%) | 0 (0%) | 9 (1%) |
| Any Hematology, Signs and Symptoms | 3 | 0 | 3 | 6 | 0 | 6 | 9 | 0 | 9 |
| Bleeding/Bruising/petechiae | 1 | 0 | 1 | 6 | 0 | 6 | 7 | 0 | 7 |
| Lymphadenopathy | 2 | 0 | 2 | 0 | 0 | 0 | 2 | 0 | 2 |
| Any Liver/Hepatic | 0 (0%) | 0 (0%) | 0 (0%) | 0 (0%) | 1 (0%) | 1 (0%) | 0 (0%) | 1 (0%) | 1 (0%) |
| Jaundice | 0 | 0 | 0 | 0 | 1 | 1 | 0 | 1 | 1 |
| Any Gastro-Intestinal | 21 (4%) | 1 (0%) | 22 (4%) | 22 (4%) | 6 (1%) | 28 (5%) | 43 (4%) | 7 (1%) | 50 (5%) |
| Appetite Loss/Decreased/anorexia | 4 | 0 | 4 | 6 | 0 | 6 | 10 | 0 | 10 |
| Diarrhea/Loose Stools | 10 | 1 | 11 | 10 | 1 | 11 | 20 | 2 | 22 |
| Distention/Flatus/gas | 1 | 0 | 1 | 0 | 1 | 1 | 1 | 1 | 2 |
| Nausea | 5 | 0 | 5 | 5 | 1 | 6 | 10 | 1 | 11 |
| Nausea And Vomiting/Vomiting | 7 | 1 | 8 | 10 | 5 | 15 | 17 | 6 | 23 |
| Any Renal | 0 (0%) | 1 (0%) | 1 (0%) | 0 (0%) | 0 (0%) | 0 (0%) | 0 (0%) | 1 (0%) | 1 (0%) |
| Renal/Urinary System Dysfunction | 0 | 1 | 1 | 0 | 0 | 0 | 0 | 1 | 1 |
| Any Reproductive | 1 (0%) | 0 (0%) | 1 (0%) | 2 (0%) | 1 (0%) | 3 (1%) | 3 (0%) | 1 (0%) | 4 (0%) |
| Discharge - - Malodorous | 0 | 0 | 0 | 0 | 1 | 1 | 0 | 1 | 1 |
| Discharge/Exudate/pus | 1 | 0 | 1 | 0 | 0 | 0 | 1 | 0 | 1 |
| Menstrual Dysfunction | 0 | 0 | 0 | 2 | 0 | 2 | 2 | 0 | 2 |
| Any Skin | 15 (3%) | 1 (0%) | 16 (3%) | 11 (2%) | 2 (0%) | 13 (2%) | 26 (2%) | 3 (0%) | 29 (3%) |
| Allergic Rash/Urticaria/welts/hives | 1 | 0 | 1 | 3 | 0 | 3 | 4 | 0 | 4 |
| Blister/Ulcer/lesions | 4 | 0 | 4 | 1 | 1 | 2 | 5 | 1 | 6 |
| Discoloration/Dyschromia | 0 | 0 | 0 | 1 | 0 | 1 | 1 | 0 | 1 |
| Erythema/Redness/inflammation | 2 | 0 | 2 | 1 | 0 | 1 | 3 | 0 | 3 |
| Induration | 2 | 0 | 2 | 0 | 0 | 0 | 2 | 0 | 2 |
| Itchy/Pruritus | 1 | 0 | 1 | 2 | 0 | 2 | 3 | 0 | 3 |
| Macules/Papules/rash | 10 | 0 | 10 | 5 | 0 | 5 | 15 | 0 | 15 |
| Mucous Membrane/Skin Abnormality | 0 | 1 | 1 | 1 | 1 | 2 | 1 | 2 | 3 |
| Patch/Plaque | 1 | 0 | 1 | 0 | 0 | 0 | 1 | 0 | 1 |
| Any Neurological | 25 (5%) | 6 (1%) | 31 (6%) | 27 (5%) | 9 (2%) | 36 (7%) | 52 (5%) | 15 (1%) | 67 (6%) |
| Agitation/Hyperactive | 0 | 0 | 0 | 3 | 0 | 3 | 3 | 0 | 3 |
| Confusion/Difficulty Concentrating | 3 | 1 | 4 | 3 | 0 | 3 | 6 | 1 | 7 |
| Consciousness Level Change/Lethargy | 3 | 2 | 5 | 2 | 0 | 2 | 5 | 2 | 7 |
| Convulsion/Seizure | 2 | 0 | 2 | 0 | 0 | 0 | 2 | 0 | 2 |
| Depression | 2 | 2 | 4 | 6 | 4 | 10 | 8 | 6 | 14 |
| Diplopia/Vision Blurred/Alteration | 3 | 0 | 3 | 0 | 0 | 0 | 3 | 0 | 3 |
| Dreams/Insomnia/sleeping Problems | 3 | 0 | 3 | 4 | 0 | 4 | 7 | 0 | 7 |
| Headache | 10 | 2 | 12 | 7 | 3 | 10 | 17 | 5 | 22 |
| Inappropriate/Changed Behavior | 3 | 0 | 3 | 3 | 0 | 3 | 6 | 0 | 6 |
| Memory Loss | 1 | 0 | 1 | 0 | 0 | 0 | 1 | 0 | 1 |
| Mental Status Changes | 4 | 0 | 4 | 6 | 2 | 8 | 10 | 2 | 12 |
| Neurologic Dysfunction | 1 | 0 | 1 | 3 | 0 | 3 | 4 | 0 | 4 |
| Numbness/Paresthesia/tingling | 2 | 0 | 2 | 1 | 1 | 2 | 3 | 1 | 4 |
| Rigid/Tight/stiff | 1 | 0 | 1 | 0 | 0 | 0 | 1 | 0 | 1 |
| Weakness | 2 | 1 | 3 | 3 | 1 | 4 | 5 | 2 | 7 |
| Any Other | 17 (3%) | 2 (0%) | 19 (4%) | 14 (3%) | 1 (0%) | 15 (3%) | 31 (3%) | 3 (0%) | 34 (3%) |
| Dizzy/Lightheaded/fainting | 17 | 0 | 17 | 13 | 1 | 14 | 30 | 1 | 31 |
| Dry | 1 | 0 | 1 | 0 | 0 | 0 | 1 | 0 | 1 |
| Other | 0 | 2 | 2 | 1 | 0 | 1 | 1 | 2 | 3 |
| Any sign/symptom | 105 (20%) | 13 (3%) | 118 (23%) | 95 (18%) | 22 (4%) | 117 (22%) | 200 (19%) | 35 (3%) | 235 (22%) |

Multiple episodes or adverse events on same row are counted only once. DAIDS Severity Grading: 3 = Severe, 4 = Life-Threatening. Worst grade for each AE category is presented and only follow-up during initial antiretroviral regimen included.
