# Supplementary material for: Efficacy and Safety of Three Antiretroviral Regimens for Initial Treatment of HIV-1: A Randomized Clinical Trial in Diverse Multinational Settings
Source: PLoS Med. 2012 Aug 14;9(8):e1001290. doi: 10.1371/journal.pmed.1001290 (PMC3419182; doi:10.1371/journal.pmed.1001290)
Supplement: Table S10 — All new serious non-AIDS diagnoses for comparison of EFV+FTC-TDF to EFV+3TC-ZDV. (DOC) [file pmed.1001290.s015.doc]

**Table S10:** All New Serious Non-AIDS Diagnoses (SNADES) through 31-May-2010 for the comparison of efavirenz plus emtricitabine-tenofovir-DF (EFV+FTC-TDF) to efavirenz plus lamivudine-zidovudine (EFV+3TC-ZDV)

|  | **Randomized study group** | | | | | | | | | | | |  | | | | | |
| --- | --- | --- | --- | --- | --- | --- | --- | --- | --- | --- | --- | --- | --- | --- | --- | --- | --- | --- |
|  | **EFV+3TC-ZDV (N=151)** | | | | | | **EFV+FTC-TDF (N=151)** | | | | | | **All participants (N=302)** | | | | | |
|  | **Gender** | | | |  | | **Gender** | | | |  | | **Gender** | | | |  | |
| **SNADES Dx** | **M** | | **F** | | **Subtotal** | | **M** | | **F** | | **Subtotal** | | **M** | | **F** | | **Subtotal** | |
| **Overall** | **78** | **(52%)** | **73** | **(48%)** | **151** | **(100%)** | **76** | **(50%)** | **75** | **(50%)** | **151** | **(100%)** | **154** | **(51%)** | **148** | **(49%)** | **302** | **(100%)** |
| **Serious Bacterial Infection** | **31** | **(21%)** | **31** | **(21%)** | **62** | **(41%)** | **29** | **(19%)** | **32** | **(21%)** | **61** | **(40%)** | **60** | **(20%)** | **63** | **(21%)** | **123** | **(41%)** |
| Bacterial pneumonia | 14 | (9%) | 10 | (7%) | 24 | (16%) | 9 | (6%) | 15 | (10%) | 24 | (16%) | 23 | (8%) | 25 | (8%) | 48 | (16%) |
| Pulmonary tuberculosis | 10 | (7%) | 8 | (5%) | 18 | (12%) | 14 | (9%) | 10 | (7%) | 24 | (16%) | 24 | (8%) | 18 | (6%) | 42 | (14%) |
| Acute gastrointestinal/diarrheal syndrome | 8 | (5%) | 9 | (6%) | 17 | (11%) | 5 | (3%) | 7 | (5%) | 12 | (8%) | 13 | (4%) | 16 | (5%) | 29 | (10%) |
| Bacterial sepsis/catheter related bacteremia/sepsis | 4 | (3%) | 2 | (1%) | 6 | (4%) | 4 | (3%) | 1 | (1%) | 5 | (3%) | 8 | (3%) | 3 | (1%) | 11 | (4%) |
| Pelvic inflammatory disease | 0 | (0%) | 5 | (3%) | 5 | (3%) | 0 | (0%) | 5 | (3%) | 5 | (3%) | 0 | (0%) | 10 | (3%) | 10 | (3%) |
| Gastrointestinal - gastrointestinal system disease/disorder, other | 0 | (0%) | 1 | (1%) | 1 | (1%) | 1 | (1%) | 0 | (0%) | 1 | (1%) | 1 | (0%) | 1 | (0%) | 2 | (1%) |
| Bacterial infection-deep tissue/other normally sterile site | 1 | (1%) | 1 | (1%) | 2 | (1%) | 0 | (0%) | 0 | (0%) | 0 | (0%) | 1 | (0%) | 1 | (0%) | 2 | (1%) |
| Persistent diarrhea | 1 | (1%) | 0 | (0%) | 1 | (1%) | 0 | (0%) | 1 | (1%) | 1 | (1%) | 1 | (0%) | 1 | (0%) | 2 | (1%) |
| **Serious Cardiovascular Disease** | **9** | **(6%)** | **4** | **(3%)** | **13** | **(9%)** | **3** | **(2%)** | **4** | **(3%)** | **7** | **(5%)** | **12** | **(4%)** | **8** | **(3%)** | **20** | **(7%)** |
| Other cardiovascular diseases - specify | 4 | (3%) | 2 | (1%) | 6 | (4%) | 0 | (0%) | 0 | (0%) | 0 | (0%) | 4 | (1%) | 2 | (1%) | 6 | (2%) |
| Other cardiovascular diseases - cardiomyopathy, etiology unknown | 3 | (2%) | 1 | (1%) | 4 | (3%) | 0 | (0%) | 0 | (0%) | 0 | (0%) | 3 | (1%) | 1 | (0%) | 4 | (1%) |
| Other cardiovascular diseases - shock | 1 | (1%) | 1 | (1%) | 2 | (1%) | 0 | (0%) | 1 | (1%) | 1 | (1%) | 1 | (0%) | 2 | (1%) | 3 | (1%) |
| Stroke, specify hemorrhagic, ischemic, or unknown | 0 | (0%) | 1 | (1%) | 1 | (1%) | 0 | (0%) | 2 | (1%) | 2 | (1%) | 0 | (0%) | 3 | (1%) | 3 | (1%) |
| Significant arrhythmia | 2 | (1%) | 0 | (0%) | 2 | (1%) | 0 | (0%) | 0 | (0%) | 0 | (0%) | 2 | (1%) | 0 | (0%) | 2 | (1%) |
| Acute myocardial infarction (symptomatic) | 1 | (1%) | 0 | (0%) | 1 | (1%) | 0 | (0%) | 1 | (1%) | 1 | (1%) | 1 | (0%) | 1 | (0%) | 2 | (1%) |
| Stroke, clinical diagnosis only | 0 | (0%) | 0 | (0%) | 0 | (0%) | 1 | (1%) | 0 | (0%) | 1 | (1%) | 1 | (0%) | 0 | (0%) | 1 | (0%) |
| Deep vein thrombosis (dvt) | 0 | (0%) | 0 | (0%) | 0 | (0%) | 1 | (1%) | 0 | (0%) | 1 | (1%) | 1 | (0%) | 0 | (0%) | 1 | (0%) |
| Coronary heart disease (CHD)/coronary artery disease (CAD) | 0 | (0%) | 0 | (0%) | 0 | (0%) | 1 | (1%) | 0 | (0%) | 1 | (1%) | 1 | (0%) | 0 | (0%) | 1 | (0%) |
| Angina pectoris | 1 | (1%) | 0 | (0%) | 1 | (1%) | 0 | (0%) | 0 | (0%) | 0 | (0%) | 1 | (0%) | 0 | (0%) | 1 | (0%) |
| **Serious Liver Disease** | **5** | **(3%)** | **6** | **(4%)** | **11** | **(7%)** | **11** | **(7%)** | **2** | **(1%)** | **13** | **(9%)** | **16** | **(5%)** | **8** | **(3%)** | **24** | **(8%)** |
| Liver disease | 4 | (3%) | 4 | (3%) | 8 | (5%) | 10 | (7%) | 2 | (1%) | 12 | (8%) | 14 | (5%) | 6 | (2%) | 20 | (7%) |
| Acute hepatitis | 1 | (1%) | 0 | (0%) | 1 | (1%) | 0 | (0%) | 1 | (1%) | 1 | (1%) | 1 | (0%) | 1 | (0%) | 2 | (1%) |
| Chronic hepatitis b | 0 | (0%) | 2 | (1%) | 2 | (1%) | 0 | (0%) | 0 | (0%) | 0 | (0%) | 0 | (0%) | 2 | (1%) | 2 | (1%) |
| Gastrointestinal - gastrointestinal system disease/disorder, other | 0 | (0%) | 0 | (0%) | 0 | (0%) | 1 | (1%) | 0 | (0%) | 1 | (1%) | 1 | (0%) | 0 | (0%) | 1 | (0%) |
| **Serious Malignancy** | **2** | **(1%)** | **1** | **(1%)** | **3** | **(2%)** | **2** | **(1%)** | **0** | **(0%)** | **2** | **(1%)** | **4** | **(1%)** | **1** | **(0%)** | **5** | **(2%)** |
| Malignancy | 2 | (1%) | 1 | (1%) | 3 | (2%) | 2 | (1%) | 0 | (0%) | 2 | (1%) | 4 | (1%) | 1 | (0%) | 5 | (2%) |
| Dermatologic - skin disease/disorder, other | 1 | (1%) | 0 | (0%) | 1 | (1%) | 0 | (0%) | 0 | (0%) | 0 | (0%) | 1 | (0%) | 0 | (0%) | 1 | (0%) |
| **Serious Metabolic Disease** | **9** | **(6%)** | **10** | **(7%)** | **19** | **(13%)** | **1** | **(1%)** | **2** | **(1%)** | **3** | **(2%)** | **10** | **(3%)** | **12** | **(4%)** | **22** | **(7%)** |
| Lipoatrophy/fat loss (lipodystrophy) | 1 | (1%) | 6 | (4%) | 7 | (5%) | 0 | (0%) | 0 | (0%) | 0 | (0%) | 1 | (0%) | 6 | (2%) | 7 | (2%) |
| Pancreatitis documentation, symptomatic | 3 | (2%) | 2 | (1%) | 5 | (3%) | 0 | (0%) | 1 | (1%) | 1 | (1%) | 3 | (1%) | 3 | (1%) | 6 | (2%) |
| Diabetes mellitus/impaired glucose - diabetes mellitus | 2 | (1%) | 0 | (0%) | 2 | (1%) | 1 | (1%) | 0 | (0%) | 1 | (1%) | 3 | (1%) | 0 | (0%) | 3 | (1%) |
| Lactic acidemia/lactic acidosis - lactic acidosis | 1 | (1%) | 2 | (1%) | 3 | (2%) | 0 | (0%) | 0 | (0%) | 0 | (0%) | 1 | (0%) | 2 | (1%) | 3 | (1%) |
| Hyperthyroidism | 1 | (1%) | 0 | (0%) | 1 | (1%) | 0 | (0%) | 1 | (1%) | 1 | (1%) | 1 | (0%) | 1 | (0%) | 2 | (1%) |
| Fat accumulation (lipodystrophy) | 1 | (1%) | 1 | (1%) | 2 | (1%) | 0 | (0%) | 0 | (0%) | 0 | (0%) | 1 | (0%) | 1 | (0%) | 2 | (1%) |
| Metabolic/endocrine disorder, other | 1 | (1%) | 0 | (0%) | 1 | (1%) | 0 | (0%) | 0 | (0%) | 0 | (0%) | 1 | (0%) | 0 | (0%) | 1 | (0%) |
| **Serious Musculosketal Disease** | **6** | **(4%)** | **2** | **(1%)** | **8** | **(5%)** | **12** | **(8%)** | **1** | **(1%)** | **13** | **(9%)** | **18** | **(6%)** | **3** | **(1%)** | **21** | **(7%)** |
| Musculoskeletal - fracture | 5 | (3%) | 0 | (0%) | 5 | (3%) | 7 | (5%) | 0 | (0%) | 7 | (5%) | 12 | (4%) | 0 | (0%) | 12 | (4%) |
| Musculoskeletal - arthritis | 1 | (1%) | 1 | (1%) | 2 | (1%) | 4 | (3%) | 0 | (0%) | 4 | (3%) | 5 | (2%) | 1 | (0%) | 6 | (2%) |
| Musculoskeletal - avascular necrosis | 0 | (0%) | 0 | (0%) | 0 | (0%) | 1 | (1%) | 0 | (0%) | 1 | (1%) | 1 | (0%) | 0 | (0%) | 1 | (0%) |
| Osteopenia/osteoporosis - osteopenia | 0 | (0%) | 0 | (0%) | 0 | (0%) | 0 | (0%) | 1 | (1%) | 1 | (1%) | 0 | (0%) | 1 | (0%) | 1 | (0%) |
| Musculoskeletal - musculoskeletal system disease/disorder, other | 0 | (0%) | 0 | (0%) | 0 | (0%) | 1 | (1%) | 0 | (0%) | 1 | (1%) | 1 | (0%) | 0 | (0%) | 1 | (0%) |
| Musculoskeletal - myositis | 0 | (0%) | 1 | (1%) | 1 | (1%) | 0 | (0%) | 0 | (0%) | 0 | (0%) | 0 | (0%) | 1 | (0%) | 1 | (0%) |
| **Serious Neuropsychiatric Disease** | **20** | **(13%)** | **27** | **(18%)** | **47** | **(31%)** | **23** | **(15%)** | **21** | **(14%)** | **44** | **(29%)** | **43** | **(14%)** | **48** | **(16%)** | **91** | **(30%)** |
| Sensory neuropathy | 9 | (6%) | 11 | (7%) | 20 | (13%) | 8 | (5%) | 14 | (9%) | 22 | (15%) | 17 | (6%) | 25 | (8%) | 42 | (14%) |
| Depression | 4 | (3%) | 9 | (6%) | 13 | (9%) | 8 | (5%) | 4 | (3%) | 12 | (8%) | 12 | (4%) | 13 | (4%) | 25 | (8%) |
| Suicidal ideation | 3 | (2%) | 3 | (2%) | 6 | (4%) | 6 | (4%) | 3 | (2%) | 9 | (6%) | 9 | (3%) | 6 | (2%) | 15 | (5%) |
| CNS disease/disorder, other | 1 | (1%) | 2 | (1%) | 3 | (2%) | 4 | (3%) | 0 | (0%) | 4 | (3%) | 5 | (2%) | 2 | (1%) | 7 | (2%) |
| Psychosis | 2 | (1%) | 1 | (1%) | 3 | (2%) | 0 | (0%) | 0 | (0%) | 0 | (0%) | 2 | (1%) | 1 | (0%) | 3 | (1%) |
| Focal neurological deficit | 0 | (0%) | 2 | (1%) | 2 | (1%) | 0 | (0%) | 0 | (0%) | 0 | (0%) | 0 | (0%) | 2 | (1%) | 2 | (1%) |
| Neurologic and/or psychiatric - mental status impairment | 1 | (1%) | 0 | (0%) | 1 | (1%) | 0 | (0%) | 1 | (1%) | 1 | (1%) | 1 | (0%) | 1 | (0%) | 2 | (1%) |
| Epilepsy | 0 | (0%) | 0 | (0%) | 0 | (0%) | 0 | (0%) | 1 | (1%) | 1 | (1%) | 0 | (0%) | 1 | (0%) | 1 | (0%) |
| Peripheral nerve disease/disorder, other | 1 | (1%) | 0 | (0%) | 1 | (1%) | 0 | (0%) | 0 | (0%) | 0 | (0%) | 1 | (0%) | 0 | (0%) | 1 | (0%) |
| Hallucinations | 0 | (0%) | 0 | (0%) | 0 | (0%) | 1 | (1%) | 0 | (0%) | 1 | (1%) | 1 | (0%) | 0 | (0%) | 1 | (0%) |
| **Serious Pulmonary Disease** | **3** | **(2%)** | **0** | **(0%)** | **3** | **(2%)** | **1** | **(1%)** | **0** | **(0%)** | **1** | **(1%)** | **4** | **(1%)** | **0** | **(0%)** | **4** | **(1%)** |
| Pulmonary - respiratory disease/disorder, other | 2 | (1%) | 0 | (0%) | 2 | (1%) | 1 | (1%) | 0 | (0%) | 1 | (1%) | 3 | (1%) | 0 | (0%) | 3 | (1%) |
| Pulmonary - respiratory failure | 1 | (1%) | 0 | (0%) | 1 | (1%) | 0 | (0%) | 0 | (0%) | 0 | (0%) | 1 | (0%) | 0 | (0%) | 1 | (0%) |
| **Serious Renal Disease** | **7** | **(5%)** | **12** | **(8%)** | **19** | **(13%)** | **6** | **(4%)** | **17** | **(11%)** | **23** | **(15%)** | **13** | **(4%)** | **29** | **(10%)** | **42** | **(14%)** |
| Genitourinary - renal system disease/disorder, other | 5 | (3%) | 11 | (7%) | 16 | (11%) | 5 | (3%) | 17 | (11%) | 22 | (15%) | 10 | (3%) | 28 | (9%) | 38 | (13%) |
| Genitourinary - nephrolithiasis | 1 | (1%) | 0 | (0%) | 1 | (1%) | 1 | (1%) | 1 | (1%) | 2 | (1%) | 2 | (1%) | 1 | (0%) | 3 | (1%) |
| Acute renal insufficiency | 1 | (1%) | 1 | (1%) | 2 | (1%) | 0 | (0%) | 0 | (0%) | 0 | (0%) | 1 | (0%) | 1 | (0%) | 2 | (1%) |
