# Supplementary material for: Efficacy and Safety of Three Antiretroviral Regimens for Initial Treatment of HIV-1: A Randomized Clinical Trial in Diverse Multinational Settings
Source: PLoS Med. 2012 Aug 14;9(8):e1001290. doi: 10.1371/journal.pmed.1001290 (PMC3419182; doi:10.1371/journal.pmed.1001290)
Supplement: Table S11 — New serious non-AIDS diagnosis categories compared for EFV+FTC-TDF versus EFV+3TC-ZDV. (DOC) [file pmed.1001290.s016.doc]

**Table S11:** Comparison of Serious Non-AIDS Diagnoses (SNADES) categories by randomized treatment arms: efavirenz plus emtricitabine-tenofovir-DF (EFV+FTC-TDF) versus efavirenz plus lamivudine-zidovudine (EFV+3TC-ZDV)

|  | | **Randomized Group** | |  | |
| --- | --- | --- | --- | --- | --- |
| **SNADES Characteristic** |  | **EFV+**  **3TC-ZDV** | **EFV+**  **FTC-TDF** | **Total** | **P-Value*** |
| Serious Bacterial Infection | yes | 62 (12%) | 61 (12%) | 123 (12%) | 0.924 |
|  | no | 457 (88%) | 465 (88%) | 922 (88%) |  |
|  | | | | | |
| Serious Cardiovascular Disease | yes | 13 (3%) | 7 (1%) | 20 (2%) | 0.182 |
|  | no | 506 (97%) | 519 (99%) | 1,025 (98%) |  |
|  | | | | | |
| Serious Liver Disease | yes | 11 (2%) | 13 (2%) | 24 (2%) | 0.837 |
|  | no | 508 (98%) | 513 (98%) | 1,021 (98%) |  |
|  | | | | | |
| Serious Malignancy | yes | 3 (1%) | 2 (0%) | 5 (0%) | 0.685 |
|  | no | 516 (99%) | 524 (100%) | 1,040 (100%) |  |
|  | | | | | |
| Serious Metabolic Disease | yes | 19 (4%) | 3 (1%) | 22 (2%) | <.001 |
|  | no | 500 (96%) | 523 (99%) | 1,023 (98%) |  |
|  | | | | | |
| Serious Musculosketal Disease | yes | 8 (2%) | 13 (2%) | 21 (2%) | 0.379 |
|  | no | 511 (98%) | 513 (98%) | 1,024 (98%) |  |
|  | | | | | |
| Serious Neuropsychiatric Disease | yes | 47 (9%) | 44 (8%) | 91 (9%) | 0.742 |
|  | no | 472 (91%) | 482 (92%) | 954 (91%) |  |
|  | | | | | |
| Serious Pulmonary Disease | yes | 3 (1%) | 1 (0%) | 4 (0%) | 0.371 |
|  | no | 516 (99%) | 525 (100%) | 1,041 (100%) |  |
|  | | | | | |
| Serious Renal Disease | yes | 19 (4%) | 23 (4%) | 42 (4%) | 0.637 |
|  | no | 500 (96%) | 503 (96%) | 1,003 (96%) |  |
|  | | | | | |
| *Fisher's Exact Test | | | | | |
