# Supplementary material for: Efficacy and Safety of Three Antiretroviral Regimens for Initial Treatment of HIV-1: A Randomized Clinical Trial in Diverse Multinational Settings
Source: PLoS Med. 2012 Aug 14;9(8):e1001290. doi: 10.1371/journal.pmed.1001290 (PMC3419182; doi:10.1371/journal.pmed.1001290)
Supplement: Text S1 — Study protocol. (DOC) [file pmed.1001290.s017.doc]

# A5175

**A PHASE IV, PROSPECTIVE, RANDOMIZED, OPEN-LABEL EVALUATION OF THE EFFICACY OF ONCE-DAILY PROTEASE INHIBITOR- AND ONCE-DAILY NON-NUCLEOSIDE REVERSE TRANSCRIPTASE INHIBITOR-CONTAINING THERAPY COMBINATIONS FOR INITIAL TREATMENT OF HIV-1 INFECTED INDIVIDUALS FROM RESOURCE-LIMITED SETTINGS (PEARLS) TRIAL**

**A Limited Center Trial of the AIDS Clinical Trials Group (ACTG)**

**Sponsored by:**

**The National Institute of Allergy and Infectious Diseases**

**Pharmaceutical Support Provided by:**

**Boehringer Ingelheim Pharmaceuticals, Inc.**

**Bristol-Myers Squibb, Inc.**

**Gilead Sciences, Inc.**

**GlaxoSmithKline**

**Merck & Co., Inc.**

**Optimization of Antiretroviral**

**Therapy (OpART) Committee: Richard Haubrich, M.D., Chair**

Protocol Co-Chairs: Thomas B. Campbell, M.D.

**Timothy Flanigan, M.D.**

**James Hakim, M.D., M.S.C.E., F.R.C.P.**

**Nagalingeswaran Kumarasamy, M.B.B.S., Ph.D.**

**DAIDS Clinical Representative: Karin L. Klingman, M.D.**

**Clinical Trials Specialists: Lara Hosey, M.A., C.C.R.P.**

**Laura E. Moran, M.P.H.**

**FINAL Version 3.0**

**March 2, 2010**

SUBSTUDY

A5185s: Effect of Initial Antiretroviral Treatment on Genital Compartment Virus in Individuals from Resource-Limited Settings

Protocol Co-Chairs: Susan Fiscus, Ph.D.

Suniti Solomon, M.D.

Susan Cu-Uvin, M.D.

A5185s will examine the effects of antiretroviral (ARV) therapy on genital compartment virus in approximately 315 male and female participants enrolled in A5175. The objectives are:

(1) to compare the baseline HIV-1 viral load in the genital secretions from participants infected with HIV subtype B with that from participants infected with subtype C;

(2) to compare baseline genital tract (GT) viral load between men and women;

(3) to measure the proportion of GT viral load below the assay detection limit between baseline and week 48;

(4) to evaluate the durability of the ARV **therapy** response in the GT by comparing GT viral load at week 48 with GT viral load measured at week 96; and

(5) to compare resistance mutations in plasma (as identified by resistance testing performed in A5175) and those found in genital secretions at baseline and study discontinuation (see Appendix VI of this document).

CONTENTS

Page

PARTICIPATING SITES [6](#__RefHeading___Toc253737380)

PROTOCOL TEAM ROSTER [7](#__RefHeading___Toc253737381)

STUDY MANAGEMENT [15](#__RefHeading___Toc253737382)

ACRONYMS [18](#__RefHeading___Toc253737383)

SCHEMA [21](#__RefHeading___Toc253737384)

1.0 HYPOTHESES AND STUDY OBJECTIVES [26](#__RefHeading___Toc253737385)

1.1 Hypotheses [26](#__RefHeading___Toc253737386)

1.2 Primary Objective [26](#__RefHeading___Toc253737387)

1.3 Secondary Objectives [26](#__RefHeading___Toc253737388)

1.4 Programmatic Objectives [27](#__RefHeading___Toc253737389)

1.5 Objectives for A5185s: A Genital Secretions Substudy of A5175. [27](#__RefHeading___Toc253737390)

2.0 INTRODUCTION [27](#__RefHeading___Toc253737391)

2.1 Antiretroviral Agents [27](#__RefHeading___Toc253737392)

2.2 HIV Primary Care [38](#__RefHeading___Toc253737393)

2.3 Rationale [39](#__RefHeading___Toc253737394)

3.0 STUDY DESIGN [42](#__RefHeading___Toc253737395)

4.0 SELECTION AND ENROLLMENT OF PARTICIPANTS [46](#__RefHeading___Toc253737396)

4.1 Step 1: Inclusion Criteria [46](#__RefHeading___Toc253737397)

4.2 Step 1: Exclusion Criteria [49](#__RefHeading___Toc253737398)

4.3 Step 2: Inclusion Criteria [50](#__RefHeading___Toc253737399)

4.4 Step 2: Exclusion Criteria [51](#__RefHeading___Toc253737400)

4.5 Step 3: Inclusion Criteria [51](#__RefHeading___Toc253737401)

4.6 Step 3: Exclusion Criteria [51](#__RefHeading___Toc253737402)

4.7 A5185s Genital Secretions Substudy of A5175: Inclusion and Exclusion Criteria [52](#__RefHeading___Toc253737403)

4.8 Study Enrollment Procedures [52](#__RefHeading___Toc253737404)

4.9 Coenrollment [54](#__RefHeading___Toc253737405)

5.0 STUDY TREATMENT [54](#__RefHeading___Toc253737406)

5.1 Regimens, Administration, and Duration [55](#__RefHeading___Toc253737407)

5.2 Drug Formulation [61](#__RefHeading___Toc253737408)

5.3 Product Supply, Distribution, and Accountability [62](#__RefHeading___Toc253737409)

5.4 Concomitant Medications [62](#__RefHeading___Toc253737410)

5.5 Adherence to Study Treatment [63](#__RefHeading___Toc253737411)

6.0 CLINICAL AND LABORATORY EVALUATIONS [64](#__RefHeading___Toc253737412)

6.1 Schedule of Events (SOE) [64](#__RefHeading___Toc253737413)

6.2 Timing of Evaluations [67](#__RefHeading___Toc253737414)

6.3 Special Instructions and Definitions of Evaluations [69](#__RefHeading___Toc253737415)

6.4 A5185s Genital Secretions Substudy Evaluations [77](#__RefHeading___Toc253737416)

7.0 ANTIRETROVIRAL MANAGEMENT: TOXICITIES, DRUG SUBSTITUTIONS, PREGNANCY, OPPORTUNISTIC INFECTIONS, AND OTHER CONDITIONS [78](#__RefHeading___Toc253737417)

7.1 Antiretroviral Therapy Dosage Reductions [78](#__RefHeading___Toc253737418)

7.2 Grades 1 or 2 Toxicity [79](#__RefHeading___Toc253737419)

7.3 Grade 3 Toxicity [79](#__RefHeading___Toc253737420)

7.4 Grade 4 Toxicity [80](#__RefHeading___Toc253737421)

7.5 Specific Management [80](#__RefHeading___Toc253737422)

7.6 Procedures in the Event of On-Study Pregnancy and Breast-Feeding [89](#__RefHeading___Toc253737423)

7.7 Recommendations for Management of Immune Reconstitution Inflammatory Syndromes [90](#__RefHeading___Toc253737424)

7.8 Management of ATV When Treating Tuberculosis [90](#__RefHeading___Toc253737425)

7.9 Procedures in the Event of Imprisonment or Involuntary Incarceration or Detention [90](#__RefHeading___Toc253737426)

7.10 Treatment of Hepatitis B Virus [91](#__RefHeading___Toc253737427)

8.0 CRITERIA FOR PERMANENT TREATMENT AND STUDY DISCONTINUATION [91](#__RefHeading___Toc253737428)

8.1 Criteria for Permanent Treatment Discontinuation (Off Treatment/On Study) [91](#__RefHeading___Toc253737429)

8.2 Criteria for Permanent Study Discontinuation (Off Treatment/Off Study) [91](#__RefHeading___Toc253737430)

9.0 STATISTICAL CONSIDERATIONS [91](#__RefHeading___Toc253737431)

9.1 General Design Issues [91](#__RefHeading___Toc253737432)

10.0 DATA COLLECTION AND MONITORING AND ADVERSE EVENT REPORTING [98](#__RefHeading___Toc253737433)

10.1 Records to Be Kept [98](#__RefHeading___Toc253737434)

10.2 Role of Data Management [98](#__RefHeading___Toc253737435)

10.3 Clinical Site Monitoring and Record Availability [99](#__RefHeading___Toc253737436)

10.4 Expedited Adverse Event (EAE) Reporting [99](#__RefHeading___Toc253737437)

11.0 STUDY PARTICIPANTS [100](#__RefHeading___Toc253737438)

11.1 Institutional Review Board (IRB) Review and Informed Consent [100](#__RefHeading___Toc253737439)

11.2 Participant Confidentiality [100](#__RefHeading___Toc253737440)

11.3 Study Discontinuation [101](#__RefHeading___Toc253737441)

12.0 PUBLICATION OF RESEARCH FINDINGS [101](#__RefHeading___Toc253737442)

13.0 BIOHAZARD CONTAINMENT [101](#__RefHeading___Toc253737443)

14.0 REFERENCES [102](#__RefHeading___Toc253737444)

APPENDIX I: VIROLOGY STUDY PLAN [106](#__RefHeading___Toc253737445)

APPENDIX II: OPPORTUNISTIC INFECTIONS AND IMMUNE RECONSTITUTION STUDY PLAN [114](#__RefHeading___Toc253737446)

APPENDIX III: MALNUTRITION, MICRONUTRIENT DEFICIENCY, IMMUNOLOGIC FUNCTIONING, AND METABOLIC STUDY PLAN [122](#__RefHeading___Toc253737447)

APPENDIX IV: ADHERENCE AND BEHAVIORAL ISSUES STUDY PLAN [126](#__RefHeading___Toc253737448)

APPENDIX V: CLINICAL PHARMACOLOGY STUDY PLAN [133](#__RefHeading___Toc253737449)

APPENDIX VI: A5185s, EFFECT OF INITIAL ANTIRETROVIRAL TREATMENT ON GENITAL COMPARTMENT VIRUS IN INDIVIDUALS FROM RESOURCE-LIMITED SETTINGS [136](#__RefHeading___Toc253737450)

APPENDIX VII: SAMPLE INFORMED CONSENT FOR A5175

APPENDIX VIII: SAMPLE INFORMED CONSENT FOR WOMEN WHO BECOME PREGNANT WHILE ON A5175

APPENDIX IX: A5185s GENITAL SECRETIONS SUBSTUDY SAMPLE INFORMED CONSENT

# PARTICIPATING SITES

Sites eligible to register to A5175 and A5185s are listed on the A5175 protocol-specific web page (PSWP) <https://www.actgnetwork.org/protocols/protocol_specific_search.aspx>.

# PROTOCOL TEAM ROSTER

Protocol Co-Chairs

Thomas B. Campbell, M.D.

University of Colorado **Denver**

**Mail Stop B-168, Room P15-11001**

**12700 E. 19th Avenue**

**Aurora, CO 80045**

**Phone: (303) 724-4929**

**Fax: (303) 724-4926**

**E-mail:** [**thomas.campbell@ucdenver.edu**](mailto:thomas.campbell@ucdenver.edu)

Timothy Flanigan, M.D.

The Miriam Hospital

164 Summit Avenue

Providence, RI 02906

Phone: (401) 793-7152

Fax: (401) 793-4323

E-mail: [tflanigan@lifespan.org](mailto:pschreiber@lifespan.org)

James Hakim, M.D., M.S.C.E., F.R.C.P.

Department of Medicine

University of Zimbabwe

P.O. Box A178, Avondale

Harare

ZIMBABWE

Phone: 263-479-1631/488-5282

Fax: 263-425-1017

E-mail: [jhakim@mweb.co.zw](mailto:jhakim@mweb.co.zw)

Nagalingeswaran Kumarasamy, M.D.

Centre for AIDS Research &

Education VHS

Tharamani

Chennai - 600113

INDIA

Phone: 91-44-22542929 x222

Fax: 91-44-22542939

E-mail: [kumarasamy@yrgcare.org](mailto:nkumarasamy@eth.net)

DAIDS Clinical Representative

Karin L. Klingman, M.D.

HIV Research Branch

TRP, DAIDS, NIAID, NIH

6700-B Rockledge Drive

MSC 7624, Room 5106

Bethesda, MD 20892-7624

Phone: (301) 435-3772

Fax: (301) 435-9282

E-mail: [kklingman@niaid.nih.gov](mailto:kklingman@niaid.nih.gov)

Clinical Trials Specialists

**Lara Hosey, M.A., C.C.R.P.**

ACTG Operations Center

Social & Scientific Systems, Inc.

8757 Georgia Avenue, 12^th^ Floor

Silver Spring, MD 20910-3714

Phone: (301) 628-**3395**

Fax: (301) 628-3302

E-mail: [lhosey@s-3.com](mailto:lhosey@s-3.com)

**Laura E. Moran, M.P.H.**

ACTG Operations Center

Social & Scientific Systems, Inc.

8757 Georgia Avenue, 12^th^ Floor

Silver Spring MD 20910-3714

Phone: (301) 628-**3373**

Fax: (301) 628-3302

E-mail: [**lmoran@s-3.com**](mailto:lmoran@s-3.com)

Statisticians

Victor De Gruttola, Ph.D.

Statistical & Data Analysis Center

Harvard School of Public Health

Adult Division

Building II, Room 439A

655 Huntington Avenue

Boston, MA 02115-0211

Phone: (617) 432-2820

Fax: (617) 432-1869

E-mail: [victor@sdac.harvard.edu](mailto:victor@sdac.harvard.edu)

Laura Smeaton, M.S.

Statistical & Data Analysis Center

Harvard School of Public Health

Virology Section, Adult Division

FXB Bldg., Room 511

651 Huntington Avenue

Boston, MA 02115-6017

Phone: (617) 432-2525

Fax: (617) 432-3163

E-mail: [smeaton@sdac.harvard.edu](mailto:smeaton@sdac.harvard.edu)

A5185s Statistician

**Abel Tilahun, Ph.D.**

Statistical & Data Analysis Center

**Harvard School of Public Health
Center for Biostatistics in AIDS Research
FXB Bldg., Room 502**

**651 Huntington Avenue
Boston, MA 02115
Phone: (617) 432-2817
Fax: (617) 432 3163**

**E-mail: atilahun@sdac.harvard.edu**

ACTG International Program

**Stephanie Warner, M.A.**

ACTG Operations Center

Social & Scientific Systems Inc.

8757 Georgia Avenue, 12^th^ floor

Silver Spring, MD 20910-3714

Phone: (301) 628-34**76**

Fax: (301) 628-3302

E-mail: [**swarner@s-3.com**](mailto:swarner@s-3.com)

Data Managers

Ann Walawander, M.A.

Frontier Science & Technology Research Foundation

4033 Maple Road

Amherst, NY 14226-1056

Phone: (716) 834-0900, ext. 7290

Fax: (716) 834-8675

E-mail: [walawander.ann@fstrf.org](mailto:walawander.ann@fstrf.org)

Apsara Nair, M.S.

Frontier Science & Technology Research Foundation

4033 Maple Road

Amherst, NY 14226

Phone: (716) 834-0900, ext. 7293

Fax: (716) 834-8432

E-mail: [nair.apsara@fstrf.org](mailto:nair.apsara@fstrf.org)

DAIDS Pharmacist

Ana I. Martinez, R.Ph.

Pharmaceutical Affairs Branch

DAIDS, NIAID, NIH

Room 4115

6700-B Rockledge Drive MSC 7620

Bethesda, MD 20892-7620

Phone: (301) 435-3734

Fax: (301) 402-1506

E-mail: [amartinez@niaid.nih.gov](mailto:amartinez@niaid.nih.gov)

Immunologist

Ronald T. Mitsuyasu, M.D.

UCLA CARE Center

BH-412 CHS

10833 Le Conte Avenue

Los Angeles, CA 90095-1793

Phone: (310) 206-8359

Fax: (310) 206-3311

E-mail: [rmitsuya@mednet.ucla.edu](mailto:rmitsuya@mednet.ucla.edu)

Co-Virologists

Susan H. Eshleman, M.D., Ph.D.

The Johns Hopkins Medical Institution

Ross Building 646

720 Rutland Avenue.

Baltimore, MD 21205

Phone: (410) 614-4734

Fax: (410) 614-3548

E-mail: [seshlem@jhmi.edu](mailto:seshlem@jhmi.edu)

Susan A. Fiscus, Ph.D.

Department of Microbiology & Immunology University of North Carolina

School of Medicine

709 Mary Ellen Jones Building

CB#7290

Chapel Hill, NC 27599-7290

Phone: (919) 966-6872

Fax: (919) 966-9873

E-mail: [fiscussa@med.unc.edu](mailto:fiscussa@med.unc.edu)

Pharmacologists

Adriana Andrade, M.D., M.P.H.

Division of Infectious Disease

Johns Hopkins University

1830 E. Monument Street

Suite 8074

Baltimore, MD 21250

Phone:(410) 614-4036

Fax: (410) 614-0691

E-mail: [aandrade@jhmi.edu](../aandrade@jhmi.edu)

**Courtney V. Fletcher, Pharm.D.**

**College of Pharmacy**

**University of Nebraska Medical Center**

**986000 Nebraska Medical Center**

**Omaha, NE 68198-6000**

**Phone: (402) 559-4333**

**Fax: (402) 449-5060**

**E-mail:** [**cfletcher@unmc.edu**](mailto:cfletcher@unmc.edu)

David W. Haas, M.D.

Infectious Diseases

Vanderbilt University Medical Center

345 24th Avenue North, Suite 105

Nashville, TN 37203

Phone: (615) 467-0154, ext. 107

Fax: (615) 467-0158

E-mail: [david.w.haas@vanderbilt.edu](mailto:david.w.haas@vanderbilt.edu)

Investigators

Farida Amod, MB CHB, FCPath, FCP

Department of Medicine

Nelson R. Mandela School of Medicine

Private Bag 7, Congella, Durban

South Africa

Phone: 27 31 260-4551/260-4637

Fax: 27 31 260-4420

E-mail: [amodf1@ukzn.ac.za](mailto:amodf1@nu.ac.za)

Vladimir Berthaud, M.D.

Infectious Disease

Vanderbilt University Medical Center

345-24th Avenue North, Suite 105

Nashville, TN 37203

Phone: (615) 467-0154

Fax: (615) 467-0154

E-mail: [vberthaud@mmc.edu](mailto:vberthaud@mmc.edu)

Investigators (Cont'd)

Robert C. Bollinger, M.D., M.P.H.

Division of Infectious Diseases

Johns Hopkins Medical School

Ross Research Building, Room 1150

720 Rutland Avenue

Baltimore, MD 21205

Phone: (410) 614-0936

Fax: (410) 614-9775

E-mail: [rcb@mail.jhmi.edu](mailto:rcb@mail.jhmi.edu)

Yvonne Bryson, M.D.

Pediatric Infectious Disease Dept.

UCLA School of Medicine

22-442 MDCC

175217 Le Conte Ave.

Los Angeles, CA 90095-1752

Phone: (310) 825-5235

Fax: (310) 206-5529

E-mail: [ybryson@mednet.ucla.edu](mailto:ybryson@mednet.ucla.edu)

David Celentano, Sc.D., M.H.S.

Department of Epidemiology

Johns Hopkins School of Hygiene and Public Health

615 North Wolfe St., Suite E-7132

Baltimore, MD 21205

Phone: (410) 955-1356

Fax: (410) 955-1836

E-mail: [dcelenta@jhsph.edu](mailto:dcelenta@jhsph.edu)

David Chilongozi, C.O., M.P.H.

UNC HIVNET c/o Staff Project

Private Bag A-104

Amina House, 3rd Floor

Chilambula Road

Lilongwe

MALAWI

Phone: 011-265-755-056

Fax: 011-265-775-954

E-mail: [dchilongozi@malawi.net](mailto:dchilongozi@malawi.net)

Myron Cohen, M.D.

University of North Carolina, Chapel Hill

547 Burnett Womack, CB 7030

Chapel Hill, NC 27514

Phone: (919) 966-2536

Fax: (919) 966-6714

E-mail: [mscohen@med.unc.edu](mailto:mscohen@med.unc.edu)

Investigators (Cont'd)

Ann C. Collier, M.D.

University of Washington

Harborview Medical Center

325 9th Avenue, Box 359929

Seattle, WA 98104

Phone: (206) 731-3293

Fax: (206) 731-3483

E-mail: [acollier@u.washington.edu](mailto:acollier@u.washington.edu)

Judith Silverstein Currier, M.D., M.Sc.

University of California, Los Angeles

UCLA CARE Center

9911 W Pico Boulevard

Suite 980

Los Angeles, CA 90035

Phone: (310) 557-1891

Fax: (310) 557-1899

Email: [jscurrier@mednet.ucla.edu](mailto:jscurrier@mednet.ucla.edu)

Susan Cu-Uvin, M.D.

The Miriam Hospital, Brown University

The Immunology Center

164 Summit Avenue

Providence, RI 02906

Phone: (401) 793-4775

Fax: (401) 793-4779

E-mail: [scu-uvin@lifespan.org](../scu-uvin@lifespan.org)

Joseph Eron, M.D.

Division of Infectious Diseases

Department of Medicine

University of North Carolina

Bioinformatics Building, 2^nd^ Floor

130 Mason Farm Road

Chapel Hill, NC 27514

Phone: (919) 966-2536

Fax: (919) 966-6714

E-mail: [jeron@med.unc.edu](mailto:jeron@med.unc.edu)

Charles Flexner, M.D.

Johns Hopkins University Hospital

Osler 524

600 North Wolfe Street

Baltimore, MD 21287-5554

Phone:(410) 955-9712

Fax: (410) 955-9708

E-mail: [flex@jhmi.edu](../flex@jhmi.edu)

Investigators (Cont'd)

Joel E. Gallant, M.D., M.P.H.

Division of Infectious Diseases

Johns Hopkins AIDS Service

Johns Hopkins University School
 of Medicine

1830 E. Monument Street

Suite 443

Baltimore, MD 21287

Phone: (410) 955-7473

Fax: (410) 614-8099

E-mail: [jgallant@jhmi.edu](mailto:jgallant@jhmi.edu)

Beatriz Grinsztejn, M.D., Ph.D.

Evandro Chagas Clinical Research Institute

Oswaldo Cruz Foundation-FIOCRUZ

Avenida Brasil 4365, Manguinhos

BRAZIL

Cep: 21045-900

Phone/Fax: 55 21 25644933

Cell phone: 55 21 96357679

Email: [gbeatriz@unisys.com.br](mailto:gbeatriz@unisys.com.br)

Roy M. Gulick, M.D., M.P.H.

The Cornell Clinical Trials Unit

Box 566

525 East 68th Street, F2400

New York, NY 10021

Phone: (212) 746-4177

Fax: (212) 746-8852

Email: [rgulick@med.cornell.edu](mailto:rgulick@med.cornell.edu)

Scott M. Hammer, M.D.

Division of Infectious Diseases

Columbia Presbyterian Medical Center

630 West 168th Street

PH 8 West, Room 876, Box 82

New York, NY 10032

Phone: (212) 305-7185

Fax: (212) 305-7290

E-mail: [smh48@columbia.edu](mailto:smh48@columbia.edu)

Irving Hoffman, P.A., M.P.H.

University of North Carolina School of Medicine

Bioinformatics Building, 2^nd^ Floor

130 Mason Farm Road

Chapel Hill, NC 27514

Phone: (919) 966-6324

Fax: (919) 966-8536

E-mail: [hoffmani@med.unc.edu](mailto:hoffmani@med.unc.edu)

Investigators (Cont'd)

Mina Hosseinipour, M.D.

**UNC Project - Kamuza Central Hospital**

**Tidziwe Centre**

**100 Mzimba Road**

**Private Bag A-104**

**Lilongwe**

**Malawi**

**Phone: 011 265 175 5056**

**Fax: 011 265 175 5954**

E-mail: [minach@med.unc.edu](mailto:minach@med.unc.edu)

Peter Kazembe, MBCHB FRCP(C)

Lilongwe Central Hospital

Private Bag 306

Capital City

Lilongwe

MALAWI

Phone: 011-265-855-447

Fax: 011-265-721-018

E-mail : [pnkazembe@malawi.net](mailto:pnkazembe@malawi.net)

Johnstone Kumwenda, MBBS, MMED

Department of Medicine

College of Medicine

Private Bag 360

Chichiri, Blantyre

MALAWI

Phone: 011-265-841-875

Fax: 011-265-674-700

E-mail: [jkumwenda@jhu.medcol.mw](mailto:jkumwenda@jhu.medcol.nw)

Newton Kumwenda, M.P.H., Ph.D.

Johns Hopkins Project

P.O. Box 1131

Blantyre

MALAWI

Phone: 011-265-631-527

Fax: 011-265-631-527

E-mail: [jhopkins@sdnp.org.mw](mailto:jhopkins@sdnp.org.mw)

Umesh Gangaram Lalloo, MBChB, FCP, DOH, MD, FRCP

Family HIV Clinic

Nelson R. Mandela School of Medicine

Private Bag 7, Congella, 4013, Durban South Africa

Phone: 27 31 260-4668/4637

Fax: 27 31 260 4420

E-mail: [lalloo@ukzn.ac.za](mailto:lalloo@nu.ac.za)

Investigators (Cont'd)

Javier R. Lama, M.D.

Asociación Civil Impacta Salud y Educación

HIV Vaccine Trial Unit

Lima

PERU

Phone: (51) 124 23072

Fax: (51) 124 22467

E-mail: [jrlama@impactaperu.org](mailto:jrlama@impactaperu.org)

Alberto La Rosa, M.D.

Asociación Civil Impacta Salud y Educación

Av. Grimaldo del Solar 805

Lima

PERU

Phone: (51) 124 23072 ext 122

Fax: (51) 124 22467

E-mail: [alarosa@impactaperu.org](mailto:alarosa@impactaperu.org)

Jody Lawrence, M.D.

University of California, San Francisco

Adult AIDS Clinical Trials Unit

3180 18th Street, Suite 305

San Francisco, CA 94110-2042

Phone: (415) 514-0550 ext. 402

Fax: (415) 476-6736

E-mail: [jlawrence@php.ucsf.edu](mailto:jlawrence@php.ucsf.edu)

Chiedza Maponga, Pharm.D.

DaTIS - Medical University of Zimbabwe

P.O. Box A178 Avondale

Harare

Zimbabwe

Phone: 263-4-791520

E-mail: [cmaponga@med-sch.uz.zw](mailto:cmaponga@med-sch.uz.zw)

Francis Martinson, M.D.

UNC Project

Private Bag A-104

Lilongwe

MALAWI

Phone: 011-265-755-056

Fax: 011-265-755-954

E-mail: [fmartinson@malawi.net](mailto:fmartinson@malawi.net)

Investigators (Cont'd)

Karin Nielsen, M.D.

UCLA School of Medicine

10833 Le Conte Ave.

Rm 22-442 MDCC, Box 951752

Los Angeles, CA 90095

Phone: (310) 206-6369

Fax: (310) 825-9175

E-mail: [knielsen@mednet.ucla.edu](mailto:knielsen@mednet.ucla.edu)

Jean William Pape, M.D.

Cornell-GHESKIO

Institut Nacional de laboratoire et de Recherches

33 Blvd. Harry Truman

Cite de l'Exposition

Port-au-Prince

HAITI

Phone: 509-22-0031

Fax: 509-23-9044

E-mail: [jwpape@gheskio.org](mailto:jwpape@gheskio.org)

Richard B. Pendame M.D., M.P.H.

Box 30377, Lilongwe 3

MALAWI

Phone: (265) 789 543

Fax: (265) 789 431

E-mail: [shp@clcom.net](mailto:shp@clcom.net) or

[rpendame@voila.net](mailto:rpendame@voila.net)

Steve Safren, Ph.D.

Department of Psychiatry WACC 815 Massachusetts General Hospital

15 Parkman Street

Boston, MA 02114

Phone: (617) 724-0817 or (617) 927-6057

Fax: (617) 726-7541

E-mail: [ssafren@partners.org](mailto:ssafren@partners.org)

Raul Salazar, M.D., FACP

Internal Medicine Service

Infectology Unit, HIV/AIDS Program

Hospital Guillermo Almenara

Av. Grau 800, La Victoria

Lima

PERU

Phone: (51 1) 4756378

Fax: (51 1) 4730956

E-mail: [salazarraul@usa.net](mailto:salazarraul@usa.net)

Investigators (Cont'd)

Jorge Sanchez, M.D.

Asociación Civil Impacta Salud y Educación

HIV Vaccine Trial Unit

Lima

PERU

Phone: (51) 124 23072

Fax: (51) 124 22467

E-mail: [jsanchez@impactaperu.org](mailto:jsanchez@impactaperu.org)

Ian M. Sanne, M.D.

University of Witwatersrand

Infectious Diseases Syndicate

Clinical Trial Unit

17 Eton Road

Parktown, Johannesburg, 2193

South Africa

Office Phone: **27 11 7172810**

Direct Phone: **27 11 7172813**

Fax: (271)148-25554

E-mail: [imsanne@yebo.co.za](mailto:imsanne@yebo.co.za)

Investigators (Cont'd)

Breno Riegel Santos, M.D.

Servico de Insectología

Hospital Nossa Senhora da Conceicao-GHC

Av. Francisco Trein, 596

91350-200 Porto Alegre, RS

Brazil

Phone: 55-51-3341-5316

Fax: 55-51-3343-2386

Email: [breno@ghc.com.br](mailto:breno@ghc.com.br)

Robert T. Schooley, M.D.

University of California, San Diego

Stein Research Bldg., Room 401

Mail Code 0711

9500 Gilman Drive

La Jolla, CA 92023-0711

Phone: 858-822-0216

Fax: 858-822-5362

E-mail: [rschooley@ucsd.edu](../rschooley@ucsd.edu)

Investigators (Cont'd)

Patrice Severe, M.D.

Internal Medicine, Infectious Diseases

Centres Gheskio/Institut de Laboratoires et de Recherches

# 33 Boulevard Harry Truman

Port-au-Prince

HAITI

Phone: (509) 222-2241

Fax: (509) 223-9044

E-mail: [patsevere@gheskio.org](mailto:patsevere@gheskio.org)

Thira Sirisanthana, M.D.

Research Institute for Health Sciences

Chiang Mai University

Chiang Mai 50200

Thailand

Phone: 665 394 5055

Fax: 6653-217144 or 945481 or 277059

E-mail: [ssirisan@mail.med.cmu.ac.th](mailto:ssirisan@mail.med.cmu.ac.th)

Suniti Solomon, M.D.

YRG Centre for AIDS Research

and Education

1, Raman St. T Nagar

Chennai - 600017

INDIA

Phone: 011-91-44-826-4242

Fax: 011-91-44-825-6900

E-mail: [yrgcar@vsnl.com](mailto:yrgcar@vsnl.com)

Khuanchai Supparatpinyo, M.D.

Department of Medicine

Chiang Mai University

Chiang Mai 50200

Thailand

Phone: 66-53-946457

Fax: 66-53-895023

E-mail: [khuanchai@idthai.org](mailto:khuanchai@idthai.org)

Taha Taha, M.D.

Johns Hopkins University

School of Hygiene & Public Health

615 North Wolfe Street, Room E-6011

Baltimore, MD 21205

Phone: (410) 614-5255

Fax: (410) 955-1836

E-mail: [ttaha@jhsph.edu](mailto:ttaha@jhsph.edu)

Investigators (Cont'd)

Srikanth Tripathy, M.D.

National AIDS Research Institute

73 G Block

MIDC, Bhosari

Pune 411026

INDIA

Phone: 91-20-268-78742

Fax: 91-20-712-1071

E-mail: [**stripathy@nariindia.org**](mailto:stripathy@nariindia.org)

Charles van der Horst, M.D.

Department of Medicine

Division of Infectious Diseases

University of North Carolina School of Medicine

547 Burnett-Womack Bldg., CB7030

Chapel Hill, NC 27599-7030

Phone: (919) 966-2536

Fax: (919) 966-6714

E-mail: [cvdh@med.unc.edu](mailto:cvdh@med.unc.edu)

Christine Wanke, M.D.

Tufts University School of Medicine

150 Harrison Ave

Boston, MA 02111

Phone: (617) 636-0921

Fax: (617) 636-3810

E-mail: [christine.wanke@tufts.edu](mailto:christinewanke@tufts.edu)

Field Representatives

Cheryl J. Marcus, R.N., B.S.N.

University of North Carolina, Chapel Hill

Bioinformatics Building, 2^nd^ Floor

130 Mason Farm Road

Chapel Hill, NC 27514

Phone: (919) 843-8761

Fax: (919) 966-8928

E-mail: [cjm@med.unc.edu](mailto:cjm@med.unc.edu)

Beverly Putnam, R.N., M.S.N

**University of Colorado Denver**

**Academic Office 1, Room L15-7519**

**Mail Stop 8205**

**12631 E. 17^th^ Avenue**

**Aurora, CO 80045**

**Phone: (303) 724-0762**

**Fax: (303) 724-0802**

**E-mail:** [**Beverly.Putnam@ucdenver.edu**](mailto:Beverly.Putnam@ucdenver.edu)

**CSS** Representative

**Martha Mensah-King**

**912 Ringwood Drive**

**Strathven**

**Harare**

**ZIMBABWE**

Phone: **(263) 470-0832**

Fax: **(263) 474-0614**

E-mail: [**m_tholanah@yahoo.com**](mailto:m_tholanah@yahoo.com)

Industry Representatives

**Lauren Petrella**

**Boehringer Ingelheim Pharmaceuticals, Inc.**

**900 Ridgebury Road**

**P.O. Box 368**

**Ridgefield, CT 06877**

**Phone: (203) 778-7320**

**E-mail:** [**cconner@rdg.boehringer-ingelheim.com**](mailto:cconner@rdg.boehringer-ingelheim.com)

**Jonathan Uy, M.D.**

**Medical Affairs**

**Bristol-Myers Squibb
777 Scudders Mill Road
Plainsboro, NJ 08536
Phone: (609) 897-4787**

**Fax: (609) 897-6068**

**E-mail:** [**jonathan.uy@bms.com**](mailto:jonathan.uy@bms.com)

Renard S. Descallar

Gilead Sciences Inc.

333 Lakeside Drive

Foster City, CA 94404

Phone: (650) 522-5052

E-mail: [renard.descallar@gilead.com](mailto:renard.descallar@gilead.com)

James F. Rooney, M.D.

Clinical Research

Gilead Sciences, Inc.

333 Lakeside Drive

Foser City, CA 94404

Phone: (650) 522-5708

Fax: (650) 522-5854

E-mail: [jim_rooney@gilead.com](mailto:jim_rooney@gilead.com)

Industry Representatives (Cont'd)

**Gary E. Pakes, Pharm.D.**

**Infectious Diseases Medicine Development Center**

**GlaxoSmithKline**

**5 Moore Drive**

**Mailstop 17.1363A.1B**

**Research Triangle Park, NC 27709**

**Phone: (919) 483-8242**

**Fax: (704) 625-9200**

**E-mail:** [**gary.e.pakes@gsk.com**](mailto:gary.e.pakes@gsk.com)

Keith A. Pappa, Pharm.D.

GlaxoSmithKline

**5** Moore Drive

P.O. Box 13398

Research Triangle Park, NC 27709-3398

Phone: (919) 483-3533

Fax: (919) 315-6029

E-mail: [keith.a.pappa@gsk.com](../keith.a.pappa@gsk.com)

**Wendy Snowden**

**Infectious Diseases Medicine Development Center**

**GlaxoSmithKline**

**Building 38-2/30, Greenford Road**

**Greenford, Middlesex UB6 0HE**

**United Kingdom**

**Phone: (440) 208-9664139**

**E-mail:** [**wendy.x.snowden@gsk.com**](mailto:wendy.x.snowden@gsk.com)

Laboratory Data Coordinator

**Kenneth Braun**

Frontier Science & Technology Research

Foundation, Inc.

4033 Maple Road

Amherst, NY 14226-1056

Phone: (716) 834-0900 ext. 7227

Fax: (716) 833-**8432**

**E-mail:** [**braun@fstrf.org**](mailto:braun@fstrf.org)

Laboratory Technologist

David L. Shugarts, M.A.

<University> of <Colorado> **<<Denver>>**

**Mail Stop B-168, Room P15-11006**

**<<12700 E. 19th Avenue>>**

**<<Aurora>, <CO> <80045>>**

Phone: **(303) 724-2981**

Fax: **(303) 724-4926**

E-mail: [david.shugarts@uchsc.edu](mailto:david.shugarts@uchsc.edu)

# STUDY MANAGEMENT

All questions concerning this protocol should be sent via e-mail to the A5175 Clinical Management Committee (CMC) at [actg.cmca5175@fstrf.org](mailto:actg.cmca5175@fstrf.org). Include “A5175” in the subject line and the name of your site in the message area. The appropriate member of the CMC will respond via e-mail with a "cc" to the A5175 CMC. A response should generally be received within 24 hours (Monday-Friday)**,** excluding major U.S. holidays.

NOTE: The A5175 CMC consists of the protocol Co-Chairs, Statisticians, DAIDS Clinical Representative and Pharmacist, Data Manager, Clinical Trials Specialists, and other protocol team members identified by the A5175 CMC.

Protocol E-mail Group

Sites registering to A5175 must contact the Computer Support Group at the Data Management Center (DMC) **via e-mail** to have relevant site staff added to the [actg.protA5175@fstrf.org](mailto:actg.protA5175@fstrf.org) e-mail group as soon as possible. Their inclusion will ensure that sites receive important information about the conduct and implementation of A5175.

- **Send an e-mail message to** [**actg.user.support@fstrf.org**](mailto:actg.user.support@fstrf.org)**.**

Clinical Management

For questions concerning entry criteria, toxicity management, concomitant medications, and coenrollment, contact the protocol A5175 CMC.

- Send an e-mail message to [actg.cmca5175@fstrf.org](mailto:actg.cmca5175@fstrf.org).
- Include the protocol number, patient identification number (PID), and a brief relevant history.

**CRSs** must contact the A5175 CMC to request permission to make Step 1 changes in antiretroviral drug regimens and to register participants to Step 2. Sites are encouraged to contact the A5175 CMC with eligibility or any other questions.

Laboratory

For questions specifically related to immunologic, virologic, or pharmacologic laboratory tests, contact the protocol Immunologist, Virologists, or Pharmacologists, respectively.

- Send an e-mail message to [actg.cmca5175@fstrf.org](mailto:actg.cmca5175@fstrf.org), ATTN:

Immunologist: Ronald Mistsuyasu, M.D.

Co-Virologists: Susan H. Eshleman, M.D., Ph.D., Susan A. Fiscus, Ph.D., or

Pharmacologists: Charles Flexner, M.D., David Haas M.D.

Data Management

For nonclinical questions about **transfers,** inclusion/exclusion criteria, case report forms (CRFs), the CRF schedule of events, randomization, registration, transfers, delinquencies, and other data management issues, contact the Data Managers.

- **For transfers, reference the “Patient Transfer from Site to Site” SOP 119, and contact Ann Walawander, M.A. or Apsara Nair, M.S., directly.**
- **For other questions, s**end an e-mail message to [actg.cmca5175@fstrf.org](mailto:actg.cmca5175@fstrf.org) (ATTN: Ann Walawander, M.A. or Apsara Nair, M.S.).
- Include the protocol number, patient identifier (number) (PID), and a detailed question.

Randomization/**Participant Registration**

For randomization***/*participant registration** questions or problems and **study identification** **number** SID lists.

- **Send an e-mail message to** [**sdac.random.desk@fstrf.org**](mailto:sdac.random.desk@fstrf.org) **or call the Statistical and Data Analysis Center (SDAC)/DMC Randomization Desk at (716) 898-7301.**

Computer and Screen Problems

Contact the SDAC/DMC programmers:

- Send an e-mail message to [actg.user.support@fstrf.org](mailto:actg.user.support@fstrf.org) or call (716) 834-0900 x7302.

Protocol Document Questions

For questions concerning the protocol document, contact the Clinical Trials Specialists.

- Send an e-mail message to [actg.cmca5175@fstrf.org](mailto:actg.cmca5175@fstrf.org) (ATTN: **Lara Hosey, M.A., C.C.R.P. and Laura Moran, M.P.H**.).

Copies of the Protocol

To request hard copies of the protocol

- Send a message to [ACTGOpsCenter@s-3.com](mailto:ACTGOpsCenter@s-3.com) (ATTN: Diane Delgado).
- Electronic copies can be downloaded from the **ACTG Web site** **(**[**https://www.actgnetwork.org**](https://www.actgnetwork.org/)**).**

Product Package Inserts or Investigator Brochures

To request copies of product package inserts or investigator brochures contact the DAIDS Regulatory Compliance Center (RCC) Safety Information Center at [RIC@tech-res.com](mailto:RIC@tech-res.com) or call (301) 897-1708.

Protocol Registration

**For protocol registration questions:**

- **Send an e-mail message to** [**Protocol@tech-res.com**](mailto:Protocol@tech-res.com) **or call (301) 897-1707.**

Study Drugs

For questions or problems regarding study drugs, doses, supplies, records, and returns:

- Call Ana I. Martinez, R. Ph., Protocol Pharmacist, at 301-496-8213 or
- E-mail [amartinez@niaid.nih.gov](mailto:amartinez@niaid.nih.gov).

Study Drug Orders

- Call the Clinical Research Products Management Center at (301) 294-0741.

Expedited Adverse Event (EAE) Questions

- Contact the DAIDS RCC Safety Office at [RCCSafetyOffice@tech-res.com](mailto:RCCSafetyOffice@tech-res.com) or call 1-800-537-9979 or 301-897-1709; or fax 1-800-275-7619 or 301-897-1710.

Phone Calls

- All telephone communications must be documented by e-mail to [actg.cmca5175@fstrf.org](mailto:actg.cmca5175@fstrf.org). This will be the site’s responsibility.

Protocol-Specific Web Page (PSWP)

Additional information concerning A5175 and A5185s study management can be found on the A5175 PSWP <https://www.actgnetwork.org/protocols/protocol_specific_search.aspx>.

# ACRONYMS

ABC abacavir

##### ACTG AIDS Clinical Trials Group

AE adverse event

##### AIDS acquired immunodeficiency syndrome

ALT alanine aminotransferase

ANC absolute neutrophil count

**ART antiretroviral therapy**

ARV antiretroviral

AST aspartate aminotransferase

ATV atazanavir

AUC area under the curve

BID twice daily

BMD bone mineral density

**BMI body mass index**

CBV lamivudine + zidovudine (Combivir®)

**Cr**Cl **creatinine** clearance

CI confidence interval

CK creatine kinase

CLIA Clinical Laboratory Improvement Amendments

C_max_ maximum concentration

C_min_ minimum concentration

##### CMC Clinical Management Committee

CNS central nervous system

CRF case report form

**CRS Clinical Research Site**

CRPMC Clinical Research Products Management Center for the ACTG

**CSS** **Community Scientific Subcommittee**

d4T stavudine immediate release

DAIDS Division of Acquired Immune Deficiency Syndrome (U.S.)

ddI EC enteric-coated didanosine

DEXA dual-energy x-ray absorptiometry

DMC Data Management Center for the ACTG

DSMB Data and Safety Monitoring Board (NIAID)

EAE expedited adverse event

EFV efavirenz

EKG electrocardiogram

ELISA enzyme-linked immunosorbent assay

EMEA European Agency for the Evaluation of Medicinal Products

FDA Food and Drug Administration (U.S.)

FDC fixed-dose combination

FTC emtricitabine

FTC/TDF emtricitabine + tenofovir disoproxil fumarate (fixed dose) [Truvada®]

GCP good clinical practice

GT genital tract

HAART highly-active antiretroviral therapy

HBcAb hepatitis B core antibody

HBsAg hepatitis B surface antigen

HBV hepatitis B virus

HCV hepatitis C virus

HCV Ab hepatitis C antibody

HDL high-density lipoprotein

HCG human chorionic gonadotropin

HIV-1 human immunodeficiency virus type 1

HPTN HIV Prevention Trials Network

HSR hypersensitivity reaction

INH isoniazid

IQA Immunology Quality Assurance

IRB institutional review board

IRD immune restoration disease

IRIS immune reconstitution inflammatory syndrome

IRS immune reconstitution syndrome

ITT intent-to-treat

IUD intrauterine device

3TC lamivudine

3TC/ZDV lamivudine + zidovudine

LDL low-density lipoprotein

LFT liver function test

LPC lab processing chart

LPV lopinavir

OI opportunistic infection

MAC Mycobacterium avium complex

MOPS Manual of Operations

MTB Mycobacterium tuberculosis

MTCT mother-to-child-transmission

NDA new drug application

NFV nelfinavir

NNRTI nonnucleoside reverse transcriptase inhibitor

NRTI nucleoside analogue reverse transcriptase inhibitor

NtRTI nucleotide reverse transcriptase inhibitor

NVP nevirapine

OHRP Office for Human Research Protections

OpART ACTG Optimization of Antiretroviral Therapy Committee

PBMC peripheral blood mononuclear cells

PCP *Pneumocystis carinii* pneumonia

PDE5 phosphodiesterase type 5

PI protease inhibitor

PID patient identifier (number)

PK pharmacokinetic(s)

PML progressive multifocal leukoencephalopathy

PO by mouth

PPD purified protein derivative

PRN as needed

PSWP protocol-specific web page

PTT partial thromboplastin time (coagulation assay)

QD once every day

QHS at every bedtime

RCC Regulatory Compliance Center (DAIDS)

**RLS resource-limited setting(s)**

RT-PCR reverse transcriptase-polymerase chain reaction

RTV ritonavir

Rx therapy/treatment

SAE serious adverse event

SD standard deviation

SGOT aspartate aminotransferase

SGPT alanine aminotransferase

SID study ID

SJS Stevens-Johnson syndrome

SOE schedule of events

STD sexually transmitted disease

TB tuberculosis

TDF tenofovir disoproxil fumarate

TEN toxic epidermal necrolysis

TID three times per day

T_max_ time at which the maximum drug concentration occurs

UB upper bound

UKNEQAS United Kingdom National External Quality Assurance Service

ULN upper limit of normal

**UNAIDS United Nations Joint Programme on HIV/AIDS**

USDHHS United States Department of Health and Human Services

VID visit identification number (i.e., study week)

VQA Virology Quality Assurance

W week

**WHO World Health Organization**

ZDV zidovudine (AZT)

# SCHEMA

A PHASE IV, PROSPECTIVE, RANDOMIZED, OPEN-LABEL EVALUATION OF THE EFFICACY OF ONCE-DAILY PROTEASE INHIBITOR- AND ONCE-DAILY NON-NUCLEOSIDE REVERSE TRANSCRIPTASE INHIBITOR-CONTAINING THERAPY COMBINATIONS FOR INITIAL TREATMENT OF HIV-1 INFECTED INDIVIDUALS FROM RESOURCE-LIMITED SETTINGS (PEARLS) TRIAL

DESIGN: A5175 is a phase IV, randomized, open-label, three-arm, antiviral efficacy trial designed to evaluate three antiretroviral (ARV) regimens for treatment-naïve HIV-infected participants: one regimen containing two nucleoside reverse transcriptase inhibitors (NRTIs) + a protease inhibitor (PI) and two regimens each containing two NRTIs + a non-nucleoside reverse transcriptase inhibitor (NNRTI).

The primary treatment comparisons in A5175 investigate: 1) if a once-daily **(QD)** regimen containing two NRTIs + PI (Arm 1B) is not inferior to a twice-daily **(BID)** regimen containing two NRTIs + NNRTI (Arm 1A), and 2) if a **QD** regimen that contains two NRTIs + NNRTI (Arm 1C) is not inferior to **BID** regimen that also contains two NRTIs + NNRTI (Arm 1A). Both evaluations will be based on the primary study endpoint, time to treatment failure of the initial regimen (Step 1).

Time to treatment failure will be defined as the time from randomization to the first occurrence of any of the following: 1) death due to any cause; 2) disease progression - defined as a new or recurrent AIDS-defining opportunistic infection or malignancy occurring at least 12 weeks following randomization (see the A5175 and A5185s Manual of Operations [MOPS]); or 3) virologic failure defined as two successive measurements of plasma HIV-1 RNA ≥1000 copies/mL, with the first measurement at the week 16 visit or later. Participants who experience virologic failure on the **initial** Step 1 regimen may receive a second ARV regimen in Step 2.

Study enrollment at both **U.S. and non-U.S. Clinical Research Sites (CRSs)** will be conducted in two stages. During Stage I, all sites will each enroll 10 participants, and then enrollment will be closed at the individual site. During this period of closure, each **site** will undergo review to identify and correct any deficiencies in good clinical practice (GCP) and assess laboratory quality assurance. The **sites** that meet the review standards will then become eligible to enroll participants in Stage II. (Note: All **sites** successfully completed **this** review as of 5/6/2006). Stage II will commence for **U.S. and non-U.S. CRSs** when six **non-U.S. CRSs** have successfully met the review standards of Stage I (see section 4.6). (Note: Stage II commenced on 12/19/2005.)

**On May 6, 2008, the Data and Safety Monitoring Board (DSMB) met to perform a regularly scheduled review of A5175/PEARLS safety and efficacy data. During that review, the DSMB found conclusive evidence that the regimen consisting of emtricitabine (FTC) + atazanavir (ATV) + didanosine-enteric coated (ddI-EC) (Arm 1B) was inferior to lamivudine (3TC)/zidovudine (ZDV) and efavirenz (EFV) (Arm 1A, the control arm) regarding the rate of primary study events. In response to the DSMB recommendation, Arm 1B was closed and Step 3 was created; all participants in Arm 1B, regardless of what ARV regimen they were receiving, were enrolled into Step 3 and given options for regimen change as described below under Step 3: DSMB-Recommended Regimen Change for Participants Enrolled in Arm 1B.**

DURATION: All participants will be followed until either 30% of participants have reached the primary endpoint or for a total study duration of 2.5 years after randomization of the first participant, whichever occurs later in time. **On November 3, 2009, the DSMB reviewed the observed rates of primary endpoints and decided that continuation of the study beyond May 2010 would not greatly improve the ability of the study to answer the primary research question regarding the comparison of Arms 1A to 1C. At the recommendation of the DSMB, the study will close to follow-up on May 31, 2010.**

SAMPLE SIZE: 1520 participants will be enrolled to ensure at least 1368 evaluable participants; 456 evaluable participants per treatment arm (see section 9.3).

POPULATION: Men and women ≥18 years of age with documentation of HIV-1 infection, CD4+ cell counts <300 cells/mm^3^ within 90 days prior to study entry and naïve to ARV treatment.

STRATIFICATION: By country and by screening plasma HIV-1 RNA (<100,000 copies/mL versus ≥100,000 copies/mL).

REGIMEN: STEP 1: Initial Regimen

At entry, participants will be randomized (1:1:1) to one of the following three treatment arms:

Arm 1A: 3TC/ZDV 150 mg/300 mg PO BID + EFV 600 mg PO QHS

Arm 1B: FTC 200 mg PO QD + ATV 400 mg PO QD + ddI-EC 400 mg PO QD for participants who weigh ≥60 kg or ddI-EC 250 mg PO QD for participants who weigh <60 kg

Arm 1C: FTC/tenofovir (TDF) 200 mg/300 mg PO QHS + EFV 600 mg PO QHS

STEP 2: Second Regimen for Management of Presumed and/or Documented Antiretroviral Drug Resistance

The purpose of Step 2 is to provide a second treatment regimen for management of presumed and/or documented loss of potency due to the development of resistance **to** the **ARV** agents used in Step 1. Participants who meet the criteria for a regimen switch on Step 1 (see sections 3.0 and 4.3) will be offered a switch to any viable combination of three or more of the A5175-provided study drugs used in Step 1 and/or non-study drugs not available through A5175, at the discretion of the site investigator.

Arm 2A: Participants for whom Arm 1A treatment fails will switch to

Two NRTIs + PI(s).

Arm 2B: Participants for whom Arm 1B treatment fails will switch to

Two NRTIs + EFV.

Arm 2C: Participants for whom Arm 1C treatment fails will switch to

Two NRTIs + PI(s).

NOTE A: A minimum of two NRTIs is required in Step 2. Since Step 2 treatment options are limited and 3TC or FTC may provide continued benefit despite the presence of high-level resistance, 3TC or FTC will be allowed as a third NRTI in Step 2 regimens when potentially viable two-NRTI combinations are not available, at the discretion of the site investigator.

NOTE B: All A5175-related ARV resistance testing will be performed retrospectively. Therefore, results will not be available to guide the selection of Step 2 regimens. Results obtained outside of A5175 may, however, be used to guide the selection of Step 2 ARV drugs.

**Step 3: DSMB-Recommended Regimen Change for Participants Enrolled in Arm 1B**

**The DSMB review of May 6, 2008, concluded that FTC + ATV + ddI-EC should not be recommended for initial therapy in ARV-naïve individuals and that this study treatment should be stopped. All participants followed on Arm 1B, regardless of what ARV regimen they were receiving, were asked to return to the site as soon as possible.**

**If participants chose to remain in the study and continue to receive ARV therapy through the study, the study provided and will continue to provide co-formulated lamivudine/zidovudine (3TC/ZDV), co-formulated FTC/tenofovir (TDF), efavirenz (EFV), and ATV from which to formulate an ARV regimen. If ATV is used together with TDF, ritonavir (RTV) must be obtained from outside the study and used to boost ATV. The regimen of FTC + ATV + ddI-EC will no longer be provided by the study. If participants chose to remain on this regimen, they will need to acquire these drugs from outside the study.**

**The following summarizes recommended study-provided options for participants on Arm 1B*:**

| **Viral Load** | **ARV Regimen** | **Recommended**  **Study-Provided Options** |
| --- | --- | --- |
| **<400 copies/mL** | **FTC (or 3TC) + ATV + ddI-EC** | **3TC/ZDV + EFV**  **OR**  **FTC/TDF + EFV and enroll to Step 3.** |
|  | **Any other regimen** | **Participant may continue the current regimen and enroll to Step 3.**  **Confer with the A5175 Clinical Management Committee (CMC) if a regimen change is felt to be needed.** |
| **Confirmed**  **400–999 copies/mL** | **FTC (or 3TC) + ATV + ddI-EC** | **Confer with the A5175 CMC for selection of a Step 3 regimen.** |
|  | **Any other regimen** | **Confer with the A5175 CMC and enroll to Step 3.** |
| **Confirmed**  **>1000 copies/mL** | **FTC (or 3TC) + ATV + ddI-EC** | **Enter Step 2 after discussion with the A5175 CMC.** |
|  | **Any other regimen** | **Enter Step 2 after discussion with the A5175 CMC.** |

***If a participant was receiving the regimen of FTC + ATV + ddI-EC in an arm other than Arm 1B, the A5175 CMC was to be consulted for management recommendations.**

SUBSTUDY: A5185s, a substudy of A5175, will compare the baseline HIV-1 viral loads in the genital secretions of participants infected with HIV subtype B with those of participants infected with subtype C. Approximately 315 men and women enrolled in A5175 will participate in A5185s until their discontinuation from A5175. Genital tract (GT) specimens will be collected at baseline, weeks 48 and 96, at regimen switch(es), and at study discontinuation (see Appendix VI).

## 1.0 HYPOTHESES AND STUDY OBJECTIVES

### 1.1 Hypotheses

Step 1

1.1.1 Three-drug antiretroviral (ARV) regimens administered once daily **(QD)** and twice daily **(BID)** provide similar ARV benefit in diverse areas of the world including resource-limited settings for HIV-1-infected persons with **CD4+ cell counts** <300 cells/mm^3^.

1.1.2 A three-drug ARV regimen containing a PI, administered **QD**, provides similar ARV benefit in diverse areas of the world including resource-limited settings for HIV-1-infected persons with **CD4+ cell counts** <300 cells/mm^3^ as compared with a three-drug ARV regimen administered **BID** that contains an NNRTI.

### 1.2 Primary Objective

1.2.1 To demonstrate the non-inferiority of a **QD** PI- and a **QD** NNRTI-containing regimen as compared with standard **BID** ARV therapy for the initial treatment of individuals infected with HIV-1 from diverse areas of the world.

### 1.3 Secondary Objectives

1.3.1 To determine the safety of combinations of ARV therapy for the treatment of HIV-1 infection and to characterize drug-associated toxicities observed in different geographic and cultural settings.

1.3.2 To characterize and compare the patterns and rates of ARV drug resistance during ARV therapy in diverse areas of the world.

1.3.3 To examine the effects of ARV therapy on quality of life in diverse areas of the world.

1.3.4 To characterize and compare the occurrence and outcomes of opportunistic infections, viral co-infections, and immune reconstitution syndromes, observed during ARV treatment in different geographic settings.

1.3.5 To examine associations between nutritional deficiencies and responses to ARV therapy.

1.3.6 To investigate efficacy of sequential ARV regimens after failure of primary regimens in diverse areas of the world.

1.3.7 To evaluate the effects of concomitant medications, traditional treatments, ethnicity, race, and measures of adherence on plasma ARV concentrations in individuals from diverse areas of the world.

### 1.4 Programmatic Objectives

Sustained, widespread application of ARV therapy in the areas of the world that are most affected by the AIDS epidemic will require both care providers who are appropriately trained in the use of ARV drugs and methods to measure treatment response that are applicable for resource-limited settings. A5175 will assist in the development of centers of HIV-1 treatment expertise by addressing the following programmatic objectives:

1.4.1 to assist with the transfer of immunologic, pharmacologic, and virologic technology and expertise relevant to the conduct of clinical studies of HIV-1 treatment in resource-limited settings.

1.4.2 to establish local specimen repositories for future evaluation of the usefulness of simplified measures of virologic and immunologic efficacy and ARV drug resistance in resource-limited settings.

### 1.5 Objectives for A5185s: A Genital Secretions Substudy of A5175.

See Appendix VI.

## 2.0 INTRODUCTION

### 2.1 Antiretroviral Agents

In developed countries, standard treatment for persons who are ARV-naïve consists of three-drug combinations of two nucleoside reverse transcriptase inhibitors (NRTIs) with either an HIV-1 protease inhibitor (PI) or a non-nucleoside reverse transcriptase inhibitor (NNRTI). These regimens provide effective inhibition of HIV-1 replication that results in functional immune reconstitution, decreased risk of AIDS-related events, and improved survival (D’Aquila et al. 1996; Hammer et al. 1997; Gulick et al. 1997; Autran et al. 1997; Palella et al. 1998). ACTG 384 demonstrated that in treatment-naive patients, zidovudine (ZDV) plus lamivudine (3TC) plus efavirenz (EFV) showed a strong trend to delaying sequential regimen failure and significantly delayed first regimen failure and first virologic failure as compared with the three-drug regimens consisting of either ZDV + 3TC + nelfinavir (NFV) or NFV or didanosine (ddI-EC) + stavudine (d4T) and either EFV or NFV (Robbins et al. 2003).

2.1.1 Lamivudine/Zidovudine Combination (Combivir®, 3TC/ZDV).

A combination tablet of ZDV 300 mg and 3TC 150 mg is approved for marketing by the United States (U.S.) Food and Drug Administration (FDA) and the European Agency for the Evaluation of Medicinal Products (EMEA), at a dosage regimen of one tablet **BID**. The 3TC/ZDV-combination tablet has been shown in a clinical study to be bioequivalent to the two individual ARV drugs (Moore et al. 1999). The efficacy of 3TC/ZDV was established in a randomized, open-label, parallel-group, multicenter study (n =223) that compared a regimen using the 3TC/ZDV combination tablet given **BID** plus a PI versus a regimen containing ZDV thrice daily plus 3TC **BID** plus a PI (Eron et al. 2000) (see 2.1.2 and 2.1.3 for adverse events [AEs] associated with ZDV and 3TC, respectively).

Additional information regarding 3TC/ZDV is available in the most recent Combivir package insert.

2.1.2 Zidovudine (Retrovir®, ZDV)

ZDV is generally well tolerated, particularly in persons with CD4+ cell counts >200 cells/mm^3^. The major side effects include headache, fatigue, malaise, nausea, anemia, and neutropenia. Long-term ZDV therapy is associated with myopathy and rare cases of steatosis with hepatic failure and death.

Additional information regarding ZDV is available in the most recent Retrovir package insert.

Drug Resistance Following ZDV Prophylaxis of Mother-to-Child Transmission (MTCT)

A number of studies have examined use of ZDV following ZDV perinatal prophylaxis. Rates of resistance vary from study to study (Eastman et al. 1998; Ekpini et al. 2002; Kully et al. 1999; Welles et al. 2000) and are likely influenced by the duration of ZDV exposure, the assay(s) used for resistance testing, the timing of testing, and other factors.

2.1.3 Lamivudine (Epivir®, 3TC)

3TC is a potent nucleoside that is widely used in the management of HIV-1-infected patients. Although 3TC is an effective NRTI, virus with a resistance mutation at codon 184 rapidly emerges within 2 weeks of monotherapy and is also seen with dual nucleoside regimens.

3TC is one of the best-tolerated NRTIs. AEs occur in less than 5% of patients. Side effects include headache, nausea and vomiting, malaise, fatigue and sleeplessness, anorexia, dizziness, rash, depression, anemia, neutropenia, and hyperamylasemia.

Hepatitis B

Persons who are coinfected with hepatitis B (HBV) may experience increased levels in liver function tests **(LFTs)** and exacerbation of hepatitis symptoms when 3TC is stopped. Usually these symptoms are self-limiting; however, death has been reported. The causal relationship to 3TC discontinuation is unknown. Participants should be followed closely with both clinical and laboratory follow-up for the first several months following 3TC discontinuation.

Additional information regarding 3TC is available in the most recent Epivir package insert.

2.1.4 Efavirenz (Sustiva®, EFV)

EFV is a **QD** NNRTI that has been shown to be effective in the treatment of HIV disease (Staszewski et al. 1999).

The most notable side effects associated with EFV are central nervous system (CNS) symptoms and rash. Fifty-three percent of those receiving EFV reported CNS symptoms. These symptoms included, but were not limited to, dizziness, impaired concentration, somnolence, abnormal dreams, and insomnia. Symptoms usually begin during the first or second day of therapy and generally resolve after the first 2 to 4 weeks of therapy. Symptoms may also be less noticeable if EFV is taken at bedtime. Potential for additive symptoms may occur if used concomitantly with alcohol or psychoactive drugs. Nervous system symptoms were severe in 2.0% of patients receiving EFV 600 mg QD and in 1.3% of patients receiving control regimens; and, 2.1% of EFV-treated patients discontinued therapy because of nervous system symptoms.

In multi-study comparisons of EFV-treated versus (vs.) controls, severe acute depression (1.6% vs. 0.6%) and suicidal ideation (0.6% vs. 0.3%) were reported. Participants with a history of psychiatric disorder are at greater risk. There have been occasional post-marketing reports of delusions and aberrant behavior, predominantly in those with a history of mental illness or substance abuse. Participants who experience psychiatric symptoms should contact their doctor immediately to assess the possibility that the symptoms may be related to EFV.

Among approximately 2200 treated individuals in studies and expanded access programs, the incidence of Grade 4 rash (e.g., erythema multiforme and Stevens-Johnson syndrome) was 0.14%. The median time to onset of rash in adults was 11 days, and the median duration was 16 days. EFV should be discontinued in persons developing severe rash associated with blistering, desquamation, mucosal involvement, or fever. Appropriate antihistamines and/or corticosteroids may improve the tolerability and hasten the resolution of rash.

Other side effects associated with EFV include upset stomach, diarrhea, anorexia, headache, tiredness, pancreatitis, elevated cholesterol (including HDL), elevated triglycerides, and elevated transaminases.

Teratogenicity/Developmental Toxicity

The U.S. FDA use-in-pregnancy category for EFV has been changed from Category C (Risk of Fetal Harm Cannot Be Ruled Out) to Category D (Positive Evidence of Fetal Risk). This change is a result of four retrospective reports of neural tube defects in infants born to women with first trimester exposure to EFV, including three cases of meningomyelocele and one of Dandy Walker syndrome. As EFV may cause fetal harm when administered during the first trimester to a pregnant woman, pregnancy should be avoided in women receiving EFV. Women of reproductive potential and women with bilateral tubal ligation should be counseled on the possible risks associated with pregnancy. Women should be instructed not to breast-feed while taking EFV. If a woman becomes pregnant while taking EFV during the first trimester of pregnancy, she should be apprised of the potential harm to the fetus.

Additional information regarding EFV is available in the most recent Sustiva package insert.

2.1.5 Tenofovir Disoproxil Fumarate (Viread®, TDF)

TDF (formerly known as PMPA prodrug or GS-4331-05) is licensed for the treatment of HIV-1 infection in combination with other agents. TDF is an orally bioavailable acyclic nucleotide analogue with activity in vitro against retroviruses, including HIV-1 and HIV-2 and hepadnaviruses. Although TDF is a nucleotide analogue, it has the same mechanism of action and resistance pattern as NRTIs. Therefore, for simplification of discussion, TDF in this study will be referred to as an NRTI.

Safety Profile

Assessment of AEs is based on two studies (902 and 907) in which 653 treatment-experienced patients received double-blind treatment with TDF 300 mg (n=443) or placebo (n=210) for 24 weeks followed by extended treatment with TDF. The most common AEs in patients receiving TDF with other ARVs in clinical trials were mild to moderate gastrointestinal events, such as nausea, diarrhea, vomiting, and flatulence. Less than 1% of patients discontinued participation in the clinical studies because of gastrointestinal AEs. Laboratory abnormalities observed in these studies occurred with similar frequency in the TDF and placebo-treated groups.

Renal Impairment

Evidence of renal toxicity was noted in four animal species at exposures (based on the area under the curve [AUC]) 2 to 20 times higher than those observed in humans.

In study 903, involving 600 treatment-naïve patients treated for up to 144 weeks, changes in renal function (serum creatinine elevation, hypophosphatemia, glycosuria, proteinuria) were observed with similar frequency in the TDF-containing arm as compared with the d4T-containing arm. No patients were discontinued from the study for renal **AE**s.

Serious renal AEs were reported in 0.3% of patients (n = 8870) treated with TDF in the Viread global expanded access program. Renal events reported in clinical practice include increased serum creatinine, renal insufficiency, renal failure, acute renal failure, proximal tubulopathy, proteinuria, acute tubular necrosis, Fanconi syndrome, and nephrogenic diabetes insipidus.

TDF should be avoided with concurrent or recent use of a nephrotoxic agent. Participants at risk for, or with a history of, renal dysfunction and patients receiving concomitant nephrotoxic agents should be carefully monitored for changes in renal function and serum phosphorus.

Hepatitis B Virus (HBV)

Exacerbations of HBV have been reported in patients after discontinuation of TDF. Participants, who are coinfected with HBV, may have increased values on **LFTs** and exacerbation of hepatitis symptoms when TDF is stopped. Usually these symptoms are self-limiting; however, serious complications have been reported. The causal relationship to TDF discontinuation is unknown. Participants coinfected with HBV and HIV should be closely monitored with both clinical and laboratory follow-up for several months after stopping TDF treatment.

Bone Toxicity

GS-01-926. Because TDF administered in toxicology studies to rats, dogs, and monkeys at exposures AUCs between 6- and 12-fold higher than those achieved in humans caused bone toxicity, study 926 included a number of assessments for bone-related toxicity in pediatric patients. All patients enrolled had baseline lumbar spine densitometry by dual-energy x-ray absorptiometry (DEXA) to measure bone mineral density (BMD). There was a high prevalence of osteopenia in these patients at baseline. Two patients were shown to have a confirmed decreases in BMD of >6% at week 24 relative to baseline, with no consistent trends in bone-related laboratory changes or TDF blood levels found.

In study 903, through 144 weeks, decreases from baseline BMD were seen at the lumbar spine and hip in both arms of the study. The proportion of patients who met a protocol-defined value of BMD loss (5% decrease in spine or 7% decrease in hip) was higher in the TDF group than in the d4T group. In addition, there were significant increases in levels of four laboratory parameters of bone metabolism (serum bone-specific alkaline phosphatase, serum osteocalcin, serum C-telopeptide, and urinary N-telopeptide) in the TDF group compared with the d4T group, suggesting increased bone turnover. Serum parathyroid hormone levels were also higher in the TDF group. There were five bone fractures reported in the TDF group compared with eleven in the d4T group; no pathologic fractures were identified over 48 weeks of study treatment. The clinical significance of changes in the BMD and the biochemical markers is unknown, and follow-up is continuing to assess long-term impact.

Teratogenicity/Developmental Toxicity

Chronic administration of TDF to fetal and immature animals of multiple species at doses higher than used in humans has resulted in bone abnormalities; these effects were dose-, exposure-, age-, and species-specific. Abnormalities ranged from minimal decrease in bone mineral density and content to severe, pathologic osteomalacia. Evidence of nephrotoxicity has been observed. Studies in rats have demonstrated that TDF is secreted in milk. Subcutaneous administration of TDF to pregnant rhesus macaque monkeys resulted in a fetal/maternal concentration of 60%, demonstrating that TDF does cross the placenta (van Rompay KKA, et al. 1998). There are no data on whether TDF crosses the placenta or is excreted in breast milk in humans. No studies of TDF have been conducted in pregnant women or neonates.

Additional information regarding TDF is available in the most recent Viread package insert.

2.1.6 Emtricitabine (Emtriva™, FTC)

FTC is a synthetic nucleoside analogue with activity against HIV-1 reverse transcriptase. In controlled clinical studies (FTC-301A, FTC-303), the antiviral activity of FTC was superior to d4T and was equivalent to 3TC.

Safety Profile

The most common AEs that occurred in patients receiving FTC with other ARVs in clinical trials were headache, diarrhea, nausea, and rash, which were generally of mild to moderate severity. Approximately 1% of patients discontinued participation in the clinical studies due to these events. All AEs were reported with similar frequency in FTC and control treatment groups with the exception of skin discoloration, which was reported with higher frequency in the FTC-treated group.

Hepatitis B Virus

Exacerbations of HBV have been reported in patients after discontinuation of FTC. Participants, who are coinfected with HBV, may have increased values on **LFTs** and exacerbation of hepatitis symptoms when FTC is stopped. Usually these symptoms are self-limiting; however, serious complications have been reported. The causal relationship to FTC discontinuation is unknown. Participants coinfected with HBV and HIV should be closely monitored with both clinical and laboratory follow-up for several months after stopping FTC treatment.

Additional information regarding FTC is available in the most recent Emtriva package insert.

2.1.7 Emtricitabine and Tenofovir Disoproxil Fumarate Fixed Dose Combination (FDC) Tablet

Gilead Sciences has developed Truvada®, a new product containing FTC 200 mg and TDF 300 mg in an FDC tablet formulation. A New Drug Application (NDA) for the FDC was filed with the U.S. FDA on March 12, 2004, and was approved on August 2, 2004.

Study GS-US-104-172 was a phase I, 28-day, randomized, four-way crossover, pharmacokinetic (PK) study in healthy volunteers designed to evaluate the bioequivalence of the FTC/TDF combination tablet compared with the FTC capsule and TDF tablet administered concurrently and also the effect of food (high-fat meal and light meal) on **PK** (Kearney et al. 2004). The results demonstrated bioequivalence between the FTC/TDF combination tablet and the FTC capsule and TDF tablet formulations when administered separately. Administration of the FTC/TDF combination tablet with either a high-fat meal or light meal increased TDF exposure by approximately 30% compared with fasted-state administration. Clinical experience with TDF indicates that the effect of food on TDF exposure is not of clinical relevance. FTC and TDF, either administered as an FDC tablet (containing FTC 200-mg/TDF 300-mg) or coadministered as FTC 200 mg capsule and TDF 300-mg tablet separately, were well tolerated.

Several studies have assessed the safety and efficacy of FTC with TDF, although none using FDC. Study M02-418 was a phase III, randomized, open-label, multicenter study designed to compare lopinavir (LPV) 800 mg/ritonavir (RTV) 200 mg QD vs. LPV 400 mg/RTV 100 mg BID with the background regimen of FTC 200 mg QD and TDF 300 mg QD in ARV-naïve patients with HIV-1 RNA >1000 copies/mL (Molina et al. 2004; Podzamczer et al. 2003; Gathe et al. 2004). A total of 190 patients between the ages of 19-75 years were enrolled; 115 to the QD arm and 75 to the BID arm. At week 48, based on the intent to treat (ITT) (NC=F) analysis, 70% of participants in the QD regimen demonstrated HIV-1 RNA <50 copies/mL compared with 64% of those in the BID group (95% CI: -7%; 20%). Gastrointestinal AEs were the most common cause for discontinuation. Overall, the most common AEs (>3%) reported were diarrhea, nausea, and vomiting, with diarrhea being reported significantly higher in the QD group (16% vs. 5%; p=0.04). The most common Grade 3/4 laboratory abnormalities (>3%) reported were increased ALT (>5 x upper limit of normal [ULN]), AST (>5 x ULN), triglyceride (>750 mg/dL), and amylase (>2 x ULN) levels; no significant differences between the two groups were observed (Gathe et al. 2004).

Study 934 is a phase III, randomized, open-label, noninferiority, multicenter study designed to compare a regimen of TDF 300 mg + FTC 200 mg + EFV QD with a regimen of ZDV 300 mg/3TC 150 mg BID (as FDC Combivir) + EFV QD in **ARV**-naïve, HIV-1-infected participants (Gallant et al. 2006). The 48-week data demonstrated that using the time to loss of virologic response as the primary analysis (where missing, switch, or early termination is counted as a failure), the proportion of participants with plasma HIV-1 RNA levels <400 copies/mL in an ITT population (n=487) was 84% in the TDF + FTC group compared with 73% in the ZDV/3TC group (p=0.002). The proportion of participants with plasma HIV-1 RNA levels < 50 copies/mL was 80% in the TDF+ FTC group versus 70% in the ZDV/3TC group (p=0.020). Significant differences were also seen between the TDF+ FTC and the ZDV/3TC groups in the proportion of participants with increases in CD4+ cell counts (190 and 158 cells/mm^3^, respectively; p = 0.002)

Safety analysis, based on 511 participants who received any study medication, showed that discontinuation due to AEs occurred more frequently in the ZDV/3TC group (9%) than in the TDF+ FTC group (4%) (p=0.02). The most common AE resulting in discontinuation related to study drug for the ZDV/3TC group was anemia (14/254) and NNRTI-associated rash (2/257) for the TDF+ FTC group. Renal safety was similar in the two groups, and no participant discontinued study medication because of renal events. A significantly (p<0.001) greater percentage of participants in the TDF+ FTC arm had a lower mean increase from baseline in fasting total cholesterol levels (21 mg/dL) compared with participants in the ZDV/3TC arm (35 mg/dL). At week 48, total limb fat was significantly less in a subset of participants receiving ZDV/3TC (mean of 6.9 kg or 15.2 pounds; n=49) compared with a subset of participants receiving TDF+ FTC (mean 8.9 kg or 19.6 pounds; n=51; p=0.03). All participants with confirmed >400 copies/mL of HIV-1 RNA at week 48 or early discontinuation were analyzed for genotypic resistance. Genotype data were limited to 23 participants on ZDV/3TC and 12 participants on TDF+ FTC and showed mostly M184V/I (3% in ZDV/3TC participants vs. 1% in TDF + FTC participants) and/or EFV-resistance mutations (7% in ZDV/3TC vs. 4% in TDF + FTC participants), with no participants developing the K65R mutation.

Lactic acidosis and severe hepatomegaly with steatosis, including fatal cases, have been reported with the use of nucleoside analogues alone or in combination, including FTC, TDF, and other ARVs.

FTC/TDF is designated as FDA use-in-pregnancy Category B. More information concerning FTC/TDF coformulation, is available in the most recent Truvada package insert.

2.1.8 Didanosine (Videx®, ddI-EC)

ddI-EC is an enteric-coated capsule of ddI. The capsule does not require the buffering used in the tablet formulation. The same restrictions on food intake apply to the **enteric-coated** capsules as to the tablets. The most common toxicities associated with ddI-EC are gastrointestinal upset, peripheral neuropathy, and pancreatitis.

TDF - ddI-EC Pharmacokinetic Interaction

ddI-EC 400 mg **QD** (all individuals ≥60 kg) given 2 hours before TDF 300 mg with a light meal, resulted in an approximately 46% increase in ddI exposure relative to the administration of ddI-EC alone in the fasted state, as measured by AUC ddI concentration. Coadministration of ddI-EC and TDF 300 mg with a light meal resulted in an approximate 60% increase in ddI exposure relative to the administration of ddI-EC alone in the fasted state. Coadministration of ddI-EC capsules had no effect on the AUC of TDF (see the Precautionary Medications section [5.4.3] of this protocol for suggested dosage adjustments).

Additional information regarding ddI is available in the most recent Videx package insert.

2.1.9 Atazanavir (Reyataz®, ATV)

ATV is one of a new class of azapeptide PIs for HIV-1 that differs from the existing peptidomimetic PIs by its C-2 symmetric chemical structure. ATV has antiviral activity that has been demonstrated in separate studies to be comparable to nelfinavir and EFV.

Phase II and III studies have demonstrated good overall safety and tolerability of ATV. The most frequently seen AEs in the phase II and III studies are infection (46%), nausea (28%), headache (24%), abdominal pain (19%), diarrhea (19%), rash (19%), peripheral neurologic symptoms (15%), vomiting (13%), flu syndrome (12%), increased cough (12%), jaundice (12%), and fever (10%).

The most common laboratory abnormality observed in clinical studies of ATV is an isolated increase in unconjugated (or indirect) bilirubin, the mechanism for which has been shown to be inhibition of the enzyme UDP glucuronosyl transferase. For individuals who received the 400 mg dose, elevations in serum levels of unconjugated bilirubin were common. While the median increase above baseline for total bilirubin was only 0.6 mg/dL, approximately 40% of individuals had a total bilirubin >2.5 times the ULN. However, only 5% of individuals had a total bilirubin >5 times the ULN. Up to 10% of individuals demonstrated clinical signs of hyperbilirubinemia (scleral icterus or jaundice). Rates of hyperbilirubinemia as well as absolute levels were higher in individuals with a genetic phenotype similar to that observed for individuals with Gilbert’s syndrome.

Results from embryo-fetal development and genetic toxicology studies show that ATV is not teratogenic in rats or rabbits and does not present a genotoxic risk to humans. To date, there are no adequate or well-controlled studies in pregnant women.

Additional information regarding ATV is available in the most recent Reyataz package insert.

2.1.10 Nevirapine (Viramune®, NVP)

NVP is an NNRTI with activity against HIV-1. The most frequently reported AEs related to NVP therapy are rash, fever, nausea, headache, and abnormal LFTs. The experience from clinical trials and clinical practice has shown that the most serious **AEs** are clinical hepatitis/hepatic failure, Stevens-Johnson syndrome (SJS), toxic epidermal necrolysis (TEN), and hypersensitivity reactions (HSRs) characterized by rash, constitutional findings, and organ dysfunction.

Hepatic Toxicity

In controlled clinical trials, symptomatic hepatic events, regardless of severity, occurred in 4% (range 0% to 11.0%) of participants who received NVP and 1.2% of participants in control groups. Increased AST or ALT levels before the start of ARV treatment and/or history of HBV or hepatitis C virus (HCV) infection are associated with a greater risk of hepatic AEs. Severe, life-threatening, and in some cases fatal hepatotoxicity (including fulminant and cholestatic hepatitis, hepatic necrosis, and hepatic failure) has been reported in patients treated with NVP. In some cases, participants presented with non-specific prodromal signs or symptoms of hepatitis and progressed to hepatic failure with transaminase elevation, with or without hyperbilirubinemia, prolonged PTT, or eosinophilia. Physicians and participants should be vigilant for the appearance of signs or symptoms of hepatitis, such as fatigue, malaise, anorexia, nausea, jaundice, bilirubinuria, acholic stools, liver tenderness, or hepatomegaly. The diagnosis of hepatotoxicity should be considered in this setting, even if **LFTs** are initially normal or alternative diagnoses are possible. Hepatic dysfunction may be isolated or associated with signs of hypersensitivity including, but not limited to, severe rash or rash accompanied by fever, general malaise, fatigue, muscle or joint aches, blisters, oral lesions, conjunctivitis, facial edema, and/or hepatitis, eosinophilia, granulocytopenia, or renal dysfunction.

The risk of hepatotoxicity in women with **CD4+ cell counts** >250 cells/mm^3^, including pregnant women receiving chronic treatment for HIV infection, is considerably higher (12-fold) compared with women with **CD4+ cell counts** ≤250 cells/mm^3^ (11% vs. 0.9%). Some of these events have been fatal. Men with higher **CD4+ cell counts** (>400 cells/mm^3^) also have a higher risk of hepatotoxicity than men with lower CD4+ cell counts (6.3% vs. 1.2%). This subset of patients was identified by analyses of CD4+ cell count at the time of NVP treatment initiation. The greatest risk of severe and potentially fatal hepatic events (often associated with rash) occurs in the first 6 weeks of NVP treatment. However, the risk continues after this time and participants should be monitored closely for the first 18 weeks of NVP treatment. In some cases, hepatic injury progresses despite discontinuation of treatment.

Intensive clinical and laboratory monitoring, including LFTs, is essential at baseline and during the first 18 weeks of NVP treatment to detect potentially life-threatening hepatic events and skin reactions. NVP should not be restarted following severe hepatic, skin, or HSR. In addition, the 14-day lead-in period with NVP 200 mg daily dosing must be strictly followed. LFTs are required at weeks 2, 4, 6, and 8, after each time NVP is initiated, then monthly through the 20th week of NVP treatment. All participants developing a rash, at any time during NVP treatment, but particularly during the first 18 weeks, should have LFTs performed at that time. After the initial 18-week period, frequent clinical and laboratory monitoring should continue throughout NVP treatment.

Rash

The most common clinical toxicity of NVP is rash (16% of patients on combination regimens in phase II/III controlled studies). Severe and life-threatening skin reactions, including fatal cases, have occurred in individuals treated with NVP. These have included cases of SJS, TEN, and HSR characterized by rash, constitutional findings, and organ dysfunction.

Severe rashes occur most frequently within the first 28 days of treatment; 25% of the patients with severe rashes required hospitalization, and one patient required surgical intervention. Approximately 7% of patients discontinue NVP due to rash.

In one trial, concomitant use of prednisone to prevent NVP-associated rash increased the incidence and severity of rash during the first 6 weeks of NVP therapy. The use of prednisone to prevent NVP-associated rash is not recommended. Participants should be advised to promptly notify their health care provider if they develop any rash or signs and symptoms of a HSR. Participants who experience rash during the first 2 weeks of treatment should not have the dose of NVP increased until the rash has resolved.

Participants developing signs or symptoms severe skin reactions or HSRs must discontinue NVP immediately and must not be re-challenged.

Drug Resistance Following Single-Dose NVP Prophylaxis

In HIVNET 012, pregnant women received a single dose of NVP at the onset of labor. NVP resistance mutations were detected in 70 of 279 (25%) women 6-8 weeks after delivery (Eshleman et al. 2004). The frequency of NVP resistance after single**-**dose **(SD)** NVP varies by HIV-1 subtype, and is particularly high among women with subtype C (Eshleman et al. 2005). Furthermore, recent studies show that population-sequencing-based genotyping assays tend to underestimate the risk of NVP resistance, compared with more sensitive resistance assays (Johnson et al. 2005). In HIVNET 012, HIV variants with NVP resistance mutations faded from detection by 12-24 months after delivery using a population-sequencing-based assay (Eshleman et al. 2001). However, more recent studies using a more sensitive resistance assay show that K103N-containing HIV-1 variants persist above baseline levels for a year or more after **SD** NVP in some women (Flys et al. 2005). Among participants who failed prior treatment with an NNRTI, selection of minority variants with NNRTI resistance mutations may compromise subsequent treatment with an EFV-containing regimen (Mellors et al. 2003; Lecossier et al 2005). In the setting of **SD** NVP prophylaxis, prior exposure of women to SD NVP may lower their virologic response to a subsequent NNRTI-containing treatment regimen (Jourdain et al. 2004). Further studies are needed to evaluate the clinical impact of NVP resistance after **SD** NVP prophylaxis.

EFV Substitution with NVP

The strategy of substituting NVP for treatment-limiting toxicity related to EFV has not been well studied. Given the known toxicity profiles of the two drugs, it would seem reasonable to try substitution of NVP for treatment-limiting CNS toxicity (e.g., dizziness, somnolence, bad dreams, and confusion) ascribed to EFV. Although the molecular structures of NVP and EFV are not related, substitution for NNRTI class-specific toxicities (e.g., increased AST/ALT, rash) is less supported, although some anecdotal information is available (Clarke et al. 2000; Podzamczer et al. 2000; Soriano et al. 2000).

Clarke et al. reported on eight individuals who experienced NVP-related rash and subsequently changed to an EFV-containing regimen. Of these eight, five continued their EFV regimen without recurrence of side effects. Podzamczer et al. reported that two individuals who experienced severe HSRs with NVP changed to an EFV-containing regimen (with a corticosteroid taper) with good tolerance. Soriano et al. reported findings from an EFV expanded access program: of eight individuals with a history of NVP-associated rash, only one developed a rash after beginning EFV. Although the temporal sequence was NVP to EFV in each report, the incidence of cross-toxicity between the two drugs is unknown.

Per the most recent Viramune package insert it is recommended that NVP be dosed BID. Additional information regarding NVP is available in the most recent Viramune package insert.

2.1.11 Stavudine (Zerit®, d4T) Immediate Release Formulation

d4T is an approved nucleoside analogue that has been approved for use in combination with other ARV drugs for the treatment of HIV-1-infected individuals (Griffith et al. 1996; Anderson et al. 1993; Murray et al. 1995; Peterson et al. 1995; Spruance et al. 1997).

The most common toxicity associated with d4T is peripheral neuropathy and, much less commonly, hepatic damage, pancreatitis, and lactic acidosis (for which women may have an increased risk when it is used with ddI in pregnancy.)

When substitution of d4T for ZDV was permitted in ACTG 320, no difference in outcome was seen with use of either NRTI combination (Hammer et al. 1997).

Additional information regarding d4T is available in the most recent Zerit package insert.

### 2.2 HIV Primary Care

Many HIV-1-infected people living in resource-limited **settings (RLS)** do not receive medical care for treatment or prevention of the complications of HIV-1 infection. Several studies suggest that standardized "HIV primary care" provides modest reductions in morbidity and mortality in HIV-1-infected persons in **RLS** (Pallela et al. 1998; Brodt et al. 1997; Dore et al. 2002). The standardized HIV primary care selected for A5175 is from **World Health Organization (**WHO**)**/**Joint United Nations Programme on HIV/AIDS (**UNAIDS**)** guidelines for HIV/AIDS Care and Support in combination with local standards of care at the study sites. Standardized HIV primary care will include treatment and prevention of vitamin deficiency, sexually transmitted diseases (STDs), diarrhea, tuberculosis (TB), endemic infections (enteric helminths and malaria), and other AIDS-associated opportunistic pathogens. A table detailing the currently available standards of care for the prevention and treatment of OIs and AIDS-defining illnesses is included in the A5175 and A5185s Manual of Operations (MOPS) located on the A5175 PSWP ([**https://www.actgnetwork.org/protocols/protocol_specific_search.aspx**](https://www.actgnetwork.org/protocols/protocol_specific_search.aspx))**.**

### 2.3 Rationale

Most previous trials of ARV efficacy have been conducted in economically developed countries of North America and Europe. Because of the demographics of the early AIDS epidemic in developed areas of the world, most participants in previous ARV clinical trials have been Caucasian men. Thus, much of what is known about factors that affect ARV efficacy comes from studies of persons with relatively homogeneous racial, cultural, and environmental backgrounds. Direct comparisons of ARV efficacy in human populations that are more reflective of the worldwide demographics of HIV-1 infection are needed.

Most HIV-1-infected persons live in **RLS**. The efficacy of ARV regimens for initial treatment of HIV-1 infection in **RLS** could be affected by differences in inherent and acquired ARV resistance among different HIV-1 subtypes; the frequency and severity of drug toxicities in different human populations; PK interactions between ARVs and other pharmaceuticals, traditional medicines, or dietary items; and the ease and convenience of dosing and storage of ARV formulations in different cultural and environmental settings. Little is known about how these factors affect the efficacy of ARV combinations in HIV-1-infected persons living in **RLS**.

Existing data suggest that strict adherence to treatment is a major determinant of ARV efficacy. Since factors that could affect adherence (e.g., drug toxicity, co-morbidities, and nutritional status) are likely to differ in **RLS**, the relative efficacy of ARV regimens in such settings may also differ. One approach to increase adherence is to decrease the complexity of ARV regimens by decreasing the number of pills in the regimen and the number of doses per day. It is expected that simple **QD** regimens will increase adherence and therefore ARV efficacy. The availability of **QD** regimens will also facilitate the implementation of directly observed models of ARV delivery. Thus, three-drug ARV combinations that require fewer pills dosed **QD** have potential advantages for the treatment of HIV-1 infection in **RLS**.

A regimen that contains co-formulated ZDV/3TC + an NNRTI (either EFV or NVP) is the current standard of care for initial ARV treatment in most areas of the world. However, there are several limitations to this regimen in **RLS**. First, the relatively short intracellular half-life of ZDV necessitates **BID** administration and limits the usefulness of this regimen in directly observed models of delivery. Second, the toxicities of NNRTIs (e.g., NVP liver toxicity and EFV teratogenicity) limit their application as well. Finally, the increasing use of **SD** NVP for the prevention of MTCT HIV-1 transmission in **RLS** and the high risk of acquiring NNRTI resistance following **SD** NVP may lead to overall decreased NNRTI efficacy in these areas of the world. If A5175 identifies compromised efficacy of ARV treatment containing an NNRTI in women with prior exposure to NVP during pregnancy, this finding will provide additional support either for the use of alternative interventions for the prevention of MTCT or for the use of ARV regimens that do not contain NNRTIs for the treatment of chronic HIV-1 infection in these populations.

A5175 will be implemented in resource-limited regions of the world that have large populations of HIV-1-infected persons. Participants will be residents of Africa (Malawi, South Africa, Zimbabwe), Asia (India, Thailand), the Caribbean (Haiti), and South America (Brazil, Peru), in addition to North America (United States). Thus, the study population will be a diverse cultural and ethnic sample and will differ significantly from the study populations of most existing clinical trials of ARV efficacy. In addition to determining whether **QD** ARV regimens are an effective approach to ARV therapy, it is expected that the results of A5175 will provide guidance for the future application of ARV in **RLS**.

Inclusion of Women Who Received Prior ARV Prophylaxis During Pregnancy

Women who have received either **SD** NVP or ZDV monotherapy for perinatal prophylaxis are eligible for enrollment in A5175 (see section 4.0). This decision was made after careful consideration of several issues. First, the A5175 protocol team believed that it would be inappropriate to exclude women in **RLS** because they previously had received perinatal prophylaxis. Second, while A5175 focuses on implementation of ARV treatment in **RLS**, prevention of MTCT **of HIV-1** in these settings is also extremely important. Although resistance to ZDV and NVP can emerge when these drugs are used for perinatal prophylaxis, it is not known if emergence of resistance will compromise subsequent treatment of HIV-infection with a multi-drug regimen.

Resistance is relatively uncommon following short courses of ZDV monotherapy. While NVP resistance is frequently seen following **SD** NVP prophylaxis, resistance mutations fade from detection in plasma after delivery. It is not known whether this brief exposure to NVP is sufficient to establish resistant variants (e.g., in latent reservoirs or as minor variants in plasma) at sufficient levels to compromise subsequent treatment with an NNRTI-containing regimen. If a sufficient number of women who have received these regimens are enrolled into A5175, it will allow us to examine whether prior perinatal prophylaxis limits the efficacy of the treatment regimens. The planned A5175 resistance studies will also determine the rate of emergence of ZDV and NNRTI resistance following treatment with each of the regimens. This information will help to evaluate the potential impact of the A5175 regimens on the efficacy of ZDV and NVP prophylaxis in future pregnancies.

Treatment of Hepatitis B Virus Infection

The U.S. Department of Health and Human Services (USDHHS) issued updated Guidelines for the Use of Antiretroviral Agents in HIV-1 Infected Adults and Adolescents in **November 2008** (see <http://aidsinfo.nih.gov/ContentFiles/AdultandAdolescentGL.pdf>). The U.S. DHHS Guidelines (henceforth “the Guidelines”) recommend that two **ARV** agents active against HBV be used when antiretroviral therapy **(ART)** is commenced in persons co-infected with HIV-1 and HBV because of the potential for development of drug-resistant HBV when a single agent is used.

The guidelines are relevant to A5175 because participants who are potentially HIV-1 and HBV coinfected are randomized to treatment arms in which the drugs active against HBV are 3TC, FTC, and TDF. Two A5175 treatment arms contain only 3TC (Arm 1A) or FTC (Arm 1B) and one treatment arm contains both FTC and TDF (Arm 1C). A5175 has been modified to allow the treatment of HIV-1 and HBV co-infected persons to be consistent both with the Guidelines and with local guidelines and standards of care in areas of the world where A5175 is conducted. Because participation in A5175 may provide a benefit to HBV coinfected persons, HBV co-infected persons will not be excluded from participation in A5175. The following special considerations will be made for participants with HIV/HBV coinfection:

- Hepatitis B surface antigen **(HBsAg)** tests will be performed at screening and results of the test will be provided to participants undergoing screening irrespective of whether they enter the study, and to all participants who were enrolled under protocol Version 1.0 and were tested at study entry. Site investigators will discuss with all HIV/HBV coinfected persons the test results and what options, if any, exist for HBV treatment outside of A5175 at their sites.
- Information about the implications of chronic HBV infection and the potential effects of the study-assigned **ARV** regimens on HBV will be included in the sample informed consent document. Participants who are infected with HBV will be encouraged to discuss with their care providers whether study participation is in their best interest.
- Whether individual HBV-HIV coinfected persons are enrolled in A5175 at individual **non-U.S and U.S. CRS**s will be left to the discretion of site investigators. If treatment of HIV-1 infection with **ARV** regimens that include 3TC or FTC but without TDF is NOT consistent with the local standard of care, site investigators may elect not to enroll HBV/HIV coinfected persons into A5175.
- HBV/HIV coinfected persons who choose to participate in A**5175** and who receive only one study-provided, active anti-HBV agent may **receive entecavir** **(**provided outside of the study**)** for the treatment of HBV infection, at the discretion of the site investigator.

**Data Safety and Monitoring Board (DSMB) Review, May 2008**

**The DSMB met in closed session on May 6, 2008, to perform a regularly-scheduled review of A5175/PEARLS safety and efficacy data. This included data on the primary study outcome, which is time to treatment failure defined by the protocol. The DSMB found conclusive evidence that the regimen consisting of FTC + ATV + ddI-EC (Arm 1B) was inferior to 3TC/ZDV and EFV (Arm 1A, the control arm) regarding the rate of primary study events (p = 0.0012). The estimated hazard ratio for treatment failure in Arm 1B compared to Arm 1A was 1.67 (99.8% confidence limits 1.02, 2.75). The hazard ratio for the primary efficacy endpoint did not change significantly over time, and there was no suggestion that the difference between these two arms in the hazard of reaching primary endpoint diminished over longer follow-up. The majority of events were virologic failures. The comparison for HIV disease progression (one component of the primary study event endpoint) also suggested inferiority of the regimen of FTC + ATV + ddI-EC compared to 3TC/ZDV and EFV. The hazard ratio for the HIV disease progression comparison in Arm 1B compared to Arm 1A was 3.0. If adjusted for the sequential monitoring in the same way as the primary endpoint, this comparison did not reach the pre-defined significance level (99.8% confidence limits 0.61, 14.9; unadjusted p = 0.0255). The small number of clinical progressions limited the confidence with which firm conclusions could be reached. There was no evidence of a mortality difference between Arms 1B and 1A. The results exhibited reasonable consistency over clinical sites.**

**Based on these results, the A5175 team recommended that study treatment with the combination of FTC (or 3TC) + ATV + ddI-EC should be changed to another ARV regimen as soon as possible. Participants who were receiving this combination were asked to return their study medications. Decisions about the choice of future ARV regimens for those participants who were receiving FTC, ddI-EC, and ATV were to be made on a case-by-case basis by participants and their physicians.**

## 3.0 STUDY DESIGN

A5175 is a phase IV, randomized, open-label, three-arm study of antiviral efficacy in 1520 HIV-1-infected, ARV treatment-naive participants ≥18 years of age with CD4+ cell counts <300 cells/mm^3^. A5175 is designed to evaluate the efficacy of three ARV combination regimens consisting of two NRTIs + PI and two NRTIs + NNRTI administered **QD** compared with a regimen containing two NRTIs + NNRTI administered **BID**. The primary objective is to compare **QD** PI-containing and **QD** NNRTI-containing regimens with standard **BID** ARV therapy. These comparisons will be evaluated with time to treatment failure of the three initial study treatment arms as defined in section 9.2.1.

All randomized participants will be followed until either 30% have reached the primary endpoint or for a total study duration of 2.5 years after randomization of the first participant, whichever occurs later in time. A minimum study duration of 2.5 years will ensure not only an adequate number of events for the primary endpoint, but will also provide adequate duration of follow-up to infer noninferiority of one treatment arm compared with another. **On November 3, 2009, the DSMB reviewed the observed rates of primary endpoints and decided that continuation of the study beyond May 2010 would not greatly improve the ability of the study to answer the primary research question regarding the comparison of Arms 1A to 1C. At the recommendation of the DSMB, the study will close to follow-up on May 31, 2010.**

The following ARV study drugs, presented by class, will be provided by A5175.

Table 1. Classes and Dosing of Study Drugs

|  | NRTI | | | | | | | | NNRTI | | PI |
| --- | --- | --- | --- | --- | --- | --- | --- | --- | --- | --- | --- |
|  | 3TC/ZDV | ZDV | 3TC | FTC | TDF | FTC/TDF | ddI-EC | d4T | EFV | NVP | ATV |
| BID | X | X | X |  |  |  |  | X |  | X |  |
| QD |  |  | X | X | X | X | X |  | X | X | X |

NOTE: Women who become pregnant on study may need to change ARV drugs (see 7.6, Procedures in the Event of On-Study Pregnancy and Breast‑Feeding, for indications).

Step 1: Initial Study Regimen

At study entry participants will be randomized (1:1:1) to one of the following treatment arms.

Arm 1A: 3TC/ZDV 150 mg/300 mg PO BID + EFV 600 mg PO QHS

Arm 1B: FTC 200 mg PO QD + ATV 400 mg PO QD + ddI-EC 400 mg PO QD for participants who weigh ≥60 kg or ddI-EC 250 mg PO QD for participants who weigh <60 kg

Arm 1C: FTC/TDF 200 mg/300 mg PO QHS + EFV 600 mg PO QHS

Drug Substitutions

See sections 5.1.1.1 and 7.5.16 for allowable drug substitutions.

All Step 1 drug substitutions must be pre-approved by the A5175 Clinical Management Committee ([actg.cmca5175@fstrf.org](mailto:actg.cmca5175@fstrf.org)).

Definition of Virologic Failure for Loss of Potency

Virologic failure is defined as two successive measurements of plasma HIV-1 RNA ≥1000 copies/mL, with the first measurement at the week 16 visit or later. This definition will be used to assess the Step 1 primary endpoint and to define virologic failure for regimen switches on Step 2.

Criteria for Switching Study Treatment Regimens

In Step 1, participants who have both virologic failure and either AIDS progression or a decrease in **CD4+ cell counts** to <50% of their Step 1 maximum, will be counseled on ARV treatment adherence and offered a switch to a Step 2 (second) regimen.

NOTE: If participants develop an AIDS-defining illness in the absence of HIV-1 RNA data, participants will be allowed to switch steps at the discretion of the site investigator.

Participants who experience virologic failure without disease progression (a new or recurrent AIDS-defining OI or malignancy occurring at the week 12 visit or later or a decreased **CD4+ cell count**), will be counseled on ARV adherence. (A list of AIDS-defining opportunistic infections and malignancies is provided in the A5175 and A5185s MOPS, located on the PSWP [**https://www.actgnetwork.org/protocols/protocol_specific_search.aspx**](https://www.actgnetwork.org/protocols/protocol_specific_search.aspx)**.** Since these participants may have continued benefit from the Step 1 regimen despite virologic failure, and second regimen options may often be limited in **RLS** of the world, a switch to a Step 2 regimen will not be required. The decision to offer these participants a switch to a Step 2 regimen will be left to the discretion of the site investigator after assessing potential for drug resistance to the current regimen and counseling the participant on ARV treatment adherence. If either AIDS progression or a decrease in CD4+ **cell** count to <50% of the Step 1 maximum occurs at any time for these participants (minimum and maximum CD4+ **cell counts** will each be a mean of two consecutive measurements), a switch to a Step 2 (second) regimen will be offered.

The clinician will decide whether the drug substitution is for toxicity or for virologic failure due to presumed drug resistance. Participants on Step 1 who have drug substitutions for toxicity will remain on Step 1. The Step 2 regimens are for management of presumed ARV drug resistance.

NOTE: Changes in the Step 1 **ARV** regimen for management of **ARV** toxicities do not require entry into Step 2.

Step 2: Second Study Regimen for Management of Presumed and/or Documented Antiretroviral Drug Resistance

The purpose of Step 2 is to provide a second treatment regimen for management of presumed and/or documented loss of potency due to the development of drug resistance with the **ARV** agents used in Step 1. Step 2 regimens will consist of any potentially effective combination of three or more ARV drugs available either through A5175 or **locally**. Site investigators, according to expected patterns of resistance following Step 1 failure, will construct Step 2 regimens. Therefore, if the Step 1 regimen included an NNRTI, participants will receive one or more PI(s) in Step 2. If the Step 1 regimen included ATV, participants will receive EFV in Step 2.

Arm 2A (follows Arm 1A failure): NRTI + NRTI + PI(s)

Arm 2B (follows Arm 1B failure): NRTI + NRTI + EFV

Arm 2C (follows Arm 1C failure): NRTI + NRTI + PI(s)

It is expected that high-level 3TC and FTC resistance will occur during most Step 1 regimen failures. However, since Step 2 treatment options are limited and 3TC or FTC may provide continued benefit despite the presence of high-level resistance, 3TC or FTC will be allowed as a third NRTI in Step 2 regimens when potentially viable two-NRTI combinations are not available, at the discretion of the site investigator.

Low-dose RTV may be used to boost ATV in Step 2, if it is available from outside of A5175 (RTV is not provided by the stud**y**)**.** More than one PI given at a therapeutic dose may be used in Step 2.

RTV must be used to boost ATV whenever ATV is given with TDF.

Participants will be followed on Step 2 for the duration of the study. The drugs used in the Step 2 regimen may be changed, as appropriate, at the discretion of the site investigator.

Since all ARV resistance testing performed in A5175 will be done retrospectively, the results of these tests will not be available to guide Step 2 drug selection. If ARV resistance testing is obtained outside of A5175, site investigators may use the results to guide the choice of Step 2 regimens.

**Step 3: Regimen Change for Participants Being Followed in Arm 1B (Receiving Any Regimen)**

**The purpose of Step 3 is to provide treatment options and collect follow-up data for participants followed on Arm 1B who did not meet the inclusion criteria for Step 2.**

**All participants followed on Arm 1B as of May 23, 2008 (date that the DSMB letters were distributed) were asked to return to the site as soon as possible.**

**If participants chose to remain in the study and continue to receive ARV therapy through the study, the study provided and will continue to provide co-formulated 3TC/ZDV, co-formulated FTC/TDF, EFV, and ATV from which to formulate an ARV regimen. If ATV is used together with TDF, RTV must be obtained from outside the study and used to boost ATV. The regimen of FTC (or 3TC) + ATV + ddI-EC will no longer be provided by the study. If participants chose to remain on this regimen, they must acquire these drugs from outside the study.**

**The following summarizes recommended study-provided options for participants on Arm 1B*:**

| **Viral Load** | **ARV Regimen** | **Recommended**  **Study-Provided Options** |
| --- | --- | --- |
| **<400 copies/mL** | **FTC (or 3TC) + ATV + ddI-EC** | **3TC/ZDV + EFV, OR**  **FTC/TDF + EFV and enroll to Step 3.** |
|  | **Any other regimen** | **Participant may continue the current regimen and enroll to Step 3.**  **Confer with the A5175 CMC if a regimen change is felt to be needed.** |
| **Confirmed**  **400–999 copies/mL** | **FTC (or 3TC) + ATV + ddI-EC** | **Confer with the A5175 CMC for selection of a Step 3 regimen.** |
|  | **Any other regimen** | **Confer with the A5175 CMC and enroll to Step 3.** |
| **Confirmed**  **>1000 copies/mL** | **FTC (or 3TC) + ATV + ddI-EC** | **Enter Step 2 after discussion with the A5175 CMC.** |
|  | **Any other regimen** | **Enter Step 2 after discussion with the A5175 CMC.** |

*** If a participant was receiving the regimen of FTC + ATV + ddI-EC in an arm other than Arm 1B, the A5175 CMC was to be consulted for management recommendations.**

**Participants will be followed on Step 3 for the duration of Arms 1A and 1C. Participants who entered Step 3 and later have virologic failure will stay on Step 3 and initiate another regimen change.**

**All participants on Arm 1B were to be encouraged to remain in study follow-up, regardless of whether or not they continued on a study-provided ARV regimen.**

## 4.0 SELECTION AND ENROLLMENT OF PARTICIPANTS

### 4.1 Step 1: Inclusion Criteria

4.1.1 HIV-1 infection, documented by a rapid HIV test or any licensed **enzyme-linked immunosorbent assay (ELISA)** test kit and confirmed by a repeat ELISA, Western blot, or plasma HIV-1 RNA at any time prior to study entry.

NOTE: The term "licensed" refers to an FDA-approved kit or, for sites located in countries other than the United States, a kit that has been certified or licensed by an oversight body within that country.

Confirmation of the initial test result must use a test that is different than the one used for the initial assessment. A reactive initial rapid test should be confirmed by either another rapid assay or an ELISA that is based on a different antigen preparation and/or different test principle (e.g., indirect versus competitive), or a Western blot or a plasma HIV-1 RNA. An initial ELISA result must be confirmed with another method such as a rapid test, Western blot or plasma HIV-1 RNA, but not by a repeat ELISA test.

4.1.2 ≤7 days of cumulative prior ARV therapy at any time prior to study entry.

NOTE: The use of single-dose NVP or ZDV for any period of time during pregnancy to prevent MTCT of HIV is allowed. Prior use of any two or more ARV drugs in combination for >7 days to prevent MTCT is not allowed.

4.1.3 CD4+ cell count <300 cells/mm^3^ obtained within 90 days prior to study entry at any Immunology Quality Assurance (IQA) or United Kingdom National External Quality Assurance Service (UKNEQAS) participating laboratory.

- - 1. The following laboratory values obtained within 45 days prior to study entry:
- Absolute neutrophil count (ANC) ≥750/mm^3^
- Hemoglobin ≥7.5 g/dL
- Platelet count ≥50,000/mm^3^
- Calculated creatinine clearance **(CrCl)** ≥60 mL/min

(See A5175 and A5185s MOPS for the Cockcroft and Gault method to calculate.)

- AST (SGOT), ALT (SGPT), and alkaline phosphatase ≤5 x ULN
- Total bilirubin ≤2.5 x ULN
  - 1. Female candidates of reproductive potential must have a negative serum or urine pregnancy result from a test with sensitivity of at least 50 mIU/mL, performed within 48 hours before entry.

NOTE: “Female candidates of reproductive potential” is defined as girls who have reached menarche or women who have not been post-menopausal for at least 24 consecutive months (i.e., who have had menses within the preceding 24 months) or have not undergone surgical sterilization (e.g., hysterectomy, or bilateral oophorectomy, or bilateral salpingotomy or tubal ligation).

- - 1. Contraception requirements

4.1.6.1 Female Candidates of Reproductive Potential

Female candidates of reproductive potential, who are participating in sexual activity that could lead to pregnancy, must agree to the following.

- If the regimen does not include EFV, they must agree to use at least one reliable method of contraception while receiving the protocol-specified drugs and for 6 weeks after stopping the medications.
- If the regimen includes EFV, they must agree to use two reliable methods of contraception: a barrier method of contraception (e.g., condoms, diaphragm, or cervical cap with or without spermicide) together with another reliable form of contraception (e.g., a second barrier method, an IUD, or a hormone-based contraceptive) while receiving EFV and for **12** weeks after stopping EFV. (NVP, or another ARV drug, will be substituted for EFV, if candidates are not able or not willing to use two concurrent forms of contraception.)

4.1.6.2 Female Candidates Who Are Not of Reproductive Potential

- If the regimen does not include EFV, women who are not of reproductive potential or whose male partner(s) has azoospermia are eligible to start study drugs without requiring the use of contraceptives.

NOTE: Acceptable documentation of lack of reproductive potential for a non-EFV-containing regimen is the woman’s self-reported history of surgical sterilization, menopause, or male partner’s azoospermia. Any statement of self-reported sterility or that of her partner’s must be entered in the source documents.

- If the regimen includes EFV, women are eligible without requiring the use of contraceptives, only if there is written documentation or oral communication of lack of reproductive potential from a clinician or clinician’s staff documented in source documents of one of the following: physician report/letter, discharge summary, or FSH measurement elevated into the menopausal range, as established by the reporting laboratory.

NOTE: If written documentation is not obtainable, the woman taking EFV must agree to use at least one barrier method of contraception with a possible second method required at the discretion of the site **investigator**.

4.1.7 Candidate does not intend to relocate away from current geographical area of residence for the duration of study participation.

4.1.8 Candidate is willing to adhere to the on-study follow-up schedule.

4.1.9 Men and women age ≥18 years.

4.1.10 Karnofsky performance score ≥70 within 45 days prior to study entry.

4.1.11 Ability and willingness of candidate to provide written informed consent, including whether permission is given to draw and store extra blood specimens.

4.1.12 Plasma HIV-1 RNA quantitation using the Roche Amplicor HIV-1 Monitor Assay within 45 days prior to study entry at any DAIDS Virology Quality Assurance (VQA) participating laboratory.

### 4.2 Step 1: Exclusion Criteria

4.2.1 Acute therapy for serious medical illnesses, in the opinion of the site investigator, within 14 days prior to study entry. Candidates with chronic, acute, or recurrent infections that are serious, in the opinion of the site investigator, who must continue with chronic (maintenance) therapy (e.g., TB), must have completed at least 14 days of therapy prior to study entry and be clinically stable (**s**ee the A5175 and A5185s MOPS)**.**

NOTE: Oral and vaginal candidiasis, mucocutaneous herpes simplex, and other minor illnesses **that,** in the opinion of the site investigator**,** present no restriction to eligibility, **are not exclusionary.**

4.2.2 Any condition that, in the opinion of the site investigator, would compromise the candidate’s ability to participate in the study.

4.2.3 Radiation therapy or systemic chemotherapy within 45 days prior to study entry.

NOTE: Systemic chemotherapy for treatment of Kaposi sarcoma during the 45 days prior to study entry is acceptable provided the systemic chemotherapy is completed prior to entry. Anticipated need for systemic chemotherapy while on study is not permitted.

4.2.4 Any immunomodulator, HIV vaccine, or other investigational therapy within 30 days prior to study entry.

NOTE: A tapering course of corticosteroids as acute therapy for Pneumocystis carinii pneumonia (PCP) or other conditions is an exception. Antipneumocystic treatment should meet the requirement of section 4.2.1.

4.2.5 Active drug or alcohol use or dependence that, in the opinion of the site investigator, would interfere with adherence to study requirements.

4.2.6 Pancreatitis within 3 years prior to study entry.

- - 1. Pregnancy.

NOTE: Breast-feeding is not a restriction on eligibility.

4.2.8 Allergy/sensitivity to any study drugs or their formulations.

4.2.9 Heart rate <40 bpm or a history of untreated active 2^nd^ or 3^rd^ degree heart block. Candidates who have symptoms potentially related to heart block (e.g., unexplained dizziness, syncope, palpitations, or dyspnea) within 45 days prior to study entry, will undergo clinical and electrocardiogram (EKG) evaluation. If the clinical evaluation or the EKG results indicates a cardiac cause of the symptoms, then the candidate is ineligible.

4.2.10 Vomiting or inability to swallow medications due to an active, pre-existing condition that prevents adequate swallowing and absorption of study medication.

4.2.11 Need for a prohibited medication listed in section 5.4.2.

4.2.12 Compulsorily detained (involuntarily incarcerated) in a correctional facility, prison, or jail or for treatment of either a psychiatric or physical (e.g., infectious disease) illness.

### 4.3 Step 2: Inclusion Criteria

4.3.1 Virologic failure defined as two successive measurements of plasma HIV-1 RNA ≥1000 copies/mL with the first measurement at the week 16 visit or later on Step 1; either AIDS progression after at least 12 weeks following randomization or a decrease in **CD4+ cell counts** to <50% of the Step 1 maximum.

or

Virologic failure defined as two successive measurements of plasma HIV-1 RNA ≥1000 copies/mL **(**with the first measurement at the week 16 visit or later on Step 1**)**, **plus** **a recommendation from the** site investigator **to initiate** a new regimen because of decreased potency of the initial Step 1 regimen, in the absence of **both** **AIDS progression** after at least 12 weeks following randomization and a decrease in CD4+ cell counts to <50% of the Step 1 maximum.

or

Either AIDS progression after at least 12 weeks following randomization or a decrease in **CD4+ cell counts** to <50% of the Step 1 maximum in the absence of confirmed virologic failure; and initiation of a new regimen is recommended by the site investigator because of decreased potency of the Step 1 regimen.

NOTE **A**: Minimum and maximum **CD4+ cell counts** will each be the mean of two consecutive measurements.

**NOTE B: AIDS progression is defined as a new or recurrent AIDS diagnosis as defined in the list of AIDS-defining opportunistic infections and malignancies provided in the A5175 and A5185s MOPS located on the PSWP.**

4.3.2 Participant has been counseled on ARV treatment adherence.

4.3.3 Participant is willing to switch to a new ARV regimen.

4.3.4 The site investigator recommends a new ARV regimen for management of presumed and/or documented ARV drug resistance.

4.3.5 Site personnel have contacted the A5175 CMC and received approval for the participant to proceed to Step 2.

### 4.4 Step 2: Exclusion Criteria

None

### 4.5 Step 3: Inclusion Criteria

**4.5.1 Randomized to Arm 1B and followed on study as of May 23, 2008 (when DSMB letters stopping Arm 1B were distributed).**

**4.5.2 Availability of plasma HIV-1 RNA result between November 23, 2007 and May 23, 2008.**

**NOTE: If the most recent RNA within this timeframe was <400 copies/mL, then the CMC did not need to be consulted regarding the Step 3 ARV regimen. If the most recent HIV-1 RNA was between 400–999 copies/mL and was confirmed within this range, then the CMC had to be consulted regarding a regimen change. If the RNA was >1000 copies/mL, then Step 2 eligibility had to be assessed.**

### 4.6 Step 3: Exclusion Criteria

None

### 4.7 A5185s Genital Secretions Substudy of A5175: Inclusion and Exclusion Criteria

Participants must be enrolled in A5175. Enrollment in A5185s occurs at the time of enrollment to A5175, Step 1 (see Appendix VI). The A5185s entry evaluations will occur prior to the initiation of A5175 study treatment at Step 1 study entry, at Step 2 entry**, and** **at Step 3 entry.**

**4.7.1** Inclusion Criteria

**4.7.1.1** Enrollment in A5175.

**4.7.1.2** A signed A5185s informed consent form.

**4.7.2** Exclusion Criteria

None

### 4.8 Study Enrollment Procedures

Prior to implementation of this protocol, **and any subsequent full version amendments**, **each site** must have the protocol and consent form(s) approved, **as appropriate**, by their local institutional review board (IRB). Protocol registration must occur before **the site can enroll any** participants into the study.

Once a candidate for study entry has been identified, details will be carefully discussed with the individual. The individual will be asked to read and sign the consent form that was approved by both the site’s IRB and the DAIDS RCC Protocol Registration Office.

**4.8.1** Two-Stage Enrollment and Allocation of Participant Spaces at **U.S. and non-U.S. CRSs**

4.8.1.1 Stage I Pilot Enrollment

**Non-U.S. CRSs**

A5175 is the first ACTG clinical trial to be conducted at most of the **non-U.S. CRSs.** Therefore, A5175 will open in a two-stage manner designed to identify and correct any deficiencies in good clinical practice (GCP) and laboratory standardization that may exist at **non-U.S. CRSs.**

After a non-U.S. CRS is registered to the protocol with the DAIDS RCC Protocol Registration Office, the site will be allocated spaces for 10 participants. Once 10 participants begin treatment at a site, further enrollment at that site will be stopped until site monitors under contract to the National Institute of Allergy and Infectious Diseases (NIAID) certify the site for further enrollment. This certification will not

commence until the 10^th^ enrolled participant has completed week 4 on study. If the site does not meet the requirements of Stage I, deficiencies must be corrected before qualifying to participate in Stage II. (Note: All non-U.S. CRSs were certified as of 5/6/2006.)

Each **non-U.S. CRS** may enroll a maximum of 10 participants in Stage I (subunit enrollment is included in the 10 spaces allotted for each main unit). All **non-U.S. CRSs** are expected to complete Stage I within 16 weeks after the protocol opens to accrual at that site.

**U.S. CRSs**

Each **U.S. CRS** may enroll up to 10 participants in Stage I (subunit enrollment is included in the 10 spaces allotted for each main unit). Once the 10 participants initiate study treatment, further enrollment will be stopped until Stage II commences.

**4.8.1.2** Stage II Open Enrollment

Stage II will commence for **U.S. and eligible non-U.S. CRSs** when six **non-U.S. CRSs** have successfully met Stage I certification requirements (Note: Stage II commenced on 12/19/2005).

If **non-U.S. CRSs** wish to enroll more than their initial allocation of 90 participant spaces, the **non-U.S. CRS** investigator must request permission from the protocol co-chairs to do so.

If any **non-U.S. CRS** does not utilize its Stage II allocated spaces, the remaining balance of enrollment spaces at that site may be reallocated by the protocol co-chairs to other **non-U.S. CRSs** that have utilized their Stage II allocated spaces.

There is no predetermined number of participant spaces allocated to individual **U.S. CRSs**; however, all **U.S. CRSs** will collectively enroll no more than 270 participants in order to reserve a minimum of 1250 spaces for **non-U.S. CRSs**. **U.S. CRSs** may enroll until notified by the A5175 CMC that enrollment of participants at **U.S. CRSs** is closed. **U.S. CRSs** will be given advance notice before enrollment is closed.

- - 1. At entry, participants will be randomized to one of three arms in Step 1. Sites will randomize participants according to standard ACTG Data Management Center (DMC) procedures.
    2. Participants who meet the requirements for Step 2 will be registered to Step 2 according to standard ACTG DMC procedures.
    3. Whether individual HBV/HIV coinfected persons are enrolled in A5175 will be left to the discretion of the site investigator. If treatment of HIV-1 infection with **ARV** regimens that include 3TC or FTC but without TDF is not consistent with the local standard of care, site investigators may elect to not enroll HBV/HIV coinfected individuals in A5175.
    4. **Participants who meet the requirements for Step 3 will be registered to Step 3 according to standard ACTG DMC procedures. A new study identification number (SID) will be assigned.**

### 4.9 Coenrollment

Coenrollment in ACTG A5128**, A5243,** A5199, **and A5271** is permitted. Coenrollment in A5001 and A5164 is not permitted.

Coenrollment into other studies will be considered on an individual basis. For specific questions and approval regarding coenrollment, contact the A5175 CMC by e-mail as described in the Study Management Section of this protocol.

## 5.0 STUDY TREATMENT

The ARV study drugs used in A5175 are 3TC/ZDV, FTC, TDF, FTC/TDF, EFV, ATV, ddI-EC, NVP, ZDV, 3TC, and d4T. All study drugs will be provided as part of the study to both **non-U.S.** and **U.S. CRSs** for the duration of the study. Listed below are the study regimens and study-allowed drug substitutions.

NOTE **A**: Participants at **U.S. CRSs** receiving 3TC/ZDV, 3TC, and ZDV via non-study prescription under Protocol Version 1.0 may switch to study-provided 3TC/ZDV, 3TC, and ZDV after the **U.S. CRS** has completed protocol registration for Version 2.0.

**NOTE B: The WHO Addendum to Guidelines on Antiretroviral Therapy for HIV Infection in Adults and Adolescents, 2006, recommends a stavudine (d4T) dose of 30 mg BID for all adult and adolescent patients regardless of body weight. While this is currently an off-label dosing recommendation for d4T, the new WHO Guidelines have been implemented in many international settings, including countries where A5175 is conducted. Since the A5175 protocol follows the current U.S. DHHS d4T dosage recommendations of 40 mg BID for patients weighing ≥60 kg and 30 mg BID for patients weighing <60 kg, the current A5175 protocol conflicts with the dosing used in practice in countries that follow the WHO Guidelines. Therefore, the protocol will allow sites to follow either the WHO or DHHS guidelines, at the discretion of the site investigator.**

### 5.1 Regimens, Administration, and Duration

- - 1. Regimens

5.1.1.1 Step 1: Initial Study Treatment Regimen

At entry, participants will be randomized (1:1:1) to one of the following three study treatment arms.

| Arm | Initial Step 1 Study Treatment Regimen |
| --- | --- |
| 1A | 3TC/ZDV 150 mg/300 mg PO BID + EFV 600 mg PO QHS |
| 1B | FTC 200 mg PO QD + ATV 400 mg PO QD + ddI-EC 400 mg PO QD for participants who weigh ≥60 kg or 250 mg PO QD for participants who weigh <60 kg |
| 1C | FTC/TDF 200 mg/300 mg PO QHS + EFV 600 mg PO QHS |

NOTE A: The drugs in Arm 1C are to be taken **QD**. It is recommended that they be taken at bedtime, though this is not required. If NVP is substituted for EFV in Arm 1C. Follow dosing instructions in Table 2.

NOTE B: Women who are randomized to EFV and who are both of reproductive potential and not willing to use two forms of contraceptives, will use either NVP (if entry CD4+ cell count is ≤250/mm^3^) or another ARV drug as shown in Table 2 below (if entry CD4+ cell count is >250/mm^3^) rather than EFV, at the discretion of the site investigator.

Women who receive EFV and who are required to use two methods of acceptable birth control will be asked if they are using two methods of birth control at every visit, starting at the week 2 visit. If a woman reports she is not using two acceptable methods of birth control, EFV will be replaced with another ARV drug. Record the drug substitution and reason in the source documents and record the drug substitution on the CRF.

NOTE C: Clinicians initiating participants on ZDV-containing regimens should be alert to clinical signs of anemia and neutropenia and consider closer monitoring of participants with risk factors at screening, such as female gender, low body mass index, low CD4+ **cell** count (and/or high HIV-1 RNA plasma viral load) and/or concurrent bacterial infections or parasitic infestations. Health care workers need to be vigilant, and participants should receive education or counseling about the symptoms of anemia.

Participants who have Grade 1 or 2 anemia and/or neutropenia at screening should be evaluated for causes of anemia and/or neutropenia, such as concurrent bacterial, fungal or mycobacterial infection, malaria, helminthiasis, malignancy and/or malnutrition, and treatment commenced prior to starting ZDV, **if possible.**

Participants with Grade 1 anemia at screening who begin study treatment with ZDV should be monitored closely for symptoms, signs, and laboratory evidence of worsened anemia during the first 2 months of ZDV treatment as deemed appropriate by the site investigator.

Participants with Grade 2 anemia and/or neutropenia at screening should have a reduction of the ZDV dose or substitution of d4T for ZDV. If alternative causes for Grade 2 anemia cannot be identified, then a transfusion should be considered prior to starting ZDV. If ZDV is dose reduced, the hematology panel should be monitored closely until abnormal values reach Grade 1. If participants with Grade 2 anemia and/or neutropenia at screening have d4T substituted for ZDV at study entry and anemia and/or neutropenia improves to Grade ≤1, ZDV may then be substituted for d4T, at the discretion of the site investigator.

NOTE D: HBV-HIV-1 coinfected participants who receive only one study-provided, anti-HBV agent may **receive entecavir** **(**provided outside of the study**)** for the treatment of HBV infection, at the discretion of the site investigator.

**NOTE E: Participants receiving FTC + TDF in Arm 1C under protocol Version 1.0 may switch** **to FTC/TDF fixed-dose combination tablet (Truvada) once their site is registered to protocol Version 2.0. A new prescription must be written in order for the pharmacist to dispense FTC/TDF under Version 2.0.**

Table 2. Medication and Dosing

| Medication | Dose | Frequency | Notes |
| --- | --- | --- | --- |
| Lamivudine/  Zidovudine  3TC/ZDV  Combivir | 150 mg/  300 mg | 1 PO BID with or without food | As appropriate, ZDV will be administered individually, with or without food, if dose reduction or substitution of ZDV is required. |
| Efavirenz  EFV  Sustiva | 600 mg | 1 PO QHS without food | Participants should be informed that if they experience CNS symptoms they should avoid potentially hazardous tasks such as driving or operating machinery; and they should be informed that CNS symptoms are likely to improve with continued therapy. Dosing at bedtime improves the tolerability of these symptoms and is recommended during the first weeks of therapy and for participants who continue to experience CNS symptoms. Those receiving EFV should be alerted to the potential for additive CNS effects when EFV is used concomitantly with alcohol or psychoactive drugs. |
| Zidovudine  ZDV  Retrovir | 300 mg | 1 PO BID  with or without food |  |

Table 2. Medication and Dosing ((Cont'd)

| Medication | Dose | Frequency | Notes |
| --- | --- | --- | --- |
| Lamivudine  3TC  Epivir | 300 mg | **1 PO** QD with or without food |  |
| Didanosine  ddI-EC  Videx | 400 mg  250 mg | 1 PO QD at least 1 hour before or 2 hours after a meal | 400 mg if weight ≥60 kg  250 mg if weight <60 kg  Should be taken on an empty stomach, i.e., at least 1 hour before or 2 hours after a meal.  ddI-EC should be taken on an empty stomach 1 hour before or 2 hours after ATV is taken with food.  If given with TDF reduce dose of ddI-EC (see section 5.4.3). |
| Tenofovir  Disoproxil  Fumarate  TDF  Viread | 300 mg | 1 PO QD with or without food |  |
| Emtricitabine  FTC  Emtriva | 200 mg capsules | 1 PO QD with or without food |  |
| Emtricitabine/  Tenofovir  Disoproxil Fumarate  FTC/TDF  Truvada | 200mg/  300mg tablets | 1 PO QD with or without food |  |
| Atazanavir  ATV  Reyataz | 400 mg  300 mg | 2 PO QD with light meal or snack | ddI-EC should be taken on an empty stomach 1 hour before or 2 hours after ATV is taken with food.  **If ATV and TDF are used together, ATV should be boosted with RTV.** |
| Nevirapine  NVP  Viramune | 200 mg for 14 days, then 400 mg | 1 PO QD for first 14 days (lead-in),  then 1 PO BID thereafter with or without food. | The drugs in Step 1, Arm 1C are to be taken QD at bedtime, if possible. However, if NVP is substituted for EFV in Arm 1C, it should be started with the lead-in of 200 mg PO QD for 14 days; then 200 mg PO BID. If the person tolerates NVP for ≥ 3 months, the dose may be changed to 400 mg PO QD. It is recommended that the QD dose be taken at bedtime, although this is not required.  Health care providers must review signs and symptoms of NVP-related hypersensitivity and hepatitis with the participant prior to dispensing NVP. Participants should contact their site physician if they develop rash or signs and symptoms of hypersensitivity or hepatitis. If rash occurs during lead-in, do not increase dose until the rash has resolved. **The lead-in dosing period should not exceed 28 days.** After reaching full dose, if NVP dosing is interrupted for >7 days, **then** NVP should be started with the lead-in of 200 mg PO QD for 14 days, **followed by** 200 mg PO BID. |
| Stavudine  d4T  Zerit | 30 mg  40 mg | 1 PO BID  with or without food | d4T 40 mg will be administered orally BID for participants weighing **≥**60 kg or as 30 mg BID for those weighing **<**60 kg, with or without food**. At the discretion of the site investigator, d4T 30 mg may be administered orally BID with or without food.** |

Drug Substitutions

Drug substitutions within initial study treatment regimens are allowed as indicated in Table 3 below and per section 7.5.16. All drug substitutions in Step 1 (except for substitution of NVP for EFV in women of childbearing potential) must be pre-approved by the A5175 CMC ([actg.cmca5175@fstrf.org](mailto:actg.cmca5175@fstrf.org)).

Table 3. Permitted Step 1 Study Treatment Regimen Drug Substitutions (follow dosing instructions in Table 2)

| Arm | If There is Toxicity or Contraindication to: |
| --- | --- |
| 1A | - ZDV, then d4T may be substituted - ZDV or d4T, then TDF may be substituted for either drug - EFV, then NVP may be substituted - Both EFV and NVP, then ATV may be substituted for EFV |
| 1B | - ATV, then either NVP or EFV may be substituted - ddI-EC, then either TDF or d4T may be substituted |
| 1C | - TDF, then ddI-EC may be substituted - Both TDF and ddI-EC, then d4T may be substituted for TDF - EFV, then NVP may be substituted - Both EFV and NVP, then ATV may be substituted for EFV if RTV is available |

- Women who become pregnant on study may need to change their ARV drugs (see section 7.6, Procedures in the Event of On-Study Pregnancy and Breast-Feeding, for indications).
- The use of d4T is recommended as a substitution drug for ZDV. However, TDF may be used instead of d4T, at the discretion of the site investigator.
- If it is necessary to administer ATV and TDF **together, RTV** must be used to “boost” ATV for **PK** effects (RTV is not provided by the study). If generic RTV is used, it should be approved by local, within-country authorities.

5.1.1.2 Step 2 Regimen

Participants who fail a Step 1 regimen as defined in section 3.0 may initiate a new Step 2 regimen of three or more ARV drugs chosen by the site investigator from study drugs available in Step 1 and/or non-study drugs not available through A5175 as follows.

| Arm | Regimen |
| --- | --- |
| 2A  (follows 1A regimen failure) | 2 NRTIs + PI(s) |
| 2B  (follows 1B regimen failure) | 2 NRTIs + EFV |
| 2C  (follows 1C regimen failure) | 2 NRTIs + PI(s) |

- Choice of specific NRTIs or PI(s) in Step 2 is at the discretion of the site investigator. More than one PI given at a therapeutic dose may be used in Step 2. RTV must be used to “boost” ATV for **PK** effects when it is administered with TDF (RTV is not provided by the study).
- A minimum of two NRTIs is required in Step 2. Since Step 2 treatment options are limited and 3TC or FTC may provide continued benefit despite the presence of high-level resistance, 3TC or FTC will be allowed as a third NRTI in Step 2 regimens when potentially viable two-NRTI combinations are not available, at the discretion of the site investigator.
- It is recommended that study-specified and provided ARV agents be utilized. If needed, generic agents that are or become approved or tentatively-approved by the U.S. FDA are preferred though not required. If utilized, generic agents should be approved by local, within-country authorities.
- Sites are required to request permission from the A5175 CMC for participants to register to Step 2.
- Although A5175 CMC pre-approval of Step 2 drug substitutions is not required, **non-U.S.** and **U.S. CRS**s may contact the A5175 CMC to discuss substitutions as needed.
- Genotyping performed outside of the study is permitted to inform drug selection for both **non-U.S. and U.S. CRSs**.

**5.1.1.3 Step 3 Regimen**

**The following summarizes recommended study-provided options for participants on Arm 1B*:**

| **Viral Load** | **ARV Regimen** | **Recommended**  **Study-Provided Options** |
| --- | --- | --- |
| **<400 copies/mL** | **FTC (or 3TC) + ATV + ddI-EC** | **3TC/ZDV + EFV, OR**  **FTC/TDF + EFV and enroll in Step 3.** |
|  | **Any other regimen** | **Participant may continue the current regimen and enroll in Step 3.**  **Confer with the A5175 CMC if a regimen change is felt to be needed.** |
| **Confirmed**  **400–999 copies/mL** | **FTC (or 3TC) + ATV + ddI-EC** | **Confer with the A5175 CMC for selection of a Step 3 regimen.** |
|  | **Any other regimen** | **Confer with the A5175 CMC and enroll in Step 3.** |
| **Confirmed**  **>1000 copies/mL** | **FTC (or 3TC) + ATV + ddI-EC** | **Enter Step 2 after discussion with the A5175 CMC.** |
|  | **Any other regimen** | **Enter Step 2 after discussion with the A5175 CMC.** |

*** If a participant was receiving the regimen of FTC + ATV + ddI-EC in an arm other than Arm 1B, the A5175 CMC was to be consulted for management recommendations.**

**If participants chose to remain in the study and continue to receive ART through the study, the study provided and will continue to provide co-formulated 3TC/ZDV, co-formulated FTC/TDF, EFV, and ATV. If ATV is used together with TDF, RTV must be obtained from outside the study and used to boost ATV. Participants will be registered to Step 3. A new SID will be assigned. The pharmacist must receive a new prescription with the new SID in order to dispense the Step 3 regimen.**

**The regimen of FTC (or 3TC) + ATV + ddI-EC will no longer be provided by the study. If participants choose to remain on this regimen, they will need to acquire these drugs from outside the study.**

### 5.2 Drug Formulation

Table 4. Drug Formulations and Storage

| Medication | Formulation | Storage |
| --- | --- | --- |
| Lamivudine/  Zidovudine  3TC/ZDV  Combivir | 150 mg/  300 mg tablet | Store at 2°-30°C (36°-86°F). |
| Efavirenz  EFV  Sustiva | 600 mg tablets | Store at 25°C (77°F). Excursions permitted between 15° and 30°C (59° and 86°F). |
| Zidovudine  ZDV  Retrovir | 100 mg capsules  300 mg tablets | Store at 15°-25°C (59° and 77°F). Capsules should be protected from moisture. |
| Lamivudine  3TC  Epivir | 150 mg tablets  300 mg tablets | Store at 25ºC (77°F); excursions permitted to 15º-30ºC (59º and 86ºF) |
| Tenofovir  Disoproxil  Fumarate  TDF  Viread | 300 mg tablets | Tablets should be stored and dispensed in the original container. Each bottle contains a silica gel desiccant canister to protect the product from humidity, and this should remain in the container. TDF should be stored at 25°C (77°F). Excursions permitted between 15° and 30°C (59° and 86°F). |
| Emtricitabine  FTC  Emtriva | 200 mg capsules | Store at 25°C (77°F), Excursions permitted between 15°-30°C (59°and 86°F). |
| Emtricitabine/  Tenofovir  Disoproxil Fumarate  FTC/TDF  Truvada | 200 mg/300 mg tablets | Store at 25°C (77°F); excursions permitted between 15°-30°C (59° and 86°F). Keep container tightly closed. Dispense only in original container. |
| Atazanavir  ATV  Reyataz | 200 mg capsules  150 mg capsules | Store at 25°C (77°F). Excursions permitted between 15°-30°C (59° and 86°F). |
| Didanosine  ddI-EC  Videx | 400 mg capsules  250 mg capsules  200 mg capsules  125 mg capsules | Store between 15° and 30°C (59° and 86°F). |
| Nevirapine  NVP  Viramune | 200 mg tablets | Store at 25° (77°F). Excursions permitted between 15° and 30°C (59° and 86°F). |
| Stavudine  d4T  Zerit | 15 mg, 20 mg, 30 mg, and 40 mg capsules | Store between 15° and 30°C (59° and 86°F). |

Sufficient study medications will be dispensed at each visit to last beyond the next scheduled study visit.

### 5.3 Product Supply, Distribution, and Accountability

- - 1. Product Supply

The following ARV study drugs will be provided to both **non-U.S.** **and U.S. sites**: TDF, FTC, FTC/TDF, NVP, ATV, EFV, d4T, ddI-EC, 3TC/ZDV, ZDV, and 3TC.

All other ARV drugs will be provided by non-study prescription.

NOTE: **Non-U.S. sites** have developed plans for the continuation of ARV therapy after study completion.

- - 1. Study Product Acquisition/**Distribution**

ARV study drugs will be available through the NIAID Clinical Research Products Management Center (CRPMC). **Site** pharmacists can obtain A5175 study agents by following the instructions in the *Pharmacy Guidelines and Instructions for DAIDS Clinical Trials Networks* manual, Study Product **Management Responsibilities**.

5.3.3 Study Product Accountability

**CRS** pharmacists are required to maintain complete records of all study products received from the NIAID CRPMC and subsequently dispensed. All unused study products must be returned to the NIAID CRPMC **(or as otherwise directed by the sponsor)** after the study is completed or terminated. The procedures to be followed are provided in the manual, *Pharmacy Guidelines and Instructions for DAIDS Clinical Trials Networks*, Study Product **Management Responsibilities**. **Non-U.S. CRS** pharmacists **must follow the instructions in the *Pharmacy Guidelines and Instructions for DAIDS Clinical Trials Networks* for the destruction of unused study products.**

### 5.4 Concomitant Medications

To avoid drug interactions and AEs, refer to the most recent package inserts of ARV and concomitant agents whenever a concomitant medication is initiated or dose is changed.

- - 1. Required Medications

No concomitant medications are required. Medications recommended for prophylaxis of AIDS-defining conditions are listed in the A5175 and A5185s MOPS.

5.4.2 Prohibited Medications

**The A5175 prohibited medications are listed on the A5175 PSWP.**

**To avoid drug interactions and AEs, refer to the most recent package inserts of ARV and concomitant agents whenever a concomitant medication is initiated or dose is changed.**

5.4.3 Precautionary Medications

**The A5175 precautionary medications are listed on the A5175 PSWP.**

To avoid drug interactions and AEs, refer to the most recent package inserts of ARV and concomitant agents whenever a concomitant medication is initiated or dose is changed.

### 5.5 Adherence to Study Treatment

See **s**ection 6.0 and Appendix IV for adherence procedures.

## 6.0 CLINICAL AND LABORATORY EVALUATIONS

### 6.1 Schedule of Events (SOE)

| Evaluation | Screening  (-7 to -45 days) | Step 1  Entry | Weeks Since Start of Regimen Initiated at  Step 1, 2, **or 3** Entry | | | | | | | | | | | Repeat On-Step Evals Step 1, 2, **or 3**^1^ | Confirm Virologic Failure:  Step 1, 2, **or 3** | Step 2 **or 3** Entry | AIDS OI or Increased LFTs  on Step 1, 2, **or 3^2^** | | Premature Rx Discontinuation  (Off Rx-On Study) | | | | Final Visit/ Off Study |
| --- | --- | --- | --- | --- | --- | --- | --- | --- | --- | --- | --- | --- | --- | --- | --- | --- | --- | --- | --- | --- | --- | --- | --- |
|  |  |  | 2 | 4 | 6 | 8 | 12 | 16 | 20 | 24 | 32 | 40 | 48 |  |  |  |  | | Primary Endpoint  Not Met^3^ | | Primary Endpoint Met^4^ | |  |
| Documentation of HIV | X |  |  |  |  |  |  |  |  |  |  |  |  |  |  |  |  | |  | |  | |  |
| Medical/Medication History | X |  |  |  |  |  |  |  |  |  |  |  |  |  |  |  |  | |  | |  | |  |
| Race/Ethnicity | X |  |  |  |  |  |  |  |  |  |  |  |  |  |  |  |  | |  | |  | |  |
| Concomitant Medications |  | X | X | X |  | X | X | X | X | X | X | X | X | Q8w |  | X | X | | X | | X | | X |
| Complete/Targeted Physical Exam | X^5^ | X | X | X |  | X | X | X | X | X | X | X | X | Q8w |  | X | X | | X | | X Q12w | | X |
| Karnofsky Performance | X |  |  |  |  |  |  |  |  |  |  |  |  |  |  |  |  | |  | |  | |  |
| Hematology | X | X | X | X |  | X | X | X | X | X | X | X | X | Q8w |  | X |  | | X | | X Q12w | | X |
| Liver Function Tests^6^ | X | X | X | X | X^6^ | X | X | X | X | X | X | X | X | Q8w |  | X |  | | X | | X Q12w | | X |
| Serum Chemistries | X | X | X | X |  | X | X | X | X | X | X | X | X | Q8w |  | X |  | | X | | X Q12w | | X |
| Lactate |  |  | Perform for symptoms suggestive of lactic acidosis | | | | | | | | | | | |  |  |  | |  | |  | |  |
| Lipase + Triglycerides |  | X^7^ | Perform for symptoms suggestive of lactic acidosis | | | | | | | | | | | |  |  |  | |  | |  | |  |
| Creatine Kinase (CK) |  |  | Perform for symptoms suggestive of lactic myopathy | | | | | | | | | | | |  |  |  | |  | |  | |  |
| Pregnancy Testing | X | X |  | X |  | X | X | X | X | X | X | X | X | Q8w |  | X |  | |  | |  | |  |
| Urinalysis |  | X |  |  |  |  |  |  |  | X |  |  | X | Q24w |  | X |  | |  | |  | |  |
| HBsAg | X |  |  |  |  |  |  |  |  |  |  |  |  |  |  |  |  | |  | |  | |  |
| EKG^8^ | X |  | Whenever clinically indicated for participants receiving ATV | | | | | | | | | | | |  |  |  | |  | |  | |  |
| CD4+ Cell Count | X^9^ |  |  | X |  | X |  | X |  | X | X | X | X | Q8w |  | X | X | | X | | X Q12w | | X |
| HIV-1 RNA (real time) | X | X |  | X |  | X |  | X |  | X | X | X | X | Q8w | X | X | X | | X | | X Q12w | | X |
| Drug Resistance Testing |  | X^10^ |  |  |  |  |  |  |  |  |  |  |  |  | X^3^ | X^3^ |  | | X^3^ | |  | | X^3^ |
| Random EFV, NVP, TDF, ATV Blood Levels |  |  |  | X^11^ |  |  |  |  |  |  |  |  |  |  |  |  |  | |  | |  | |  |
| Trough ATV Blood Level |  | After at least 14 days of concurrent ATV and Rifabutin use | | | | | | | | | | | | |  | | | | | | | | |
| Quality of Life/Psychosocial Interview |  | X |  |  |  |  |  | X |  |  | X |  | X | Q48w |  | X |  | | X | | X Q48w | |  |
| Adherence Interview |  |  | X | X |  | X | X | X | X | X | X | X | X | Q8w | X | X | X | | X | |  | | X |
| Adherence and Sexual Risk Reduction Counseling |  | X | As needed at the discretion of the site investigator | | | | | | | | | | | | | | |  | |  | |  | |
| Pill Counts (Step 1 only) |  | X |  | X |  | X | X | X | X | X | X | X | X | Q8w | X |  | X | | X | | X | | X |
| Anthropometric Measures |  | X |  |  |  |  |  | X |  |  | X |  | X | Q16w^3^ |  | X |  | |  | |  | |  |
| Stored serum & plasma for protocol-specified research |  | X |  |  |  |  |  |  |  | X |  |  | X | Q24w | X | X | X | | X | | X Q24w | | X |
| Stored serum & plasma for future ACTG research^12^ |  |  |  |  |  | X |  | X |  |  |  |  |  |  |  |  |  | |  | |  | |  |
| Stored PBMC for future ACTG research^12^ |  | X |  |  |  |  |  |  |  |  |  |  |  |  | X | X | X | | X | | X | | X |
| A5185s Genital Secretions |  | X^13^ |  |  |  |  |  |  |  |  |  |  | X | Q48w |  | X |  | | X (Off Study) | |  | | X |

^1^ Repeat post 48-week evaluations on Step 1, 2, **or 3** as indicated.

^2^ Perform as soon as possible after diagnosis of a new AIDS OI or new Grade ≥ 3 ALT (SGPT) or AST (SGOT). PBMC DNA, Adherence Interview, Adherence and Sexual Risk Reduction Counseling, and Pill Counts, are not required for a new Grade ≥ 3 ALT (SGPT) or AST (SGOT).

^3^ For participants who prematurely discontinue study treatment + have NOT reached a primary study endpoint, continue evaluations per Step 1, 2, **or 3** as appropriate. See section 9.2.

^4^ For participants who prematurely discontinue study treatment + reach a primary study endpoint, obtain indicated evaluations and follow-up evaluations on the SOE as indicated. See section 9.2.

^5^ Complete physical examination required at screening only.

^6^ Obtain LFTs at week 2, 4, 6, and 8 after any time NVP is initiated, and then monthly through the 20^th^ week of NVP treatment. Participants not initiating NVP at entry need not present for a week 6 visit.

^7^ Obtain baseline lipase/triglycerides at any initiation of ddI-EC on either Step 1 or Step 2 entry, or at any time ddI-EC is substituted for another drug.

^8^ Perform at screening only for participants who have symptoms potentially related to heart block (e.g., unexplained dizziness, syncope, palpitations or dyspnea) within 45 days prior to study entry.

^9^ CD4+ cell count for determining study eligibility must be obtained within 90 days prior to study entry.

^10^ Stored samples for resistance testing are in addition to those that are stored for future studies.

^11^ Step 1 only: If evaluation at week 4 is not possible, perform after week 4 but no later than week 8.

^12^ **Plasma and sera for future ACTG research is collected and stored at weeks 8 and 16. In addition, any plasma or sera collected and stored at the points designated for protocol-specified research may also be used for future ACTG research if the study participant is taking part in sample storage for future ACTG research.** PBMC**, plasma, and sera** preparation and storage for future ACTG research will not be performed at sites that do not have this capability or at sites that are not able to store specimens for future unspecified research.

^13^ Collect prior to initiation of study treatment.

### 6.2 Timing of Evaluations

6.2.1 Prerandomization Evaluations

Occur prior to the participant taking any study medications, treatments, or interventions.

Screening

Screening evaluations to determine eligibility must be completed within 7 to 45 days prior to study entry; the exception is CD4+ cell count, which must be completed within 90 days prior to study entry.

6.2.2 Randomization and On-Study Evaluations

- - - 1. Entry - Step 1: Initial Regimen

Entry evaluations must be completed at least 7 days after screening evaluations, no more than 3 days after randomization, and before dispensing Step 1 study treatment.

A negative serum or urine β-HCG pregnancy test result must be obtained within 48 hours before starting study treatment. (The urine test must have a sensitivity of 25-50 mIU/mL)

Study treatment will begin as soon as possible (within 3 days whenever possible) following randomization.

NOTE: There must be documentation that female participants of reproductive potential, who are randomized to EFV, agree to use two methods of contraception before receiving study treatment.

Participants who have not started study treatment within 14 days following randomization will be required to repeat the entry laboratory evaluations. If the repeated entry laboratories are within the limits of the study eligibility criteria, participants may initiate study treatment. If not, the participant will not initiate study treatment but may be followed on study until study closure.

Participants who are randomized but withdraw from the study prior to starting Step 1 study treatment should have week 0 and off-study forms completed and keyed. No further follow-up is required for these participants.

- - - 1. On-study evaluations must be scheduled on the weeks per the Schedule of Events (SOE). A window of ± 7 days will be allowed through the first 8 weeks after entry into Step 1, 2, or 3. After week 8 on Step 1, 2, or 3, a window of ± 14 days will be allowed. For purposes of calculating allowed windows for the SOE, weeks are determined from the date of study (or step) entry/randomization and not from start of study treatment date, if that differs from the entry date.

NOTE: Women of reproductive potential who are randomized to receive EFV will be asked at each visit (starting at week 2) whether they are using two methods of contraception. If they are not using two methods of contraception, they will be switched to NVP or another ARV drug. This information must be recorded in the source documents.

- - - 1. Participants who have virologic failure during Step 1, 2, **or 3** should have confirmatory samples drawn as soon as possible after the initial plasma HIV-1 RNA result >1000 copies/mL and, if possible, no later than the next scheduled study visit. Participants should continue their Step 1, 2 **or 3** regimen until the confirmatory plasma HIV-1 RNA test is completed. Evaluations per the SOE should be completed at the time of confirmatory testing for suspected virologic failure.

NOTE: Virologic failure is defined as two successive measurements of plasma HIV-1 RNA ≥1000 copies/mL, with the first measurement at the week 16 visit or later.

- - - 1. Participants who develop an AIDS-related **OI** or malignancy while on study treatment will require the evaluations per the SOE as soon as possible after diagnosis.
      2. After entry to Step 2 **or 3**, the evaluations will be repeated starting at week 2 per the SOE.

6.2.3 Premature Treatment Discontinuation Evaluations

Premature discontinuation of study treatment is defined as permanently stopping all study treatment prior to study completion.

Although off study treatment, participants will be encouraged to continue on study and attend all scheduled study visits and receive all study evaluations as outlined in the SOE.

For participants who prematurely discontinue study treatment plus have NOT reached a primary study endpoint, continue evaluations per Step 1, 2, **or 3** as appropriate.

6.2.4 Off-Study Evaluations

Are required at the time of the participant’s final visit.

6.2.5 Pregnancy

Women who become pregnant while on A5175 will be allowed to continue on study/on study treatment (except for EFV), but must first sign a pregnancy informed consent form. Pregnancy-related information will be recorded on the CRF at each study visit (see section 7.6, Procedures in the Event of On-Study Pregnancy and Breast-Feeding). If a pregnancy occurs between scheduled visits, complete and key all CRFs related to that pregnancy at the time the pregnancy is determined.

Sites or study participants are encouraged to prospectively register pregnancies that occur on study to The Antiretroviral Pregnancy Registry by fax at **+1 910-256-0637** (for non-U.S. sites) or **1-800-258-4263** from within the U.S. More information is available at: [http://www.apregistry.com](http://www.apregistry.com/).

### 6.3 Special Instructions and Definitions of Evaluations

**All clinical and laboratory information required by this protocol is to be present in the source documents. Sites must refer to the Source Document Guidelines on the DAIDS Web site for information about what must be included in the source document:** [**http://www3.niaid.nih.gov/research/resources/DAIDSClinRsrch/PDF/SourceDocAppndx.pdf**](http://www3.niaid.nih.gov/research/resources/DAIDSClinRsrch/PDF/SourceDocAppndx.pdf)

6.3.1 Documentation of HIV-1

Rapid HIV test or any licensed ELISA test kit, confirmed with a repeat ELISA, Western blot, or plasma HIV-1 RNA at any time prior to study entry.

NOTE: The term "licensed" refers to a FDA-approved kit or, for sites located in countries other than the United States, a kit that has been certified or licensed by an oversight body within that country.

The initial test result must be confirmed by a test that is different than the one used for the initial assessment. A reactive initial rapid test should be confirmed by either another rapid assay or an ELISA that is based on a different antigen preparation and/or different test principle (e.g., indirect versus competitive), or a Western blot, or a plasma HIV-1 RNA. An initial ELISA result must be confirmed with another method such as a rapid test, Western blot, or plasma HIV-1 RNA, but not by a repeat ELISA test.

**HIV-1 documentation is not recorded on the CRF.**

6.3.2 Medical History

A medical history, including allergies to any medications and their formulations, will be collected at screening and must be present in the source documents. All diagnoses identified by the ACTG criteria for clinical events and other diseases must be recorded on the case report forms (CRFs). See ACTG criteria at [**https://www.fstrf.org/apps/cfmx/apps/common/Portal/index.cfm**](https://www.fstrf.org/apps/cfmx/apps/common/Portal/index.cfm)**.**

6.3.3 Medication History

A medication history will be collected at screening and must be present in source documents, including: 1) a complete HIV treatment history, with start and stop dates of any ARV medication (estimated if the exact dates cannot be obtained);
2) immune-based therapy; 3) HIV-related vaccines, including blinded study medications; 4) any prescription medications taken for the treatment or prophylaxis of OIs, including actual or estimated start and stop dates, and, 5) all prescription and nonprescription medications taken within 30 days prior to entry, including actual or estimated start and stop dates.

Certain medications, whether they are prescription or nonprescription, will be recorded on the CRF and in the source document. The medications to be recorded on the CRF and in the source document are listed in the A5175 and A5185 MOPS.

All previous ARV drugs taken at any time prior to entry and all current medications should be recorded on the CRF, including **ARV drugs taken prior to study entry to prevent MTCT of the HIV virus.**

6.3.4 Race and Ethnicity

Information on race or ethnicity will be collected at screening only and entered at study enrollment. Information should be based on patient self-report, rather than the judgment of site personnel. Site-specific Race/Ethnicity Group Tables have been developed by each **non-U.S. CRS**. See the A5175 and A5185s MOPS.

6.3.5 Concomitant Medications

After entry, all prescription, non-prescription, alternative, and traditional concomitant medications taken since the last visit will be recorded in the source documents. All non-study-provided ARVs and certain prescription and non-prescription medications will also be recorded on the CRF. The medications to be recorded on the CRF are listed in the A5175 and A5185s MOPS. Alternative and traditional medications will be reported as Yes/No on the CRF.

6.3.6 Study Treatment Modifications

All modifications to study drug(s) including initial doses, participant-initiated and/or protocol-mandated interruptions, modifications, and permanent discontinuation of study drugs will be recorded on the CRF and in the source documents at each visit. Participant-initiated and protocol-mandated interruptions include both inadvertent and deliberate interruptions of any study drug(s). Treatment interruption is failure for any cause to take study drugs for more than 48 hours.

6.3.7 Clinical Assessments

Complete Physical Exam

A complete physical exam will be performed during the screening period only and will include auxiliary or oral temperature (centigrade), pulse, and blood pressure, weight, height, and examination of the following: HEENT (head, eyes, ears, nose, and throat), neck, chest (auscultation and percussion of the chest), heart, abdomen, extremities, skin, and neurologic.

Targeted Physical Exam

A targeted physical examination will be performed during the study, to be driven by any signs or symptoms previously identified or that the participant has experienced since the last visit (and must include at a minimum weight, auxiliary or oral temperature [centigrade], pulse, blood pressure; examination for the presence of thrush, lymphadenopathy, and rash; and lung examination [auscultation and percussion of the chest]).

Signs and Symptoms

At entry, record all signs/symptoms that occurred within 30 days prior to study entry on the CRF.

For on-study visits, record all Grade ≥3 signs/symptoms on the CRF. Any signs or symptoms that led to a change in ARV drugs, regardless of grade, must be recorded on the CRF.

See the Division of AIDS (DAIDS) Table for Grading the Severity of Adult and Pediatric Adverse Events, Version 1.0, December 2004 **(Clarification dated August 2009)**, at the RCC Web site [**http://rcc.tech-res.com**](http://rcc.tech-res.com/). For clinical signs, symptoms, and events that are not graded on the DAIDS Table, use the “estimating severity grade” scale.

If an EAE or pregnancy occurs between scheduled visits, complete and key all CRFs related to that EAE or pregnancy at the time the EAE is reported or the pregnancy is determined.

Diagnoses

Report in the source documents and on the CRF, diagnoses identified by the ACTG Criteria for Clinical Events and Other Events at study entry and since the last visit. Refer to the study CRF for the appropriate appendix used for the current ACTG criteria. If a woman becomes pregnant and remains on study, pregnancy-related information will be reported on a CRF.

All related signs, symptoms, laboratory results, or diagnostic test results observed or performed as part of establishing a diagnosis are also reportable on the CRF, regardless of grade.

Karnofsky Performance Score

Participants’ functional capabilities will be rated according to the Karnofsky Performance Scale at screening and recorded in the source documents. See the A5175 and A5185s MOPS.

6.3.8 Laboratory Evaluations

All laboratory values must be documented in the source documents.

At entry, record all laboratory values on the CRF, regardless of **g**rade.

**Post-entry, record on the CRF:**

- **Grade >3 laboratory toxicities**
- **Any laboratory toxicities that (a) led to a change in study treatment, regardless of grade or (b) are reported to the DAIDS RCC Safety Office, regardless of grade**
- **Any laboratory result that establishes a diagnosis**
- **All ALT and AST results (at each study visit)**
- **Albumin results Q24 weeks** **from study entry**
- **All bilirubin results for the first 24 weeks of ATV**

For serum chloride and urine glucose (which are not graded in the DAIDS Table for Grading the Severity of Adult and Pediatric Adverse Events, Version 1.0, December 2004 **[Clarification dated August 2009]**), record laboratory values that are clinically significant and indicate an “8” on the CRF (see the A5175 and A5185s MOPS for a definition of clinically significant).

Hematology

Hemoglobin, red blood cells (RBC), mean corpuscular volume (MCV), white blood cell count (WBC), differential WBC, absolute neutrophil count (ANC), and platelets.

Liver Function Tests

Total bilirubin, AST (SGOT), ALT (SGPT), and alkaline phosphatase.

NOTE: In addition to tests per the SOE, LFTs will be drawn at weeks 2, 4, 6, and 8 any time after NVP is initiated, and then monthly through the 20^th^ week on NVP treatment.

**Retrospectively, record on the CRF (regardless of grade) the following:**

- **All ALT and AST results since study entry that have not been reported previously**
- **Albumin results Q24 weeks from study entry**
- **All bilirubin results during the first 24 weeks of ATV treatment that have not been reported previously**

Serum Chemistries

Sodium, potassium, chloride, phosphate, bicarbonate (or total CO_2_), creatinine, and albumin.

Calculated **CrCl** will be used to determine study eligibility and to dose adjust renally excreted ARV drugs. See the A5175 and A5185s MOPS for calculation procedure.

Baseline calculated **CrCl** is obtained at screening and recorded on the eligibility checklist (not on a CRF).

Record any calculated **CrCl** associated with a change in study treatment or study drug discontinuation on the CRF.

**For all participants randomized to Arm A or Arm C, record on the CRF (both retrospectively and prospectively) CrCl (may be calculated CrCl using Crockcoft-Gault equation) at study entry and Q24 weeks from study entry.**

Other Chemistry Evaluations

Lactate

Lactate is recommended for evaluation of participants with suspected lactic acidosis. See A5175 and A5185s MOPS for lactate collection and storage guidelines.

Lipase

Lipase will be measured whenever needed for evaluation of suspected pancreatitis. A triglyceride level should be drawn with the lipase, whether fasting or not.

Obtain lipase/triglycerides at any initiation of ddI-EC **at entry into any step**, or at any time ddI-EC is substituted for another drug.

Triglycerides

Testing is recommended for participants with suspected pancreatitis.

NOTE: Cholesterol and triglyceride entry and on-study evaluations may be performed retrospectively on stored, frozen samples. Samples can be obtained either fasting or non-fasting. It should be noted in the source document whether samples were collected in a fasting or non-fasting state. The time and date of food or liquid ingestion, other than water, prior to these visits will be recorded on the CRF. **Fasting is defined as no food or liquid ingestion, other than water, for 8 hours prior to the study visit.**

Creatine Kinase (CK)

CK will be measured for evaluation of suspected ARV drug-related myopathy. If a baseline measurement is needed, it will be obtained from stored samples.

Pregnancy Test

For women with reproductive potential: **S**erum or urine β-HCG (urine test must have a sensitivity of **15-25** mIU/mL) should be performed within 48 hours before initiating study treatment and per the SOE.

If a pregnancy occurs between scheduled visits, complete and key all CRFs related to that pregnancy at the time the pregnancy is determined.

Urinalysis

A dipstick urinalysis for protein and glucose will be performed per the SOE.

Hepatitis B Serologies

**HBsAg** will be performed at screening and recorded on the CRF. Results will be given to all individuals who were screened, irrespective of whether they enter the study or not; and results will be given to all participants who were enrolled under protocol Version 1.0 and were tested at entry. In addition, the site investigator will discuss what options, if any, exist for HBV treatment outside of A5175 at their site. The decision of whether to enroll individuals with HBV/HIV coinfection will be left to the discretion of the site investigator.

6.3.9 Electrocardiogram

An EKG will be obtained at screening for participants who have symptoms potentially related to heart block (e.g., unexplained dizziness, syncope, palpitations or dyspnea) within 45 days prior to study entry. Perform whenever clinically indicated for participants receiving ATV. Record these data on the CRF.

6.3.10 Immunologic Studies

CD4+ and Total Lymphocyte Cell Counts

CD4+ cell counts (both absolute counts and subset percentage) will be performed with flow cytometry at screening (obtained within 90 days prior to study entry) and throughout the study at the same IQA or UKNEQAS participating laboratory, if possible.

Because of the diurnal variation in CD4+ cell counts these determinations for each participant should be obtained consistently in either the morning or the afternoon throughout the study.

Each time a CD4+ cell count is obtained, the local laboratory must perform a WBC and differential from a sample obtained at the same time.

All CD4+ cell count results will be recorded in the source documents and on the CRFs.

6.3.11 Virologic Studies

Plasma HIV-1 RNA

Quantitation will be performed on site in real time using the Roche Amplicor Monitor assay at a laboratory participating in the VQA proficiency program. See Virology Appendix I and the A5175 and A5185s MOPS.

All plasma HIV-1 RNA results will be recorded in the source documents. The appropriate CRFs will be completed as directed.

NOTE: Study visits after week 8 (Step 1, 2, or 3), can be scheduled ±14 days of the SOE weeks (e.g., the HIV-1 RNA result used for the week 16 visit may occur between weeks 14 and 18).

During the first 24 weeks on study, if a scheduled plasma HIV-1 RNA is missed because of a missed visit or for any other reason (e.g., specimen lost or laboratory error), then a plasma HIV-1 RNA should be performed at the next scheduled visit, even if one is not normally scheduled per the SOE.

Resistance Testing

Resistance testing will be performed retrospectively, using the Celera Diagnostics ViroSeq HIV-1 Genotyping Assay. See Virology Appendix I and the A5175 and A5185s MOPS.

6.3.12 Pharmacology Studies

EFV, NVP, ATV, or TDF Random Timed Specimens

Blood samples for randomly timed specimens will be collected at week 4, only from participants in Step 1 receiving EFV, NVP, ATV, or TDF. Time of blood draw and time of last EFV, NVP, ATV, or TDF dose will be recorded on the CRF and source documents. If evaluation at Week 4 is not possible, it can be done after Week 4 but no later than Week 8.

If a participant on Step 1 has been on an NNRTI and had a week 4 PK random sample collected but subsequently had the NNRTI changed for toxicity, a separate PK sample needs to be obtained for the second NNRTI approximately 4-8 weeks after substitution.

Concurrent Use of Rifabutin and ATV

After participants have been concurrently treated with rifabutin and ATV for at least 14 days, trough blood samples will be collected immediately prior to the next dose of ATV. The time of blood draws and times of last ATV dose will be recorded on the CRF. If the trough time point collection is at the same visit as the random time point collection (week 4 for ATV), only one sample needs to be obtained.

See Pharmacology Plan, Appendix V, and the A5175 and A5185s MOPS for sample collection, processing, and storage instructions.

6.3.13 Adherence

Trained site personnel will:

- Conduct the combined Adherence/Quality of Life/Psychosocial interviewer-administered questionnaires (quality of life, substance abuse, social support, and sexual risk taking) and record on the CRF (see Appendix IV).
- Administer the Adherence Interview and record on the CRF.

NOTE: Trained site personnel are defined as site pharmacist, nursing staff, clinician, or other trained clinical personnel (e.g., adherence counselors, social workers).

6.3.14 Adherence and Sexual Risk Reduction Counseling

Trained site personnel will provide the adherence and sexual risk-reduction counseling at study entry and as needed. Ideally, both the physician and trained site personnel will play an active role in counseling.

A checklist of discussion points will be utilized. The adherence counseling will cover information about adherence, motivational training, and adherence skills (e.g., scheduling and techniques for remembering). The sexual risk-reduction counseling will similarly follow an information, motivation, and behavioral skills building model. See the A5175 and A5185s MOPs for the counseling materials.

- - 1. **Pill Counts**

**Trained site personnel will perform** pill counts as outlined in the SOE for each Step 1 HIV medication (3TC/ZDV, FTC/TDF, EFV, ATV, ddI-EC, NVP, ZDV, d4T), and record on the CRF. **Record the number of pills dispensed at every visit on Step 1 including the entry visit.** Pill counts are not conducted on Step 2 **or 3.**

**6.3.16** Anthropometric Measurements

Height (at entry only), weight, mid-arm, mid-waist (conventional), mid-thigh, and hip circumferences will be measured and recorded in centimeters. See the A5175 and A5185s MOPS or the ACTG metabolic web site for measurement guidelines at

[**https://www.actgnetwork.org/members/download/other/metabolic/CIRCmeasures.doc**](https://www.actgnetwork.org/members/download/other/metabolic/CIRCmeasures.doc)

**6.3.17** Storage of Plasma, Serum, and PBMC at Sites

**The** purpose **of** collection and storage of plasma and serum is to conduct retrospective A5175-specific analyses as described in Appendices I (Virology Study Plan), II (Opportunistic Infections and Immune Reconstitution Study Plan), III (Malnutrition, Micronutrient Deficiency, Immunologic Functioning, and Metabolic Study Plan), and V (Clinical Pharmacology Plan). The A5175 and A5185s MOPS and Lab Processing Chart (LPC) describe the specific procedures for the collection and storage of these specimens. **U.S. CRS**s should ship the specimens to the BRI Repository. **Non-US CRS**s **should follow their quarterly shipment schedule for sending specimens to BRI.**

In addition, study participants may agree to have 2 tablespoons (30 mL) of blood collected for use in future ACTG-approved HIV-related research (non-A5175 research). The collection of blood for future ACTG research is separate from any blood that is collected for A5175-specific purposes, as described in the previous paragraph. To meet local or in-country requirements, sites may elect not to collect blood for future ACTG research. If a site or participant elects not to participate in the collection of blood for future ACTG research, collection and storage of PBMC specimens will not take place. However, collection and storage of plasma and sera for A5175-specified research is still required at Step 1 entry, weeks 24 and 48, then every 24 weeks thereafter, and as soon as possible after a new Grade ≥ 3 ALT (SGPT) or AST (SGOT).

### 6.4 A5185s Genital Secretions Substudy Evaluations

Specimens will be collected at study entry, weeks 48 and 96, regimen switch, and at study discontinuation (see Appendix VI and the A5175 and A5185s MOPS). **U.S. CRS**s will ship these specimens to the ACTG BRI repository according to their shipment schedule. **Non-U.S. CRSs** will store the specimens locally until requested.

## 7.0 ANTIRETROVIRAL MANAGEMENT: TOXICITIES, DRUG SUBSTITUTIONS, PREGNANCY, OPPORTUNISTIC INFECTIONS, AND OTHER CONDITIONS

This section provides guidelines for management of ARV study drugs provided through A5175. When one ARV drug is held for resolution of toxicity, all ARV drugs in the regimen should be held concurrently unless otherwise specified.

Every attempt should be made to continue to follow participants who discontinue study treatment because of a Grade 3 or 4 AE until resolution of the AE can be documented.

Toxicity management may require reliance on clinical symptoms, clinician judgment, and available laboratory markers, since alternatives to study-provided ARV drugs may be very limited and baseline levels of certain laboratory parameters (e.g., hemoglobin) may be different than in other settings. The A5175 CMC is available to discuss toxicity management of both study- and non-study ARV drugs with investigators. **Non-U.S. and** **U.S. CRS**s must contact the A5175 CMC to request permission to make Step 1 changes in **ARV** drug regimens and to register participants to Step 2.

It is recommended that NRTIs be continued for 7 days **after** NNRTI (e.g., EFV, NVP) discontinuation unless they are suspected in a given toxicity. If available, a PI may be substituted for the 7 days that the NNRTI has been discontinued and then also stopped when the NRTIs are discontinued at the discretion of the site investigator

3TC has activity against HBV. FTC and TDF may have activity against HBV. Permanent discontinuation of 3TC, FTC, or TDF may result in re-activation of HBV.

A5175 will use the DAIDS Table for Grading the Severity of Adult and Pediatric Adverse Events, Version 1.0, December 2004 **(Clarification dated August 2009),** as a guideline for managing toxicities related to study drugs unless otherwise noted. The table is located at the RCC web site [**http://rcc.tech-res.com**](http://rcc.tech-res.com/)**.**

### 7.1 Antiretroviral Therapy Dosage Reductions

Dosage reductions (as specified in Table 7 below) are permitted for **ARV** toxicity management.

Table 7. Dosage Reductions

| Drug | Initial Dose | Daily Dose | Reduced Dose | Daily Reduced Dose |
| --- | --- | --- | --- | --- |
| EFV | 600 mg QHS | 600 mg | None | None |
| ATV | 400 mg QD | 400 mg QD | None | None |
| NVP | 200 mg QD x 14 days, then 200 mg BID^3^ | 200 mg x 14 days, then 400 mg | None | None |
| 3TC | 150 mg BID | 300 mg | None | None |
| TDF | 300 mg QD | 300 mg | None | None |
| FTC | 200 mg QD | 200 mg | None | None |
| ZDV | 300 mg BID | 600 mg | 200 mg BID | 400 mg |
| ddI-EC^1^ | 400 mg QD | 400 mg | 250 mg QD | 250 mg |
| ddI-EC^2^ | 250 mg QD | 250 mg | 125 mg QD | 125 mg |
| d4T^1^ | 40 mg q12h | 80 mg | 20 mg q12h | 40 mg |
| d4T^2^ | 30 mg q12h | 60 mg | 15 mg q12h | 30 mg |

^1^For participants weighing ≥60 kg. **For d4T, this reduction only applies to those participants who initially received d4T 40 mg BID.**

^2^For participants **<60 kg taking ddI-EC and for participants** **at any weight who are receiving d4T 30 mg BID.**

^3^ If 200 mg BID is tolerated for ≥3 months, the dose may be changed to 400 mg QD.

### 7.2 Grades 1 or 2 Toxicity

Participants who develop a Grade 1 or 2 AE or toxicity may continue study drugs without alteration of the dosage except as stated in section 7.5, Specific Management of Laboratory Abnormalities and Clinical Syndromes. Participants experiencing Grade 1 or 2 toxicities will be managed at the discretion of the site investigator.

### 7.3 Grade 3 Toxicity

If there is compelling evidence that the AE has NOT been caused by the study drug(s), dosing may continue. Except as stated in section 7.5, participants who develop a Grade 3 AE or toxicity thought secondary to study medications or of unknown etiology may have all of their ARV study drugs withheld, at the site investigator’s discretion. Dose reductions are not permitted.

Investigators are encouraged to discuss toxicity management with the A5175 CMC. The participant should be re-evaluated weekly if possible until the AE returns to Grade ≤2, at which time the study drugs may be reintroduced at the discretion of the site investigator.

### 7.4 Grade 4 Toxicity

Participants who develop a symptomatic Grade 4 AE or toxicity, not specifically addressed below, will have all study drug(s) withheld until resolution of the AE to a Grade ≤2. Under certain circumstances the study drug thought most likely to be related to the AE may be resumed at the discretion of the site investigator after discussion with the A5175 CMC. Alternative study-provided or non-study-provided ARV drugs should be considered.

Participants with Grade 4 asymptomatic laboratory abnormalities, not specifically addressed below, may continue study drug therapy if the site investigator has compelling evidence that the toxicity is NOT related to the study drug(s), or if benefit of the study drug(s) outweighs the potential risk.

### 7.5 Specific Management

7.5.1 Rash Management for Participants Not on NVP

Grade 1 or 2

Study treatment should continue without interruption. Participants with a Grades 1 or 2 rash may be treated symptomatically with permitted antipyretic, antihistamine, and/or nonsteroidal anti-inflammatory medications, but should be monitored closely by the site investigator.

Grade 3

All study treatment should be held for any Grade 3 rash, unless the rash is determined to be unrelated to study medications. May restart study medications if clinically indicated when resolution to Grade ≤2.

Grade 4

**Permanently discontinue** all study medications.

7.5.2 Rash Management for Participants on NVP

LFTs should be performed promptly, and participants evaluated for signs and symptoms relating to clinical hepatitis and hypersensitivity reactions.

If LFTs are increased above the baseline level, NVP must be permanently discontinued regardless of the grade of the rash or grade change in the LFTs.

If participants have constitutional symptoms not thought to be due to other intercurrent illness (fever, blistering rash, oral mucosal lesions, facial edema, myalgia/arthralgia) and rash of any grade, NVP must be permanently discontinued.

If participants have signs or symptoms of clinical hepatitis (which may include nausea and/or vomiting, anorexia, jaundice, acholic stools, hepatomegaly, hepatic tenderness, fever, fatigue, arthralgia) and rash, NVP should be permanently discontinued.

For participants on NVP who develop a Grade 1 or 2 rash but with no constitutional symptoms, no increase above baseline of the LFTs, and no evidence of clinical hepatitis, NVP may be continued with very close follow-up at the discretion of the site investigator.

For participants on NVP who develop a Grade 3 or 4 rash, but with no constitutional symptoms, no increase above baseline of the LFTs, and no evidence of clinical hepatitis, NVP should be discontinued.

For participants who have mild to moderate urticaria, without constitutional symptoms, without increases above baseline of the LFTs, or without evidence of clinical hepatitis, NVP may be continued with close follow-up at the discretion of the site investigator. If participants have an urticarial rash and NVP is discontinued for whatever reason, NVP should not be restarted.

7.5.3 Lipase Elevations and Pancreatitis

Pancreatitis will be reported as a clinical finding (i.e., symptomatic pancreatitis). The primary enzyme abnormality used for making the diagnoses is the lipase level. When obtained, lipase determinations will be recorded in the CRF. A triglyceride level should be drawn with the lipase.

Lipase will be obtained for participants if development of clinical symptoms suggests pancreatitis. If a baseline measurement is needed, it will be performed from stored entry samples (**p**ancreatic amylase is also acceptable).

Obtain baseline (entry) lipase/triglycerides at any initiation of ddI-EC on either Step 1 or Step 2 entry or at any time ddI-EC is substituted for another drug.

For symptomatic gastrointestinal symptoms (particularly abdominal pain) with elevations in lipase:

Grade 1

Search for other causes of symptoms. If none is found and symptoms persist, repeat lipase within 2 weeks. IF REPEAT IS ELEVATED THEN PARTICIPANT SHOULD STOP ddI-EC.

Grade ≥2

Follow participants and repeat lipase as soon as possible. If lipase is persistently elevated and accompanied by symptoms, then participants should be considered to have clinical pancreatitis. CT scan of the abdomen, if available, may also be helpful in determining whether clinical pancreatitis is present. Exclude other possible diagnoses (e.g., renal insufficiency causing false elevations in lipase). If none is found, diagnose as clinical pancreatitis.

For a diagnosis of pancreatitis (clinical), all study medications should be held.

After complete resolution of the episode in a setting in which other concomitant illness might have reasonably contributed to the development of pancreatitis, rechallenge with study medications may be performed in consultation with the A5175 CMC. If the study regimen included ddI-EC or d4T, these medications should be permanently discontinued. ddI-EC should be substituted with TDF. d4T should be substituted with ZDV and rechallenge with the new regimen.

Upon rechallenge, lipase determinations should be performed monthly. Any elevation of lipase of Grade ≥2 or any recurrence of symptoms during this period will lead to a reevaluation and permanent discontinuation of the suspected study drugs(s).

7.5.4 CK Elevation

CK measurements will not be performed routinely as part of the protocol. CK will be measured only if participants develop clinical symptoms consistent with a diagnosis of myopathy. If a baseline measurement is needed, it will be performed from stored samples.

For persistent CK elevations >3000 mg/dL (about 20 x ULN) in symptomatic participants (before treatment modifications are made) CK should be redrawn after participants abstain from exercise for 24 hours. If CK is still >3000 mg/dL, ZDV should be discontinued and replaced with d4T or another NRTI as appropriate.

7.5.5 AST and ALT Elevation

Nearly all the ARV drugs, isoniazid (INH), and concomitant illness can cause alterations in LFTs. Therefore, changes in AST or ALT should be evaluated within the clinical context of the participant. Initiation of ARV treatment and INH has been staggered to facilitate the interpretation of LFTs. Because INH and the NNRTIs have been most associated with serious, life-threatening hepatitis, evaluation of LFTs in the setting of these drugs is discussed separately.

General Considerations: For asymptomatic elevation in AST or ALT of 5-10 × ULN (Grade 3), medications other than NNRTIs and INH may be continued at the discretion of the site investigator. Careful assessments should be done to rule out alcohol use, non-study medication-related drug toxicity, the lactic acidosis syndrome, and viral hepatitis as the cause of the transaminase elevation. If the AST/ALT elevation is considered most likely to be due to concomitant illness or medication, standard management, including discontinuation of the likely causative agent, should be undertaken.

For asymptomatic elevation 5-10 × ULN (Grade 3) believed secondary to study medications, all agents must be held until levels are Grade ≤2, at which time therapy may be reintroduced with the substitution of ATV for EFV or NVP, if applicable.

For asymptomatic or symptomatic elevation of AST or ALT >10 × ULN (Grade 4), all medications must be discontinued and held until levels are Grade ≤2, at which time therapy may be reintroduced with the substitution of ATV for EFV or NVP, if applicable. All medications may be restarted if the laboratory abnormalities were thought secondary to a concomitant illness. If the participant was receiving an NNRTI (EFV or NVP), either of these medications should be considered the most likely cause of the elevations. The medications should be substituted and the NRTI medications can be resumed. If elevations >10 × ULN (Grade 4) recur in the absence of an NNRTI drug, all current ARV treatment and isoniazid (INH) (if participant is receiving INH) must be discontinued. Alternative ARV treatment and TB prophylactic regimens may be considered, at the discretion of the site investigator.

INH Prophylaxis: Participants will not start INH if AST/ALT are >3 × ULN. At one month following initiation of INH, if AST/ALT are >3 times the baseline value, INH will be discontinued. In the event of AST or ALT >5 × ULN (Grade ≥ 3) at any point thereafter, INH should be discontinued.

Clinical (Symptomatic) Hepatitis with NVP or EFV: Participants taking EFV or NVP should be monitored for the development of signs and symptoms of hepatitis, which may include fatigue, malaise, anorexia, nausea, acholic stools, bilirubinuria, jaundice, liver tenderness, or hepatomegaly, with or without initially abnormal serum transaminase levels. Anyone with these signs and symptoms must seek medical attention immediately and have LFTs performed. If the study clinician determines that the participant has clinical hepatitis with or without LFT abnormality or regardless of the degree of LFT abnormality, and NVP cannot be excluded as the cause, NVP should be permanently discontinued and not restarted after recovery. For asymptomatic elevation in AST (SGOT) or ALT (SGPT) >5 × ULN (Grade ≥3), NVP should be discontinued. **If any participants on NVP are found to have Child-Pugh Class B or C hepatic impairment, they will be switched to an alternative ARV medication either provided through the study or obtained outside the study. CRSs should continue to follow the drug substitution guidelines in sections 5.1.1.1 and 5.1.1.2.**

HBV or HCV Coinfection: At study entry, HBsAg will be obtained to facilitate management of HBV coinfected participants in the event 3TC, FTC, or TDF need to be discontinued, which would potentially worsen HBV disease.

7.5.6 Anemia/Neutropenia/Thrombocytopenia

Clinicians should be alert to clinical signs of anemia, neutropenia and thrombocytopenia and participants should receive education or counseling about the associated symptoms. Participants who develop anemia, neutropenia, and/or thrombocytopenia on study should be evaluated for causes of anemia and/or neutropenia, such as concurrent bacterial, mycobacterial or fungal infection, malaria, helminthiasis malignancy and/or malnutrition. Transfusion should be considered if clinically appropriate.

**Grade 1**

Participants who develop Grade 1 anemia and/or neutropenia during treatment with ZDV, which is considered treatment-limiting in the opinion of the site investigator, may have the ZDV dose reduced to 200 mg BID or substituted with d4T. If treatment-limiting symptoms persist after dose reduction, d4T may be substituted for ZDV. If ZDV is dose reduced, a hematology panel should be rechecked 4 weeks after dose reduction.

**Grade 2**

Participants who develop Grade 2 anemia and/or neutropenia during treatment with ZDV should have a reduction of the ZDV dose or substitution of d4T for ZDV. If ZDV is dose reduced, a hematology panel should be rechecked 4 weeks after dose reduction.

**Grade 3**

For Grade 3 anemia, neutropenia, or thrombocytopenia believed secondary to ZDV, d4T should be substituted for ZDV. Alternatively, ZDV may be held until the toxicity event returns to Grade ≤2, at which time ZDV may be resumed at a reduced dose. If the same Grade 3 AE recurs on a reduced dose of ZDV, then ZDV should be replaced with d4T. The investigator, in conjunction with the A5175 CMC, may choose to continue ZDV at a reduced dose in the setting of Grade 3 anemia if the risks of discontinuing the ZDV outweigh the benefits and a switch to an alternative NRTI (preferably d4T) is not feasible. A hematology panel should be rechecked within 4 weeks after treatment modification.

**Grade 4**

Participants with Grade 4 anemia, neutropenia or thrombocytopenia attributed to ZDV will have treatment interrupted until the AE has returned to Grade ≤2. Once the toxicity has returned to Grade ≤2, all ARV therapy should be restarted, with d4T substituted for ZDV, or if necessary, ZDV administered at a reduced dose of 200 mg BID. Alternatively, at the discretion of the site investigator, d4T or an equivalent NRTI may be substituted for ZDV without interruption of study streatment. A hematology panel should be rechecked within 4 weeks after restarting ARV treatment.

Recurrent Grade 4 anemia, neutropenia, or thrombocytopenia will result in discontinuation of ZDV and substitution of d4T or an equivalent NRTI.

Additionally, if Grade 2 to 4 anemia and/or neutropenia do not respond to reduction of the ZDV dose, or the interruption/discontinuation of ZDV, then other causes should be sought. Invasion of the bone marrow by mycobacterial infection or lymphoma or sepsis should be considered and excluded.

NOTE: The use of d4T is recommended as a substitution drug for management of ZDV-related anemia, neutropenia or thrombocytopenia. However, TDF may be used instead of d4T at the discretion of the site investigator.

7.5.7 CNS Symptoms with EFV

There have been reports of delusions and inappropriate behavior, predominantly in individuals with a history of mental illness or substance abuse. Severe acute depression has also been infrequently reported in both EFV-treated and control-treated individuals. Discontinuation of EFV may be required and substitution of NVP implemented.

In the event that a study participant experiences treatment-limiting CNS AEs attributable to EFV, EFV should be discontinued and may be replaced with NVP.

7.5.8 Peripheral Neuropathy

Grade 1

Study medications may be continued at their present dosage. Symptomatic treatment may be provided at the discretion of the site investigator.

Grade 2

Participants experiencing Grade 2 symptoms will be managed per site investigator discretion, which may include dose reduction (see Table 7, Dosage Reductions, in section 7.1), temporary cessation of d4T or ddI-EC, or symptom management.

If Grade 2 toxicity resolves to Grade 1 within 28 days after a dose reduction in d4T, then d4T may be continued at the reduced dose or increased back to the initial dose at the discretion of the site investigator.

Grade 3

Participants who experience symptoms consistent with peripheral neuropathy that is unrelieved with non-narcotic analgesics (Grade 3) must have d4T and/or ddI-EC permanently discontinued, and another NRTI should be substituted. Symptomatic treatment may be provided at the discretion of the site investigator.

7.5.9 Nausea (with or without vomiting)

Isolated nausea following initiation of ARV drugs usually subsides or resolves during the first few weeks of treatment.

Steps in the management of nausea include taking the medication with food (with the exception of ddI-EC) and administration of antiemetics. In the event of intractable nausea and pancreatitis, lactic acidosis, etc. have been investigated, substituting another NRTI for ddI-EC or ZDV is permitted.

7.5.10 Lactic Acidosis

The following definition will be used.

Symptomatic Hyperlactatemia

New, otherwise unexplained and persistent (≥2 weeks) occurrence of one or more of the following symptoms:

- Nausea and vomiting
- Abdominal pain or gastric discomfort
- Abdominal distention
- Increased LFTs
- Unexplained fatigue
- Dyspnea

and

Lactate level (if available) >2 × ULN confirmed by repeat lactate level analysis. In the absence of lactate levels, serum bicarbonate levels and anion gap should be assessed. The presence of depressed bicarbonate levels or an increased anion gap would suggest the possibility of lactic acidosis.

NOTE: All lactates >2 × ULN should be repeated as soon as possible, generally within 1 week. If the second result confirms hyperlactatemia (>2 × ULN), participants should immediately discontinue their current study regimen. Substitution of TDF for ZDV or d4T should be considered once symptoms resolve and lactate levels return to <2 ×ULN.

See the A5175 and A5185s MOPS for background, lactate collection, and storage guidelines.

7.5.11 Diarrhea

Diarrhea is a common side effect of infection and medication toxicity. If no infectious cause of diarrhea is found and onset is temporally related to new medication, symptomatic management with antidiarrheal agents is appropriate.

7.5.12 Hypophosphatemia

**Grades 1 and 2**

For Grades 1 and 2 hypophosphatemia, phosphate should be repeated as soon as possible (within 2 weeks is optimal), and TDF may be continued without other signs of renal tubular acidosis (see A5175 and A5185s MOPS) at the discretion of the site investigator.

**Grades 3 and 4**

For Grades 3 and 4 hypophosphatemia, the phosphate should be repeated preferably within 1 week. Supplemental phosphate or foods high in phosphates should be given and other causes of low phosphate should be investigated. If Grade 3 or 4 hypophosphatemia persists discontinue TDF permanently.

7.5.13 Hyperbilirubinemia (for participants on ATV)

Participants taking ATV may experience asymptomatic elevations in indirect (unconjugated) bilirubin. This hyperbilirubinemia is reversible upon discontinuation of ATV. Hepatic transaminase elevations that occur with hyperbilirubinemia should be evaluated for alternative etiologies. No long-term safety data are available for participants experiencing persistent elevations in total bilirubin ≥5 **x** ULN. ATV discontinuation may be considered if jaundice or scleral icterus associated with bilirubin elevations presents cosmetic concerns for the participant. Dose reduction of ATV is not permitted. ATV-related, asymptomatic hyperbilirubinemia (Grade 4 total bilirubin) or jaundice without LFT elevation (Grade ≤ 1 AST, ALT, and alkaline phosphatase) does not require expedited adverse event (EAE) reporting. For EAE reporting requirements see section 10.4.

7.5.14 Cardiac Management (for participants on ATV)

Obtain EKG for participants who have symptoms potentially related to heart block (e.g., unexplained dizziness, syncope, palpitations or dyspnea).

7.5.15 Renal Insufficiency

Dose modifications are recommended for TDF, FTC, ddI-EC, d4T, and 3TC in participants with reduced **CrCl** (see the most recent package inserts). TDF should be held for a confirmed calculated **CrCl** <50 ml/min until an underlying etiology for the renal insufficiency is determined. If no other etiology is determined or the renal insufficiency improves with holding TDF, permanently stop TDF.

Baseline serum creatinine will be the mean of the screening and entry values. If at any time serum creatinine is increased >1.5-fold above baseline, the serum creatinine should be repeated as soon as possible (preferably within 1 week). Participants with confirmed serum creatinine increases >1.5-fold above baseline should undergo an evaluation for potential causes of decreased renal function. Participants with confirmed increased serum creatinine >1.5-fold above baseline should have serum creatinine monitored more frequently, at the discretion of the site investigator, until serum creatinine either stabilizes or decreases to ≤1.5-fold above baseline. Drug dosing adjustments should be done based on the calculated **CrCl**. See A5175 and A5185s MOPS.

7.5.16 Drug Substitutions

In the event of treatment-limiting toxicity, the following substitutions are allowed at any time on either Step 1 or 2. All Step 1 drug substitutions must be pre-approved by the A5175 CMC ([actg.cmca5175@fstrf.org](mailto:actg.cmca5175@fstrf.org)).

Table 8. Suggested Drug Substitutions

| Initial Drug | Substitution Drug | Other Indication/Comment |
| --- | --- | --- |
| ZDV | d4T | For anemia at Step 1 study entry, at any regimen switch, or toxicity management.  The protocol recommends d4T as a substitution drug for ZDV; however, TDF may be used instead of d4T for any reason stated in these sections, at the discretion of the site investigator. Where ZDV is used as part of a **FDC** with 3TC, and d4T is substituted for ZDV, then 3TC will be substituted by FTC.  d4T and ZDV should never be used together. |
| d4T | ZDV | None |
| EFV | NVP | NVP may be substituted for EFV.  There are little data available about the risks/benefits of changing from EFV to NVP for EFV-related AEs.  NVP will require LFT monitoring at weeks 2, 4, 6 and 8, after initiation of NVP, then monthly through the 20^th^ week of NVP treatment.  If NVP is substituted for EFV in Arm 1A or Arm 1C, dose NVP 200 mg PO QD for the first 14 days. Thereafter, dose NVP 200 mg PO BID. If NVP is tolerated for ≥3 months, the dose may be changed to 400 mg QD.  ATV may be substituted for EFV if there is toxicity or contraindication to both EFV and NVP. |
| ATV | EFV | If EFV is contraindicated, NVP may be used. |
| TDF | ddI-EC | Use if participants experience dose-limiting toxicity to TDF (e.g., confirmed calculated **CrCl** <50mL/min).  d4T may be substituted for TDF if there is toxicity or contraindication to both TDF and ddI-EC. |
| ddI-EC | TDF | Use for dose-limiting toxicity with ddI-EC (e.g., pancreatitis).  d4T may be substituted for ddI-EC if there is toxicity or contraindication to ddI-EC or TDF.  TDF will not be used in the ATV arm unless ATV is boosted with low-dose ritonavir. |

### 7.6 Procedures in the Event of On-Study Pregnancy and Breast-Feeding

A5175 will not provide perinatal care for women. Women who become pregnant will be referred to local clinics and/or other research studies for prenatal and postpartum care.

After a pregnancy informed consent is obtained, monitoring for toxicity related to ARV drugs will continue during and after pregnancy. If CD4+ **cell** counts are ≥ 250 cells/mm^3^, women may discontinue ARV therapy during the first trimester of pregnancy.

7.6.1 Women on an EFV-Containing Regimen

Women who are taking EFV and become pregnant will immediately stop EFV and substitute a different ARV drug such as NVP when appropriate. The site investigator will determine which ARV drug should be substituted for EFV and whether the woman should return to EFV following pregnancy. In particular for pregnant women with CD4+ **cell** counts >250 cells/mm^3^, an ARV drug other than NVP should be considered.

7.6.2 Women on a ddI-EC plus d4T-Containing (Step 2) Regimen

The use of ddI-EC plus d4T is not allowed for pregnant women. ddI-EC will be replaced with 3TC, and/or d4T will be replaced with ZDV. Women may return to their secondary regimen following pregnancy at the discretion of the site investigator.

- - 1. Women on a TDF-, FTC-, and/or ATV-Containing Regimen

Data on the safety of TDF, FTC, and ATV in pregnancy are limited. Therefore, women who become pregnant while taking TDF, FTC, and/or ATV will be informed of the lack of safety data in humans taking these drugs during pregnancy. The appropriate dose of ATV for use in pregnancy has not yet been determined. If other ARV drugs are available and appropriate (e.g., ZDV), women may be counseled to change therapy to an appropriate substitute.

7.6.4 Women on a Regimen Containing Two ARV Drugs with Hepatotoxic Potential

Women who become pregnant on study should be monitored closely for liver toxicities when they are taking two hepatotoxic ARV drugs (e.g., d4T, NVP) concurrently.

7.6.5 Women Who Breast-Feed

Changes in ARV treatment for women who are breast-feeding will be at the discretion of the site investigator. EFV is used in HIV-exposed infants and HIV-infected children. Data on the use of TDF and other ARV agents during breast-feeding are limited. Breast-feeding participants receiving study drugs will be allowed to continue their use while breast-feeding at the discretion of the site investigator. Breast-feeding is permitted where formula feeding is not a viable option per WHO guidelines. See WHO guidelines in the A5175 and A5185s MOPS for women who breast-feed.

### 7.7 Recommendations for Management of Immune Reconstitution Inflammatory Syndromes

When these syndromes are suspected, the following management plan and consultation with the A5175 CMC are recommended:

- Continue ARV treatment;
- Confirm diagnosis of OI;
- Continue or initiate specific therapy for the infection;
- Evaluate the participant clinically to exclude a new infectious process if the participant was already receiving therapy for the OI; and
- Initiate anti-inflammatory agents, initially non-steroidals or, if needed, corticosteroids at the discretion of the site investigator in consultation with the A5175 CMC.

When OIs are associated with inflammatory signs or symptoms and accompanied by an increase in the CD4+ **cell** counts and a drop in plasma HIV-1 RNA levels, these events may not represent clinical failure and should not initially be considered clinical endpoints. These events should be captured as clinical events without virologic and immunologic failure and managed as outlined above (i.e., continue study treatment while treating the OI.)

When OIs occur in the setting of virologic and/or immunologic failure:

- These events will be considered as primary study endpoints.
- Initiate specific therapy for the clinical event.
- Switch ARV regimen if criterion for virologic failure is met (see section 3.0).

### 7.8 Management of ATV When Treating Tuberculosis

Participants who are taking ATV and require rifampicin for the treatment of active TB should either replace rifampicin with rifabutin or replace ATV with EFV, during the period of rifampicin treatment. Participants should wait 2 weeks after stopping rifampicin before resuming ATV. Participants who receive rifabutin for the treatment of TB may remain on ATV (see the most recent ATV package insert).

### 7.9 Procedures in the Event of Imprisonment or Involuntary Incarceration or Detention

Participants who become detained, imprisoned, or involuntarily incarcerated during the course of the study will have study treatment and follow-up interrupted during the period of incarceration, detention, or imprisonment; but study treatment and follow-up may be resumed, at the discretion of the site investigator and with the approval of the A5175 CMC, after participants are released from incarceration, detention, or imprisonment.

### 7.10 Treatment of Hepatitis B Virus

HBV/HIV coinfected participants who receive only one study-provided, active anti-HBV agent may **receive entecavir (provided** outside of the study**)** for the treatment of HBV infection**,** at the discretion of the site investigator.

## 8.0 CRITERIA FOR PERMANENT TREATMENT AND STUDY DISCONTINUATION

### 8.1 Criteria for Permanent Treatment Discontinuation (Off Treatment/On Study)

- Drug-related toxicity (see section 7.0).
- Requirement for prohibited concomitant medications (see section 5.4).
- Failure by the participant to attend three consecutive clinic visits for any reason.
- Participant repeatedly noncompliant with study medications as prescribed.
- Clinical reasons believed life threatening by the physician, even if not addressed in the toxicity management of the protocol.
- Request of the primary care provider if s/he thinks the study treatment is no longer in the best interest of the participant.

### 8.2 Criteria for Permanent Study Discontinuation (Off Treatment/Off Study)

- Request by the participant to withdraw.
- Participant judged by the site investigator and the A5175 Clinical Management Committee to be at significant risk of failing to comply with the provisions of the protocol as to cause harm to self or others.
- At the discretion of the ACTG, NIAID, **Office for Human Research Protections (OHRP),** investigator, pharmaceutical **supporter**(s), within-country national health agency, or site **IRB**.

## 9.0 STATISTICAL CONSIDERATIONS

### 9.1 General Design Issues

- - 1. **Original Study Design**

A5175 is a phase IV, randomized, open-label, three-arm trial of antiviral efficacy. HIV-1-infected men and women ≥18 years of age and naïve to HIV ARV treatment with CD4+ cell count <300 cells/mm^3^ will be enrolled at study entry.

Randomization will be stratified by country and by screening plasma HIV-1 RNA <100,000 copies/mL versus ≥100,000 copies/mL.

A total of 1520 participants will be enrolled to ensure 1368 evaluable participants (456 participants per treatment arm), assuming no more than a 10% dropout rate. All randomized participants will be followed until either 30% of participants have reached the primary endpoint or for a total study duration of 2.5 years after randomization of the first participant, whichever occurs later in time. Visits are scheduled at screening, entry, and weeks 2, 4, 8, 12, 16, 20, 24, and every 8 weeks thereafter. At entry to Step 2 post-entry visits conducted in Step 1 are repeated.

The primary study objectives investigate whether treatment Arm 1B, a regimen of two NRTIs QD + a PI QD is not inferior to Arm 1A, a regimen of two NRTIs BID + NNRTI QD; and whether Arm 1C, a regimen of two NRTIs QD + NNRTI QD is not inferior to Arm 1A. Specifically, the comparisons between Arm 1B and Arm 1A and between Arm 1C and Arm 1A will be based on one-sided non-inferiority evaluations. These evaluations will be based on the primary study endpoint, time to treatment failure as defined in 9.2.1. The primary analysis will be based on information until the time of study closure (after 30% of participants have reached a primary endpoint or 2.5 years after the date that the first participant is randomized, whichever occurs later) and will be based on all information collected to that time.

**9.1.2 Updates Following Closure of Arm 1B at May 2008 DSMB Interim Review**

**Arm 1B was closed on May 23, 2008, following the DSMB interim review on May 6, 2008. The closing of Arm 1B was based upon conclusive evidence of inferiority of this arm compared to the control arm (1A) for the primary study endpoint of regimen failure: (P = 0.0012). The estimated hazard ratio was 1.67 with 99.8% confidence limits [1.02, 2.75].**

**Participants from the closed arm continued to be followed, but on a different antiretroviral regimen than that assigned by Arm 1B, and using an administrative Step 3 to track and monitor this follow-up.**

**The total study duration was amended to be when 30% of participants who were randomized to Arm 1A or 1C (i.e., the arms remaining open for efficacy comparison) reached the primary efficacy endpoint. Because 2.5 years since the first randomization had already elapsed, study follow-up was based upon primary efficacy endpoint accumulation.**

**Finally, the study objective related to comparing Arm 1B to Arm 1A was answered by the May 2008 interim review. Therefore, the remaining primary objective relates to comparison of Arm 1C to Arm 1A.**

**9.1.3 Updates Following November 2009 DSMB Meeting**

**The final analysis will commence once study follow-up is completed on May 31, 2010, according to ACTG guidelines and standard operating procedures on publications and disclosure of study results SOP 11 (01/01/07) available on the ACTG website.**

9.2 Endpoints

9.2.1 Primary endpoint

Time to treatment failure will be defined as the time from randomization to the first occurrence of any of the following:

- Death (due to any cause).
- Disease progression - defined as a new or recurrent AIDS-defining opportunistic infection or malignancy occurring at least 12 weeks following randomization. A list of AIDS-defining opportunistic infections and malignancies is provided in the A5175 and A5185s MOPS, located on the PSWP [**https://www.actgnetwork.org/protocols/protocol_specific_search.aspx**](https://www.actgnetwork.org/protocols/protocol_specific_search.aspx)**.**

NOTE: Immune reconstitution inflammatory syndrome (IRIS) will not be counted as a primary study endpoint.

- Virologic failure - defined as plasma HIV-1 RNA ≥1000 copies/mL on two consecutive measurements obtained at the week 16 visit or later.

NOTE **A**: Women who become pregnant during Step 1 and stop all study treatment will not be considered to have reached a primary endpoint. After pregnancy, study treatment will be restarted and women who fail to achieve plasma HIV-1 RNA <1000 copies/mL within 16 weeks after restarting study treatment will be considered failures at that time. Women who do not restart study treatment prior to the end of the study or do not have 16 weeks of follow-up after restarting study treatment will be considered as censored observations at the time study treatment was originally discontinued.

NOTE **B**: Since study visits for either step after week 8 can be scheduled ±14 days of the weeks per the SOE (section 6.2.2), virologic failure may occur at weeks 14 through 18, for example.

- - 1. Secondary endpoints
- Time to discontinuation of initial ARV therapy: defined as premature discontinuation of participation in the study, failure to take ARV therapy for ≥8 consecutive weeks, or switch to another ARV regimen for any reason. This definition does not include substitution of d4T or TDF for ZDV, NVP for EFV, or ddI-EC for TDF. It does, however, include any substitution of ATV for EFV or NVP (and conversely).
- Time to immunologic failure: defined to be a CD4+ **cell** count <100 cells/mm^3^ occurring at or after week 48. Participants who never achieve CD4+ **cell** counts above 100 cells/mm^3^ or who achieve CD4+ **cell** counts above 100 but drop below 100 before week 48 and stay below 100 until week 48 will be assigned a failure time of week 48.
- Change in CD4+ cell counts from baseline over time.
- Time to first dose modification or Grades 3 or 4 **adverse event**.
- Time to second regimen failure (defined per section 9.2.1).
- Plasma HIV-1 RNA <400 copies/mL at weeks 24 and 48).
- Time to loss of virologic response (TLOVR). (The FDA definition of TLOVR is provided in <http://www.fda.gov/cder/guidance/3647fnl.pdf>.)

9.3 Sample Size and Accrual

- - 1. **Original Study Sample Size**

Accrual is expected to be completed in 54 weeks following initiation of Stage II enrollment (see section 4.6.1). It is expected that 30% of the participants will have reached the primary endpoint no later than 96 weeks after enrollment of the last participant.

The experimental arm (Arm 1B or 1C) will be considered non-inferior to the reference arm (Arm 1A) if the upper one-sided 95% confidence bound for the experimental to reference arm hazard ratio is ≤1.35. Primary analysis will take place when 30% of participants, averaged across treatment arms, have reached the primary endpoint unless this occurs before 2.5 years after the date the first participant is randomized (earliest termination date). It is expected that 30% of the participants will have reached the primary endpoint, no later than 96 weeks after randomization of the last participant. If the underlying failure rates of the experimental arm (Arm 1B or 1C) or plural arms and the reference arm (Arm 1A) are truly the same and if the percentage of participants reaching primary endpoint averages 30% across the two treatment arms being compared, then 1368 evaluable participants (456 participants per arm) provide 80% power to demonstrate non-inferiority of the experimental arm (Arm 1B or Arm 1C) as compared to reference Arm 1A. Assuming that no more than 10% of participants are lost to follow-up, a total sample size of 1520 participants is required to ensure 80% power for each test. If 30% of participants reach the primary endpoint within 2.5 years after the date that the first participant is randomized, the primary analysis will be based on the information available until 2.5 years after the first randomization. In this case, more than 30% of participants will have reached endpoint, which will provide additional power.

9.3.2 Updates Following Closure of Arm 1B at May 2008 DSMB Interim Review

**Accrual was completed in August 2007. Because one arm (1B) was closed following the May 2008 interim review, the primary analysis timing listed above was amended to the following: when 30% of participants who were randomized to Arm 1A or 1C have reached the primary endpoint. As the power calculations above relate to each of the pairwise comparisons of experimental arm to control arm, then the power calculation above applies to the remaining efficacy comparison of Arm 1C to Arm 1A. Additionally, because accrual commenced in May 2005, the criterion of 2.5 years following first randomization has already passed, and therefore is no longer part of the functional definition of study follow-up duration.**

9.4 Monitoring

**9.4.1 Original Study Design**

When 20% (304) of participants have reached 8 weeks of follow-up, a report of Grades 3/4 toxicities and treatment discontinuation grouped across study arms and by site, will be distributed to the core protocol team. Based on this report, the actions of the core team may include the following: no action; modification of doses or drugs; or request for a DAIDS DSMB review of a more detailed toxicity report by study arm, regardless of whether the criteria for an interim analysis have been met. Thereafter, toxicity reports will be compiled and reviewed on a monthly basis. All reports will include reporting of AEs by clinical site.

Criteria for Planned Interim Analyses

Interim efficacy analyses will be conducted annually starting one year after the study has opened. Comparison of the primary endpoint between Arms 1A and 1B and Arms 1A and 1C will be done as described in section 9.5.1. If statistical tests for comparison of treatment arms demonstrate inferiority of an experimental arm (1B or 1C) to the referent arm 1A at the level of p<0.001, consideration will be given to stopping the inferior arm.

As an additional safety mechanism, an interim analysis will take place when 40% of participants in any given treatment arm have discontinued their study regimen for any reason, regardless of the time until the next scheduled review. Should this situation occur, the study will be reviewed by the DSMB at the earliest possible time. If either arm is shown to be inferior to the other, consideration will be given to stopping the study. The DSMB will conduct a review of A5175 on at least an annual basis.

**9.4.2 Updates Following Closure of Arm 1B at May 2008 DSMB Interim Review**

**Even though Arm 1B was closed on May 23, 2008, monitoring of all participants on A5175 will continue at least annually. When efficacy is reviewed, comparison will be only between Arms 1A and 1C. However, efficacy information for participants who were assigned to Arm 1B (and are currently being followed on other regimens in Step 3) will be described and summarized separately by pre- versus post-Arm 1B closure. For safety reviews, comparisons will be limited to the two remaining arms (1A and 1C), and safety data of continued (i.e., Step 3) follow-up of Arm 1B participants will be described and summarized.**

**9.4.3 Updates Following November 2009 DSMB Meeting**

**Due to planned closure to all study follow-up by May 31, 2010, the DSMB decided that they would not need to perform any subsequent reviews of the PEARLS study.**

9.5 Analyses

All analyses are ITT with the exception of an as-treated secondary analysis described in section 9.5.2. In analyses of time-to-event data, all available follow-up information prior to the event of interest is used and participants are assigned to their randomized treatment arm regardless of whether they have previously discontinued the assigned treatment. Participants lost to follow-up are censored at the planned visit week of their last clinic visit.

9.5.1 Primary Analyses

- - - 1. **Original Study Design**

The primary analysis will be ITT. Primary analysis will be conducted using the log-rank test, stratified by country and by screening plasma HIV-1 RNA (< versus ≥100,000 copies/mL). Hazard ratio 95% upper bounds (UBs) will be calculated using proportional hazards regression. The experimental arms (Arms 1B and 1C) will be considered non-inferior to the reference arm (Arm 1A) if the one-sided 95% UB of the hazard ratio comparing them is ≤1.35 (see section 9.3).

The time to treatment failure is defined as the planned visit week at which the first event among disease progression or virologic failure occurs. For virologic failure, this visit week is the time at which the first measurement of the two successive measurements was taken. The earliest failure time is week 16. The possible failure times and censoring times, corresponding to the planned visit weeks, for virologic failure are weeks 16, 24, 32, and every 8 weeks thereafter. For disease progression, this visit week is the time at which the onset of an AIDS-defining OI or malignancy occurred. For this endpoint criterion, the earliest possible failure time is week 12. The possible failure times and censoring times, corresponding to the planned visit weeks, for disease progression are weeks 12, 16, 20, 24, and every 8 weeks thereafter. Participants lost to follow-up are considered censored in the analysis at the planned visit week of the last clinic visit. Missing plasma HIV-1 RNA values will not be considered as providing evidence of reaching primary endpoint.

When a first plasma HIV-1 RNA measurement is ≥1000 copies/mL but the participants have not returned for a confirmatory level, this event is counted as a virologic failure endpoint at the time of the last visit.

NOTE: If participants develop an AIDS-defining illness in the absence of plasma HIV-1 RNA data, the participants will be allowed to switch treatment steps at the discretion of the site investigator.

- - - 1. **Updates Following Closure of Arm 1B at May 2008 DSMB Interim Review**

The primary analysis specifications listed above apply only to the comparison of Arm 1C to Arm 1A. The primary analysis of Arm 1B versus Arm 1A was based upon the comparisons performed for the May 2008 interim review and reflect the analysis specifications for interim monitoring as outlined in section 9.4 above.

9.5.2 Secondary Analyses

- - - 1. **Original Study Design**

The primary analysis will be repeated, treating the time to an endpoint as interval censored if participants have missed a visit or visits prior to observed virological or clinical failure. The interval will be taken to be the time from the last visit prior to the endpoint and the time of the primary endpoint. For this analysis, participants who have one viral load measurement that meets the failure criteria but do not return for a confirmatory measurement will be treated in two ways: as failures and as censored at the time of the last visit.

- The primary comparison will be repeated in an as-treated analysis, i.e., censoring participants when they discontinue initial study regimen.
- The distribution of the time from randomization to discontinuation of initial ARV therapy will be compared using the log-rank test, stratified by country and screening plasma HIV-1 RNA (< versus ≥100,000 copies/ml). Hazard ratios and 95% CI will be calculated using a stratified proportional hazards regression.
- The distribution of the time from randomization to CD4+ cell count <100/mm^3^ will be compared using the log-rank test, stratified by country and by screening plasma HIV-1 RNA. Participants whose CD4+ cell counts never achieve a level ≥100/mm^3^ will be assigned a failure time of 48 weeks, as will participants whose CD4+ cell **counts** drop to <100/mm^3^ before 48 weeks. Hazard ratios and 95% CI will be calculated using a stratified proportional hazards regression.
- A Wei-Johnson global test that makes use of all of the CD4+ cell count data collected over time will be performed on the change in counts from baseline. The test will be inverted to get a point estimate for the difference in average level of CD4+ **cell** count (averaged over measurements) and the 95% CI.
- The distribution of the time from start of treatment to the first dose-modifying or Grade 3 or 4 **adverse event** will be compared using the log-rank test, stratified by country and by screening plasma HIV-1 RNA (< versus ≥100,000 copies/mL). Hazard ratios and 95% CI will be calculated using a stratified proportional hazards regression.
- Estimate and compare proportions of participants in each arm with plasma HIV-1 RNA <400 copies/mL at weeks 24 and 48 - **ITT** analysis; estimation will use pointwise exact 95% CI on the binomial proportion end point (stratified Cochran-Mantel-Haenzsel tests).
- Estimate TLOVR using the method of Kaplan and Meier and compare between arms using log-rank tests and multivariate Cox regression modeling to incorporate appropriate covariate adjustment.
  - - 1. Update Following Closure of Arm 1B at May 2008 DSMB Interim Review

**The secondary analyses above refer to the pairwise comparison of Arm 1C to Arm 1A. Secondary analyses of Arm 1B to Arm 1A will use follow-up until the closing of Arm 1B (i.e., study visits up to May 23, 2008). Significance level for secondary analyses hypothesis tests of Arm 1B to Arm 1A will be specified in the statistical analysis plan.**

## 10.0 DATA COLLECTION AND MONITORING AND ADVERSE EVENT REPORTING

### 10.1 Records to Be Kept

CRFs will be provided for each participant. Participants must not be identified by name on any CRFs. Participants will be identified by the patient identification number (PID) and study identification number (SID) provided by the ACTG DMC upon randomization at study entry.

### 10.2 Role of Data Management

10.2.1 Instructions concerning the recording of study data on CRFs will be provided by the ACTG DMC. Each **site** is responsible for submitting the data in a timely fashion.

10.2.2 It is the responsibility of the ACTG DMC to assure the quality of computerized data for each ACTG study. This role extends from protocol development to generation of the final study databases.

### 10.3 Clinical Site Monitoring and Record Availability

10.3.1 Site monitors under contract to the National Institute of Allergy and Infectious Diseases (NIAID) will visit participating clinical research sites to review the individual participant records, including consent forms, CRFs, supporting data, laboratory specimen records, and medical records (physicians’ progress notes, nurses’ notes, participants’ hospital charts), to ensure protection of study participants, compliance with the protocol, and accuracy and completeness of records. The monitors also will inspect sites’ regulatory files to ensure that regulatory requirements are being followed and sites’ pharmacies to review product storage and management.

10.3.2 The investigator will make study documents (e.g., consent forms, drug distribution forms, CRFs) and pertinent hospital or clinic records readily available for inspection by the local IRB, the site monitors, NIAID, OHRP, the pharmaceutical **supporter**s, or the **supporter**s’ designee for confirmation of the study data.

### 10.4 Expedited Adverse Event (EAE) Reporting

The expedited adverse event (EAE) reporting requirements, definitions for this study, and the methods for expedited reporting of adverse events (AEs) to the DAIDS RCC Safety Office are defined in “The Manual for Expedited Reporting of Adverse Events to DAIDS” (DAIDS EAE Manual), dated May 6, 2004. The DAIDS EAE Manual is available on the RCC website: [**http://rcc.tech-res.com/safetyandpharmacovigilance/**](http://rcc.tech-res.com/safetyandpharmacovigilance/).

AEs reported on an expedited basis must be documented on the DAIDS Expedited Adverse Event Reporting Form (EAE Reporting Form available on the RCC website: [**http://rcc.tech-res.com/safetyandpharmacovigilance/**](http://rcc.tech-res.com/safetyandpharmacovigilance/)) and submitted to the DAIDS Safety Office through the RCC.

If an EAE or pregnancy occurs between scheduled visits, complete and key all CRFs related to that EAE or pregnancy at the time the EAE is reported or the pregnancy is determined.

This study uses the Standard Level of expedited AE reporting as defined in the DAIDS EAE Manual. This level of reporting is required during the entire study follow-up period (from enrollment until the study participant finishes or leaves the study). Infants born to mothers enrolled in A5175 will be followed for the same reporting requirements as above. Additionally, standard reporting requirements for the infant will continue until the infant is 18 months of age.

The study agents that must be considered in determining relationships of AEs requiring expedited reporting to DAIDS are: atazanavir (ATV), Combivir® (3TC/ZDV), didanosine (ddI-EC), Truvada® (FTC/TDF), emtricitabine (FTC), lamivudine (3TC), stavudine (d4T), tenofovir (TDF), zidovudine (ZDV), efavirenz (EFV), and nevirapine (NVP).

NOTE: ATV-related, asymptomatic hyperbilirubinemia (Grade 4 total bilirubin) or jaundice without LFT elevation (Grade ≤ 1 AST, ALT, and alkaline phosphatase) does not require Expedited Adverse Event (EAE) reporting.

IRIS events (for a definition see Appendix II, section 4.0) are not caused by a specific or individual drug, but rather are the result of being on a successful ARV treatment regimen. Therefore, IRIS events do not fulfill the DAIDS EAE reporting criteria and reporting the event to the DAIDS on an expedited basis is not required, even if the event is severe in intensity.

The Division of AIDS Table for Grading the Severity of Adult and Pediatric Adverse Events (DAIDS AE Grading Table), Version 1.0, December 2004 **(Clarification dated 2009)**, must be used and is available on the RCC website at [**http://rcc.tech-res.com/safetyandpharmacovigilance/**](http://rcc.tech-res.com/safetyandpharmacovigilance/).

In addition to submitting EAE information to the DAIDS Safety Office through the RCC, the site investigator is required to submit AE information as required by local regulatory or other local authority.

After the end of the protocol-defined EAE reporting period stated above, sites must report serious, unexpected, clinical suspected adverse drug reactions if the study site staff becomes aware of the event on a passive basis, i.e., from publicly available information.

## 11.0 STUDY PARTICIPANTS

### 11.1 Institutional Review Board (IRB) Review and Informed Consent

This protocol and the informed consent documents (see Appendices VII, VIII, and IX) and any subsequent modifications will be reviewed and approved by the IRB, and other country-specific review boards that are responsible for oversight of the study. A signed consent form will be obtained from the participant. The consent form will describe the purpose of the study, the procedures to be followed, and the risks and benefits of participation. A copy of the consent form will be given to the participant, and this fact will be documented in the participant’s record.

### 11.2 Participant Confidentiality

All laboratory specimens, evaluation forms, reports, and other records that leave the site will be identified by coded number only to maintain participant confidentiality. All hard copy records will be kept locked. All computer entry and networking programs will be done with coded numbers only. Clinical information will not be released without written permission of the participant, except as necessary for monitoring by IRB, the NIAID, the OHRP, or the pharmaceutical **supporter**s or their designees.

### 11.3 Study Discontinuation

The study may be discontinued at any time by the **ACTG,** NIAID, the pharmaceutical supporters, **the OHRP**, or other government agencies as part of their duties to ensure that research participants are protected.

## 12.0 PUBLICATION OF RESEARCH FINDINGS

Publication of the results of this trial will be governed by ACTG policies. Any presentation, abstract, or manuscript will be made available for review by the pharmaceutical sponsors prior to submission.

## 13.0 BIOHAZARD CONTAINMENT

As the transmission of HIV and other blood-borne pathogens can occur through contact with contaminated needles, blood, and blood products, appropriate blood and secretion precautions will be employed by all personnel in the drawing of blood and shipping and handling of all specimens for this study, as currently recommended by the Centers for Disease Control and Prevention and the National Institutes of Health.

All infectious specimens will be transported **using** **packaging mandated by CFR 42 Part 72. Please refer to instructions detailed in the International Air Transport Association (IATA) Dangerous Goods Regulations.**

## 14.0 REFERENCES

Anderson R, Meyer W, Balch F, et al. Anti-viral effects of stavudine (d4T) therapy. [Abstract WeB1010] In: Abstracts of the Eighth International Conference on AIDS/III STD World Congress, The Netherlands; 1993.

Autran B, Carcelain G, Li TS, et al. Positive effects of combined antiretroviral therapy on CD4+ T cell homeostasis and function in advanced HIV disease. Science 1997;277:112-6.

Brodt HR, Kamps BS, Gute P, Knupp B, Staszewski S, Helm EB. Changing incidence of AIDS-defining illnesses in the era of antiretroviral combination therapy. AIDS 1997;11(14):1731-8.

Clarke S, Harrington P, Barry M, Mulcahy F. The tolerability of efavirenz after nevirapine-related adverse events. Clin Inf Dis 2000;31:806-7.

D'Aquila RT, Hughes MD, Johnson VA, et al. Nevirapine, zidovudine, and didanosine compared with zidovudine and didanosine in patients with HIV-1 infection. A randomized, double-blind, placebo-controlled trial. National Institute of Allergy and Infectious Diseases AIDS Clinical Trials Group Protocol 241 Investigators. Ann Intern Med 1996;124(12):1019-30.

Dore GJ, Li Y, McDonald A, Ree H, Kaldo JM. Impact of highly active antiretroviral therapy on individual AIDS-defining illness incidence and survival in Australia. JAIDS 2002;29:388-395.

Eastman PS, Shapiro DE, Coombs RW, et al. Maternal viral genotypic zidovudine resistance and infrequent failure of zidovudine therapy to prevent perinatal transmission of human immunodeficiency virus type 1 in Pediatric AIDS Clinical Trials Group Protocol 076. J Infect Dis 1998; 177:557-564.

Ekpini RA, Nkengasong JN, Sibailly T, et al. Changes in plasma HIV-1-RNA viral load and CD4 cell counts, and lack of zidovudine resistance among pregnant women receiving short-course zidovudine. AIDS 2002; 16:625-30.

Eron JJ, Yetzer ES, Ruane PJ, et al. Efficacy, safety, and adherence with a twice-daily combination lamivudine/zidovudine tablet formulation, plus a protease inhibitor, in HIV infection. AIDS 2000;14:671-81.

Eshleman SH, Mracna M, Guay LA, et al., Selection and fading of resistance mutations in women and infants receiving nevirapine to prevent HIV-1 vertical transmission (HIVNET 012). AIDS 2001;15:1951-7.

Eshleman SH, Guay LA, Mwatha A, et al. Characterization of nevirapine (NVP) resistance mutations in women with subtype A vs. D HIV-1 6-8 weeks after single dose NVP (HIVNET 012). J Acquir Immune Defic Syndr 2004;35:126-30.

Eshleman SH, Hoover DR, Chen S, et al. Nevirapine (NVP) resistance in women with subtype C, compared to subtypes A and D, after administration of single-dose NVP. J Infect Dis 2005;192:30-6.

Flys T, Nissley DV, Claasen CW, et al. Sensitive drug resistance assays reveal long-term persistence of HIV-1 variants with the K103N nevirapine (NVP) resistance mutation in some women and infants after single dose NVP: HIVNET 012. J Infect Dis 2005;192:24-9.

Gathe J, Podzamczer D, Johnson M, et al. Once-daily vs. twice-daily lopinavir/ritonavir in antiretroviral-naïve patients: 48-week results [Poster Number 570]. 11th Conference on Retrovirus and Opportunistic Infections (CROI); 2004 February 8-11; San Francisco, CA.

Gallant JE, Dejesus E, Arribas JR et al. Tenofovir DF, emtricitabine, and efanirenz vs. zidovudine, lamivudine, and efavirenz for HIV. N Eng J Med 2006:354:251-60.

Griffith BP, Brett-Smith H, Kim G, et al. Effect of stavudine on human immunodeficiency virus type 1 virus load as measured by quantitative mononuclear cell culture, plasma RNA, and immune complex-dissociated antigenemia. J Infect Dis 1996;173(5):1252-5.

Gulick RM, Mellors JW, Havlir D, et al. Treatment with indinavir, zidovudine, and lamivudine in adults with human immunodeficiency virus infection and prior antiretroviral therapy. N Engl J Med 1997;337:734-9.

Hammer SM, Squires KE, Hughes MD, et al. A controlled trial of two nucleoside analogues plus indinavir in persons with human immunodeficiency virus infection and CD4 cell counts of 200 per cubic millimeter or less. AIDS Clinical Trials Group 320 Study Team. N Engl J Med 1997;337(11):725-33.

Johnson JA, Li JF, Morris L, et al. Emergence of drug-resistant HIV-1 after intrapartum administration of single-dose nevirapine is substantially underestimated. J Infect Dis 2005;192:16-23.

Jourdain G, Ngo-Giang-Huong N, Le Coeur S, et al. Intrapartum exposure to nevirapine and subsequent maternal responses to nevirapine-based antiretroviral therapy. N Engl J Med 2004;351:229-40.

Kearney BP, Zong J, Begley J, et al. Bioequivalence of combination tenofovir DF/emtricitabine tablets for one-pill once daily administration [Poster No. 7.3]. 5th International Workshop on Clinical Pharmacology of HIV Therapy; 2004 April 1-3; Rome, Italy.

Kully C, Yerly S, Erb P, Kind C, Krautheim A, Perrin L, Rudin C. Codon 215 mutations in human immunodeficiency virus-infected pregnant women. Swiss Collaborative 'HIV and Pregnancy' Study. J Infect Dis 1999; 179:705-8.

Lecossier D, Shulman NS, Morand-Joubert L, et al. Detection of minority populations of HIV-1 expressing the K103N resistance mutation in patients failing nevirapine. J Acquir Immune Defic Syndr 2005;38:37-42.

Mellors J, Palmer S, Nissley D, et al. Low frequency NNRTI-resistant variants contribute to failure of efavirenz-containing regimens in NNRTI-experienced patients with negative standard genotypes for NNRTI mutations. Antiviral Therapy 2003;8:S150.

Molina JM, Gathe J, Lim PL, et al. Comprehensive resistance testing in antiretroviral-naïve patients treated with once-daily lopinavir/ritonavir plus tenofovir DF and emtricitabine: 48-week results from Study 418 [WePeB5701]. XV International AIDS Conference; 2004 July 11-16; Bangkok, Thailand.

Moore KHP, Shaw S, Laurent AL, et al. Lamivudine/ZDV as a combined formulation tablet: Bioequivalence compared with lamivudine and ZDV administered concurrently and the effect of food on absorption. J Clin Pharmacol 1999;39:593-605.

Murray HW, Squires KE, Weiss W, et al. Stavudine in patients with AIDS and AIDS-related complex: AIDS Clinical Trials Group 089. J Infect Dis 1995;171(Suppl.2):S123-30.

Palella FJ Jr, Delaney KM, Moorman AC, et al. Declining morbidity and mortality among patients with advanced human immunodeficiency virus infection. HIV Outpatient Study Investigators. N Engl J Med 1998;338:853-60.

Peterson EA, Ramirez-Ronda CH, Hardy WD, et al. Dose-related activity of stavudine in patients infected with human immunodeficiency virus. J Infect Dis 1995;171(Suppl.2):S131-9.

Podzamczer D, Consiglio E, Ferrer E, Gudiol F. Efavirenz associated with corticosteroids in patients with previous severe hypersensitivity reaction due to nevirapine (letter). AIDS 2000;14:331-2.

Podzamczer D, Gathe J, Johnson M, et al. Study 418: Once-daily vs. twice-daily lopinavir/r in antiretroviral-naïve patients: 24-week results [Abstract]. 9th European AIDS Conference; 2003 October 26-29; Warsaw, Poland.

Robbins GK, De Gruttola V, Shafer RW, et al. Comparison of sequential three-drug regimens as initial therapy for HIV-1 infection. N Engl J Med 2003; 349:2293-03.

Soriano V, Dona C, Barreiro P, Gonzalez-Lahoz J. Is there cross-toxicity between nevirapine and efavirenz in subjects developing rash? (letter). AIDS 2000;14:1672-3.

Spruance SL, Pavia AT, Mellors JW, et al. Clinical efficacy of monotherapy with stavudine compared with zidovudine in HIV-infected, zidovudine-experienced patients. A randomized, double-blind, controlled trial. Bristol-Myers Squibb Stavudine/019 Study Group. Ann Intern Med 1997;126(5):355-63.

Staszewski S, Morales-Ramirez J, Tashima KT, et al. Efavirenz plus zidovudine and lamivudine, efavirenz plus indinavir and indinavir plus zidovudine and lamivudine in the treatment of HIV-1 infection in adults. N Engl J Med 1999;341:1865-73.

van Rompay KKA, Marthas ML, Lifson, JD, et al. Administration of 9-[2-(phosphonomethoxy)propyl]adenine (PMPA) for prevention of perinatal simian immunodeficiency virus infection in rhesus macaques. AIDS Res Hum Retroviruses 1998;14:761-73.

Welles SL, Pitt J, Colgrove R, et al. HIV-1 genotypic zidovudine drug resistance and the risk of maternal-infant transmission in the Women and Infants Transmission Study. AIDS 2000; 14:263-271.

#### APPENDIX I: VIROLOGY STUDY PLAN

Primary Investigators: Susan H. Eshleman, M.D., Ph.D.

Susan A. Fiscus, Ph.D.

1.0 OBJECTIVES

1.1 To characterize the patterns and rates of ARV drug resistance during ARV therapy in **RLS**.

1.2 To compare the patterns of ARV drug resistance mutations among different HIV-1 subtypes.

1.3 To determine whether response to ARV treatment is associated with HIV-1 subtype.

1.4 To assist with the transfer of relevant virology laboratory technology to investigators in diverse settings.

2.0 BACKGROUND

Many patients receiving ARV regimens for treatment of HIV-1 infection are unable to maintain effective suppression of HIV-1 replication. Incomplete suppression of viral replication in the presence of ARV regimens often leads to selection of HIV-1 variants with drug resistance mutations in blood. Because many resistance mutations also confer cross-resistance to other ARV drugs in the same class, emergence of drug resistance may severely limit a patient’s future treatment options.

As use of ARV drugs for prevention and treatment of HIV-1 infection increases in **RLS**, ARV drug resistance will likely become more prevalent. A variety of regional factors may influence the effectiveness of ARV regimens and the emergence of resistant strains. The effectiveness of treatment programs will be influenced by cultural, behavioral, and logistical factors that vary from one region to another. There are also regional differences in the prevalence of other infectious diseases (e.g., TB, hepatitis) and other clinical illnesses, which could influence immunologic and other factors important in viral containment. Such comorbidities are also associated with regional differences in use of other medications, which may in turn influence the activity and PK of ARV drugs. Regional differences in host genetics may also influence response to ARV regimens, and regional differences in HIV-1 strains may not only influence the susceptibility of the virus to ARV drugs but also the emergence of resistant strains under treatment. Finally, in **RLS**, regimens used to prevent MTCT transmission of HIV-1 are likely to include single drug regimens (e.g., ZDV monotherapy or single-dose NVP), increasing the probability that resistance will emerge.

Worldwide, at least three distinct lineages of HIV-1 exist (groups M, N, and O) and each represents a separate introduction of primate lentiviruses into human populations (Hu et al. 1996; Robertson 1999; Robertson 2000). The vast majority of HIV-1 infections are due to group M (Major). Within group M, 10 distinct subtypes have been identified. Most HIV-1 infections in Western Europe and North America are subtype B. In South America subtype B is prevalent, but subtype F and C infections also occur. In Southeast Asia subtype B is common among intravenous drug users but subtype CRF01 infection is widespread among persons who acquire HIV-1 by sexual contact (Ou et al. 1993). Africa has the greatest intracontinent HIV-1 genetic diversity (subtypes A, C, D, G, H, J, and K), but distinct geographic patterns of subtype distribution exist within Africa. Most HIV-1 infections in southern Africa are subtype C, and subtype C is prevalent in India and parts of Asia. Approximately half of all HIV-1 infections worldwide are subtype C.

Although data are limited, some studies suggest that the natural susceptibility of HIV-1 to ARV drugs may be influenced by subtype (Descamps et al. 1998; Apetrei et al. 1998). Polymorphisms associated with drug resistance are frequently detected in ARV drug-naïve individuals with non-subtype B infection (Pieniazek et al. 2000), and HIV-1 subtype may also influence the pattern of resistance mutations selected by ARV drugs. For example, in Brazil, patients with subtype F who failed PI-containing regimens generally acquired the G48V and V82A/F resistance mutations, whereas patients with subtype B acquired predominantly the L90M mutation (Caride et al. 2001). Furthermore, in the Ugandan HIVNET 012 trial, the rate of NVP resistance following single dose NVP prophylaxis was different for women with subtype A versus D HIV-1 (Eshleman et al. 2001). This suggests that the rates of resistance emerging during ARV drug exposure may vary from region to region, depending on which subtypes are prevalent. HIV-1 subtype may also affect viral fitness in the presence or absence of drug resistance mutations. Such differences could in turn influence the dynamics of emergence of drug resistance following ARV drug exposure.

Some data suggest that HIV-1 subtype influences ARV response. A retrospective study comparing 50 patients with subtype B and 50 patients with non-B subtypes did not find a difference in virologic responses to HAART (Loveday et al. 2001). However, that study did not compare the response among patients with different non-B subtypes. In another study, 79 drug-naïve African patients with different non-B subtypes had a similar response to HAART (Frater et al. 2001). However, the number of patients in that study with each subtype was small. Furthermore, in both studies, different ARV regimens were used to treat individual patients; this may have made it difficult to detect a subtype-based difference in response to treatment.

3.0 RATIONALE

To date, almost all studies of HIV-1 drug resistance have been performed for subtype B, the most common subtype in North America and Europe. In contrast, there is remarkably little information on drug resistance in other subtypes. Research on this topic in cohorts infected with non-B subtype HIV-1 is important for two reasons: (1) the prevalence of non-B subtype is increasing in the United States and other regions where ARV drugs are widely used; and (2) the availability and use of ARV drugs is growing throughout the world, where most infections are caused by non-B subtype HIV-1.

A5175 provides a unique opportunity to study ARV drug resistance in **RLS**. The design will allow a comparison of ARV resistance rates across the study sites for each treatment regimen. Data collected may also help identify other regional variables that influence resistance rates, since most participants are expected to be infected with non-B subtypes, with different subtype distributions at each site (Table 1).

Table 1. Predominant HIV-1 Subtypes Found in Countries with A5175 Sites

| Country | Predominant Subtypes (approximate %) | Reference |
| --- | --- | --- |
| Brazil | B (80%), F (14%), C (3%) | Bongertz et al. 2000  Tanuri et al. 1999 |
| Haiti | B (most) | LANL HIV Database |
| India | C (80%-95%), B (2%-10%), A (2%) | Sahni et al. 2002, Gadkari et al. 1998 |
| Malawi | C (most) | Candotti et al. 2001 |
| Peru | B (98%) | Russell et al. 2000 |
| South Africa | C (most) | Bredell et al. 2002  Van Harmelen et al. 1999 |
| Thailand | CRF01_AE (80%-95%), B (5%-20%) | Nguyen et al. 2002  Subbarao et al. 1998  Ruxrungtham et al. 2001 |
| Zimbabwe | C (70%), B (12%), A (12%), D (7%) | Obi et al. 1997  Shafer et al. 1997 |
| United States | B (most) | Perrin et al. 2003 |

4.0 EVALUATIONS

HIV-1 Resistance Testing

Currently, resources are not available at most A5175 **non-U.S. CRS**s to perform resistance testing in real time. Therefore, all of the resistance testing planned in A5175 will be performed retrospectively, and results will not be used to guide the treatment of individual participants.

The Johns Hopkins University (JHU) HPTN Network Laboratory (NL) will coordinate the resistance testing in A5175. HIV-1 genotyping will be performed using the Celera Diagnostics ViroSeq HIV-1 Genotyping System. Regional international laboratories will perform genotypic resistance testing, in addition to the HPTN NL. Laboratories in Africa (Johannesburg) and Latin America (Rio de Janeiro) have already been certified to perform resistance testing. Training is ongoing in Pune and Chennai, India, for analysis of samples from Asia. Samples from U.S. sites and some non-US sites will also be tested at the HPTN NL or at a designated ACTG VSL (to be determined).

Selected samples tested at international laboratories will be analyzed in duplicate at the HPTN NL or at a designated ACTG VSL for quality control. In addition to the core analysis (see below) the HPTN NL may also analyze samples collected at other time points to examine the timing of emergence of resistance in selected cases, and will perform detailed genotypic analyses of cases of suspected HIV transmission. Other laboratories may participate in specialized virologic testing, or may join as additional genotyping laboratories at a future date. Some specialized testing (e.g., fitness or phenotypic resistance testing) may be performed in commercial laboratories. See the A5175 and A5185s MOPS.

HIV-1 genotyping of reverse transcriptase and protease will be performed on the following samples:

- Confirmed virologic failure (plasma HIV-1 RNA ≥1000 copies/mL) of the Step 1 treatment regimen with initial quantitation at week 16 or later.
- Study entry for participants who subsequently have confirmed virologic failure of the Step 1 treatment regimen.
- Study entry for a control group of an equal number of participants who did not fail Step 1 treatment.
- Entry into Step 2.

Genotyping may also be performed on samples from participants prematurely discontinuing treatment or going off study.

NOTE: Baseline and time-of-failure samples will be analyzed from all participants who fail Step 1 treatment. Analysis of all treatment failures will improve our ability to study the patterns of reverse transcriptase mutations in low prevalence subtypes. If 50% of participants fail Step 1 treatment, there will be 563 treatment failures distributed across the twelve **non-U.S. CRS**s (approximately 47 failures per site).

Other Resistance Studies

A subset of samples will be examined using phenotypic resistance and viral fitness (replication capacity) assays. For example, phenotypic resistance assays and fitness assays may be performed to compare the baseline drug susceptibilities and replication capacities of different non-B subtypes. Phenotypic assays may also be performed for further analysis of failure samples that lack known resistance mutations. To date, resistance mutations have been identified and characterized almost exclusively in subtype B. It is possible that participants enrolled in A5175 who have non-B subtype HIV may fail therapy due to resistance, yet lack known mutations. Those participants may have virus with novel (as of yet unidentified) drug resistance mutations, which would be genotypically "wild type," but phenotypically resistant.

HIV-1 Subtype Determination

Phylogenetic subtyping will be coordinated by the HPTN NL. Data generated by the automated DNA sequence analysis of reverse transcriptase and protease for detection of ARV drug resistance mutations will be used to determine HIV-1 subtype. The subtype of each virus will be determined by construction of inferred phylogenetic trees of the reverse transcriptase and protease DNA sequences. This will allow us to explore the relationship between subtype, treatment response, and drug resistance. Additional analyses of sequences from other regions of the HIV-1 genome may be performed for more detailed subtype determination and analysis of inter-subtype recombination.

Advancing Technology for Drug Resistance Testing in Resource-Limited Countries

A5175 will help advance technology for HIV-1 drug resistance testing in resource-limited countries.

There are two commercial systems for HIV-1 genotyping: the Celera Diagnostics ViroSeq HIV-1 Genotyping System and the Bayer TRUGENE assay. These systems were both developed and optimized for analysis of subtype-B HIV-1. The FDA-cleared version of the ViroSeq system has been shown to perform well for all group M subtypes. This assay is currently being used for resistance testing at the HPTN NL as well as in the A5175 laboratories in Rio de Janeiro and Johannesburg.

The A5175 protocol team will collaborate with existing ACTG working groups to facilitate transfer of resistance testing technology to A5175 sites and other ACTG international sites. This may include detailed comparisons of performance characteristics of the ViroSeq, TRUGENE, and less expensive assays with non-B subtypes. Different assays may be appropriate for different international testing sites.

5.0 ANALYSES

- At study entry, amino acid substitutions in reverse transcriptase and protease for participants who have virologic failure on the Step 1 regimen will be compared with participants who do not have virologic failure. Virologic failure will be defined as confirmed plasma HIV-1 RNA ≥1000 copies/mL, with the first measurement at week 16 or later.
- Time to Step 1 treatment failure for women who received ARV prophylaxis for MTCT transmission in previous pregnancies compared with women who have not been previously exposed to ART **for prevention of MTCT**.
- Time to Step 1 treatment failure by HIV-1 subtype.
- The pattern of reverse transcriptase and protease amino acid substitutions at the time of confirmed Step 1 treatment failure by HIV-1 subtype.
- Quantitative measure of future drug options as determined by mutation pattern in reverse transcriptase and protease of participant-derived virus at the time of virologic failure and at the time of ARV treatment switch for failure. A possible metric to measure future drug options after 48 weeks of strategy intervention is that of Jiang et al. (2003).
- Change in future drug options from baseline to time of failure will be compared using the Wilcoxon rank-sum test with stratification by country and by screening HIV-1 RNA at a two-sided 0.05 significance level. For those who are undetectable throughout the study, we assume no change in future drug options scores.

6.0 REFERENCES

Anderson JP, Bradac JA, Carr JK, Foley B, Funkhouser RK, et al. HIV-1 nomenclature proposal. Science 2000; 288:55-6.

Apetrei C, Descamps D, Collin G, Loussert-Ajaka I, Damond F, Duca M, Simon F, Brun Vezinet F. Human immunodeficiency virus type 1 subtype F reverse transcriptase sequence and drug susceptibility. J Virology 1998;72:3534-38.

Bongertz V, Bou-Habib DC, Brigido LF, Caseiro M, Chequer PJ, Couto-Fernandez JC, Ferreira PC, et al. HIV-1 diversity in Brazil: genetic, biologic, and immunologic characterization of HIV 1 strains in three potential HIV vaccine evaluation sites. Brazilian Network for HIV Isolation and Characterization. J Acquir Immune Defic Syndr 2000;23:184-93.

Bredell H, Hunt G, Casteling A, Cilliers T, Rademeyer C, Coetzer M, Miller S, et al. HIV-subtype A, D, G, Ag and unclassified sequences identified in South Africa. AIDS Res Hum Retroviruses 2002, 18:681-3.

Candotti D, Mundy C, Kadewele G, Nkhoma W, Bates I, Allain JP: Serological and molecular screening for viruses in blood donors from Ntcheu, Malawi: high prevalence of HIV-1 subtype C and of markers of hepatitis B and C viruses. J Med Virol 2001, 65:1-5.

Caride E, Hertogs K, Larder B, Dehertogh P, Brindeiro R, Machado E, de Sa CA, et al. Genotypic and phenotypic evidence of different drug-resistance mutation patterns between B and non-B subtype isolates of human immunodeficiency virus type 1 found in Brazilian patients failing HAART. Virus Genes 2001;23:193-202.

Descamps D, Apetrei C, Collin G, Damond F, Simon F, Brun-Vezinet F. Naturally occurring decreased susceptibility of HIV-1 subtype G to protease inhibitors. AIDS 1998;12:1109-11.

Eshleman SH, Becker-Pergola G, Deseyve M, Guay LA, Mracna M, Fleming T, et al. Impact of HIV-1 subtype on women receiving single dose NVP prophylaxis to prevent HIV-1 vertical transmission (HIVNET 012). J Infect Dis 200l; 184:914-7.

Frater AJ, Beardall A, Ariyoshi K, Churchill D, Galpin S, Clarke JR, et al. Impact of baseline polymorphisms in RT and protease on outcome of highly active antiretroviral therapy in HIV-1-infected African patients. AIDS 2001; 15:1493-502.

Gadkari DA, Moore D, Sheppard HW, Kulkarni SS, Mehendale SM, Bollinger RC. Transmission of genetically diverse strains of HIV-1 in Pune, India. Indian J Med Res 1998; 107:1-9.

Hu DJ, Dondero TJ, Rayfield MA, George R, Schochetman G, Jaffe HW, et al. The emerging genetic diversity of HIV. JAMA 1996; 275: 210-6.

Jiang H, Deeks S, Kuritzkes D, Lallemant M, Katzenstein D, Albrecht M, De Gruttola V. Assessing resistance costs of antiretroviral therapies via measures of future drug options. J Inf Dis, 2003; 188:1001-08.

Los Alamos National Laboratory (LANL) HIV Database, http://www.hiv.lanl.gov/content/hiv-db/mainpage.html.

Loveday C, Burke A, van Hooff F, Johnson M. Equivalence in virologic responses to HAART in patients with HIV-1 subtype non-B infections: a case-controlled study (n=100). 8th Conf. on Retroviruses and Opportunistic Infections [Abstract #526]. 2001; Chicago, IL.

Nguyen L, Hu DJ, Choopanya K, Vanichseni S, Kitayaporn D, Van Griensven F, et al. Genetic analysis of incident HIV-1 strains among injection drug users in Bangkok: Evidence for multiple transmission clusters during a period of high incidence. J Acquir Immune Defic Syndr 2002; 30:248-56.

Obi CL, McAdoo HP, Onigbinde AO, Murray M, Tswana SA, Moyo SR. Subtypes of HIV-1 and the impact of dual infections of HIV-1 and measles virus on micronutrient levels of pregnant women in Harare, Zimbabwe. Cent Afr J Med 1997; 43:165-72.

Ou CY, Takebe Y, Weniger BG, Luo CC, Kalish ML, Auwanit W, et al. Independent introduction of two major HIV-1 genotypes into distinct high-risk populations in Thailand. Lancet 1993; 341, 1171-4.

Perrin, L, Kaiser L, Yerly, S. Travel and the spread of HIV-1 genetic variants. Lancet 2003 3:22-27.

Pieniazek D, Rayfield M, Hu DJ, Nkengasong J, Wiktor SZ, Downing R, et al. Protease sequences from HIV-1 group M subtypes A-H reveal distinct amino acid mutation patterns associated with protease resistance in protease inhibitor-naive individuals worldwide. HIV Variant Working Group. AIDS 2000; 14:1489-95.

Robertson DL, Anderson JP, Bradac JA, Carr JK, Foley B, Funkouser RK, et al. HIV-1 Nomenclature Proposal: A Reference Guide to HIV-1 Classification in Human Retroviruses and AIDS: a Compilation and Analysis of Nucleic and Amino Acid Sequences, Los Alamos National Laboratory, Los Alamos, NM. pp 492-505. 1999.

Russell Kl, Carcamo C, Watts DM, Sanchez J, Gotuzzo E, Euler A, et al. Emerging genetic diversity of HIV-1 in South America. AIDS 2000; 14:1785-91.

Ruxrungtham K, Phanuphak P. Update on HIV/AIDS in Thailand. J Med Assoc Thai 2001; 84 Suppl 1:S1-17.

Sahni AK, Prasad VV, Seth P. Genomic diversity of human immunodeficiency virus type-1 in India. Int J S AIDS 2002; 13:115-8.

Shafer RW, Eisen JA, Merigan TC, Katzenstein DA. Sequence and drug susceptibility of subtype C reverse transcriptase from human immunodeficiency virus type 1 seroconverters in Zimbabwe. J Virol 1997; 71:5441-8.

Subbarao S, Limpakarnjanarat K, Mastro TD, Bhumisawasdi J, Warachit P, Jayavasu C, et al. HIV type 1 in Thailand, 1994-1995: persistence of two subtypes with low genetic diversity. AIDS Res Hum Retroviruses 1998; 14:319-27.

Tanuri A, Swanson P, Devare S, Berro OJ, Savedra A, Costa LJ, et al. HIV-1 subtypes among blood donors from Rio de Janeiro, Brazil. J Acquir Immune Defic Syndr Hum Retrovirol 1999; 20:60-6.

Van Harmelen JH, Van Der Ryst E, Loubser AS, York D, Madurai S, Lyons S, et al. A predominantly HIV type 1 subtype C-restricted epidemic in South African urban populations. AIDS Res Hum Retroviruses 1999; 15:395-8.

#### APPENDIX II: OPPORTUNISTIC INFECTIONS AND IMMUNE RECONSTITUTION STUDY PLAN

Primary Investigators: Judith Silverstein Currier, M.D., M.Sc.

Robert C. Bollinger, M.D., M.P.H.

##### 1.0 OBJECTIVES

1. To characterize and compare the occurrence and outcomes of OIs **and co-infections** in HIV-1-infected persons in **RLS**.

2. To characterize immune reconstitution syndromes **(IRS)** observed during ARV treatment in different geographic settings.

3. To evaluate factors associated with the development of **IRS** during ARV therapy.

4. To examine the relationships between development of an **IRS** and altered response to ARV treatment.

5. To compare the effects of ARV regimens that include 3TC, FTC, and FTC + TDF on HBV replication.

6. To examine the contribution of HBV flares to hepatotoxicity during HAART.

##### 2.0 BACKGROUND

The clinical benefit of ARV treatment in reducing the risk of disease progression has been best demonstrated for those with a CD4+ **cell** count <200 cells/mm^3^. While it is clear that the use of ARV treatment among patients with low CD4+ cell counts may be associated with the subsequent development of atypical presentations of OIs, the benefits of initiating ARV treatment in patients with advanced HIV-1 infection clearly outweigh the low risk of developing these rare conditions.

The benefit of initiating ARV treatment in the setting of an acute OI includes improvement of immune function, which would potentially contribute to faster resolution of the OI. The beneficial impact of starting ARV treatment during an acute OI has been best demonstrated for OIs for which there are no effective therapies. Reports detailing the resolution of cryptosporidiosis, microsporidiosis, progressive multifocal leukoencephalopathy (PML), and Kaposi sarcoma after the initiation of ARV treatment provide evidence that improving immune function can lead to improved outcome in the setting of an acute OI (Carr et al. 1998; Murdaca et al. 2002).

Immune reconstitution **inflammatory** syndrome (IRIS), also known as immune reconstitution syndrome (IRS) or immune restoration disease (IRD), ha**s** been documented to complicate the management of patients who have recently initiated ARV treatment (Shelburne et al.; DeSimone et al., 2000). IRISs have been described for mycobacterial infections (both *Mycobacterium avium* complex [MAC], and *M. tuberculosis* (MTB), *Pneumocystis carinii* pneumonia (PCP), toxoplasmosis, cryptococcal infection, and PML (Phillips et al., 1999; Navas et al., 2002; Race et al.; 1998; Dworkin et al., 1998; Wislez et al. 2001; Lanzafame et al., 1999; Foudraine et al. 1998; Miralles et al. 2001; Cinque et al.2001; Giudici et al. 2000). An IRIS is characterized by high fever and worsening of the clinical manifestations of the OI. In several case reports in the literature, successful management has included continuing ARV treatment with the addition of anti-inflammatory agents including corticosteroids. In most of these cases the IRISs have developed in patients with a CD4+ cell counts of <50/mm^3^ and no evidence of the OI at the time ARV treatment was initiated (French et al. 2000; Hassell et al. 2000). In some cases, these syndromes were thought to reflect an immune reaction to a subclinical infection (Race et al. 1998; Phillips et al. 2000; Worrell et al. 2001; Fox et al. 1999).

Severe inflammatory reactions have also been reported after HAART was started in patients with an established OI. Wislez et al. (2001) reported three patients with acute PCP that initially improved with anti-PCP and adjunctive steroid therapy, but these patients subsequently developed respiratory failure (and no new pathogens were identified) 7 to 17 days after the start of HAART. With an interruption in HAART and the use of steroids, all three patients recovered. In A5175, participants with a history of an established OI at entry are required to be stable on therapy prior to enrollment.

The high prevalence of MTB infection in the countries where A5175 will be conducted suggests that TB IRS will be frequent. Previous studies have reported a higher rate (36%) of paradoxical reaction (IRIS) among HIV-1 positive patients with MTB who were started on ARV treatment compared with those not started on ARV treatment and to patients with MTB who were not HIV infected. Fishman et al. (2000) reported transient worsening on chest radiography in 14 (45%) of 31 AIDS patients receiving ARV treatment, including seven patients (23%) who showed severe worsening. Recent WHO guidelines (see the A5175 and A5185s MOPS) for the management of tuberculosis and ARV treatment recommend that ARV treatment be delayed in patients with active tuberculosis, unless the risk of HIV disease progression is very high (as defined by a CD4+ **cell** count of <50 cells/mm^3^). For those with CD4+ cell counts between 50 and 200, an initial 2-month course of anti-MTB therapy is recommended prior to starting ARV treatment.

To date there are limited prospectively-collected data to support these recommendations. A5175 will provide an opportunity to assess the outcome of TB among participants who initiate ARV treatment.

Hepatitis B

Chronic infection with HBV is a growing cause of morbidity and mortality worldwide (Lee et al. 1997). HBV/HIV coinfection is common because of shared modes of transmission. The natural history of HBV infection is influenced in the presence of HIV coinfection. Persistent HBV infection is likely to develop in HIV-infected patients, and reactivation may occur despite seroconversion of antibody to HBsAg.

Data on the seroprevalence of HBV infection are available from several sites involved in A5175. The seroprevalence for HBsAg among HIV-positive male patients in Malawi was estimated to be 16.9%. (Sutcliffe et al. 2002). Data from a seroprevalence study done at an STD clinic in Pune, India, reported a seroprevalence of 3.6% for HBsAg and 42% anti-HBc in the overall population, whereas, among the subset with HIV infection 69.4% were anti-HBc seropositive (Risbud et al. 2002).

3TC has well-established antiviral activity against HBV, and recent data provide evidence that FTC also inhibits HBV replication. 3TC resistance may limit the long-term efficacy of 3TC and FTC for the treatment of HBV in coinfected patients. More recently, the activity of TDF against HBV (including 3TC resistant strains) has been appreciated (Benhamou et al. 2002). A5175 provides an opportunity to prospectively collect samples for future testing of HBV DNA and compare the outcomes for participants who receive FTC and TDF with those who receive either 3TC or FTC alone.

3.0 RATIONALE

It is expected that MTB infection will be common among A5175 participants. All participants with active MTB will receive at least 2 weeks of MTB therapy prior to starting HAART and will be clinically stable at enrollment. Thus, A5175 will allow a prospective evaluation of the outcome of TB in participants who begin HAART after initiation of MTB treatment. The information gained by these studies will be important for the application of ARV treatment in areas of the world where coinfection with MTB and HIV-1 are common.

Although the prevalence of chronic infections such as MTB suggests that IRIS will be a significant complication of ARV treatment in **RLS**, to date there is limited information on the incidence and characteristics of IRIS in this setting. Furthermore, there is limited information on the incidence of IRIS in persons who initiate HAART with a CD4+ **cell** count <200 cells/mm^3^. Therefore, A5175 provides a unique opportunity to determine the incidence of IRS, identify predictors of this syndrome, and describe the outcome of these events in a prospectively designed study.

HBV/HIV-1 coinfection is common in many areas of the world and is an important cause of liver failure and hepatocellular carcinoma in resource-limited settings. Although both FTC and TDF are potent inhibitors of HBV replication, there are limited data on the effects of combination therapy with both FTC and TDF on HBV replication. A5175 provides an opportunity to assess the anti-HBV virus activity of the 3TC, FTC, and FTC//TDF in combination in **RLS**.

4.0 EVALUATIONS

Definitions of Opportunistic Infections

Given the limitations of ancillary laboratory, radiographic, and invasive tests, the diagnosis of OIs will be categorized as either confirmed or presumptive for each infection on the basis of the supporting clinical and laboratory data. Confirmatory testing is preferred, if available at each site. Clinical evaluations for OI diagnoses will occur at either scheduled monthly visits or unscheduled visits. ACTG diagnosis criteria (see the A5175 and A5185s MOPS) will be used for presumptive and confirmed OI diagnoses.

Definition of IRIS

- Evidence of an increase in CD4+ cell count by ≥50 cell/mm^3^ and/or a decrease in the HIV-1 viral load of >0.5 log and/or weight gain or other investigator defined signs of clinical improvement in response to starting ARV therapy

and

- Symptoms and/or signs that are consistent with an infectious/inflammatory condition and temporally related to initiation of **ART**

and

- Symptoms and/or signs that cannot be explained by a newly acquired infection, the expected clinical course of a previously recognized infectious agent, or the side effects of ARV therapy itself.

Retrospective Measurement of Hepatitis B Virus Load

The concentration of HBV DNA will be determined retrospectively from samples collected at Step 1 entry or at screening, weeks 24 and 48 and from samples collected as soon as possible after a new Grade ≥3 AST (SGOT) or ALT (SGPT). HBV DNA analysis will be performed retrospectively for all HBsAg-positive participants and also for new Grade ≥ 3 AST (SGOT) or ALT (SGPT), regardless of baseline HBsAg results. Since the estimated prevalence of chronic HBV infection across the 12 **non-U.S. CRS**s is roughly 10%, samples from approximately 125 participants will be included in this analysis.

All HBV virus loads will be performed retrospectively on stored specimens. Since assays to quantify HBV DNA are not available at all A5175 sites, it is expected that samples will be shipped in batch to a single site. The results of these tests will not be used for clinical management.

5.0 EXPLORATORY ENDPOINTS

- Descriptions of the occurrence and outcomes of OIs by country and treatment arm.
- The frequency of different IRIS in each geographic region (Caribbean, Indian subcontinent, Southeast Asia, South America, Southern Africa).
- The following variables will be evaluated as potential risk factors for IRIS: age, baseline CD4+ **cell** count, baseline plasma HIV-1 RNA, country, sex, and history of prior or concurrent TB.
- Time to Step 1 primary endpoint in participants who do or do not experience IRIS.
- Change of plasma HBV DNA from baseline to weeks 24 and 48 for HBsAg-positive participants in study arms 1A, 1B, and 1C.
- Proportion of participants experiencing an OI (including IRIS) while on ARV treatment.
- Time to occurrence of OI (IRIS) while on ARV treatment.
- Proportion of participants **experiencing coinfections** while on ARV treatment.
- Time to occurrence **of coinfection** while on ARV treatment.

5.1 Opportunistic Infections of Interest

- Candidiasis of bronchi, trachea, or lungs
- Candidiasis, esophageal
- Cervical cancer, invasive
- Coccidioidomycosis, disseminated or extrapulmonary
- Cryptococcosis, extrapulmonary
- Cryptosporidiosis, chronic intestinal (greater than 1 month's duration)
- Cytomegalovirus disease (other than liver, spleen, or nodes)
- Cytomegalovirus retinitis
- Herpes simplex: chronic ulcer(s) (greater than 1 month's duration); or bronchitis, pneumonitis, or esophagitis
- Histoplasmosis, disseminated or extrapulmonary
- Isosporiasis, chronic intestinal (greater than 1 month's duration)
- Mycobacterium avium complex or M. kansasii, disseminated or extrapulmonary
- Mycobacterium tuberculosis, any site (pulmonary or extrapulmonary)
- Mycobacterium, other species or unidentified species, disseminated or extrapulmonary
- Penicilliosis Marneffei, Disseminated
- Pneumocystis carinii pneumonia
- Pneumonia, recurrent
- Progressive multifocal leukoencephalopathy
- Salmonella septicemia, recurrent
- Toxoplasmosis of brain
- Wasting syndrome due to HIV

5.2 **Co-infections** of Interest

- CMV
- Hepatitis B
- Hepatitis C
- HSV
- VZV
- **KSHV/HHV-8**
- **Malaria**

6.0 REFERENCES

Benhamou Y, Bochet M, Tubiana R, Thibault V, Suffisseau L, Sullivan M et al. Tenofovir disoproxil fumarate suppresses lamivudine resistant HBV replication in patients co-infected with HIV/HBV. J Hepatol. 2002;36(Suppl 1):138 (abstract).

Carr A, Marriott D, Field A, Vasak E, Cooper DA. Treatment of HIV-1-associated microsporidiosis and cryptosporidiosis with combination antiretroviral therapy. Lancet. 1998;351(9098):256-61.

Cinque P, Pierotti C, Vigano MG, Bestetti A, Fausti C, Bertelli D, Lazzarin A The good and evil of HAART in HIV-progressive multifocal leukoencephalopathy. J Neurovirol 2001;7(4):358-63.

DeSimone JA, Pomerantz RJ, Babinchak TJ. Inflammatory reactions in HIV-1-infected persons after initiation of highly active antiretroviral therapy. Ann Intern Med. 2000;133(6):447-54.

Dworkin MS, Fratkin MD. Mycobacterium avium complex lymph node abscess after use of highly active antiretroviral therapy in a patient with AIDS. Arch Intern Med 1998;158(16):1828.

Fishman JE, Saraf-Lavi E, Narita M, Hollender ES, Ramsignhani R, Ashkin D. Pulmonary tuberculosis in AIDS patients: transient chest radiographic worsening after initiation of antiretroviral therapy. Am J Roentgenol 2000;174(1):43-9.

Fox PA, Barton SE, Francis N, Youle M, Henderson DC, Pillay D, et al. Chronic erosive herpes simplex virus infection of the penis, a possible immune reconstitution disease. HIV Med 1999 Oct;1(1):10-8.

Foudraine NA, Weverling GJ, van Gool, T et al. Improvement of chronic diarrhea in patients with advanced HIV-1 infection during potent antiretroviral therapy. AIDS 1998;12:35-41.

French MA, Lenzo N, John M, Mallal SA, McKinnon EJ, James IR, Price P, Flexman JP, Tay-Kearney ML. Immune restoration disease after the treatment of immunodeficient HIV-infected patients with highly active antiretroviral therapy. HIV Med 2000 Mar;1(2):107-15.

Giudici B, Vaz B, Bossolasco S, Casari S, Brambilla AM, Luke W, Lazzarin A, Weber T, Cinque P.Highly active antiretroviral therapy and progressive multifocal leukoencephalopathy: effects on cerebrospinal fluid markers of JC virus replication and immune response. Clin Infect Dis 2000 Jan;30(1):95-9.

Hassell M, French MA.Mycobacterium avium infection and immune restoration disease after highly active antiretroviral therapy in a patient with HIV and normal CD4+ counts. Eur J Clin Microbiol Infect Dis. 2001 Dec;20(12):889-91.

Lanzafame M, Trevenzoli M, Carreetta G, Lazzarini L, Vento S, Concia E. Mediastinal lymphadenitis due to cryptococcal infection in HIV-positive patients on highly active antiretroviral therapy. Chest. 1999 Sep;116(3):848-9.

Lee WM. Hepatitis B virus infection. N Engl J Med 1997;337:1733-45.

Miralles P, Berenguer J, Lacruz C, Cosin J, Lopez JC, Padilla B, Munoz L, Garcia-de-Viedma D. Inflammatory reactions in progressive multifocal leukoencephalopathy after highly active antiretroviral therapy. AIDS 2001 Sep 28;15(14):1900-2.

Murdaca G, Campelli A, Setti M, Indiveri F, Puppo F. Complete remission of AIDS/Kaposi's sarcoma after treatment with a combination of two nucleoside reverse transcriptase inhibitors and one non-nucleoside reverse transcriptase inhibitor. AIDS 2002;16(2):304-5.

Navas E, Martin-Davila P, Moreno L, Pintado V, Casado JL, Fortun J, Perez-Elias MJ, Gomez-Mampaso E, Moreno S. Paradoxical reactions of tuberculosis in patients with the acquired immunodeficiency syndrome who are treated with highly active antiretroviral therapy. Arch Intern Med 2002 Jan 14; 162(1): 97-9.

Phillips P, Kwiatkowski MB, Copland M, Craib K, Montaner J. Mycobacterial lymphadenitis associated with the initiation of combination antiretroviral therapy. J Acquir Immune Defic Syndr Hum Retrovirol. 1999 Feb 1;20(2):122-8.

Phillips P, Craib K, Sestak P, De Wet J, Voigt R, O'Shaughnessy M, Montaner JSG. Localized mycobacterial immune reconstitution syndrome (LMIRS): long-term outcome and discontinuation of mycobacterial therapy (MRx). 7th Conference on Retroviruses and Opportunistic Infections, January 30-February 2, 2000. Abstract 257.

Race EM, Adelson-Mitty J, Kriegel GR, Barlam TF, Reinmann KA, Letvin NL, Japour AJ. Focal mycobacterial lymphadenitis following initiation of protease-inhibitor therapy in patients with advanced HIV-1 disease. Lancet 1998 Jan 24;351(9098):252-5.

Risbud A, Mehendale S, Basu S, Kulkarni S, Walimbe A, Arankalle V, Gangakhedkar R, Divekar A, Bollinger R, Gadkari D, Paranjape R. Prevalence and incidence of hepatitis B virus infection in STD clinic attendees in Pune, India. Sex Transm Infect 2002;78: 169-173. Shelburne et al. Immune reconstitution inflammatory syndrome. Medicine 2002;81:213-27.

Sutcliffe S, Taha TE, Kumwenda NI, Taylor E, Liomba GN. HIV-1 prevalence and herpes simplex virus 2, hepatitis C virus, and hepatitis B virus infections among male workers at a sugar estate in Malawi. J Acquir Immune Defic Syndr 2002 Sep 1;31(1):90-7.

Wislez M, Bergot E, Antoine M, Parrot A, Carette MF, Mayaud C, Cadranel J. Acute respiratory failure following HAART introduction in patients treated for Pneumocystis carinii pneumonia. Am J Respir Crit Care Med 2001 Sep 1;164(5):847-851.

Worrell S, Deayton J, Hayes P, Emery VC, Gotch F, Gazzard B, Larsson-Sciard EL.Molecular correlates in AIDS patients following antiretroviral therapy: diversified T-cell receptor repertoires and in vivo control of cytomegalovirus replication. HIV Med 2001; 2(1):11-9.

#### APPENDIX III: MALNUTRITION, MICRONUTRIENT DEFICIENCY, IMMUNOLOGIC FUNCTIONING, AND METABOLIC STUDY PLAN

Primary Investigators: Ronald T. Mitsuyasu, M.D.

Robert C. Bollinger, M.D., M.P.H.

Javier R. Lama, M.D.

##### 1.0 ObjectiveS

1. To examine associations between nutritional deficiencies and response to ARV therapy.

2. To define baseline nutritional, metabolic, and body shape parameters and monitor changes in these parameters in response to ARV therapy.

2.0 BACKGROUND

Malnutrition has been associated with an increased risk of many infectious diseases in **RLS**. In particular, vitamin A and carotenoid deficiencies are immunodeficiency disorders from nutritionally poor diet and characterized by widespread pathologic alterations of mucosal epithelia, including the genitourinary tract, as well as impaired antibody and T-lymphocyte immune responses (Abdelgani, 1990; Beach, 1983; Bendich, 1989).

Vitamin A and carotenoid deficiencies have been associated with increased risk of, and higher mortality from, respiratory and diarrheal disease in children (Bendich, 1996; Evans, 1971; Faherty, 1990; Galan, 1990). Increased risk of schistosomiasis, malaria, tuberculosis, and leprosy has also been associated with vitamin A deficiency (John, 1997; Maciaszek, 1998; Mehendale, 2001; Pokhrel, 1994; Ross, 1998). Some carotenoids, such as β-carotene and β-cryptoxanthin are provitamin A carotenoids and may be converted to vitamin A in the intestinal tract. Effects of β-carotene and other carotenoids on immune function may be independent of their provitamin A activity (Semba, 1997). Carotenoids are also strong antioxidants, which may play a role in reducing oxidative stress. Low vitamin A and carotenoid levels have been associated with HIV-1 infection and AIDS (Semba, 1997; Semba, 1994; Sher, 1981) as well as an increased risk of vertical HIV-1 transmission in Malawi (Sommer, 1986). A study in Tanzania of patients attending STD clinics (Sturchler, 1987) indicated that low levels of β-carotene are associated with a high relative risk of subsequent HIV infection.

Retinol and its metabolites have been shown to down-regulate HIV-1 replication in monocytes, thought by some to be the primary target of HIV (Tang, 1997). A study from Kenya has also demonstrated an increased risk of HIV DNA shedding in the vaginal mucosa of HIV-1-infected women who have severe vitamin A deficiency (Usha, 1991). Increased viral replication in the genital mucosal monocyte-derived cells could result in increased genital shedding and increased risk of HIV-1 infection in genitally exposed individuals. Carotenoids also play a role in enhancing T- and B-lymphocyte function (West, 1995; West, 1997).

The metabolic and body shape changes that are occurring in patients infected with HIV may be related to the ARV drugs themselves, to the immune reconstitution that occurs with therapy, or because patients are surviving long enough for these changes to be recognized. Genetic predisposition to glucose intolerance, abnormalities in lipid metabolism, and/or cardiovascular disease varies in different populations and within populations by diet and nutritional status. In different populations around the world, the presentation of the metabolic and body shape abnormalities may differ because of differences in genetic predisposition, diet, baseline nutritional status, or body composition. As HIV is treated around the world, the ability to determine what, if any, additional medical risks for diabetes or cardiovascular disease may accompany ARV therapy will be of critical importance in planning long-term treatment strategies.

3.0 RATIONALE

Malnutrition and micronutrient deficiencies are prevalent in **RLS**. Immunologic dysfunction associated with vitamin A and carotenoid deficiency increases the risk for HIV-1 and a number of other infectious diseases. Therefore, it is important to assess whether malnutrition and micronutrient deficiency limits the benefits of ARV therapy in A5175 and to assess metabolic and body shape changes in response to ARV therapy in the resource-limited setting.

4.0 EVALUATIONS

Anthropometrical Measurements

Weight, height, conventional mid-waist, hip, mid-thigh, and mid-arm circumferences will be measured at study entry and per the Schedule of Events.

Laboratory Measurements

Albumin and ALT will be measured on-site in real-time as part of the scheduled evaluations. Total cholesterol, LDL cholesterol, HDL cholesterol, triglycerides, insulin, carotenoids, and vitamin A assays will be performed retrospectively from stored samples collected at Step 1 (entry) and every 6 months thereafter until the end of the study. The time and date that a study participant ate or drank something other than water prior to these visits will be recorded. If some or all of these assays are not available at all A5175 sites, samples will be shipped in batch to other laboratories for analysis. The results of these tests will not be used for clinical management.

5.0 Exploratory ANALYSES

The following variables will be evaluated as potential risk factors for reaching a Step 1 primary endpoint: baseline body mass index **(BMI)**, arm circumference, age, sex, total cholesterol, LDL cholesterol, HDL cholesterol, triglycerides, albumin, ALT, carotenoids, and vitamin A levels.

- Among participants who do not reach a Step 1 primary endpoint prior to week 96, the relationship between change in CD4+ **cell** count from baseline to weeks 24 and 96 with baseline **BMI**, arm circumference, age, total cholesterol, LDL cholesterol, HDL cholesterol, triglycerides, albumin, ALT, carotenoids, and vitamin A levels will be determined.
- Change in **BMI** in response to therapy will be documented. Change in body circumferences will be determined in response to changes in body weight. Changes from baseline total HDL and LDL cholesterol, triglycerides, and glucose and insulin (as HOMA Insulin Resistance [insulin in micro-u/mL x glucose(mmol/L)/ 22.5] will be tracked during the course of therapy.

6.0 REFERENCES

Abdelgani SM, Hussein L, Shaaban S. Vitamin A status in different stages of schistosomal cases and the effectiveness of oral vitamin A therapy. Z Ernahrungswiss 1990; 29: 249-255.

Beach RS, Laura PF. Nutrition and the acquired immunodeficiency syndrome. Annals of Internal Medicine 1983; 99: 565-566.

Bendich A. Carotenoids and the immune response. J Nutrition 1989; 119: 112.Bendich A. Antioxidant vitamins and human immune responses. Vitam Horm 1996; 52: 35-62.

Evans DI, Attock B. Folate deficiency in pulmonary tuberculosis: relationship to treatment and to serum vitamin A and beta-carotene. Tubercle 1971; 52: 288-294.

Faherty DA, Bendich A. Characterization of immunomodulatory activity of retinoids. Methods Enzymol 1990; 190: 252-259.

Galan P, Samba C, Luzeau R, Amedee-Manesme O. Vitamin A deficiency in pre-school age Congolese children during malarial attacks. Part 2: Impact of parasitic disease on vitamin A status. Int J Vitam Nutr Res 1990; 60: 224-228.

John GC, Nduati RW, Mbori-Ngacha D, et al. Genital shedding of human immunodeficiency virus type 1 DNA during pregnancy: association with immunosuppression, abnormal cervical or vaginal discharge, and severe vitamin A deficiency. J Infect Dis 1997; 175: 57-62.

Maciaszek JW, Coniglio SJ, Talmage DA, Viglianti GA. Retinoid-induced repression of human immunodeficiency virus type 1 core promoter activity inhibits virus replication. J Virol 1998; 72: 5862-5869.

Mehendale SM, Shepherd ME, Brookmeyer RS, Semba RD, Paranjape RS, Divekar AD, Gangakhedkar RR, Joshi S, Damble SS, Menon PA, Yadav R, Risbud AR, Gadkari DA, Bollinger RC. Low Carotenoid Concentration and the Risk of HIV Seroconversion in Pune, India. JAIDS 2001. 26:352-359.

Pokhrel RP, Khatry SK, West KP, Jr., Shrestha SR, Katz J, Pradhan EK, et al. Sustained reduction in child mortality with vitamin A in Nepal. Lancet 1994; 343: 1368-1369.

Ross DA. Vitamin A and public health: challenges for the next decade. Proc Nutr Soc 1998; 57: 159-165.

Semba RD. Vitamin A and human immunodeficiency virus infection. Proc Nutr Soc 1997; 56: 459-469.

Semba RD, Miotti PG, Chiphangwi JD, et al. Maternal vitamin A deficiency and mother-to-child transmission of HIV-1. Lancet 1994; 343: 1593-1597.

Sher R, Shulman G, Baily P, Politzer WM. Serum trace elements and vitamin A in leprosy subtypes. Am J Clin Nutr 1981; 34: 1918-1924.

Sommer A, Tarwotjo I, Djunaedi E, et al. Impact of vitamin A supplementation on childhood mortality. A randomized controlled community trial. Lancet 1986; 1: 1169-1173.

Sturchler D, Tanner M, Hanck A, et al. A longitudinal study on relations of retinol with parasitic infections and the immune response in children of Kikwawila village, Tanzania. Acta Trop 1987; 44: 213-227.

Tang AM, Graham NM, Semba RD, Saah AJ. Association between serum vitamin A and E levels and HIV-1 disease progression. AIDS 1997; 11: 613-620.

Usha N, Sankaranarayanan A, Walia BN, Ganguly NK. Assessment of preclinical vitamin A deficiency in children with persistent diarrhea. J Pediatr Gastroenterol Nutr 1991; 13: 168-175.

West KP, Jr., Katz J, Shrestha SR, et al. Mortality of infants <6 mo of age supplemented with vitamin A: a randomized, double-masked trial in Nepal. Am.J.Clin.Nutr. 1995; 62: 143-148.

West KP, LeClerq SC, Shrestha SR, et al. Effects of vitamin A on growth of vitamin A-deficient children: field studies in Nepal. J.Nutr. 1997; 127: 1957-1965.

#### APPENDIX IV: ADHERENCE AND BEHAVIORAL ISSUES STUDY PLAN

Primary Investigators: Steve Safren, Ph.D.

David Celentano, Sc.D. M.H.S.

##### 1.0 OBJECTIVES

1. To examine adherence to ARV treatment over time and to characterize its association with viral response, ARV treatment regimen, and other factors felt to be associated with adherence.

2. To examine the effects of ARV treatment on quality of life and explore the inter-relationship between quality of life, adherence, and HIV-1 viral load.

3. To examine the degree to which sexual risk-taking behavior changes longitudinally over the course of ARV treatment.

##### 2.0 BACKGROUND

Adherence is a difficult but critical part of ARV treatment. Nonadherence **to** combination therapy can result in increased HIV replication and the development of viral mutations, which can lead to medication resistance (Bangsberg et al. 2000; Carpenter et al. 1997, 2000; Hirsch et al. 1998; Wainberg & Friedland 1998). Several studies have established through self-report, pill count, or electronic pill-cap data that poor adherence is associated with poor medical outcome as measured by viral load or CD4+ cell count (Bangsberg et al. 2000; Catz et al. 2000; Gifford et al. 2000; Hecht et al. 1998; Montaner et al. 1998; Paterson et al. 2000; Stansell et al. 2001). In the present study, challenges to adherence include the complexity of the regimen, understanding of the regimen, potential side effects, the duration of the regimen, and the need to continue treatment despite symptomatic improvement. As a result, guidelines for adherence training have been developed to provide uniform methods for educating participants of the importance of ARV treatment adherence. Training will attempt to ensure that participants understand their own ARV treatment regimen and the importance of adherence to their health. Training will also include strategizing with the participants about barriers to and facilitators of adherence.

The success of any clinical trial of ARV treatment is dependent on the ability to adequately assess adherence. Poor adherence can lead to ARV resistance in both blood and genital tract. Sentinel surveillance in several countries suggests that there are an increasing number of new infections in individuals with multidrug resistance. Adherence can therefore be an important secondary factor in the transmission of resistant virus. Because nonadherence is known to be one of the factors associated with viral replication and development of resistance, it is crucial to have a reliable and valid assessment of adherence in order to determine moderators of study outcome and to determine the role of adherence, and potentially other factors, in the transmission of HIV. Proper assessment of ARV treatment adherence is also a necessary factor in adapting U.S.-based adherence interventions (Safren et al. 1999, 2001a) to other settings and cultures.

Assessment of HIV medication adherence is a difficult and complex undertaking. Currently there is no standardized "best" way to assess adherence, and therefore a multidimensional assessment is recommended (Chesney et al. 2000; Halkitis, 1998). For example, patients’ estimates of adherence to ARV treatment can be up to 30% higher than data from an electronic monitoring system of the pill bottles (MEMS or TRACK caps) that records when patients open their pill bottles (Arnsten 2000; Safren et al. 2001b).

Self-report through questionnaires or a structured interview provides an expedient way to determine the self-perceived level of adherence. However, many individuals forget whether they took pills while others may misrepresent their adherence to please the experimenter or medical provider. Pill diaries can assist participants with remembering to take their pills everyday and may constitute a good way to assess adherence through self-report. However, participants may forget to complete the diaries, may be motivated to please the clinician, and/or may complete the diaries the day before the study visit.

Electronic pill caps provide a way to validate externally the assessment of adherence. They have been validated as an assessment of adherence in several studies (Cramer et al. 1989; Rudd et al. 1990; Waterhouse et al. 1993) and function as an objective indicator of adherence. However, participants who refuse to take pills because of side effects or other reasons can still open the bottle at the prescribed times without taking pills, yielding an overestimate of adherence. Others may take out several doses at a time, possibly yielding an underestimate of adherence (Chesney et al. 2000).

Using punch cards to store medications also provides an easy way to determine whether medicines are taken; however, issues of privacy can inhibit individuals from carrying a large card with them, and they could punch out pills for the day and then forget to take them – yielding a false estimate of adherence.

In planning for the first ACTG-sponsored, large-scale, multisite, international trial of ARV treatment, several other behavioral factors warrant investigation. Measuring quality of life will help determine the degree to which individuals in **RLS** can return to their normal life-routine following successful initiation of ARV treatment. Measuring factors hypothesized to be associated with adherence and potential reasons for nonadherence (and therefore failure to achieve optimal benefit from treatment) will provide data for the successful planning of future trials. Assessing variables thought to be associated with adherence will provide useful data for adherence interventions.

Although ARV treatment slows disease progression, reduces serum viral load, and allows individuals with HIV to live longer, healthier lives, ARV treatment is not a cure. The potential success of ARV treatment in conjunction with ongoing new infections results in large numbers of individuals living with HIV who can pass the virus to others. Increased plasma HIV-1 RNA has been associated with increased sexual transmission of HIV in a drug-naïve population (Quinn, 2000). HPTN 052 is a study of ARV treatment to prevent HIV transmission in serodiscordant couples. The A5175 protocol is being developed in conjunction with and linked to HPTN 052 and will use many of the same study sites.

3.0 RATIONALE

An objective measure of adherence is necessary to validly assess adherence in the context of an international trial in **RLS**. Cultural factors are likely to play a significant role in the accuracy of self-reporting of adherence. For example, in Chennai, India, it is typical for patients to revere their physicians to the extent that many state that they are 100% adherent to their medications. External validation is necessary to determine whether this is the case. Additionally, due to the low availability of ARV treatment, and other demand characteristics of the provider-patient setting in these countries, participants may be motivated to present their adherence in the best possible light. Because nonadherence is one potential cause of viral nonresponse (but not the only cause), an external validator in addition to viral outcome and self-report is necessary.

Accurate measurement of adherence will help in the accurate interpretation of the major study endpoints: it will assist in determining whether failure to suppress viral load is due to the medications or caused by behavioral variables. Measurement of psychosocial variables related to adherence will provide the data that can aid in the planning of future trials as well as planning interventions to optimize adherence.

Measuring quality of life will assist in determining the degree to which symptomatic and disease-related biological improvements lead to improvements in participant functioning.

Finally, assessing the changes in sexual behavior in HPTN 052 and A5175 will provide insight into the degree to which sexual risk-taking behavior changes with successful treatment of HIV. In the absence of a cure for HIV, this strategy is consistent with current recommendations that link prevention with treatment for curtailing this virus.

4.0 EVALUATIONS

Adherence assessment procedures will be identical to those used in HPTN 052 and will involve both self-report and one objective indicator of adherence, pill counts. An HPTN 052 working group performed adherence preparedness activities that included qualitative research to determine the feasibility and acceptability of ways to assess adherence, including techniques such as pill counts, electronic pill caps, punch cards, and diaries.

Subjective Adherence Assessment

*Self-report*

Items from the ACTG adherence assessment instruments will be used for a self-report assessment of adherence. These measures ask individuals to recall the number of pills they took for varying time periods, including “yesterday,” “the day before yesterday,” “3 days ago,” and “the past 2 weeks.” In addition, it asks the frequency with which participants typically take their pills, using a Likert-type scale with anchor points ranging from “all of the time” to “sometimes” to “never.” A similar assessment will be based on the results of the adherence preparedness activities for HPTN 052. We intend to ask about discreet time periods as well as a “typical” week.

Objective Adherence Assessment

*Pill Counts*

While on Step 1 only, participants will be asked to bring all **ARV** pill containers to each study visit, whether the study provides the **ARV** drug or not. At each visit (including entry), the site pharmacist, nursing staff, clinician, or other trained clinical personnel (e.g., adherence counselors) will count the number of pills remaining of each medication to determine the number taken. Pill counts will be recorded on the CRF. Pill counts will not be conducted on Step 2.

Additional Assessments

*Quality of Life*

Quality of life will be assessed using the ACTG Health Survey Manual (ACTG Outcomes Committee, 1999). This measure focuses on health-related quality of life and is designed to be sensitive in detecting the degree to which medical treatment results in changes in an individual’s physical, social, and cognitive functioning, and subjective well-being (Health Survey Manual, p. 1). This measure is closely linked to the SF-21 (Bozette) which is the most widely used health-related quality-of-life measure but has specific application to HIV-infected individuals.

Variables Hypothesized to be Associated with Adherence

Perceived social support and the frequency of use of various substances will be assessed using modified standardized forms of the ACTG Outcomes Committee. Additionally, potential reasons for nonadherence (e.g., “forgetting,” “working,” “needed food”) that have been developed based on study site input will be assessed in a format similar to that used in the standard ACTG Outcomes Committee assessment forms.

A brief measure of sexual risk-taking behavior will be used. This self-report measure is a standard form used in HPTN studies and asks about frequency of anal and vaginal sex.

All interviewer-administered questionnaires used in this study will be translated and back-translated to help ensure that both staff and study participants understand them.

5.0 ANALYSES

Final analysis plans will be formulated closer to the time of analysis, but it is anticipated that unless independent research in this area suggests otherwise, analyses of adherence with be based on a dichotomous variable defining whether an adherence ratio calculated for each measurement occasion as the number of doses reported missed divided by the number of doses expected, over the 4-day recall period < or ≥95%. Summary scores for quality of life (Wu et al. 1998) will be calculated as previously described for the measurement tool being used.

Where appropriate, analyses will be stratified by country and by screening HIV-1 RNA

(< versus ≥100,000 copies/mL). If appropriate, Cox models may additionally be stratified by treatment regimen.

In addition to descriptive and exploratory analyses:

- Differences in adherence over time across the three treatment arms will be assessed using the methods of Wei-Johnson.
- Associations between adherence and HIV viral load will use Cox proportional hazards models to examine early and recent (using time-dependent adherence covariates) adherence as a predictor of time to virologic failure. Normal errors regression models with generalized estimating equations will be utilized to examine associations between adherence and changes in HIV viral load from baseline. These analyses will model adherence as a predictor of concurrent evaluations of HIV-1 RNA. All of these analyses will adjust for additional covariates as appropriate including but not limited to treatment regimen, study site, sex, age, and the incidence of viral resistance at baseline (from stored samples).
- Logistic and normal errors regression models with generalized estimating equations will be utilized as appropriate to examine trends in adherence, quality of life, and sexual risk-taking behavior over time and to examine their association with psychosocial and demographic predictors including, but not limited to, treatment regimen, study site, social support, sex, age, substance use, and other social and demographic variables examined in the self-report assessment battery. Further analyses will be explored to investigate the association of adherence with viral load, and quality of life.
- Cox proportional hazards models will be used to assess the association between adherence and the time to regimen failure, regimen discontinuation, and safety parameters.

6.0 REFERENCES

Arnsten J, Demas P, Gourevitch M, Buono D, Farzadegan H, Schoenbaum E. Adherence and viral load in HIV-infected drug users: comparison of self-report and medication event monitors (MEMS). Poster presented at the 7th Conference on Retroviruses and Opportunistic Infections. 2000, San Francisco, CA.

Bangsberg DR, Hecht FM, Charlebois ED, Zolopa AR, Holodniy M, Sheiner L, et al. Adherence to protease inhibitors, HIV-1 viral load, and development of drug resistance in an indigent population. AIDS 2000;14, 357-66.

Carpenter CC, Cooper DA, Fischl MA, Gatell JM, Gazzard BG, Hammer SM, et al. Antiretroviral therapy in adults: Updated recommendations from the International AIDS Society - USA Panel. JAMA 2000;283:381-90.

Carpenter, C J, Fischl MA, Hammer SM, Hirsch MS, Jacobsen DM, Katzenstein DA, et al. Antiretroviral therapy for HIV infection in 1997: Updated recommendations of the International AIDS Society - USA panel. JAMA 1997;277:1962-9.

Carey JW, Morgan M, Oxtoby MJ. Intercoder agreement in response to open-ended questions: Examples in tuberculosis research. Cultur Anthropol Meth 2000;8(3):1-5.

Catz SL, Kelly JA, Bogart LM, Benotsch EG, McAuliffe TL. Patterns, correlates, and barriers to medication adherence among persons prescribed new treatments for HIV disease. Health Psychol 2000;19:124-33.

Chesney MA, Ickovics JR, Chambers DB, Gifford AL, Neidig J, Zwickl B, et al. Patient Care Committee & Adherence Working Group of the Adult AIDS Clinical Trials Group. Self-reported adherence to antiretroviral medications among participants in HIV clinical trials: The ACTG adherence instruments. AIDS Care 2000;12:255-66.

Cramer JA, Mattson RH, Prevey ML, Scheyer RD, Ouellette VL. How often is medication taken as prescribed? A novel assessment technique. JAMA 1989;261:3273-7.

Gifford AL, Bormann JE, Shively MJ, Wright BC, Richman DD, Bozzette SA. Predictors of self-reported adherence and plasma HIV concentrations in patients on multidrug antiretroviral regimens. J Acquire Immune Defic Syndr 2000;23:386-95.

Halkitis, PN. The triangulation of measurement of antiretroviral adherence. Meeting of the American Psychological Association, 1998 Boston, MA.

Hecht FM, Colfax G, Swanson M, Chesney MA. Adherence and effectiveness of protease inhibitors in clinical practice. 5^th^ Conference on Retroviruses and Opportunistic Infections. 1998, Chicago, Ill.

Hirsch MS, Conway B, D'Aquila RT, Johnson VA, Brun-Vezinet F, Clotet B, et al. Antiretroviral drug resistance testing in adults with HIV infection: implications for clinical management. International AIDS Society--USA Panel. JAMA 1989;279:1984-91.

Montaner JS, Reiss P, Cooper D, Vella S, Harris M, Conway B, et al. A randomized, double-blind trial comparing combinations of nevirapine, didanosine, and zidovudine for HIV-infected patients. JAMA 1989;279: 930-7.

Paterson DL, Swindells S, Mohr J, Vergis EN, Squire C, Wagener MM, Singh N. Adherence to protease inhibitor therapy and outcomes in patients with HIV infection. Ann Intern Med 2000;133:21-30.

Quinn TC, Wawer MJ, Sewankambo N, Serwadda, D, Li, C, Wabwire-Mangen, F, Meehan, MO, Lutalo, T, Gray, RH. Viral load and heterosexual transmission of human immunodeficiency virus type 1. Rakai Project Study Group. N Engl J Med 2000; 342: 921-9.

Rudd P, Ahmed S, Zachary V, Barton C, Bonduelle D. Improved compliance measures: applications in an ambulatory hypertensive drug trial. Clin Pharmacol Ther 1990; 48: 676-85.

Safren SA, Otto MW, Worth J. Life-steps: applying cognitive-behavioral therapy to patient adherence to HIV medication treatment. Cog Behav Prac 1999;6:332-41.

Safren SA, DeSousa N, Hendriksen ES, Mayer K, Boswell S. Use of an on-line cue-controlled paging system to increase adherence to HIV antiretroviral medications. Paper presented at the annual meeting of the Association for the Advancement of Behavior Therapy, 2001a, Philadelphia, PA.

Safren SA, Otto MW, Worth J, Salomon E, Johnson W, Mayer K, Boswell S. Two strategies to increase adherence to HIV antiretroviral medication: Life-Steps and medication monitoring. Behav Res Ther 2001b; 39:1151-62.

Stansell J, Holtzer C, Mayer S, DeGuzman D, Hamel E, Lapins D. Factors affecting treatment outcomes in a medication event monitoring system. 8^th^ Conference on Retroviruses and Opportunistic Infections 2001, Chicago, Il.

Wainberg MA, Friedland G. Public health implications of antiretroviral therapy and HIV drug resistance. JAMA 1998;279:1977-83.

Waterhouse DM, Calzone KA, Mele C, Brenner DE. Adherence to oral tamoxifen: a comparison of patient self-report, pill counts, and microelectronic monitoring. J Clin Oncol 1993;11: 2457-8.

Wu AW, Hays RD, Kelly S, et al. Applications of the medical outcomes study health-rela quality of life measures in HIV/AIDS. Qual Life Res 1998;6, 531-54.

#### APPENDIX V: CLINICAL PHARMACOLOGY STUDY PLAN

Primary Investigators: David W. Haas, M.D.

Adriana Andrade, M.D., M.P.H.

Charles Flexner, M.D.

1.0 OBJECTIVES

A5175 offers the opportunity to explore the influence of concomitant medications, ethnicity, race, intercurrent disease, and nutritional status on **PK**. This study will also offer an opportunity to assess infrastructure needs for PK laboratory analysis in **RLS**. Important issues affecting drug interactions include (1) the availability of pharmacologically active agents over-the-counter (nonprescription) in some countries but by prescription only in other countries; (2) the variable prevalence of intercurrent infections (e.g., malaria and tuberculosis) that require drugs with potential interactions with study drugs; and (3) variable use of traditional, complementary, and alternative treatments. Fortunately, the NRTIs used in the initial regimens of A5175 are cleared primarily by the kidney and are therefore not prone to drug interactions. EFV, NVP, and ATV are metabolized by the liver and can induce or inhibit cytochrome P450 drug-metabolizing enzymes. Since EFV, NVP, ATV, and TDF have long elimination half-lives, a single sample from each participant will provide useful information for population PK analysis.

To avoid excessive phlebotomy and minimize protocol complexity, only sparse sampling will be done. Although trough plasma concentrations would be preferred, requiring that samples be drawn at trough would increase protocol complexity considerably, especially with EFV, which is taken at bedtime. Because of these limitations, all of the pharmacology components of the study will be exploratory.

Six exploratory questions will be addressed:

1. Are the population PK profiles of EFV, NVP, ATV, and TDF comparable to historic control data from previous studies?
2. Do some individuals have either extremely high or extremely low NVP, EFV, ATV, or TDF concentrations in comparison to other participants in this study?
3. Among individuals with either extremely high or extremely low NVP, EFV, ATV, or TDF concentrations, is there any apparent relationship with concomitant medications (to include prescription medications, nonprescription medications, and traditional, complementary, and alternative treatments)?
4. Is there any relationship between population PK profiles of the four study drugs and race or ethnicity?
5. Do concentrations of NVP, EFV, ATV, and TDF correlate with adherence measures obtained during this study?
6. Does rifabutin lower ATV trough concentrations when both agents are used concurrently?

2.0 PRIMARY AND SECONDARY DATA TO BE ACCESSED

*Evaluations*

Plasma Drug Levels

All participants participating in Step 1, Arms A, B, and C (receiving NVP, EFV, ATV or TDF) will have a single blood sample obtained for population PK analysis at week 4. (If for any reason it is not possible to obtain this sample at week 4, the sample may be obtained on any later date before week 8). The date and time of the previous dose of either of these drugs will be recorded by questioning the participant. The date and time of each blood sample draw will be recorded.

The following estimated number of plasma samples will be collected, based on 1520 participants, approximately 507 in each arm (population PK), and assuming equal use of NVP and EFV in Arms A and C, and ATV and TDF use in Arms C and B:

507 plasma samples for NVP*

507 plasma samples for EFV*

507 plasma samples for ATV

*A single blood sample of either NVP or EFV should be enough to perform PK analysis for TDF.

Concurrent Use of Rifabutin and ATV

Single trough blood samples will be collected whenever participants must use rifabutin and ATV concurrently. A single sample will be collected 14 days after initiation of rifabutin or ATV if ATV is initiated during the ongoing use of rifabutin (e.g., at study entry).

Concomitant Medications

All prescription and nonprescription medications being used by the participant should be recorded as completely as possible at study entry and any time a plasma sample is obtained for drug concentrations. Alternative and traditional medicines will be reported as Yes/No on the CRF. This documentation will allow later exploratory analysis of PK interactions.

3.0 STUDY DESIGN, MODELING, AND DATA ANALYSIS

With only an estimated 507 participants undergoing population PK analysis for each drug, all statistics comparing drug concentrations with historic controls will be exploratory, because these are non-paired comparisons and there is significant variability in the PK of these drugs across a population. The collected data will be examined to detect whether the estimated population mean, median, and standard deviations for PK parameters (C_max_, C_min_, and AUC) in this study are different from historical data. The A5175 data and historical data will be compared using a confidence interval of the difference between mean and median PK parameters. The plasma concentrations of the drugs will also be correlated to the measures of adherence to therapy.

Because of the exploratory nature of these analyses, and because numerous important factors cannot be predicted a priori, there will be no formal power calculations. Because assay data for population PK analyses should be available long before overall completion of A5175, analysis of some data for selected pharmacology objectives (e.g., comparing EFV, NVP, ATV, and TDF PK data to historical controls) may be done prior to overall protocol completion, if this is approved by A5175 protocol statisticians.

4.0 ANTICIPATED OUTCOMES

In a study of this nature, we cannot accurately anticipate outcomes. Monitoring plasma concentrations may identify drug-drug interactions that are potentially important for both safety and efficacy reasons, since NVP, EFV, ATV, and TDF have not been extensively studied in **RLS**. The exploratory examinations of the correlation of plasma concentrations with concomitant medications, race and ethnicity, and adherence measures will maximize information gathered from this study.

5.0 SAMPLE ANALYSIS, PROCESSING, AND SHIPMENT

NVP, EFV ATV, and TDF assays will be performed at a single ACTG pharmacology support laboratory or at a laboratory that has demonstrated assay proficiency in the ACTG Pharmacology Committee QA/QC program. The assay specifics and the shipment procedure are provided in the A5175 and A5185s MOPS.

#### APPENDIX VI: A5185s, EFFECT OF INITIAL ANTIRETROVIRAL TREATMENT ON GENITAL COMPARTMENT VIRUS IN INDIVIDUALS FROM RESOURCE-LIMITED SETTINGS

Substudy of A5175

Optimization of Antiretroviral

Therapy (OpART) Committee: **Richard Haubrich, M.D., Chair**

Protocol Co-Chairs Susan A. Fiscus, Ph.D.

Suniti Solomon, M.D.

Susan Cu-Uvin, M.D.

Statisticians: **Abel Tilahun, Ph.D.**

Laura Smeaton, M.S.

Data Managers: Ann Walawander, M.A.

Apsara Nair, M.S.

DAIDS Clinical Representative: Karin L. Klingman, M.D.

Clinical Trials Specialists: **Lara Hosey, M.A., C.C.R.P.**

**Laura E. Moran, M.P.H.**

A5185s

###### SCHEMA

EFFECT OF INITIAL ANTIRETROVIRAL TREATMENT ON GENITAL COMPARTMENT VIRUS IN INDIVIDUALS FROM RESOURCE-LIMITED SETTINGS

SUBSTUDY OF A5175

DESIGN: HIV-1 RNA will be measured in the genital secretions of men and women enrolled in A5175.

DURATION: Participants will participate in A5185s until they discontinue participation in A5175

SAMPLE SIZE: Approximately 315 participants

POPULATION: Participants in A5175 who agree to provide genital tract (GT) specimens at study entry (baseline), weeks 48 and 96, regimen switch, and at study discontinuation.

REGIMEN: Same as A5175.

1.0 STUDY OBJECTIVES

1.1 Primary Objectives

- - 1. To compare the baseline HIV-1 viral load in the genital secretions of participants infected with HIV subtype B with that of participants infected with subtype C.
    2. To compare baseline genital tract (GT) viral load between men and women.
  1. Secondary Objectives
     1. To measure the proportion of GT viral load that falls below the assay detection limit between baseline and week 48.
     2. To evaluate the durability of the ARV response in the GT by comparing GT viral loads at week 48 and at week 96.
     3. To compare resistance mutations in plasma (as identified by resistance testing performed in A5175) and those found in genital secretions at baseline and at study discontinuation.

2.0 INTRODUCTION

2.1 Background

Factors affecting GT viral load have been studied by several groups (reviewed in Fiscus et al. 1998; Mostad and Kreiss, 1996). Typically, approximately 30%-70% of ARV therapy-naïve individuals shed HIV-1 RNA in the GT (Vernazza et al. 1997; Hart, et al. 1999; Cu-Uvin et al. 2000). Higher GT viral loads have been demonstrated in men and women with higher plasma viral loads and with lower CD4+ cell counts (Vernazza et al. 1997; Hart et al. 1999). However, ARV treatment has been shown to reduce HIV viral load in the genital tracts of men (Gilliam et al. 1997; Gupta et al. 1997; Eron et al. 2000) and women (Si-Mohamed et al. 2000; Cu-Uvin et al. 2000). In fact, this is the rationale for HPTN 052, a protocol that will be conducted parallel to A5175 at many A5175 sites. In addition, since CD4+ cell counts will be lower in the participants enrolled in A5175 compared with those in HPTN 052, we will be better able to assess the role of ARV treatment on GT HIV-1 RNA levels; since more participants should be positive at baseline and thus be able to demonstrate a decrease in viral load.

No studies have been done to date on the effects of ARV treatment on genital secretion viral load in individuals infected with non-B subtypes, though there are some indications that both blood (Neilson et al. 1999; Dyer et al. 1998) and seminal plasma viral loads may be higher with subtype C infection compared with other subtypes (Dyer et al. 1998). Higher levels of HIV subtype C in genital secretions might help to explain the rapid spread of the epidemic in areas of the world where subtype C is most prevalent. Furthermore, concerns about the differences in treatment response by different subtypes make it even more important to study viral load in the GTs of treated participants. Most of the participants enrolled in A5185s will be infected with either HIV subtype C (about 50%) or subtype B (about 35%). A5185s will allow these important issues to be addressed.

Table 1. Predominant HIV-1 Subtypes Found in Countries with A5175 Enrollment Sites

| Country | Predominant Subtypes (approximate %) | Reference |
| --- | --- | --- |
| Brazil | B (80%), F (14%), C (3%) | Bongertz et al. 2000  Tanuri et al. 1999 |
| Haiti | B (most) | LANL HIV Database |
| India | C (80%-95%), B (2%-10%), A (2%) | Sahni et al. 2002  Gadkari et al. 1998 |
| Malawi | C (most) | Candotti et al. 2001 |
| Peru | B (98%) | Russell et al. 2000 |
| South Africa | C (most) | Bredell et al. 2002 Van Harmelen et al. 1999 |
| Thailand | CRF01_AE (80%-95%), B (5%-20%) | Nguyen et al. 2002  Subbarao et al. 1998 Ruxrungtham et al. 2001 |
| Zimbabwe | C (70%), B (12%), A (12%), D (7%) | Obi et al. 1997  Shafer et al. 1997 |
| United States | B (most) | Perrin et al. 2003 |

Differences in viral loads between men and women with similar CD4+ cell counts have been observed in individuals infected with subtype B virus (Farzadegan et al. 1998; Sterling et al. 1999; Napravnik et al. 2002). It is unknown whether similar differences are observed in the **GT**, because GT studies have typically been gender-specific; and few clinical trials, in which GT viral load has been measured, have enrolled both men and women. When this has occurred, women are usually under-represented (Gunthard et al. 2001). There have been some suggestions that women are less likely to durably suppress viral load in the GT compared with men (Gunthard et al. 2001). In addition,

several studies have shown that the GT is a separate compartment and that HIV may evolve differently in the GT, especially in response to therapy (Eron et al. 1998; Zhang et al. 1998; Si-Mohamed et al. 2000; Yamamura et al. 2001). These studies have demonstrated differences in resistance mutations between the GT and blood plasma.

2.2 Hypotheses

2.2.1 Baseline viral load in genital secretions of participants infected with HIV subtypes B and C will be similar.

- - 1. Baseline viral load in the GT of males and females will be similar.
    2. Viral load will decrease between baseline and week 48, and there will be no difference among the three treatment arms.
    3. Viral load will remain low, or undetectable, throughout the duration of the study.
    4. Resistance mutations to ARV treatment will be similar in blood and genital secretions.

2.3 Rationale

As a companion protocol to HPTN 052, A5175 will provide a unique opportunity to determine the effect and durability of ARV treatment on GT viral loads in both men and women, since participants in A5175 will have lower viral load levels at study entry than participants in HPTN 052. It is hoped that sufficient numbers of men and women infected with different HIV-1 subtypes will enroll, such that we will be able to evaluate differences that might be due to sex, subtype, or country. Determining the presence of resistance mutations to ARV treatment drugs and their potential transmission will be important for both studies.

3.0 STUDY DESIGN

Genital secretions from men and women will be collected at baseline (prior to initiation of A5175 study treatment), weeks 48 and 96, regimen switch at A5175 Step 2 **or Step 3** entry, and at study discontinuation. HIV-1 RNA will be measured in the genital secretions. If HIV-1 RNA is detected in the GT, the specimens will be tested for mutations associated with resistance to ARV drugs.

4.0 SELECTION AND ENROLLMENT OF PARTICIPANTS

4.1 Inclusion Criteria

- - 1. Enrollment in the main study A5175.

4.1.2 A signed A5185s informed consent.

4.1.3 Ability to provide a GT specimen prior to initiation of A5175 study treatment.

4.2 Exclusion Criteria

None.

4.3 Study Enrollment Procedures

- - 1. Prior to implementation of A5185s, sites must have the protocol and informed consent approved by their local institutional review board (IRB). Sites must be registered with and be approved by the DAIDS RCC Protocol Registration Office. Protocol registration must occur before any participants can be enrolled in A5185s.

Once a candidate for entry into A5185s has been identified, details will be carefully discussed with the participant. The participant will be asked to read and sign the consent form that was approved by both the **non-U.S. and U.S. CRS** IRBs and the DAIDS RCC Protocol Registration Office.

4.3.2 Sites will register participants to A5185s according to standard ACTG DMC procedures.

4.3.3 Coenrollment

Coenrollment into other studies will be addressed on an individual basis. For specific questions and approval regarding coenrollment, contact the A5175 CMC.

5.0 STUDY TREATMENT

All ARV treatment will be provided through A5175.

6.0 CLINICAL AND LABORATORY EVALUATIONS

The Schedule of Events (SOE) in section 6.1 includes all evaluations for A5185s.

The definitions for the SOE included in section 6.2 define the evaluations and provide timelines, while section 6.3 includes special considerations or instructions for evaluations.

- 1. Schedule of Events

| Evaluation | Entry^1^ | Week | | At Regimen Switch | Off Study/Final Visit |
| --- | --- | --- | --- | --- | --- |
|  |  | 48 | 96 |  |  |
| Genital Secretions Collection**^2^** | X | X | X | X | X |

^1^ A5175 Step 1 entry and **upon entry** to Step 2 **or 3**.

^2^ Genital secretions will be stored at **non-U.S. CRS**s until requested. **U.S. CRS**s will ship these specimens to the ACTG central repository according to their regular shipment schedule.

6.2 Timing of Evaluations

6.2.1 On-Study Evaluations

Evaluations will occur after registration into A5185s. Entry evaluations will occur prior to the initiation of A5175 study treatment at **entry to** Step 1 **and upon entry to Step 2 or 3**.

After week 8 on Step 1**,** 2, **or 3,** study visits will follow the SOE ± 14 days.

6.2.2 Off-Study Evaluations

These evaluations are required at the participant’s final study visit.

6.3 Special Instructions

6.3.1 Genital Secretions Collection and Processing

See the A5175 and A5185s MOPS for specimen collection and processing instructions.

Virologic Studies:

HIV-1 RNA will be measured in seminal plasma and cervical secretions using the Roche HIV-1 RNA assay.

Male Semen Collection

Semen must be collected at the **U.S. or non-US. CRS** or collected <2 hours prior to arrival at the study site. The participant should refrain from any kind of sexual activity for 48 hours prior to donation of semen.

Female Genital Secretions

Endocervical Canal Fluid Collection

The participant must refrain from any kind of sexual activity, douching, or inserting any intravaginal products for at least 48 hours prior to the collection of vaginal/cervical specimens. Although specimen collection during menses is acceptable, minimal blood contamination is preferred; therefore, sites should not schedule specimen collection during menses**,** if possible.

Women who have had a total hysterectomy may participate in the study. In such cases the specimen has to be taken from the vaginal vault instead of the cervix.

The collection of genital secretions in pregnant female participants is allowed. However, collection should not be conducted during labor or with signs of vaginal bleeding or rupture of membranes. Collections should resume 6 weeks after delivery.

7.0 TOXICITY MANAGEMENT

Same as per A5175

8.0 CRITERIA FOR TREATMENT DISCONTINUATION

Same as per A5175

9.0 STATISTICAL CONSIDERATIONS

9.1 General Design Issues

None

9.2 Endpoints

- - 1. Primary endpoints

9.2.1.1 Baseline viral load in the GT.

- - 1. Secondary endpoints
       1. Viral load in GT below the assay detection limit at week 48.
       2. Viral load in GT below the assay detection limit at weeks 48 and 96.
       3. Genotype of HIV-1 in GT.

9.3 Sample Size and Accrual

A5185s is powered for the primary comparison described in objective 1.1.1: a comparison of baseline GT viral load between participants infected with subtype B virus with participants infected with subtype C virus. We also calculate the power for the comparison described in objective 1.1.2: comparison of baseline GT viral load in men and women. We assume that 50% of participants will be infected with subtype C,and 33% with subtype B. With 301 participants, we have 80% power to detect a difference of 0.50 in baseline GT viral load between groups, defined by subtypes using a t-test. This number of participants provides greater than 99% power to detect a difference of 0.50 between men and women. The standard deviation of the GT viral load in men is assumed to be 1.45 (based on presented data, Coombs et al. 1998). For women, the standard deviation is assumed to be 1.31 (based on data from ACTG 866, which used the relationship that the standard deviation = [3rd quartile – median]/0.674). As the actual test used for these comparisons will be a nonparametric Wilcoxon rank sum test, the sample size is increased to 315 to adjust for the slight loss of power resulting from using a nonparametric test.

9.4 Analyses

A Wilcoxon rank-sum test will be used to compare the viral load in the GT between participants with subtypes B and C virus and between men and women.

We will determine the proportion of participants who had detectable viral load in the GT at baseline, but undetectable viral load at week 48, and develop binomial confidence intervals for this proportion. We will also calculate these proportions and confidence intervals at other time points listed in section 9.2.2.

To compare the proportion below detection at week 48 to this proportion at week 96, we will create 2 x 2 tables, which will provide the proportion above at both detection time points, the proportion above/below at both time points, the proportion above detection at week 48 but not at week 96, and vice versa. Statistical tests will be based on the McNemar test.

Descriptive and exploratory methods will be used to compare the genotypes in plasma and in the GT. For each codon, we will form histograms showing the proportions of sequences that are mutant in both plasma and GT, wild type in both, mutant in plasma but not GT, and vice versa. Statistical comparisons will be adjusted for multiple testing using the False Discovery Rate of Benjamin and Hochberg. Clustering of sequences will be performed separately for plasma and GT sequences.

10.0 DATA COLLECTION AND MONITORING AND ADVERSE EXPERIENCE REPORTING

Same as per A5175

11.0 HUMAN STUDY PARTICIPANTS

Same as per A5175

12.0 PUBLICATION OF RESEARCH FINDINGS

Same as per A5175

13.0 BIOHAZARD CONTAINMENT

Same as per A5175

14.0 REFERENCES

Bongertz V, Bou-Habib DC, Brigido LF, Caseiro M, Chequer PJ, Couto-Fernandez JC, Ferreira PC, Galvao-Castro B, Greco D, Guimaraes ML, Linhares de Carvalho MI, Morgado MG, Oliveira CA, Osmanov S, Ramos CA, Rossini M, Sabino E, Tanuri A, Ueda M. HIV-1 diversity in Brazil: genetic, biologic, and immunologic characterization of HIV-1 strains in three potential HIV vaccine evaluation sites. Brazilian Network for HIV Isolation and Characterization. J Acquir Immune Defic Syndr 2000;23:184-93.

Bredell H, Hunt G, Casteling A, Cilliers T, Rademeyer C, Coetzer M, Miller S, Johnson D, Tiemessen Ct, Martin Dj, Williamson C, Morris L: HIV-1 subtype A, D, G, Ag and unclassified sequences identified in South Africa. AIDS Res Hum Retroviruses 2002, 18:681-3.

Candotti D, Mundy C, Kadewele G, Nkhoma W, Bates I, Allain JP: Serological and molecular screening for viruses in blood donors from Ntcheu, Malawi: high prevalence of HIV-1 subtype C and of markers of hepatitis B and C viruses. J Med Virol 2001, 65:1-5.

Coombs R, Speck C, Hughes J, et al. Association between culturable human immunodeficiency virus type 1 (HIV-1) in semen and HIV-1 RNA levels in semen and blood: evidence for compartmentalization of HIV-1 between semen and blood. J Infec Dis 1998; 177:320-30).

Cu-Uvin S, Caliendo AM, Reinert S, et al. Effect of highly active antiretroviral therapy on cervicovaginal HIV-1 RNA. AIDS 2000;14:415-21.

Dyer JR, Kazembe P, Vernazza PL, et al. High levels of HIV-1 in blood and semen of seropositive men in sub-Saharan Africa. J. Infect Dis 1998;177: 1742-46.

Eron JJ, Vernazza, PL, Johnston D.M, Seillier-Moiseiwitsch F, Alcorn TM, Fiscus SA, Cohen MS. Resistance of HIV-1 to antiretroviral agents in blood and seminal plasma: implications for transmission. AIDS 1998; 612: F181-189.

Eron J.J., Smeaton, L.M, Fiscus, S.A., Gulick, R.M., Currier, J.S., Lennox, J.L., D’Aquila, R.T., Rogers, M.D., Tung, R., and Murphy, R.L. HIV-1 levels in semen: the effects of protease inhibitor therapy on HIV-1 levels in semen. J Infect Dis 2000; 181:1622-1628.

Farzadegan H, Hoover DR, Astemborski J, Lyles CM, Margolick JB, Markham RB, Quinn TC. Sex differences in HIV-1 viral load and progression to AIDS. Lancet 1998;352:1510-1514.

Fiscus SA, Vernazza PL, Gilliam B, Dyer J, Eron JJ, Cohen, M.S. Factors associated with changes in HIV shedding in semen. AIDS Res and Human Retrovirus 1998; 14: S-27-31.

Gadkari DA, Moore D, Sheppard HW, Kulkarni SS, Mehendale SM, and Bollinger RC. Transmission of genetically diverse strains of HIV-1 in Pune, India. Indian J Med Res 1998; 107:1-9.

Gilliam BL., Dyer JR, Fiscus SA, Marcus C, Cohen MS, Eron JJ Effects of reverse transcriptase inhibitor therapy an HIV-1 viral burden in semen. JAIDS 1997;15:54-60.

Gunthard HF, Havlir DV, Fiscus S, et al. Residual human immunodeficiency virus-1 RNA and DNA in lymph node and HIV RNA in genital secretions and in cerebral spinal fluid after two years of suppression of viremia in the Merck 035 cohort. J Infect Dis 2001;183:1318-27.

Gupta P, Mellors J, Kingsley L, et al. High viral load in semen of human immunodeficiency virus type 1-infected men at all stages of disease and its reduction by therapy with protease and nonnucleoside reverse transcriptase inhibitors. J Virol 1997;71:6271-5.

Hart CE, Lennox JL, Pratt-Palmore M, et al. Correlation of human immunodeficiency virus type 1 RNA levels in blood and the female genital tract. J Infect Dis 1999; 871-82.

Los Alamos National Laboratory (LANL) HIV Database, <http://www.hiv.lanl.gov/content/hiv-db/mainpage.html>.

Mostad SB, Kreiss JK. Shedding of HIV-1 in the genital tract. AIDS 1996;10:1305-15.

Napravnik S, Poole C, Thomas JC, Eron JJ. Gender difference in HIV RNA levels: a meta-analysis of published studies. JAIDS 2002;31:11-9.

Neilson JR, John GC, Carr JK, Lewis P, Kreiss JK, Jackson S, Nduati RW, Mbori-Ngacha D, Panteleeff DD, Bodrug S, Giachetti C, Bott MA, Richardson BA, Bwayo J, Ndinya-Achola J, Overbaugh J. Subtypes of human immunodeficiency virus type 1 and disease stage among women in Nairobi, Kenya. J Virol. 1999 May;73(5):4393-403.

Nguyen L, Hu DJ, Choopanya K, Vanichseni S, Kitayaporn D, Van Griensven F, Mock PA, Kittikraisak W, Young NL, Mastro TD, and Subbarao S. Genetic Analysis of Incident HIV-1 Strains Among Injection Drug Users in Bangkok: Evidence for Multiple Transmission Clusters During a Period of High Incidence. J Acquir Immune Defic Syndr 2002; 30:248-56.

Obi CL, McAdoo HP, Onigbinde AO, Murray M, Tswana SA, and Moyo SR. Subtypes of HIV-1 and the impact of dual infections of HIV-1 and measles virus on micronutrient levels of pregnant women in Harare, Zimbabwe. Cent Afr J Med 1997; 43:165-72.

Perrin, L, Kaiser L, Yerly, S. Travel and the spread of HIV-1 genetic variants. Lancet 2003 3:22-27.

Russell Kl, Carcamo C, Watts Dm, Sanchez J, Gotuzzo E, Euler A, Blanco Jc, Galeano A, Alava A, Mullins Ji, Holmes Kk, Carr Jk: Emerging Genetic Diversity of HIV-1 in South America. AIDS 2000; 14:1785-1791.

Ruxrungtham K, Phanuphak P. Update on HIV/AIDS in Thailand. J Med Assoc Thai 2001; 84 Suppl 1:S1-17.

Sahni AK, Prasad VV, Seth P. Genomic diversity of human immunodeficiency virus type-1 in India. Int J S AIDS 2002; 13:115-8.

Shafer RW, Eisen JA, Merigan TC, and Katzenstein DA. Sequence and drug susceptibility of subtype C reverse transcriptase from human immunodeficiency virus type 1 seroconverters in Zimbabwe. J Virol 1997; 71:5441-5448.

Si-Mohamed A, Kazatchkine M, Heard I, et al. Selection of drug-resistant variants in the female GTof human immunodeficiency virus type 1-infected women receiving antiretroviral therapy. J Infect Dis 2000;182:112-22.

Sterling TR, Lyles CM, Vlahov D, Astemborski J, Margolick JB, Quinn TC. Sex differences in longitudinal human immunodeficiency virus type 1 RNA levels among seroconverters. J Infect Dis 1999; 180: 666-672.

Subbarao S, Limpakarnjanarat K, Mastro TD, Bhumisawasdi J, Warachit P, Jayavasu C, Young NL, Luo CC, Shaffer N, Kalish ML, and Schochetman G. HIV type 1 in Thailand, 1994-1995: persistence of two subtypes with low genetic diversity. AIDS Res Hum Retroviruses 1998; 14:319-327.

Tanuri A, Swanson P, Devare S, Berro OJ, Savedra A, Costa LJ, Telles JG, Brindeiro R, Schable C, Pieniazek D, and Rayfield M. HIV-1 subtypes among blood donors from Rio de Janeiro, Brazil. J Acquir Immune Defic Syndr Hum Retrovirol 1999; 20:60-6.

Van Harmelen Jh, Van Der Ryst E, Loubser As, York D, Madurai S, Lyons S, Wood R, Williamson C: A predominantly HIV type 1 subtype C-restricted epidemic in South African urban populations. AIDS Res Hum Retroviruses 1999; 15:395-8.

Vernazza, PL, Gilliam BL, Dyer J, Fiscus SA, Eron JJ, Frank AC, Cohen MS Quantitation of HIV in semen: correlation with antiviral treatment and immune status. AIDS 1997;11:989-93.

Yamamura Y, Tirado G, Soto A, Sepulveda G, Zorilla C. Resident HIV of vaginal compartment are distinct from plasma HIV in both drug resistance mutations and viral evolution. 8^th^ CROI, February 2001, Chicago, IL Abstract 719.

Zhang H, Dornadula G, Beumont M, Livornese L Jr, Van Uitert B, Henning K, Pomerantz RJ. Human immunodeficiency virus type 1 in the semen of men receiving highly active antiretroviral therapy. N Engl J Med 1998;339:1803-9.

APPENDIX VII

DIVISION OF AIDS

AIDS CLINICAL TRIALS GROUP (ACTG)

SAMPLE INFORMED CONSENT

A5175

Title: A Phase IV, Prospective, Randomized, Open-Label Evaluation of the Efficacy of Once-Daily Protease Inhibitor and Once-Daily Non-Nucleoside Reverse Transcriptase Inhibitor-Containing Combinations for Initial Treatment of HIV-1-Infected Individuals from Resource-Limited Settings (PEARLS)

SHORT TITLE FOR THE STUDY:

Evaluation of Once-Daily PI and NNRTI Regimens as Initial HIV Therapy in Individuals from Resource-Limited Settings

INTRODUCTION

You are being asked to take part in this research study because you are infected with the HIV virus. This study is sponsored by the **U.S.** National Institutes of Health (NIH**)**. The doctor in charge of this study at this site is (insert name of Principal Investigator). Before you decide if you want to be a part of this study we want you to know about the study.

This consent form gives you information about this study. The research staff will talk with you about this information. Ask questions and discuss any concerns you may have with the research staff. If you agree to take part in this study, you will be asked to sign this consent form. You may keep a copy of this consent.

Please note that:

- Your participation in this study is entirely voluntary.
- You may stop taking part in the study at any time without losing your standard health care.

WHY IS THIS STUDY BEING DONE?

This study will look at how well different combinations of anti-HIV drugs (ART) work to decrease the amount of HIV in the blood (viral load) of people living in different areas of the world. It will also look at whether taking the same amount of ART once a day works about as well as taking ART twice a day to decrease viral load. The study will also see how well you tolerate the drug combinations and how safe they are.

OVERVIEW OF THE STUDY

There are about 20 anti-HIV drugs from different drug groups that can be combined in varying three-drug regimens. These drug combinations allow people infected with HIV to live longer and to feel healthier. These drugs however do not cure HIV/AIDS. All of the drugs used in this study are approved by the U**.S.** Food and Drug Administration (FDA) for treating HIV/AIDS.

Nine different combinations of anti-HIV drugs will be used in this study. The drugs are: lamivudine (3TC), zidovudine (ZDV), didanosine enteric-coated (ddI-EC), emtricitabine (FTC), stavudine (d4T), tenofovir disoproxil fumarate (TDF), efavirenz (EFV), nevirapine (NVP) and atazanavir (ATV). The study will provide all of these drugs while you are on the study. When you begin the study you will only take three of the nine drugs.

You will be randomized (assigned by chance, like flipping a coin) to one of three treatment groups. This means that you will have an equal chance of being in one of the three groups listed below. You will not be able to choose which group you are in, but both you and your doctor will know which group you are in.

Group 1: 3TC/ZDV (one pill given twice a day) + EFV (one pill given once a day)

Group 2: FTC (one pill given once a day) + ATV (two pills given once a day) + ddI-EC (one pill given once a day)

Group 3: FTC/TDF (one pill given once a day) + EFV (one pill given once a day)

A drug may be switched if you have a bad reaction to the drug or if you cannot take the drug for some other medical reason either when you begin the study or during the study.

If you are infected with both the hepatitis B virus (HBV) and HIV, you should know that three of the anti-HIV study drugs (3TC, FTC, TDF) are also used to treat HBV.  Recent U.S. Department of Health and Human Services treatment guidelines recommend using two drugs together to treat HBV. If you are assigned to Group 1 or 2, you will only receive one drug to treat HBV, either 3TC or FTC.  If you are infected with HBV, you should talk to your doctor about whether you should participate in this study and if you should take other medications for HBV that are not provided by this study.

WHAT DO I HAVE TO DO IF I PARTICIPATE IN THIS STUDY?

If you decide to enroll in this study, you will need to come to the clinic for physical examinations, interviews, and laboratory tests frequently - at least 12 times during the first year and at least every 8 weeks after that. Most visits will require 1½ to 2 hours but some visits might require 2½ hours. Any time that results of exams and laboratory tests such as viral load, CD4+ cell counts (immune cells that help fight infection such as HIV), and safety tests are known, they will be given to you. There may be times when you must come for additional visits if these exams or tests are abnormal. Some of the blood drawn throughout the study will be stored. We will use some of this blood for study-related tests that will be done later or to check the study results later.

Screening Visit

If, after discussing the study in detail with the research staff, you decide to enter the study you will sign the informed consent and undergo a series of lab tests and medical evaluations to see if you can participate in the study.

- You will have a physical examination.
- You will be asked questions about your medical history and any medicines that you have taken, including prescription and nonprescription medicines, alternative therapies, dietary supplements, and herbal treatments.
- If you are a woman and able to become pregnant, you will either have about 1 teaspoon (5 mL) of blood drawn or be asked to provide some urine for a pregnancy test. If you are pregnant you may not enter this study.
- You will also have about 2 tablespoons (30 mL) of blood drawn for routine blood tests, CD4+ cell counts, viral load, and hepatitis B. You will be told the results of your hepatitis B test.
- You will be asked how to be contacted in case you miss a visit or there are ever problems with your lab test results.
- You may need to have an EKG (a way to measure electrical signals in the heart). This procedure may take about 1 hour. You will be told if any problems are found.

Entry Visit

If the screening visit showed that you can be in the study, you will return to clinic no sooner than 7 days after the screening visit. You will be asked questions about any medicines or any medical problems you have had since the screening visit. You will be given a physical exam, and have about 6 to 7 tablespoons (90-105 mL) of blood drawn for routine tests and viral load. You will have some body measurements taken (arm, waist, thigh, and hip). You will be asked questions about how you are feeling and other lifestyle issues including sexual behavior and substance use (this will take about 10 minutes). One of the clinic staff and/or your doctor will discuss ways to help you take your study medicines as prescribed and ways to lower sexual risk behavior. If you are a woman and able to become pregnant, you will be asked to either provide some urine or give permission to use some of your blood for a pregnancy test.

After you complete all entry tests, you will be assigned by chance as described above to one of three treatment groups. You will be given your study medicines, instructed on how to take them, and what to do if you have any side effects or problems with them. You should begin your study medicines within 3 days after **you are assigned to a treatment group.**

Scheduled Follow-up Clinic Visits

During the study, you will come to the clinic frequently for scheduled visits. You will be asked to bring your study medicine bottles with you to all visits. The research staff will count the number of pills in each bottle and give you enough study medicines to last until your next study visit. At each study visit, you will be asked about any medicines you have had since the last visit, **have** a physical exam, and have between 1 and 7 tablespoons (15-105 mL) of blood drawn for routine tests, CD4+ cell counts, and viral load. At some visits you will have some body measurements taken. At some study visits you will be asked questions about how you are feeling and lifestyle issues. At other visits you will also be asked questions about taking your study medicines. At some visits the clinic staff or your doctor may discuss ways to take your study medicines as prescribed and ways to lower your sexual risk behavior. If you are a woman and able to become pregnant, you will be asked to either provide some urine or give permission to use some of your blood for a pregnancy test.

Other Study Visits

You may need to come into the clinic for reasons other than the regularly scheduled evaluations. At these visits you will have most of the same procedures that were done at the routine study visit.

These visits may be for the following reasons:

- For increases in viral load. If your viral load increases or is not lowered by the study drugs, you will come to the clinic for an additional visit. About 4 to 5 tablespoons (60-75 mL) of blood will be drawn. You will be asked to complete the questionnaire about taking your study medicines and your pills will be counted.
- For switching to a new ART combination. If your viral load increases and stays up or your CD4+ cell **counts** decrease by a large amount or you have an AIDS condition, you will be offered an opportunity to switch to a different ART regimen. You may also need to switch to a new regimen if the regimen you are taking causes unwanted or toxic side effects.
- **For participants randomized to Group 2 (FTC + ATV + ddI-EC). An independent group called a Data and Safety Monitoring Board (DSMB), which monitors studies in progress to ensure the safety and well-being of study participants, reviewed A5175 on May 6, 2008. This group found that one of the study regimens (FTC + ATV + ddI-EC) was not as good as the 3TC/ZDV + EFV study regimen in treating HIV in participants who had not been treated for their HIV in the past. All participants who were receiving FTC + ATV + ddI-EC were asked to:**

1. **Discuss their treatment options with their site study team and their doctors or primary care providers.**
2. **Return to the study clinic with the current study drugs and change to another study-provided ARV regimen.**

**If you were receiving FTC + ATV + ddI-EC and do not want to take another study-provided regimen, you will be asked to continue to participate in the study and receive laboratory monitoring through the study; however, the regimen of FTC + ATV + ddI-EC must be obtained from outside the study.**

- For permanent discontinuation of study treatment. If you stop your study drugs before the end of the study, you will come to the clinic. You will also be asked to continue to participate in the study and to return for regularly scheduled visits and procedures.

Other

If you agree, about 2 extra teaspoons (10 mL) of blood will be taken at some study visits. This blood will be stored with protection of your identity and may be used for future ACTG-approved HIV related research.

________ YES ________ NO

HOW MANY PEOPLE WILL TAKE PART IN THIS STUDY?

**1571 people have taken part in** this study. Other countries taking part in the study include Haiti, Peru, Brazil, Malawi, Zimbabwe, South Africa, India, Thailand, and the United States. [*delete your country from this list]*

HOW LONG WILL I BE IN THIS STUDY?

**Originally, the team estimated that you would be in this study for 2.5 to 3 years. In order to gather enough information to answer the primary research question of the study, it has lasted longer than anticipated. However, on November 3, 2009, a DSMB reviewed the study and decided that continuation of the study beyond May 2010 would not greatly improve the ability of the study to answer the primary research question regarding the comparison of 2 of the study groups: those randomized to receive 3TC/ZDV + EFV and those randomized to receive FTC/TDF + EFV. At the recommendation of the DSMB, the study will close to follow-up on May 31, 2010.**

Once the study is over, the study will no longer provide you with anti-HIV drugs. At that time, you may have to stop a drug combination that has worked well for you, either because you cannot afford the treatment or because those drugs are not available in your country. Efforts will be made by your doctor to find a way to continue ART drugs after the study is over. Continued treatment cannot be guaranteed.

WHY WOULD THE DOCTOR TAKE ME OFF STUDY MEDICINES OR OFF STUDY EARLY?

The study doctor may need to take you off the study early without your permission if:

- you are at risk of not following the study requirements and might cause harm to yourself or others;
- the study is canceled by the United States National Institutes of Health, the AIDS Clinical Trials Group (ACTG), **the Office of Human Research Protections (OHRP),** your country’s national health agency, the drug companies supporting this study, your site’s Institutional Review Board (IRB) (An IRB is a committee that watches over the safety and rights of research participants.); or
- a Data Safety Monitoring Board (DSMB) recommends that the study be stopped early (**a** DSMB is a group of experts **who are not involved with the study, but monitor the study).**

The study doctor may also need to take you off the study drugs without your permission if:

- continuing the study drugs may be harmful to you;
- you need a treatment that you may not take while on the study;
- you are not able to take the study drugs as required by the study; or
- you are unable, for any reason, to attend the study visits as required by the study.

IF I HAVE TO PERMANENTLY STOP TAKING STUDY MEDICINES (3TC, ZDV, DDI-EC, FTC, D4T, TDF, EFV, NVP, AND ATV) OR ONCE I LEAVE THE STUDY, HOW WOULD THESE MEDICINES BE PROVIDED?

*During the study:*

If you must permanently stop taking study-provided drugs before your study participation is over, the study staff will discuss other options that may be of benefit to you.

*After the study:*

After you have completed your study participation, the study will not be able to continue to provide you with the drugs that you received on the study. If continuing to take these or similar drugs would be of benefit to you, the study staff will discuss how you may be able to obtain them.

WHAT ARE THE RISKS OF THE STUDY?

The anti-HIV drugs used in this study may have side effects, some of which are listed below. Please note that these lists do not include all the side effects seen with these drugs. These lists include the more serious or common side effects with a known or possible relationship. If you have questions concerning additional study drug side effects, please ask the medical staff at your site.

**Immune Reconstitution Syndrome: In some people with advanced HIV infection, signs and symptoms of inflammation from other infections may occur soon after anti-HIV treatment is started.**

The use of potent **ARV** drugs may be associated with an abnormal placement of body fat and wasting. Some of the body changes include:

- Increase in fat around the waist and stomach area
- Increase in fat on the back of the neck
- Thinning of the face, legs, and arms
- Breast enlargement

##### Nucleoside and Nucleotide Analogues

Lactic acidosis (elevated lactic acid levels in the blood) and severe hepatomegaly (enlarged liver) with steatosis (fatty liver) that may result in liver failure, other complications or death have been reported with the use of **ARV** nucleoside analogues alone or in combination. The liver complications and death have been seen more often in women on these drug regimens. Some nonspecific symptoms that might indicate lactic acidosis include: unexplained weight loss, stomach discomfort, nausea, vomiting, fatigue, cramps, muscle pain, weakness, dizziness and shortness of breath.

*Zidovudine (Retrovir®, ZDV)*

- Decrease in the number of white blood cells that help fight infection
- Decrease in the number of red blood cells, which may cause weakness, dizziness, and fatigue
- Muscle aches
- Weakness and wasting
- Headache
- Upset stomach
- Vomiting
- Decrease in appetite
- Vague overall feeling of discomfort
- Lack of energy
- Feeling tired
- Sleeplessness
- Heartburn

*Risks of Lamivudine (Epivir®, 3TC)*

- **Headache**
- **Feeling tired**
- **Dizziness**
- **Numbness tingling, and pain in the hands or feet**
- **Depression**
- **Trouble sleeping**
- **Rash**
- **Upset stomach, vomiting, nausea, loose or watery stools**
- **Pancreatitis (inflammation of the pancreas), which may cause death. If you develop pancreatitis, you may have one or more of the following: stomach pain, nausea, and vomiting.**
- **Abnormal pancreatic and liver function blood tests**

**If you are infected with both hepatitis B and HIV, you should be aware that your liver function tests may increase, and symptoms associated with hepatitis (an acute inflammation of the liver) may worsen if lamivudine is stopped. Although most of these cases have resolved without treatment, some deaths have been reported.**

*Risks of Lamivudine/Zidovudine (Combivir®, 3TC/ZDV)*

No new or unexpected side effects are observed with the 3TC 150mg/ZDV 300mg combination tablet than those observed when each drug is given separately.

***Risks of*** *Tenofovir Disoproxil Fumarate (Tenofovir DF, Viread®, TDF)*

- Upset stomach, vomiting, gas, loose or watery stools
- **Generalized weakness**
- Dizziness
- **Depression**
- **Headache**
- Abdominal pain
- **Worsening or new k**idney damage or failure
- Inflammation or swelling and possible damage to the pancreas **and liver**
- Shortness of breath
- Rash
- Allergic reaction**: symptoms** may include fever, rash, upset stomach, vomiting, loose or watery stools, abdominal pain, achiness, shortness of **breath, a** general feeling of illness**, or a potentially serious swelling of the face, lips, and/or tongue.**
- **B**one **pain or bone changes such as bone thinning and softening which may increase the risk of breakage**
- **Muscle pain and muscle weakness**

NOTE: If you are infected with both Hepatitis B and HIV, you should be aware that your liver function tests may increase, and symptoms associated with hepatitis (an acute inflammation of the liver) may worsen if tenofovir is stopped.

NOTE: Because there is only a small amount of information on tenofovir in pregnant women, tenofovir should be used during pregnancy only if clearly needed.

*Risks of Emtricitabine (Emtriva™, FTC)*

- Headache
- Dizziness
- Tiredness
- Inability to sleep, unusual dreams
- Loose or watery stools
- Upset stomach (nausea) or vomiting
- Abdominal pain
- Rash, itching, which sometimes can be a sign of an allergic reaction
- Skin darkening of the palms and/or soles
- Increase cough
- Runny nose
- Abnormal liver function tests, which could mean liver damage
- Increases in pancreatic enzyme (substances in the blood), which could mean a problem with the pancreas
- Increased triglycerides
- Increased creatine phosphokinase (CPK), which could mean muscle damage

**NOTE: If you are infected with both hepatitis B and HIV, you should be aware that your liver function tests may be abnormal, and symptoms associated with hepatitis (an acute inflammation of the liver) may worsen if emtricitabine is stopped.**

*Emtricitabine, FTC/Tenofovir Disoproxil Fumarate, TDF (Truvada™)*

No new or unexpected side effects are observed with the FTC 200 mg/TDF 300 mg combination tablet than those observed when each drug is given separately.

*Risks of Didanosine (Videx®, ddI-EC)*

- Pancreatitis (inflammation of the pancreas), which may cause death. If you develop pancreatitis, you may have one or more of the following: stomach pain, nausea, and vomiting.
- Deaths from liver failure have been reported in pregnant women receiving the combination of didanosine and stavudine with other anti-HIV drugs.
- Numbness, tingling, and pain in the hands or feet
- Abnormal vision changes
- Upset stomach, vomiting, and loose or watery stools
- Headache
- Abnormal pancreatic function blood tests or abnormal liver function blood tests
- Increase in uric acid in the bloodstream
- When didanosine is used with other medicines with similar side effects, these side effects may be seen more often and may be more severe than when didanosine is used alone.
- People who take didanosine together with stavudine, with or without hydroxyurea, may be at greater risk for pancreatitis or liver problems or both. These conditions may result in death.

*Risks of Stavudine (Zerit®, d4T)*

- Deaths from liver failure have been reported in pregnant women receiving the combination of stavudine and didanosine with other anti-HIV drugs.
- People who take stavudine together with didanosine, with or without hydroxyurea, may be at greater risk for pancreatitis or liver problems or both. These conditions may result in death.
- Numbness, tingling, and pain in your hands or feet
- Pancreatitis (inflammation of the pancreas), which may cause death. If you develop pancreatitis, you may have one or more of the following: stomach pain, nausea, and vomiting.
- Rash
- Upset stomach, vomiting and loose or watery stools
- Abdominal pain
- Abnormal liver function blood tests or abnormal pancreatic function blood tests
- Rare cases of muscle weakness, which may progress to paralysis and inability to breathe; this may be associated with elevation of lactic acid in the blood.

**When stavudine is used with other medicines with similar side effects, these side effects may be seen more often, and may be more severe than when stavudine is used alone.**

Non-Nucleoside Reverse Transcriptase Inhibitors

*Risks of Efavirenz (Sustiva®, EFV)*

A small number of people may experience the following serious psychiatric problems:

- Depression, which may be severe
- Suicidal thoughts or attempts (rarely)
- Aggressive behavior
- Psychosis-like symptoms, such as abnormal thinking, paranoia, and delusions

People with a history of psychiatric problems may be at greater risk for these serious psychiatric problems.

Side effects associated with the central nervous system may include the following:

- Dizziness
- Trouble sleeping
- Abnormal dreams
- Drowsiness
- Confusion
- Difficulty concentrating
- Hallucinations
- A feeling of strangeness and losing touch with reality
- An exaggerated feeling of well-being
- Agitation or anxiety

If alcohol or mind- or mood-altering drugs are used with efavirenz, it is possible that the central nervous system side effects could become worse.

Additional side effects include:

- Rash
- Upset stomach
- Loose or watery stools
- Headache
- Pancreatitis (inflammation of the pancreas), with one or more of the following: stomach pain, nausea, or vomiting
- Hepatitis (inflammation of the liver)
- Abnormal increases in pancreatic and liver enzyme levels in the blood.
- Abnormal increases in the amount of triglycerides and cholesterol in the blood
- Abnormal vision
- Fever

Efavirenz and Pregnancy:

The use of this drug during pregnancy and especially early pregnancy should be avoided. Efavirenz may cause fetal harm when taken during the first three months of pregnancy. Serious birth defects, including those of the central nervous system, have been seen in the offspring of animals and women on Efavirenz.

A false-positive urine-screening test for marijuana has been seen with one particular test brand and has not been seen when using other screening tests or with tests used to confirm results for marijuana.

*Risks of Nevirapine (Viramune®, NVP)*

Severe liver damage that can result in death may occur and is often associated with a rash. Being female or having a higher CD4+ cell count, regardless of gender, increases the risk of developing liver damage.

Women with CD4+ cell counts greater than 250, including pregnant women receiving chronic nevirapine therapy, are at greatest risk for developing liver damage. Men with CD4+ cell counts greater than 400 are also at increased risk. However, these reactions can happen at any CD4+ **cell** count in both men and women. People who have abnormal liver function tests before starting nevirapine and people with active hepatitis B or C infection are also at higher risk for liver damage.

If you are developing liver damage, you may have one or more of the following:

- Tiredness
- General feeling of illness or flu-like feeling
- Loss of appetite
- Nausea
- Pale stools
- Dark urine
- Yellowing of the skin or whites of your eyes
- Liver tenderness or abnormal liver function tests

Hypersensitivity reactions (“allergic reaction”) may occur. These reactions are rarely fatal. The symptoms that you may notice are: rash, fever, tiredness, muscle or joint aches, flu-like feeling, blisters, mouth sores, facial swelling, red eyes and irritation of the eyes, general feeling of discomfort, and/or liver damage described above, kidney problems, and/or changes in white blood cell levels.

**Muscle breakdown causing muscle aches or pain has been observed in some people experiencing skin and/or liver reactions associated with nevirapine.**

Rash is the most common side effect associated with nevirapine. Rash occurs more often in women. Most rashes occur early during treatment. The rash may be severe and rarely may cause death. One of the risk factors for developing serious skin reactions includes failure to take nevirapine properly during the first 14 days of treatment.

The risk of people developing any of the serious side effects listed above is greatest during the first few months of treatment, but these side effects also can occur later. If you develop any of the side effects listed above, no matter how long you have been receiving nevirapine, you must contact your health care provider right away and before you take your next dose. Your health care provider will instruct you on what to do next. If you and your doctor then decide to stop your treatment because of liver damage, hypersensitivity, or severe skin reactions, you should never take nevirapine again.

Other than the serious side effects listed above, additional side effects include:

- Fever
- Headache
- Upset stomach (nausea, vomiting)

Protease Inhibitors (PI)

**The use of protease inhibitors may be associated with the following:**

- **Increases in the amount of triglycerides and/or cholesterol in the blood (this has not been observed with atazanavir)**
- **Development of diabetes or the worsening of high blood sugar**

There have been reports of increased bleeding in HIV-infected persons with hemophilia who were treated with protease inhibitors. It is not known if protease inhibitors were the cause of these bleeding episodes.

Risks of Atazanavir (Reyataz®, ATV)

- Increased bilirubin, which may be associated with yellowing of the skin or eyes
- **Rash, which may be severe and rarely may cause death**
- **A change in the way your heart beats (heart rhythm change). Symptoms you may experience if this occurs include dizziness or lightheadedness.**
- Upset stomach, vomiting, and diarrhea
- Abdominal pain
- Increases in liver function tests
- **Muscle pain**
- **Cough**
- Fever
- **Kidney stones**
- **Gallbladder disorders such as gallstones and gallbladder inflammation**

Other Risk Information

After you have begun taking the treatment, do not stop taking any of the drugs unless you have discussed it with your doctor. Suddenly stopping your treatment can cause a sudden increase in the amount of HIV in your blood, and the HIV can become resistant to the treatment.

There is a risk of serious and/or life-threatening side effects when nonstudy medications are taken with study drugs. For your safety, you must tell the study doctor or nurse about all medications you are taking before you start the study and also before you take any treatment. You must also tell the study doctor or nurse before taking any nonstudy therapies while you are on the study. Deaths have occurred when HIV-infected people have taken therapies such as herbs with anti-HIV drugs. In addition, you must tell the study doctor or nurse before enrolling in any other clinical trials while on the study.

Risks of Blood Draws

Pain, bleeding, or bruising where the needle enters the skin; lightheadedness; and in rare cases, fainting or infection.

ARE THERE STUDY DRUG RISKS RELATED TO PREGNANCY?

It is not known if some of the drug combinations in this study would harm unborn babies. Tests in pregnant animals do show some risks for some drugs. If you are having sex that could lead to pregnancy, you must agree not to become pregnant.

You must use one method of birth control that you discuss with the study staff. You may choose from any of the following birth control methods:

1. Birth control drugs that prevent pregnancy given by pills, shots or placed on or under the skin
2. Male or female condoms with or without a cream or gel that kills sperm
3. Diaphragm or cervical cap with a cream or gel that kills sperm
4. Intrauterine device (IUD)

However, if you take a drug combination that includes EFV, you must use two methods of birth control that you discuss with the study staff. One method must be a barrier method. You must continue to use both methods of birth control until 12 weeks after stopping EFV. If you are a woman and are unable to use two methods, your doctor will talk with you about taking NVP or another ART drug rather than EFV.

ARE THERE SPECIAL RISKS RELATED TO BREAST-FEEDING?

A mother who is infected with HIV may infect her baby through breast milk. It is recommended that HIV-infected mothers who are able to obtain baby formula and clean water not breast-feed their babies. It is unknown whether the study medicines pass through the breast milk and may cause harm to your infant. It is also unknown whether study drugs may reduce the chances that HIV can pass to your baby through your breast milk.

WHAT HAPPENS IF I BECOME PREGNANT?

If you think you may be pregnant at any time during the study, tell your study staff right away. The study staff will talk to you about your choices and refer you to a provider of prenatal care if you do not have one. If you decide to continue in A5175, you will be asked to sign another consent form. The A5175 study will not provide or pay for care related to your pregnancy or the delivery of your baby.

ARE THERE BENEFITS TO TAKING PART IN THIS STUDY?

If you participate in this study, there may be a direct benefit to you, but no guarantee can be made. Anti-HIV drug combinations do allow people infected with HIV to live longer and to feel healthier. Also, your health will be followed more closely than usual while you are on the study, which may help you to feel better. Laboratory tests to monitor the effects of these drugs will be provided by the study. It is also possible that you may receive no benefit from being in this study.

Information learned from this study may help others who have HIV/AIDS. It may also help improve the ability of people to get ART, by identifying drug combinations that cost less and are as effective as more expensive drug combinations.

WHAT OTHER CHOICES DO I HAVE BESIDES THIS STUDY?

*[Insert general information about HIV/AIDS treatment availability in your country or locale.]*

Antiretroviral medications, laboratory tests to monitor the effectiveness of these medications, and quality medical care for HIV/AIDS may or may not be available to you outside the study. The clinic staff will discuss with you other treatment choices in your area and the risks and the benefits of all the choices.

WHAT ABOUT CONFIDENTIALITY?

The study team will provide you with an identification number. The identification number (not your name or other information that could be used to identify you) will be used for laboratory tests or blood work stored for testing in future studies. Your medical records and the list of names, addresses, and identification numbers will be kept in a locked room. Only the study staff will have the keys. Any publication of this study will not use your name or identify you personally.

Efforts will be made to keep your personal information confidential. We cannot guarantee absolute confidentiality. Your personal information may be disclosed if required by law. Your records may also be reviewed by the (insert name of site IRB), ethics committee, National Institutes of Health (NIH), your country’s national health agency, study staff, study monitors, and drug companies supporting this study.

***[****Delete the following for non-U.S. sites****]****.* In addition to the efforts of the study staff to help keep your personal information private, we have gotten a Certificate of Confidentiality from the U.S. Federal Government. This certificate means that researchers cannot be forced to tell people who are not connected with this study, such as the court system, about your participation. Also, any publication of this study will not use your name or identify you personally. Having a Certificate of Confidentiality does not prevent you from releasing information about yourself and your participation in the study. Even with the Certificate of Confidentiality, if the study staff learns of possible child abuse and/or neglect or a risk of harm to yourself or others, we will be required to tell the proper authorities.

WILL I RECEIVE ANY PAYMENT?

[*Insert site-specific information on compensation to study participants*].

WHAT ARE THE COSTS TO ME?

There will be no cost to you for study-related visits, physical examinations, laboratory tests or other procedures. You, your insurance company, or your health care system may need to assume the cost of ART drugs not provided by the study. (delete reference[s] to insurance company or health care system if not applicable at site). In some cases, it is possible that your insurance company or health care system will not pay for these costs because you are participating in a research study.

WHAT HAPPENS IF I AM INJURED?

If you are injured as a result of being in this study, you will be given immediate treatment for your injuries, and you will be referred for further treatment, if necessary. However, you may/may not (as per site/country policy) have to pay for this care. There is no program for compensation either through [this institution] or the U.S. National Institutes of Health (NIH). You will not be giving up any of your legal rights by signing this consent form.

WHAT ARE MY RIGHTS AS A VOLUNTEER IN A RESEARCH STUDY?

Taking part in this study is completely voluntary. You may choose not to take part in this study or leave this study at any time. You will be treated the same no matter what you decide.

We will tell you about new information from this or other studies that may affect your health, welfare, or willingness to stay in this study. If you want the results of the study, let the study staff know.

WHAT DO I DO IF I HAVE QUESTIONS OR PROBLEMS?

For questions about this study or a research-related injury, contact:

- (name of the investigator or other study staff)
- (telephone number of above)

For questions about your rights as a volunteer in a research study contact:

- (name or title of person on the Institutional Review Board (IRB) or other organization appropriate for the site)
- (telephone number of above)

SIGNATURE PAGE

If you have read this consent form (or had it explained to you), all your questions have been answered and you agree to take part in this study, please sign your name below.

Participant’s Name (print) Participant’s Signature and Date

Participant’s Legal Guardian (print) Legal Guardian’s Signature and Date

(As appropriate)

Study Staff Conducting Study Staff Signature and Date

Consent Discussion (print)

Witness’s Name (print) Witness’s Signature and Date

(As appropriate)

APPENDIX VIII

DIVISION OF AIDS

AIDS CLINICAL TRIALS GROUP (ACTG)

SAMPLE INFORMED CONSENT

For

WOMEN WHO BECOME PREGNANT ON A5175

Title: A Phase IV, Prospective, Randomized, Open-Label Evaluation of the Efficacy of Once-Daily Protease Inhibitor and Once-Daily Non-Nucleoside Reverse Transcriptase Inhibitor-Containing Combinations for Initial Treatment of HIV-1 Infected Individuals from Resource-Limited Settings (PEARLS) Trial

SHORT TITLE FOR THE STUDY:

Evaluation of Once Daily PI and NNRTI Regimens as Initial HIV Therapy in Individuals from Resource-Limited Settings

INTRODUCTION

Because you are now pregnant, you are being asked if you want to continue taking part in this research study. This study was designed so that women who were pregnant could not join the study. However, because you were already in the study when you became pregnant, you will be allowed to stay in the study and come for the same study visits whether or not you continue study medicines during your pregnancy

This is a consent form. It gives you more information about how continuing on this study may affect your pregnancy and your baby. The research staff will talk with you about this information. You may also talk with your own doctor about what is best for you and your baby, if you should remain on study-provided antiretroviral **(ARV)** drugs, or choose other AR**Vs**. If you agree to stay in this study, you will be asked to sign this consent form. You will get a copy to keep. You are free to ask questions of the research staff at any time.

WHAT DO I HAVE TO DO IF I STAY IN THIS STUDY?

It is not known if some of the drug combinations in this study harm unborn babies. Tests in pregnant animals do show some risk for some drugs. If you become pregnant while taking efavirenz (EFV), your doctor will tell you to stop taking it and replace it with another anti-HIV medicine. This study will not provide care related to your pregnancy, the delivery of your baby, or the care of your baby. You must arrange for you and your baby’s care outside of this study. Long-term medical follow-up is recommended for a baby whose mother takes ART during pregnancy. The research staff will talk with you about long-term follow-up and the possibility of enrolling your baby in a long-term follow-up study.

WHAT ARE THE RISKS RELATED TO STAYING IN THE STUDY?

Now that you are pregnant, there are some possible risks you should know. These possible risks to you and your baby are in addition to the risks that are described in the A5175 study consent you already signed.

Risks to You if You Stay on **ARV drugs**:

1. Different side effects or more severe side effects may occur in pregnant women taking anti-HIV medicines. This may make it more difficult for the medicines to work on the HIV in your blood.
2. The amount of **ARV drugs** in **your** blood may change during pregnancy. This means that the amounts of **ARV drugs** in your blood may decrease and not work as well or cause the HIV to become resistant to the drugs.
3. It is not known if some risks of pregnancy might be made worse by study medicines and may result in death.

Risks to Your Baby if You Stay on **ARV drugs**:

1. It is not known if some study medicines may cause you to have a baby that is born early or dead.

2. It is not known if some study medicines may cause your baby to be sick or have birth defects. Not all birth defects are seen at birth. Some birth defects are seen later as the baby grows.

3. In the U.S., only zidovudine (ZDV, Retrovir) is approved by the FDA to decrease the risk of passing HIV from mother to baby. The U.S. Public Health Service recommends that women discuss with their doctor the use of ZDV alone and with other anti-HIV drugs to decrease the risk of passing HIV to their baby.

There is little safety information regarding the use of TDF, FTC**,** and ATV in pregnancy.

BREAST-FEEDING

After delivery, if you decide to breast-feed your baby you may continue taking study medicines. Researchers know that HIV can pass through breast milk, and taking study medicines has not been proven to decrease the chance of passing HIV through your breast milk to your baby.

ARE THERE BENEFITS TO STAYING IN THIS STUDY?

Use of combination anti-HIV drugs during later pregnancy significantly decreases the chance that the baby will become HIV-infected during pregnancy. However, if you continue to take part in this study, no guarantee can be made of a benefit to you or your baby. It is possible that you and your baby will receive no benefit from continuing in this study. Information learned from this study may help others who have HIV.

WHAT OTHER CHOICES DO I HAVE BESIDES STAYING ON STUDY DRUGS?

Instead of staying on the study medicines you have the choice of:

- treatment with prescription medicines available to you
- treatment with experimental drugs being studied for use during pregnancy, if you qualify
- no treatment

Please talk to your doctor about these and other choices available to you. Your doctor will explain the risks and benefits of these choices.

WHAT ABOUT CONFIDENTIALITY?

Efforts will be made to keep your personal information confidential. We cannot guarantee absolute confidentiality. Your personal information may be disclosed if required by law. Any publication of this study will not use your name or identify you personally.

People who may review your records include: (insert name of site) IRB, National Institutes of Health (NIH), study staff, study monitors, drug companies supporting this study, and their designees.

***[****Delete following for non-U.S. sites****]****.* In addition to the efforts of the study staff to help keep your personal information private, we have gotten a Certificate of Confidentiality from the U.S. Federal Government. This certificate means that researchers cannot be forced to tell people who are not connected with this study, such as the court system, about your participation. Also, any publication of this study will not use your name or identify you personally. Having a Certificate of Confidentiality does not prevent you from releasing information about yourself and your participation in the study. Having a Certificate of Confidentiality does not prevent you from releasing information about yourself and your participation in the study. Even with the Certificate of Confidentiality, if the study staff learns of possible child abuse and/or neglect or a risk of harm to yourself or others, we will be required to tell the proper authorities.

WHAT ARE THE COSTS TO ME?

In addition to any costs that are described in the study consent you already signed, this study will not cover any cost related to your pregnancy, delivery of your baby, or care of your baby.

WILL I RECEIVE ANY PAYMENT?

**[*Insert site-specific information on compensation to study participants*].**

WHAT HAPPENS IF MY BABY OR I AM INJURED?

If your baby or you are injured as a result of being in this study, you will both be given immediate treatment for your injuries. However, you may or may not have to pay for this care, **depending on the policy at your site or in your country.** There is no program for compensation either through this institution or the **U.S.** National Institutes of Health (NIH). You will not be giving up any of your legal rights by signing this consent form.

WHAT ARE MY RIGHTS AS A RESEARCH PARTICIPANT?

Continuing to take part in this study is completely voluntary. You may choose not to continue in this study or leave this study at any time. You will be treated the same no matter what you decide.

We will tell you about new information from this or other studies that may affect your health, welfare or willingness to stay in this study. If you want the results of the study, let the study staff know.

WHAT DO I DO IF I HAVE QUESTIONS OR PROBLEMS?

For questions about this study or a research-related injury, contact:

- (site insert name of the investigator or other study staff)
- (site insert telephone number of above)

For questions about your rights as a research participant, contact:

- (site insert name or title of person on the Institutional Review Board (IRB) or other organization appropriate for the site)
- (site insert telephone number of above)

SIGNATURE PAGE

If you have read this consent form (or had it explained to you), all your questions have been answered and you agree to take part in this study, please sign your name below.

Participant’s Name (print) Participant’s Signature and Date

Participant’s Legal Guardian (print) Legal Guardian’s Signature and Date

(As appropriate)

Study Staff Conducting Study Staff Signature and Date

Consent Discussion (print)

Witness’s Name (print) Witness’s Signature and Date

(As appropriate)

APPENDIX IX

DIVISION OF AIDS

AIDS CLINICAL TRIALS GROUUP (ACTG)

SAMPLE INFORMED CONSENT

A5185s

TITLE: A5185s: EFFECT OF INITIAL ANTIRETROVIRAL TREATMENT ON GENITAL COMPARTMENT VIRUS IN INDIVIDUALS FROM RESOURCE-LIMITED SETTINGS: A SUBSTUDY OF A5175

SHORT TITLE FOR THE STUDY:

A5185s: Genital Secretions Substudy of A5175.

WHY IS THIS SUBSTUDY BEING DONE?

This study is being done to see what happens to the type and amount of HIV in genital secretions when you take antiretroviral **(ARV)** drugs.

PROCEDURES

You may decide not to join this substudy or join it and leave before it is completed and still stay in the main A5175 study. **371 people have taken** part in this substudy. **Originally, the team estimated that you would be in this study for 2.5 to 3 years, the same time as A5175. In order to gather enough information to answer the primary research question of A5175, this study has lasted longer than anticipated. However, on November 3, 2009, a DSMB reviewed the A5175 study and decided that continuation of the A5175 study beyond May 2010 would not greatly improve the ability of the study to answer the primary research question regarding the comparison of 2 of the study groups: those randomized to receive 3TC/ZDV + EFV and those randomized to receive FTC/TDF + EFV. At the recommendation of the DSMB, A5175 and A5185s will close to follow-up on May 31, 2010.**

You can schedule study visits on the same day as the main A5175 study visits.

Eligibility

If you qualify and decide to take part in this study, you will be asked to sign this consent form.

Study Entry and Evaluations

You will be asked to give genital secretions on the day you begin the study, once every year, if you change your ART regimen, and at the end of the study (about 5 times). Results of the genital secretion tests will not be available until the end of the study.

Genital Secretions Collection:

**For Men Only**

**You may not have any sexual activity for 48 hours before to giving your semen specimen. You will need to cleanse your penis and then masturbate (without lubricants other than water) in a private room at the clinic and put your semen in a sterile container given to you. If you can bring your sample to the clinic within 2 hours, you may do this at home.**

**For Women Only**

**You may not have any sexual activity, or douche, or use any vaginal products in or around your vagina area for at least 48 hours before specimens are obtained.**

You will have a pelvic exam (the same exam used to do a Pap smear) performed to obtain specimens. A speculum (an instrument that stretches the opening of the vagina) will be eased into the vagina. Tiny absorbent strips will be inserted into the entrance of the cervix (the cervix is the opening of the womb or uterus) and left in place for about 1 minute to absorb the sample.

Leaving the Substudy Early

If you decide to leave this substudy before it ends, you will be asked to return to the clinic and give a genital specimen.

RISKS AND/OR DISCOMFORTS

Risks of Genital Secretions Collection

The risks of collection of genital secretions (male or female) are pain and discomfort.

PREGNANCY AND BREAST-FEEDING

If you become pregnant during this study, you may continue to take part in this substudy and have genital secretions collected during your pregnancy. You will not have specimens collected during labor or if you have signs of vaginal bleeding or rupture of membranes. You may have genital secretions collected again 6 weeks after delivery. Women who are breast-feeding may take part in this substudy.

BENEFITS

There will be no direct benefit to you from being in this study. However, knowledge gained from this study may help others who have HIV/AIDS in the future.

WILL I RECEIVE ANY PAYMENT?

**[*Insert site-specific information on compensation to study participants*].**

SIGNATURE PAGE

If you have read this consent form (or had it explained to you), all your questions have been answered and you agree to take part in this study, please sign your name below.

All other information in the main A5175 study consent that you signed, applies to this substudy consent as well.

Participant’s Name (print) Participant’s Signature and Date

Participant’s Legal Guardian (print) Legal Guardian’s Signature and Date

(As appropriate)

Study Staff Conducting Study Staff Signature and Date

Consent Discussion (print)

Witness’s Name (print) Witness’s Signature and Date

(As appropriate)
