# Supplementary material for: Efficacy and Safety of Three Antiretroviral Regimens for Initial Treatment of HIV-1: A Randomized Clinical Trial in Diverse Multinational Settings
Source: PLoS Med. 2012 Aug 14;9(8):e1001290. doi: 10.1371/journal.pmed.1001290 (PMC3419182; doi:10.1371/journal.pmed.1001290)
Supplement: Text S3 — Appendix 60. Diagnoses appendix. (PDF) [file pmed.1001290.s019.pdf]

# APPENDIX 60

## Version 1.2

## January 2009

FSTRF maintains this code set on behalf of several clinical trials networks for which it is the data center.
Permission must be obtained through user.support@fstrf.org to use or copy it for any other purpose.

# TABLE OF CONTENTS


I. PARASITIC INFECTIONS ....... 3

II. FUNGAL INFECTIONS ....... 8

III. BACTERIAL/MYCOBACTERIAL INFECTIONS ....... 16

IV. VIRAL INFECTIONS ....... 29

V. NEOPLASTIC DISEASES ....... 42

VI. PERINATAL/GYNECOLOGIC CONDITIONS ....... 49

VII. NEONATAL DISORDERS ....... 57

VIII. BIRTH DEFECTS ....... 60

IX. MITOCHONDRIAL DISORDERS ....... 62

X. METABOLIC/ENDOCRINE DISORDERS ....... 65

XI. NEUROLOGICAL DISORDERS ....... 67

XII. OTHER HIV ASSOCIATED DISEASES ....... 72

XIII. CARDIOVASCULAR DISEASES ....... 76

XIV. TOXICITY EVALUATION ....... 82

XV. OTHER DISEASE CODES ....... 94



FSTRF maintains this code set on behalf of several clinical trials networks for which it is the data center.
Permission must be obtained through user.support@fstrf.org to use or copy it for any other purpose.

# I. PARASITIC INFECTIONS

## CHAGAS' DISEASE - CENTRAL NERVOUS SYSTEM INVOLVEMENT

**61030 CONFIRMED**

At least one of the following:

a. Direct finding of Trypanosomes in liquor, or brain biopsy

*and*

b. CSF pleocytosis, increased protein and occasionally decreased glucose levels

**61031 PROBABLE**

1. Person came from endemic area

*and*

2. CNS mass lesion with contrast enhancing effect that does not improve with Toxoplasmic treatment

*and*

3. CSF pleocytosis, increased protein and occasionally decreased glucose levels

*and*

4. Positive serology

## CHAGAS' DISEASE - MYOCARDITIS

**61032 CONFIRMED**

1. Finding of Trypanosomes nests in myocardium biopsy or in buffy coat

*and*

2. EKG right bundle branch block

**61033 PROBABLE**

1. Person came from endemic area

*and*

2. Clinical myocarditis

*and*

3. EKG with a variety of disturbances, most commonly right bundle branch block

*and*

4. Positive serology

FSTRF maintains this code set on behalf of several clinical trials networks for which it is the data center.
Permission must be obtained through user.support@fstrf.org to use or copy it for any other purpose.

## CRYPTOSPORIDIOSIS

**61035 CONFIRMED**

1. At least one of the following:
    a. Diarrhea defined as 2 or more non-formed stools per day for 2 or more days.
    b. Presence of at least one of the following abdominal symptoms: nausea, vomiting or abdominal pain.
    c. Presence of at least one of the following: biliary colic, jaundice or elevation in total bilirubin, alkaline phosphatase or gamma-glutamyl transpeptidase (GGTP) greater than or equal to 2.5 times the upper limit of normal (ULN.)

*and*

2. Microscopic evidence of cryptosporidium present in stool, body fluid or tissue specimen.

**PROBABLE**

There is no acceptable definition to be used within the ACTG.

## CYCLOSPORA GASTROENTERITIS

**61015 CONFIRMED**

1. At least one of the following:
    a. Diarrhea defined as 2 or more non-formed stools per day for 2 or more days.
    b. Presence of at least one of the following abdominal symptoms: nausea, vomiting or abdominal pain.
    c. Presence of at least one of the following: biliary colic, jaundice or elevation in total bilirubin, alkaline phosphatase or gamma-glutamyl transpeptidase (GGTP) greater than or equal to 2.5 times the upper limit of normal (ULN.)

*and*

2. Microscopic evidence of cyclospora present in stool, body fluid or tissue specimen.

**PROBABLE**

There is no acceptable definition to be used within the ACTG.

## ISOSPORIASIS

**61045 CONFIRMED**

1. At least one of the following:
    a. Diarrhea defined as 2 or more non-formed stools per day for 2 or more days.
    b. Presence of at least one of the following abdominal symptoms: nausea, vomiting or abdominal pain.
    c. Presence of at least one of the following: biliary colic, jaundice or elevation in total bilirubin, alkaline phosphatase or gamma-glutamyl transpeptidase (GGTP) greater than or equal to 2.5 times the upper limit of normal (ULN.)

*and*

2. Microscopic evidence of isospora present in stool, body fluid or tissue specimen.

**PROBABLE**

There is no acceptable definition to be used within the ACTG.

FSTRF maintains this code set on behalf of several clinical trials networks for which it is the data center.
Permission must be obtained through user.support@fstrf.org to use or copy it for any other purpose.

**LEISHMANIASIS**

There are four forms of leishmaniasis: visceral, cutaneous disease, mucosal disease and diffuse cutaneous leishmaniasis. Specify which form of disease.

**61048 CONFIRMED**

1. Histologic evidence of disease from an aspirate or biopsy.

*and*

2. Compatible clinical syndrome.

**61049 PROBABLE**

1. Compatible clinical syndrome.

*and*

2. Specific treatment initiated or recommended.

**MALARIA**

**61006 CONFIRMED**

1. Identification of Plasmodium sp. on a smear of peripheral blood

*and*

2. Compatible clinical syndrome.

**61007 PROBABLE**

1. Compatible clinical syndrome

*and*

2. Specific treatment initiated or recommended.

**MICROSPORIDIOSIS**

**61055 CONFIRMED**

1. At least one of the following:
   a. Diarrhea defined as 2 or more non-formed stools per day for 2 or more days.
   b. Presence of at least one of the following abdominal symptoms: nausea, vomiting or abdominal pain.
   c. Presence of at least one of the following: biliary colic, jaundice or elevation in total bilirubin, alkaline phosphatase or gamma-glutamyl transpeptidase (GGTP) greater than or equal to 2.5 times the upper limit of normal (ULN.)

*and*

2. Microscopic evidence of microsporidia present in stool, body fluid or tissue specimen.

**PROBABLE**

There is no acceptable definition to be used within the ACTG.

FSTRF maintains this code set on behalf of several clinical trials networks for which it is the data center.
Permission must be obtained through user.support@fstrf.org to use or copy it for any other purpose.

**PNEUMOCYSTIS CARINII PNEUMONIA (PCP)** (also known as Pneumocystis jiroveci pneumonia)

**61011 CONFIRMED**

1. A history (within the past three months) of shortness of breath, dyspnea on exertion, cough or fever

*and*

2. Histological or cytological evidence of Pneumocystis carinii on bronchoalveolar lavage, lung biopsy or sputum specimen.

**61012 PROBABLE**

1. A history (within the past three months) of shortness of breath, dyspnea on exertion, cough or fever

*and*

2. Abnormal chest X-ray (or CT scan) or hypoxemic arterial blood gas $P_aO_2$ <80 mmHg or (A-a) $DO_2$ mm Hg >15, on room air

*and*

3. Specific anti-pneumocystis therapy was recommended or initiated.

**61013**

Clinical diagnosis only, clinical history, consistent chest X-ray and improvement on PCP therapy.

**EXTRA PULMONARY PNEUMOCYSTOSIS**

**61017 CONFIRMED**

Histological or cytological evidence of extra pulmonary pneumocystosis.

**61018 PROBABLE (EYE DISEASE ONLY)**

1. Pneumocystis lesions of the retina as indicated by characteristic lesions consistent with Pneumocystis choroiditis according to an experienced ophthalmologist

*and*

2. Clinical improvement with systemic anti-pneumocystosis therapy.

FSTRF maintains this code set on behalf of several clinical trials networks for which it is the data center.
Permission must be obtained through user.support@fstrf.org to use or copy it for any other purpose.

## TOXOPLASMIC ENCEPHALITIS

**61020 CONFIRMED**

1. Histologic evidence of Toxoplasma gondii in tissue obtained by brain biopsy or autopsy

*or*

2. All of the following:
    a. Compatible clinical syndrome consisting of headache, seizure, neurologic deficits and/or fever.
    b. Presence of characteristic mass lesion(s) on brain imaging study (CT or MRI).
    c. Response after a minimum of two weeks of antitoxoplasmosis therapy with documented clinical or radiographic improvement.
    d. Positive blood culture for Toxoplasma gondii.

**61021 PROBABLE**

1. Compatible clinical syndrome consisting of headache, seizure, neurologic deficits and/or fever

*and*

2. Presence of characteristic mass lesion(s) on brain imaging study (CT or MRI)

*and*

3. Response after a minimum of two weeks of antitoxoplasmosis therapy with documented clinical or radiographic improvement.

**61024**

Clinical Diagnosis only, compatible clinical syndrome consisting of headache, seizure, neurologic deficits and/or fever and response after a minimum of two weeks of antitoxoplasmosis therapy with documented clinical improvement.

## NON-CNS TOXOPLASMOSIS

**61028 CONFIRMED**

Histologic evidence of Toxoplasma gondii present in tissue or body fluid obtained by biopsy or aspirate.

**PROBABLE**

There is no acceptable definition to be used within the ACTG.

FSTRF maintains this code set on behalf of several clinical trials networks for which it is the data center.
Permission must be obtained through user.support@fstrf.org to use or copy it for any other purpose.

# II. FUNGAL INFECTIONS

## DISSEMINATED BLASTOMYCOSIS

**62051 CONFIRMED**

Evidence of B. dermatitidis by positive culture or positive histopathology identifying characteristic appearance of organisms within body tissue or fluids.

**PROBABLE**

There is no acceptable definition to be used within the ACTG.

## CANDIDIASIS OF BRONCHI, TRACHEA OR LUNGS, SPECIFY SITE (BRONCHI, TRACHEA OR LUNGS)

**62080 CONFIRMED**

1. Characteristic white plaques in the bronchi or trachea on bronchoscopic examination.

*and*

2. Positive culture, KOH or histopathology from the bronchi or trachea.

**62081 PROBABLE**

1. Characteristic white plaques in the bronchi or trachea on bronchoscopic examination.

*and*

2. Response to specific antifungal therapy.

## ESOPHAGEAL CANDIDIASIS

**62010 CONFIRMED**

1. Compatible clinical syndrome, consisting of one or more of the following signs or symptoms: white plaques in esophagus, typical filling defects on barium swallow, odynophagia (midline retrosternal discomfort with swallowing).

*and*

2. Positive culture, KOH or histopathology from esophagus.

**62011 PROBABLE**

1. Either:
    a. Compatible clinical syndrome, consisting of two or more of the following signs or symptoms: white plaques in esophagus; typical filling defects on barium swallow; odynophagia (midline retrosternal discomfort with swallowing)

    or:

    b. Confirmed or probable oropharyngeal candidiasis and odynophagia

*and*

2. Response to specific antifungal therapy for the treatment of esophagitis.

FSTRF maintains this code set on behalf of several clinical trials networks for which it is the data center.
Permission must be obtained through user.support@fstrf.org to use or copy it for any other purpose.

## ORAL/OROPHARYNGEAL CANDIDIASIS, SPECIFY ORAL OR OROPHARYNGEAL

**CONFIRMED**

1. Compatible clinical syndrome, consisting of one or more signs/symptoms as follows:

**62060 Pseudomembranous candidiasis**

White or yellow/creamy spots or plaques that may be located in any part of the oral cavity and can usually be wiped off to reveal an erythematous surface. There may be no pain or possible mild to moderate burning pain. The lesions/symptoms are usually intermittent, but may be long-standing.

**62062 Erythematous candidiasis**

Patchy erythema or red areas usually located on the palate and dorsum of the tongue, but occasionally on the buccal mucosa. At times, white spots or plaques of pseudomembranous candidiasis may also be present. There may be no pain or possible mild to moderate burning pain. The lesions/symptoms are usually intermittent, but may be long-standing.

*and*

2. Positive culture, KOH or histopathology.

**PROBABLE**

1. Compatible clinical syndrome, consisting of two or more signs/symptoms as follows:

**62061 Pseudomembranous candidiasis**

White or yellow spots or plaques that may be located in any part of the oral cavity and can usually be wiped off to reveal an erythematous surface. There may be no pain or possible mild to moderate burning pain. The lesions/symptoms are usually intermittent, but may be long-standing.

**62063 Erythematous candidiasis**

Patchy erythema or red areas usually located on the palate and dorsum of the tongue, but occasionally on the buccal mucosa. At times, white spots or plaques of pseudomembranous candidiasis may also be present. There may be no pain or possible mild to moderate burning pain. The lesions/symptoms are usually intermittent, but may be long-standing.

*and*

2. Specific antifungal therapy initiated or recommended.

## VULVOVAGINAL CANDIDIASIS

**62070 CONFIRMED**

1. Compatible clinical syndrome, consisting of one or more signs or symptoms as follows: vulvovaginal pruritus, irritation/soreness or dyspareunia; mucous membrane erythema, white plaques/exudates adherent to vaginal mucosa or thick, curdy vaginal discharge.

*and*

2. Positive culture or KOH.

**62071 PROBABLE**

1. Compatible clinical syndrome, consisting of two or more signs or symptoms as follows: vulvovaginal pruritus, irritation/soreness or dyspareunia; mucous membrane erythema, white plaques/exudates adherent to vaginal mucosa or thick, curdy vaginal discharge

*and*

2. Specific antifungal therapy initiated or recommended.

## OTHER CANDIDIASIS

**62099**

This includes disseminated candidemia and invasive candidiasis.

FSTRF maintains this code set on behalf of several clinical trials networks for which it is the data center.
Permission must be obtained through user.support@fstrf.org to use or copy it for any other purpose.

## DISSEMINATED COCCIDIOIDOMYCOSIS

**62041 CONFIRMED**

Identification of the fungal organism C. immitis by:

a. Positive culture.

*or*

b. Positive histopathology: identification of characteristic appearance of organism within body tissue or fluids.

**PROBABLE**

There is no acceptable definition to be used within the ACTG.

## COCCIDIOIDAL MENINGITIS (PROBABLE ONLY)

**62044 PROBABLE**

1. Positive complement fixation serology

*and*

2. Compatible clinical syndrome consisting of CSF lymphocytic pleocytosis, fever and one or more of the following signs and symptoms of meningitis: headache, altered mental status, stiff neck, and/or photophobia, seizures, and/or focal deficits

*and*

3. Specific antifungal therapy initiated or recommended.

**62045**

Clinical diagnosis only, compatible clinical syndrome consisting of CSF lymphocytic pleocytosis, fever and one or more of the following signs and symptoms of meningitis: headache, altered mental status, stiff neck, and/or photophobia, seizures, and/or focal deficits and specific antifungal therapy initiated or recommended.

FSTRF maintains this code set on behalf of several clinical trials networks for which it is the data center.
Permission must be obtained through user.support@fstrf.org to use or copy it for any other purpose.

## CRYPTOCOCCAL MENINGITIS

**62023 CONFIRMED**

1. Identification of cryptococcus in CSF or CNS tissue by:
   a. Positive culture

*or*

   b. Histopathology of cryptococcal organisms.

*or*

2. Compatible clinical syndrome including fever, and one or more of the following signs or symptoms of meningitis: headache, altered mental status, stiff neck and/or photophobia, seizures and/or focal deficits.

*and*

3. Positive CSF cryptococcal antigens and/or CSF India Ink preparation.

**62024 PROBABLE**

1. Compatible clinical syndrome including fever, and one or more of the following signs or symptoms of meningitis: headache, altered mental status, stiff neck and/or photophobia, seizures and/or focal deficits.

*and*

2. Positive serum cryptococcal antigen.

*and*

3. Specific antifungal therapy initiated or recommended.

**62025**

Clinical diagnosis only, compatible clinical syndrome including fever, and one or more of the following signs or symptoms of meningitis: headache, altered mental status, stiff neck and/or photophobia, seizures and/or focal deficits and specific antifungal therapy initiated or recommended with treatment response.

## DISSEMINATED CRYPTOCOCCOSIS

**62020 CONFIRMED**

Identification of the fungal organism C. neoformans by:

   a. Positive culture.

*or*

   b. Positive histopathology: Identification of characteristic appearance of organism within body tissue or fluids.

**62021 PROBABLE**

1. Compatible clinical syndrome consisting of fever > 38°C.

*and*

2. Detection of positive cryptococcal serum antigen ≥ 1:8.

*and*

3. Specific antifungal therapy initiated or recommended.

FSTRF maintains this code set on behalf of several clinical trials networks for which it is the data center.
Permission must be obtained through user.support@fstrf.org to use or copy it for any other purpose.

---

## PARACOCCIDIOIDOMYCOSIS

**62014 CONFIRMED**

1. Fever and advanced immunosuppression (CD4 <200 cells/mL)

*and*

2. Positive culture for P. brasiliensis from sputum, bronchoalveolar lavage, cerebrospinal fluid lymph nodes, lung tissue, skin or any other tissue.

**62015 PROBABLE**

1. Clinical signs of lung, mucous, skin or lymph node involvement and fever

*or*

2. New infiltrates on thorax CT imaging or chest X-ray.

*and*

3. Observation of the characteristic "pilot wheel" shape of P. brasiliensis by direct examination of sputum of bronchoalveolar lavage (KOH prep), or by silver stain of tissue or sputum.

---

## DISSEMINATED HISTOPLASMOSIS

**62031 CONFIRMED**

Identification of the fungal organism H. capsulatum by:

a. Positive culture.

*or*

b. Positive histopathology: Identification of characteristic appearance of organism within body tissue or fluids.

**62032 PROBABLE**

1. Compatible clinical syndrome consisting of one or more signs or symptoms as follows: anemia, leukopenia, thrombocytopenia, elevated alkaline phosphatase, ALT, LDH, or bilirubin, enlarged lymph nodes, spleen and/or liver, skin lesions or gastrointestinal ulcers.

*and*

2. Detection of positive histoplasma antigen > 1 unit obtained from body fluid.

*and*

3. Specific antifungal therapy initiated or recommended.

FSTRF maintains this code set on behalf of several clinical trials networks for which it is the data center.
Permission must be obtained through user.support@fstrf.org to use or copy it for any other purpose.

**PULMONARY HISTOPLASMOSIS, BLASTOMYCOSIS, COCCIDIOIDOMYCOSIS OR CRYPTOCOCCOSIS**
[Since these diagnoses are not AIDS-defining there is no distinction between confirmed and probable.]

**62027 PULMONARY CRYPTOCOCCOSIS**
**62034 PULMONARY HISTOPLASMOSIS**
**62043 PULMONARY COCCIDIOIDOMYCOSIS**
**62053 PULMONARY BLASTOMYCOSIS**

1. Abnormal chest X-ray or CT scan.

_and_

2. Either:
    a. Positive histopathology of lung tissue or culture of lung tissue, sputum or BAL of:
    C. neoformans (cryptococcosis)
    H. capsulatum (histoplasmosis)
    C. immitis (coccidioidomycosis)
    B. dermatitidis (blastomycosis)

    or:

    b. Detection of one of the following:
    Histoplasma antigen (>1 unit) in serum, urine, BAL or sputum
    Coccidioidal positive complement fixation titer
    Cryptococcal serum antigen ≥1:8 or other antibody test

_and_

3. No evidence of extrapulmonary infection.

FSTRF maintains this code set on behalf of several clinical trials networks for which it is the data center.
Permission must be obtained through user.support@fstrf.org to use or copy it for any other purpose.

**MOLD INFECTIONS, SPECIFY SPECIES** (e.g. aspergillus species, mucormycosis and others)

**62197 CONFIRMED**

1. Evidence of invasive disease on histopathology.

*and*

2. Positive culture.

*and*

3. One of the following:
   a. Compatible clinical syndrome consistent with signs and symptoms of pulmonary fungal infection.

*or*

   b. Localized clinical syndrome in sinus, nose, orbit or ear consisting of any of the following: pain, headache, nasal or ear discharge, changes in vision or hearing, facial tenderness, ulceration or necrotic membrane in nose or face, perforation of tympanic membrane, ocular paralysis, otitis externa or media, or radiographic evidence of sinus opacity or bony erosion.

*or*

   c. Compatible clinical syndrome consistent with signs and symptoms of skin or soft tissue infection, osteomyelitis, cerebral abscess or meningitis or other organ disease.

**62198 PROBABLE**

1. Either:
   a. Positive histopathology, cytology or KOH prep from tissue.

   or:

   b. Positive culture.

*and*

2. One of the following:
   c. Compatible clinical syndrome consistent with signs and symptoms of pulmonary fungal infection.

*or*

   d. Localized clinical syndrome in sinus, nose, orbit or ear consisting of any of the following: pain, headache, nasal or ear discharge, changes in vision or hearing, facial tenderness, ulceration or necrotic membrane in nose or face, perforation of tympanic membrane, ocular paralysis, otitis externa or media, or radiographic evidence of sinus opacity or bony erosion.

*or*

   e. Compatible clinical syndrome consistent with signs and symptoms of skin or soft tissue infection, osteomyelitis, cerebral abscess or meningitis or other organ disease.

*and*

3. Specific antifungal therapy initiated or recommended.

## FUNGAL NAIL INFECTIONS

**62196 CONFIRMED**

1. Fungal culture of the nail or nail plate material.

**62195 PROBABLE**

1. Paronychia (painful red and swollen nail bed) or onycholysis (separation of the nail from the nail bed) of the fingernails (white discoloration- especially involving proximal part of nail plate- with thickening and separation of the nail from the nail bed).

## PENICILLIOSIS MARNEFFEI, DISSEMINATED

**62180 CONFIRMED**

Isolation of Penicillium marneffei from blood, bone marrow, tissue, or other normally sterile body fluids.

**62181 PROBABLE**

One of the following major criteria:

1. A finding of elongated yeast like organism with clear central septum in Wright's-stained skin biopsy touch smear or scraping of a skin lesion.

*and*

At least two of the following minor criteria:

2. Fever, weight loss, papulonecrotic skin lesions, lymphadenopathy, hepatomegaly, splenomegaly, anemia, leukopenia, and thrombocytopenia.

## OTHER FUNGI

**62997 CONFIRMED**

1. Histologic evidence of invasive disease.

*and*

2. Positive culture or smear from a sterile tissue site.

*and*

3. Compatible clinical syndrome.

**62998 PROBABLE**

1. Compatible clinical syndrome.

*and*

2. Positive culture or smear from a non-sterile site.

*and*

3. Specific antifungal treatment initiated or recommended.

FSTRF maintains this code set on behalf of several clinical trials networks for which it is the data center.
Permission must be obtained through user.support@fstrf.org to use or copy it for any other purpose.

# III. BACTERIAL/MYCOBACTERIAL INFECTIONS

## BACTERIAL INFECTION OF DEEP TISSUE, BODY CAVITY OR OTHER NORMALLY STERILE SITE, SPECIFY SITE

**65686 CONFIRMED**

This category includes, for example, organ parenchymal, deep soft tissue (including pyomyositis) or abdominal abscesses, empyema, purulent pericarditis, meningitis and bone and joint infections.

Demonstration of bacterial pathogen(s) in deep tissue, viscera, body cavity, or other normally sterile site by one of the following methods:

a. Isolation of a bacterial pathogen(s) from an aspirate or biopsy specimen.
b. Appropriate histopathology stain of a specimen.
c. Demonstration of bacterial pathogen(s) by appropriate Gram or microbiological stain of aspirate or biopsy specimen.

**65687 PROBABLE**

1. Evidence of an infection in a deep tissue, body cavity or other normally sterile site demonstrated by appropriate diagnostic sampling or imaging procedures such as fluid aspiration, biopsy, computerized tomography, ultrasonography, magnetic resonance imaging, radioisotope scanning or plain radiograph.

*and*

2. Clinical signs and symptoms compatible with the infection.

*and*

3. Appropriate treatment initiated and response demonstrated. (Appropriate treatment may include drainage procedures and/or antibacterial therapy.)

## CATHETER EXIT SITE AND/OR TUNNEL INFECTION

**65401 CONFIRMED**

1. Erythema, tenderness, induration and/or purulent drainage along the subcutaneous tract or at the skin exit site.

*and*

2. At least one of the following:
   a. Isolation of a bacterial pathogen(s) from the exit site, tunnel and/or catheter tip.
   b. Appropriate antibacterial therapy is initiated or recommended.

**PROBABLE**

There is no acceptable definition to be used within the ACTG.

FSTRF maintains this code set on behalf of several clinical trials networks for which it is the data center.
Permission must be obtained through user.support@fstrf.org to use or copy it for any other purpose.

**ACUTE GASTROINTESTINAL/DIARRHEAL SYNDROME,** Specify
When completing the form specify either acute gastrointestinal syndrome or acute diarrheal syndrome

**65001 CONFIRMED**

1. Clinical syndrome with acute onset of 3 or more bowel movements (or stools) in a 24 hour period

*and*

2. Duration lasting greater than or equal to (≥) 3 days and less than or equal to (≤) 14 days.

*and*

3. Pathogen identified

**65002 PROBABLE**

1. Clinical syndrome with acute onset of 3 or more bowel movements (or stools) in a 24 hour period

*and*

4. Duration lasting greater than or equal to (≥) 3 days and less than or equal to (≤) 14 days.

*and*

2. No pathogen identified

**CHRONIC DIARRHEA**

**65005 CONFIRMED**

1. Clinical syndrome of 3 or more bowel movements (or stools) in a 24 hour period.

*and*

2. Duration greater than or equal to (≥ ) 28 days

*and*

3. Pathogen identified

**65006 PROBABLE**

1. Clinical syndrome of 3 or more bowel movements (or stools) in a 24-hour period.

*and*

2. Duration greater than or equal to (≥) 28 days

*and*

3. Either diagnostic testing was done and no pathogen was identified or diagnostic testing was not available.

**PERSISTENT DIARRHEA**

**65007**

1. Three (3) or more bowel movements (or stools) in a 24 hour period.

*and*

2. Duration greater than (>) 14 to less than (<) 28 days.

FSTRF maintains this code set on behalf of several clinical trials networks for which it is the data center.
Permission must be obtained through user.support@fstrf.org to use or copy it for any other purpose.

## ACUTE DYSENTERY

### 65003 CONFIRMED

1. Clinical syndrome with acute onset of 3 or more bowel movements (or stools) in a 24 hour period.

*and*

2. Visible blood in the stool

*and*

3. Duration lasting less than or equal to (≤ ) 14 days

*and*

4. Pathogen identified

### 65004 PROBABLE

1. Clinical syndrome with acute onset of 3 or more bowel movements (or stools) in a 24 hour period.

*and*

2. Visible blood in the stool

*and*

3. Duration lasting less than or equal to (≤) 14 days

*and*

4. No pathogen identified

FSTRF maintains this code set on behalf of several clinical trials networks for which it is the data center.
Permission must be obtained through user.support@fstrf.org to use or copy it for any other purpose.

## BACTERIAL ENDOCARDITIS

**65424 CONFIRMED**

1. Persistently positive blood cultures (at least two sets of blood cultures obtained on separate occasions, with either two of four positive cultures, three of six positive cultures, or at least 70% of cultures positive, if four or more sets of blood cultures were obtained.)

*and*

2. Demonstration of a bacterial pathogen(s) by Gram stain, other histologic stain or culture of valvular vegetation or endocardial tissue.

**65425 PROBABLE (A OR B)**

**EITHER A:**

1. Persistently positive blood cultures (at least two sets of blood cultures obtained on separate occasions, with either two of four positive cultures, three of six positive cultures, or at least 70% of cultures positive, if four or more sets of blood cultures were obtained.)

*and*

2. At least one of the following:
    a. New murmur by physical examination.
    b. Echocardiographic (or other imaging procedure) demonstration of valvular vegetation(s).
    c. Evidence of distal embolus (e.g. petechiae, splinter hemorrhages, conjunctival hemorrhages, Roth spots, Osler's nodes, Janeway lesions, hematuria with active urine sediment consistent with glomerulonephritis, or embolic phenomena.)
    d. Septic pulmonary emboli.

*and*

3. Specific antibacterial therapy initiated or recommended.

**OR B:**

1. Negative or intermittently positive blood cultures.

*and*

2. At least three of the following:
    a. Fever greater than (>) 38°C.
    b. New murmur by physical examination.
    c. Evidence of distal embolus (e.g. petechiae, splinter hemorrhages, conjunctival hemorrhages, Roth spots, Osler's nodes, Janeway lesions, hematuria with active urine sediment consistent with glomerulonephritis, or embolic phenomena.)
    d. Echocardiographic (or other imaging procedure) demonstration of valvular vegetation(s).
    e. Demonstration of a bacterial pathogen(s) by Gram stain, other histologic stain or culture of peripheral embolus obtained by biopsy.
    f. Septic pulmonary emboli.

*and*

3. Specific antibacterial therapy initiated or recommended.

FSTRF maintains this code set on behalf of several clinical trials networks for which it is the data center.
Permission must be obtained through user.support@fstrf.org to use or copy it for any other purpose.

## HANSEN'S DISEASE/LEPROSY

### 63031 CONFIRMED

1. Appropriate clinical setting with characteristic dermatological and/or neurological manifestations with consistent histopathology:
   Multibacillary leprosy: Acid fast bacilli seen on the smear
   Paucibacillary leprosy: Well formed non-caseating granuloma and nerve involvement (AFB need not be seen).

### 63030 PROBABLE

1. Characteristic infiltrative skin lesion, hypoesthesia, or peripheral neuropathy in the appropriate clinical setting.

## MYCOBACTERIUM AVIUM COMPLEX (MAC)

### 63018 CONFIRMED

MAC identified from a normally sterile site (blood, bone marrow, lymph node, liver, cerebrospinal fluid or other normally sterile body fluid, tissue or organ). Conventional (e.g., culture) and DNA probe technologies are acceptable for identification of MAC from cultures.

### 63019 PROBABLE

1. MAC identified from bronchopulmonary, gastrointestinal, skin surface or other non-sterile site(s) (as the only pathogen) coupled with histopathologic confirmation of AFB/MAC in tissue specimen(s) from which MAC was identified. Conventional (e.g., culture) and DNA probe technologies are acceptable for identification of MAC from cultures.

*and*

2. A clinical MAC syndrome consisting of one or more of the following: persistent fever greater than or equal to (≥) 38°C for more than one week, night sweats, diarrhea, weight loss or wasting, radiographically documented pulmonary infiltrates, hepatomegaly, splenomegaly, anemia (hemoglobin less than ( <) 8.5 gm/dL), and alkaline phosphatase elevated to greater than twice the upper limit of normal (72 x ULN).

*and*

3. Treatment initiated or recommended for MAC

FSTRF maintains this code set on behalf of several clinical trials networks for which it is the data center.
Permission must be obtained through user.support@fstrf.org to use or copy it for any other purpose.

## MYCOBACTERIAL INFECTION, OTHER NON-TUBERCULOUS, NON-MAC

**CONFIRMED**
**63021 CONFIRMED,** *M. kansasii*
**63023 CONFIRMED,** *M. genovensii*
**63027 CONFIRMED***, other non-MAC, non-TB mycobacteria*

1. Other mycobacterial species cultured from blood, bone marrow, lymph node, liver, cerebrospinal fluid, or any other normally sterile body fluid, tissue or organ.

**PROBABLE**
**63022 PROBABLE,** *M. kansasii*
**63024 PROBABLE,** *M. genovensii*
**63028 PROBABLE, OTHER** *other non-MAC, non-TB mycobacteria*

1. Other mycobacterial species cultured from bronchopulmonary, gastrointestinal, urine, skin surface or other non-sterile site(s).

*and*

2. Clinical symptoms, signs, or radiograph/laboratory abnormalities compatible with mycobacterial infection consisting of one or more of the following: persistent fever greater than or equal to (≥) 38°C for more than one week, night sweats, diarrhea, weight loss or wasting, radiographically documented pulmonary infiltrates, hepatomegaly, splenomegaly, anemia (hemoglobin less than (<) 8.5 gm/dL), and alkaline phosphatase elevated to greater than twice the upper limit of normal (ULN).

*and*

3. No alternative pathogen(s) identified or symptoms/signs persist after treatment for and/or elimination of alternative pathogen(s).

*and*

4. Treatment initiated or recommended for non-tuberculous, non-MAC mycobacteria.

**63029**
Clinical diagnosis only, clinical symptoms, signs, or radiograph/laboratory abnormalities compatible with mycobacterial infection consisting of one or more of the following: persistent fever greater than or equal to (≥) 38˚C for more than one week, night sweats, diarrhea, weight loss or wasting, radiographically documented pulmonary infiltrates, hepatomegaly, splenomegaly, anemia (hemoglobin less than (<) 8.5 gm/dL), and alkaline phosphatase elevated to greater than twice the upper limit of normal and no alternative pathogen(s) identified or symptoms/signs persist after treatment for and/or elimination of alternative pathogen(s) and treatment initiated or recommended for non-tuberculous, non-MAC mycobacteria.

## OSTEOMYELITIS

**61300 CONFIRMED**
Specific test on blood or bone or by histology. Specify bone(s) involved and pathogen(s) identified.

**61302 PROBABLE**
Suspected clinically and radiologically, negative or no specific test on blood and/or bone. Specify bone(s) involved.

FSTRF maintains this code set on behalf of several clinical trials networks for which it is the data center.
Permission must be obtained through user.support@fstrf.org to use or copy it for any other purpose.

## PELVIC INFLAMMATORY DISEASE

**69606 CONFIRMED**

1. Evidence of purulent material in the peritoneal cavity by culdocentesis or laparoscopic examination.

*or*

2. Endometrial biopsy findings consistent with acute endometrial infection.

*or*

3. 
    a. Examination elicits cervical motion, adnexal, and/or uterine tenderness.

*and*

    b. At least one of the following:
        i. Pelvic abscess or inflammatory complex (tubo-ovarian abscess.)
        ii. Evidence of cervical infection with N. gonorrhea or C. trachomatis.
        iii. Purulent cervical discharge.

**69607 PROBABLE**

1. Examination elicits cervical motion, adnexal, and/or uterine tenderness.

*and*

2. At least one of the following:
    a. Temperature greater than 38°C.
    b. Bimanual exam detects pelvic mass consistent with pelvic abscess or inflammatory complex (tubo-ovarian abscess.)
    c. Microscopic examination of the wet mount demonstrating markedly increased numbers of inflammatory cells.

*and*

3. Treatment initiated or recommended.

**69608**

Clinical diagnosis only, testing technology not available to determine diagnosis.

## BACTERIAL PNEUMONIA

**65100 CONFIRMED**

1. Chest radiographic examination shows new or progressive infiltrate, consolidation or cavitation.

*and*

2. At least one of the following:
    a. Bacterial organism(s) cultured from blood with no alternative site of infection.
    b. Isolation of a bacterial pathogen(s) from a culture specimen obtained by transtracheal aspirate, protected bronchial brushing or biopsy.
    c. Histopathologic evidence of pneumonia with bacterial organism(s) demonstrated by Gram stain or culture of tissue specimen or positive Quellung test for pneumococcus.
    d. Demonstration of a predominant bacterial organism by positive culture or Gram stain of an adequate sputum specimen (fewer than 10 epithelial cells and greater than (>) 25 PMNs per high power field).
    e. Fluorescent antibody or other antigen detection method positive for Legionella, Chlamydia or Mycoplasma spp. And no other pathogen identified.

FSTRF maintains this code set on behalf of several clinical trials networks for which it is the data center.
Permission must be obtained through user.support@fstrf.org to use or copy it for any other purpose.

**65101 PROBABLE**

1. Chest radiographic examination shows new or progressive infiltrate, consolidation or cavitation.

*and*

2. At least one of the following:
   a. Fever and/or cough.
   b. New onset of purulent sputum or change in character of sputum.
   c. Appropriately collected (acute and convalescent) serologic tests positive for Legionella, Chlamydia or Mycoplasma and no other pathogen identified.

*and*

1. Appropriate antibacterial therapy initiated or recommended.

**65102**

Clinical diagnosis only; including history/examination and improvement with antibacterial therapy.

---

**BACTERIAL SEPSIS/CATHETER RELATED BACTEREMIA/SEPSIS**

**65200 CONFIRMED**
**BACTERIAL SEPSIS**

NOTE: These criteria apply only to bloodstream infections that are unrelated to infection at another site. See criteria for bacterial endocarditis and catheter related sepsis as necessary.

Laboratory-confirmed bloodstream infection must meet at least one of the following criteria:

1. A recognized bacterial pathogen(s) isolated from one or more blood cultures.

*or*

2. Both:
   a. The presence of at least one of the following signs or symptoms: fever greater than (>) 38°C, chills/rigors or hypotension (systolic pressure greater than or equal to (≤) 90 mm Hg).

*and*

   b. Common skin flora (e.g. diphtheroids, coagulase-negative staphylococci, Bacillus spp., proprionibacterium spp., or micrococci) isolated from two or more blood cultures drawn on separate occasions.

**CATHETER RELATED BACTEREMIA/SEPSIS**

1. Isolation of a known bacterial pathogen(s) from a blood culture in a study participant with an indwelling intravascular catheter and no other alternative site of infection.

*or*

2. All of the following:
   a. The presence of at least one of the following signs or symptoms: fever greater than (>) 38°C, chills/rigors or hypotension (systolic pressure greater than or equal to (≤) 90 mm Hg).

*and*

   b. Common skin flora (e.g. diphtheroids, coagulase-negative staphylococci, Bacillus spp., Proprionibacterium spp., or micrococci) isolated from two or more blood cultures drawn on separate occasions from a study participant with an indwelling catheter.

*and*

   c. No other alternative site of infection.

FSTRF maintains this code set on behalf of several clinical trials networks for which it is the data center.
Permission must be obtained through user.support@fstrf.org to use or copy it for any other purpose.

**65201 PROBABLE**
**BACTERIAL SEPSIS**
NOTE: These criteria apply only to bloodstream infections that are unrelated to infection at another site. See criteria for bacterial endocarditis and catheter related sepsis as necessary.

1. The presence of at least one of the following signs or symptoms: fever > 38°C, chills/rigors or hypotension (systolic pressure ≤ 90 mm Hg).

*and*

2. At least one of the following:
   a. Common skin flora (e.g. diphtheroids, coagulase-negative staphylococci, Bacillus spp., Proprionibacterium spp., or micrococci) isolated from one blood culture.
   b. Positive antigen test on blood (e.g. *Hemophilus influenzae, Streptococcus pneumoniae, Neisseria meningitides* or Group B Streptococcus).

*and*

3. Signs and symptoms and positive laboratory results are not related to an alternative etiology.

*and*

4. Appropriate antibacterial therapy initiated or recommended.

**CATHETER RELATED BACTEREMIA/SEPSIS**
1. The presence of at least one of the following signs or symptoms: fever > 38°C, chills/rigors or Hypotension (systolic pressure ≤ 90 mm Hg).

*and*

2. Common skin flora (e.g. diphtheroids, coagulase-negative staphylococci, Bacillus spp., Proprionibacterium spp., or micrococci) isolated from one blood culture drawn from a study participant with an indwelling catheter.

*and*

3. Appropriate antibacterial therapy is initiated or recommended.

**65202**
Clinical diagnosis only, testing technology not available to determine diagnosis.

---

**SALMONELLA SEPSIS (NON-TYPHOID)**

**65204 CONFIRMED**
1. Positive blood culture

**65203 PROBABLE**
1. Clinical exam

*and*

2. Stool culture positive

---

### BACTERIAL SINUSITIS

**65473 CONFIRMED**
**EITHER A:**

1. Isolation of a bacterial pathogen(s) from specimen(s) obtained by drainage procedure(s) of involved sinus(es).

<u>*or*</u>

2. Demonstration of PMNs and bacterial organism(s) on Gram stain (or other microbial staining technique) from specimen(s) obtained by drainage procedure(s) of involved sinus(es).

**OR B:**

1. Acute and/or chronic radiographic changes of one or more sinuses as depicted by plain radiograph, CT or MRI scan.

<u>*and*</u>

2. Isolation of a bacterial pathogen(s) from one or more blood cultures with either:
   a. no material obtained for cultures by a drainage procedure(s) of involved sinus(es),

<u>*or*</u>

   b. specimen obtained yielded no growth and there is no other focus of infection.

**65474 PROBABLE**
**EITHER A:**

1. A compatible clinical syndrome consisting of fever > 38°C and one or more of the following:
   a. Nasal congestion.
   b. Postnasal drainage.
   c. Facial pain, tenderness or headache.

<u>*and*</u>

2. Acute and/or chronic changes of one or more sinuses as depicted by plain radiograph, CT or MRI scan.

<u>*and*</u>

3. Appropriate antibacterial therapy initiated or recommended.

**OR B:**

1. A compatible clinical syndrome consisting of fever > 38°C and two or more of the following:
   a. Nasal congestion.
   b. Postnasal drainage.
   c. Facial pain, tenderness or headache.

<u>*and*</u>

2. Appropriate antibacterial therapy initiated or recommended.

**65475**

Clinical diagnosis only, testing technology not available to determine diagnosis.

FSTRF maintains this code set on behalf of several clinical trials networks for which it is the data center.
Permission must be obtained through user.support@fstrf.org to use or copy it for any other purpose.

## RECURRENT UPPER RESPIRATORY TRACT INFECTIONS

Current event plus one or more in last six-month period.

**CONFIRMED**
**65017 TONSILLITIS**
**65019 OTITIS MEDIA**
**65021 PHARYNGITIS**

1. Laboratory studies where available, such as culture of suitable body fluid.

**PROBABLE**
**65016 TONSILLITIS**
**65018 OTITIS MEDIA**
**65021 PHARYNGITIS**

1. Symptom complex, such as unilateral face pain with nasal discharge (sinusitis), painful, inflamed eardrum (otitis media), or tonsillopharyngitis without features of viral infection (such as coryza or cough).

## LATENT TUBERCULOSIS (TB) INFECTION

**63016 CONFIRMED**

1. Positive PPD defined by greater than or equal to (≥)5 mm induration for HIV-infected persons or other approved tuberculosis screening test.

*and*

2. No clinical, bacteriologic, or radiographic evidence of active tuberculosis

FSTRF maintains this code set on behalf of several clinical trials networks for which it is the data center.
Permission must be obtained through user.support@fstrf.org to use or copy it for any other purpose.

## PULMONARY TUBERCULOSIS (TB)

### 63007 CONFIRMED

Demonstration of mycobacterium tuberculosis (acid fast bacilli) using the Ziehl Nielson or Auramine stain, a positive culture or any future approved diagnostic technologies in any of the following secretions or tissue samples:

- a. Sputum
- b. Pleural, pericardial and peritoneal fluid
- c. Aspirate of lymph node, cold abscess, joint fluid
- d. Cerebrospinal fluid
- e. Bone marrow aspirate
- f. Stool specimen
- g. Gastric lavage
- h. Bronchoalveolar lavage fluid
- i. Histopathology of tissue from any site where mycobacterium tuberculosis has been identified within granulomatous inflammatory lesions. These usually include inter alia
  - i. Lymph node biopsy
  - ii. Pleural and peritoneal biopsy
  - iii. Liver biopsy
  - iv. Bone marrow biopsy

### 63008 PROBABLE

1. AFB not demonstrable.

*or*

2. Compatible clinical syndrome of one or more of the following:
   - a. Radiologic chest X-ray consistent with pulmonary TB
   - b. Fever > 38˚C for more than 2 weeks
   - c. Unintentional weight loss of more than 10% of body weight
   - d. Night sweats
   - e. Positive TB exposure
   - f. Absence of another possible diagnosis

*and*

3. Specific antituberculous therapy initiated.

### 63005

Clinical diagnosis only. The clinician's judgement that the study participant may have pulmonary TB but above criteria are not fulfilled.

1. A fever of unknown origin or a study participant who is deteriorating and pulmonary TB is considered a possibility and therefore empiric TB treatment is instituted.

*and*

2. A typical chest radiographic pattern of pulmonary TB but the CONFIRMED or PROBABLE criteria above are not satisfied and the clinician feels active TB is likely.

FSTRF maintains this code set on behalf of several clinical trials networks for which it is the data center.
Permission must be obtained through user.support@fstrf.org to use or copy it for any other purpose.

## EXTRA PULMONARY TUBERCULOSIS

**63012 CONFIRMED**

[Including Miliary (disseminated) tuberculosis]
Positive culture for Mycobacterium tuberculosis from extrapulmonary site.

**63013 PROBABLE**

[Including Miliary (disseminated) tuberculosis]

1. Positive AFB smear or a positive histopathology from an extrapulmonary site.

*and*

2. Specific multi-drug antituberculous therapy initiated.

**63015**

[Including Miliary (disseminated) tuberculosis]
Clinical diagnosis only, one or more of the following signs or symptoms consistent with a clinical syndrome for extra pulmonary TB: fever greater than (>) 38˚C, night sweats, malaise, weight loss and/or adenopathy and specific multi-drug antituberculous therapy initiated.

FSTRF maintains this code set on behalf of several clinical trials networks for which it is the data center.
Permission must be obtained through user.support@fstrf.org to use or copy it for any other purpose.

# IV. VIRAL INFECTIONS

## CMV COLITIS, ADULT ACTG CRITERIA

**64014 CONFIRMED**

3. Presence of at least one of the following symptoms: abdominal pain or diarrhea (typically in small volume and associated with mucus and blood).

*and*

4. Tissue biopsy demonstrating CMV by antigen, detection of viral nucleic acids (e.g. PCR) or characteristic cytopathic changes.

**64114 PROBABLE**

1. Presence of at least one of the following symptoms: abdominal pain or diarrhea (typically in small volume and associated with mucus and blood).

*and*

2. Appropriate visualization procedure (endoscopy) that reveals mucosal erythema, erosion or ulceration.

*and*

3. CMV is isolated from the lesion.

*and*

4. Anti-CMV therapy initiated or recommended.

## CMV COLITIS, INTERNATIONAL

**64214 CONFIRMED**

1. Symptoms: persistent or chronic diarrhea for > 14 days, abdominal pain and fever.

*and*

2. At least one of the following positive results:
    a. Isolation of CMV from the GI tissue
    b. Detection of CMV antigen
    c. Isolation of CMV DNA

**64219 PROBABLE**

1. Symptoms: persistent or chronic diarrhea for > 14 days, abdominal pain and fever.

*and*

2. Colonoscopy report that demonstrates widespread submucosal hemorrhages and diffuse mucosal ulcerations.

FSTRF maintains this code set on behalf of several clinical trials networks for which it is the data center.
Permission must be obtained through user.support@fstrf.org to use or copy it for any other purpose.

## CMV ENCEPHALITIS

**64017 CONFIRMED**

1. Rapidly progressive cognitive impairment, progressive change in mental status or delirium, or signs and symptoms of brain stem injury.

*and*

2. Detection of viral nucleic acids (e.g. PCR) in CSF or CSF CMV culture positive or brain biopsy demonstrating CMV by antigen, detection of viral nucleic acids (e.g. PCR) or characteristic cytopathic changes.

**64117 PROBABLE**

1. Rapidly progressive cognitive impairment, progressive change in mental status or delirium, or signs and symptoms of brain stem injury.

*and*

2. MRI or contrast CT scan performed which:
   a. Excludes toxoplasmosis, lymphoma, PML or other intracranial process.

*and*

   b. Demonstrates periventricular inflammation or meningeal enhancement.

*and*

3. Other etiologies ruled out.

*and*

4. CMV end-organ disease (e.g. retinitis, colitis) present.

*and*

5. Specific therapy initiated, changed or recommended.

**64217**

Clinical diagnosis only, rapidly progressive cognitive impairment, progressive change in mental status or delirium, or signs and symptoms of brain stem injury, other etiologies ruled out, CMV end-organ disease (e.g. retinitis, colitis) present and specific therapy initiated, changed or recommended.

FSTRF maintains this code set on behalf of several clinical trials networks for which it is the data center.
Permission must be obtained through user.support@fstrf.org to use or copy it for any other purpose.

## CMV ESOPHAGITIS

**64012 CONFIRMED**

1. Presence of at least one of the following symptoms: retrosternal pain or odynophagia (midline retrosternal discomfort with swallowing).

*and*

2. Tissue biopsy demonstrating CMV by detection of antigen, viral nucleic acids (e.g. PCR) or characteristic cytopathic changes.

**64112 PROBABLE**

1. Presence of at least one of the following symptoms: retrosternal pain or odynophagia (midline retrosternal discomfort with swallowing.)

*and*

2. Appropriate visualization procedure (endoscopy) that reveals mucosal erythema, erosion and/or ulceration.

*and*

3. CMV is isolated from the lesion.

*and*

4. Anti-CMV therapy initiated or recommended.

**64210**

Clinical diagnosis only with primary symptom of dysphagia and failure to respond to empiric antifungal therapy within 72 hours after presenting with dysphagia, fever and weight loss or anatomopathological exam with characteristic cytopathic changes.

## CMV GASTROENTERITIS

**64015 CONFIRMED**

1. Presence of abdominal pain.

*and*

2. Tissue biopsy demonstrating CMV by antigen, detection of viral nucleic acids (e.g. PCR) or characteristic cytopathic changes.

**64115 PROBABLE**

1. Presence of abdominal pain.

*and*

2. Appropriate visualization procedures (endoscopy) that reveal mucosal erythema, erosion or ulceration.

*and*

3. CMV is isolated from the lesion.

*and*

4. Anti-CMV therapy initiated or recommended.

**64215**

Clinical diagnosis only, testing technology not available to determine diagnosis.

FSTRF maintains this code set on behalf of several clinical trials networks for which it is the data center.
Permission must be obtained through user.support@fstrf.org to use or copy it for any other purpose.

## MUCOCUTANEOUS CMV ULCERS

**64018 CONFIRMED**

1. Direct visualization of oral, vulvovaginal, or perianal ulcers.

*and*

2. CMV culture of lesion or histologic demonstration of typical CMV cytopathology on biopsy of lesion.

**PROBABLE**

There is no acceptable definition to be used within the ACTG.

## CMV PNEUMONITIS

**64011 CONFIRMED**

1. Hypoxemia and infiltrates on chest X-ray or CT/MRI scan.

*and*

2. Tissue biopsy or cells obtained by BAL demonstrating CMV by antigen, detection of viral nucleic acids (e.g. PCR) or characteristic cytopathic changes.

*and*

3. No other pathogens identified by routine testing *or* signs/symptoms persist or recur after treatment of copathogens.

**64111 PROBABLE**

1. Hypoxemia and infiltrates on chest X-ray or CT/MRI scan.

*and*

2. Positive culture, detection of viral antigen, or detection of viral nucleic acids (e.g. PCR) of CMV from fluid obtained by BAL.

*and*

3. No other pathogens identified by routine testing *or* signs/symptoms persist or recur after treatment of copathogens.

*and*

4. Specific antiviral treatment initiated or recommended.

**64211**

Clinical diagnosis only, testing technology not available to determine diagnosis.

FSTRF maintains this code set on behalf of several clinical trials networks for which it is the data center.
Permission must be obtained through user.support@fstrf.org to use or copy it for any other purpose.

## CMV PROCTITIS

**64016 CONFIRMED**

1. Presence of rectal pain often associated with tenesmus, mucus and blood.

*and*

2. Tissue biopsy demonstrating CMV by antigen, detection of viral nucleic acids (e.g. PCR) or characteristic cytopathic changes.

**64116 PROBABLE**

1. Presence of rectal pain often associated with tenesmus, mucus and blood.

*and*

2. Appropriate visualization procedures (endoscopy) that reveal mucosal erythema, erosion or ulceration.

*and*

3. CMV is isolated from the lesion.

*and*

4. Anti-CMV therapy initiated or recommended.

**64216**

Clinical diagnosis only, testing technology not available to determine diagnosis.

## CMV RETINITIS

**64013 CONFIRMED**

Typical lesions including white areas with or without hemorrhages and/or gray-white areas of retinal necrosis with or without hemorrhages. Lesion(s) has/have irregular, dry-appearing granular border, with little or no overlying vitreous inflammation. Must be diagnosed by an experienced ophthalmologist using indirect ophthalmoscopy and documented by retinal photography that can be independently verified.

**64113 PROBABLE**

Typical lesions including white areas with or without hemorrhages and/or gray-white areas of retinal necrosis with or without hemorrhages. Lesion(s) has/have irregular, dry-appearing granular border, with little or no overlying vitreous inflammation. Must be diagnosed by an experienced ophthalmologist using indirect ophthalmoscopy, but is not documented by retinal photographs.

**64213**

Clinical diagnosis only, history and endoscopic appearance suggestive of CMV disease.

FSTRF maintains this code set on behalf of several clinical trials networks for which it is the data center.
Permission must be obtained through user.support@fstrf.org to use or copy it for any other purpose.

## OTHER CMV SYNDROMES, SPECIFY

**64019 CONFIRMED** (this includes but is not limited to the following)
Hepatitis or cholangitis:

1. ALT or alkaline phosphatase significantly elevated above the study participant's baseline values.

*and*

2. Tissue biopsy demonstrating CMV by antigen, detection of viral nucleic acids (e.g. PCR) or characteristic cytopathic changes.

Radiculomyelopathy:

1. Clinical presentation compatible with CMV end-organ disease, including all of the following:
    a. Decreased lower extremity strength and reflexes or syndrome consistent with a cord lesion present subacutely (over days to weeks).
    b. Myelogram or MRI reveals no mass lesions but lower spinal nerve roots thickened.
    c. CMV positive culture in CSF *or* detection of CMV viral nucleic acids (e.g. PCR) in CSF.

**PROBABLE**
There is no acceptable definition to be used within the ACTG.

## EPSTEIN BARR VIRUS (EBV)

**66380 CONFIRMED**
Infection (infectious mononucleosis), proven by EBV serology (monospot not acceptable).

**66381 PROBABLE**
Positive monospot only.

## ACUTE HEPATITIS

**64066 CONFIRMED**

1. Hepatic inflammation diagnosed within 6 months of having a known normal (asymptomatic) liver or chronic stable hepatitis.

   Hepatic inflammation is defined by:
    a) aminotransferase elevation of at least 5 X ULN

   *and/or*

    b) seropositive for IgM antibody to HAV (acute hepatitis A) or IgM antibody to HBc with presence of new HBsAg (acute hepatitis B) or evidence of HCV viremia without HCV antibody or documented seroconversion within 6 months to anti-HCV with an aminotransferase elevation above the normal level (acute Hepatitis C).

## CHRONIC HEPATITIS B

**64060 CONFIRMED**

Confirmed Chronic Replicative Hepatitis B

1. Hepatitis B surface antigen ($Hb_sAg$) detected 6 or more months ago.

*and*

2. Hepatitis B virus DNA (HBV DNA) *or* a Hepatitis B e antigen ($Hb_eAg$) is detected 6 or more months after the initial detectable Hepatitis B surface antigen ($Hb_sAg$).

Confirmed Chronic Non-Replicative Hepatitis B

1. Hepatitis B surface antigen ($Hb_sAg$) detected by repeat testing performed 6 or more months after the initial detectable Hepatitis B surface antigen ($Hb_sAg$).

*and*

2. Tests for Hepatitis B virus DNA (HBV DNA) *and* Hepatitis B e antigen ($Hb_eAg$) are negative 6 or more months after the initial $Hb_sAg$.

**64061 PROBABLE**

Probable Chronic Replicative Hepatitis B

1. A Hepatitis B surface antigen ($Hb_sAg$) detected on two occasions at least 6 months apart.

*and*

2. Anticore antibody (Anti-$Hb_cAB$) test positive for either IgG alone or for total antibodies (IgG detectable and IgM not detectable).

**64062**

Clinical diagnosis only, testing technology not available to determine diagnosis.

## CHRONIC HEPATITIS C

**64063 CONFIRMED**

1. Hepatitis C infection documented 6 or more months ago by HCV ELISA (confirmed by RIBA or PCR) or HCV RNA.

*and*

2. HCV ELISA or HCV RNA is detectable 6 or more months after the initial testing noted above.

**64064 PROBABLE**

1. ALT (SGPT) results greater than the upper limit of normal (ULN) on two or more occasions at least 6 months apart.

*and*

2. Hepatitis C infection detected any time by HCV ELISA (confirmed by RIBA or PCR) or HCV RNA.

**64065**

Clinical diagnosis only, testing technology not available to determine diagnosis.

## HHV6/ROSEOLA INFANTUM

**66481**

Suspected infection because of clinical diagnosis of roseola, virus detection studies not done or negative.

FSTRF maintains this code set on behalf of several clinical trials networks for which it is the data center.
Permission must be obtained through user.support@fstrf.org to use or copy it for any other purpose.

## HSV ESOPHAGITIS

**64020 CONFIRMED**

1. Presence of at least one of the following symptoms: retrosternal pain or odynophagia (midline retrosternal discomfort with swallowing).

_and_

2. Tissue biopsy demonstrating HSV by detection of antigen, viral nucleic acids (e.g. PCR) or characteristic cytopathic changes.

**64021 PROBABLE**

1. Presence of at least one of the following symptoms: retrosternal pain or odynophagia (midline retrosternal discomfort with swallowing).

_and_

2. Appropriate visualization procedure (endoscopy) that reveals mucosal erythema, erosion or ulceration.

_and_

3. HSV is isolated from the lesion.

_and_

4. Anti-HSV therapy initiated or recommended.

## MUCOCUTANEOUS HERPES SIMPLEX

**64023 CONFIRMED**

1. Typical (vesicular or ulcerative) HSV lesion(s) in any of the following sites: anogenital (external genitalia, cervix, vagina, perineum, perirectal, rectal), oral, perioral or finger.

_and_

2. Any one of the following:
   a. HSV isolated from lesion.
   b. HSV antigen detected by immunoassay from vesicular fluid or cells obtained from the base of a vesicle or ulcer.
   c. Recurrence of lesion in same general location: anogenital (external genitalia, cervix, vagina, perineum, perirectal, rectal), oral, perioral or finger with prior documented positive HSV culture.

**64024 PROBABLE**

1. Clinically apparent typical (vesicular or ulcerative) HSV lesion(s) with prodromal and/or concurrent symptoms of discomfort (burning, itching, pain).

_and_ either 2 or 3:

For an Initial episode:

2. Typical herpes virus inclusions and/or multinucleated giant cells evident in cells obtained from the base of an ulcer or vesicular fluid.

For Recurrence:

3. Specific antiviral treatment initiated or recommended.

**64025**

Clinically apparent typical (vesicular or ulcerative) HSV lesion(s) with prodromal and/or concurrent symptoms of discomfort (burning, itching, pain) and response to treatment.

FSTRF maintains this code set on behalf of several clinical trials networks for which it is the data center.
Permission must be obtained through user.support@fstrf.org to use or copy it for any other purpose.

## HSV PNEUMONITIS

**64026 CONFIRMED**

1. Hypoxemia and infiltrates on chest X-ray or CT/MRI scan.

*and*

2. Tissue biopsy or cells obtained by BAL demonstrating HSV by antigen, detection of viral nucleic acids (e.g. PCR) or characteristic cytopathic changes.

*and*

3. No other pathogens identified by routine testing *or* signs/symptoms persist or recur after treatment of copathogens.

**64027 PROBABLE**

1. Hypoxemia and infiltrates on chest X-ray or CT/MRI scan.

*and*

2. Positive culture, detection of viral antigen, or detection of viral nucleic acids of HSV from fluid obtained by BAL.

*and*

3. No other pathogens identified by routine testing *or* signs/symptoms persist or recur after treatment of copathogens.

*and*

4. Specific antiviral treatment initiated or recommended.

## HERPES LABIALIS

**64028**

Clinical presentation of single or multiple vesicles or ulcers with crusting on vermillion portion of lips and adjacent facial skin. Usually mild to moderate pain. The lesion(s) is usually present for at most 10-14 days. There may be a prior history of (or recurrent) lesion(s).

## INTRA-ORAL HERPES SIMPLEX

**64029**

Clinical presentation of solitary, or cluster of multiple or confluent ulcers that may be noted together with vesicles on keratinized mucosa, including hard palate, attached gingival and dorsum of tongue. Exceptionally, non-keratinized tissue may be involved. Round to slightly irregular (map-like) margins with minimal to no erythematous halos are present. The base of the ulcers is usually pink. Usually mild to moderate pain. The lesion(s) is usually present for at most 10-14 days. There may be a prior history of lesion (or recurrent).

## MEASLES/RUBEOLA

**66500 CONFIRMED**

Documented by serology and/or virus detection.

**66501 PROBABLE**

NOT documented serologically or by virus detection.

FSTRF maintains this code set on behalf of several clinical trials networks for which it is the data center.
Permission must be obtained through user.support@fstrf.org to use or copy it for any other purpose.

## MUMPS/PAROTITIS

**66862 CONFIRMED**

Unilateral or bilateral swelling of the parotid gland with loss of the angle of the mandible, causative agent identified.

**66863 PROBABLE**

Unilateral or bilateral swelling of the parotid gland with loss of the angle of the mandible, no causative agent identified.

## ORAL HAIRY LEUKOPLAKIA

**64850 CONFIRMED**

Identification of Epstein-Barr virus (EBV) in the epithelial cells of an oral lesion by electron micrograph, in situ hybridization, or immunocytochemistry.

**64851 PROBABLE**

Presence of whitish/grey lesions on the lateral margins of the tongue that are otherwise asymptomatic. They are not removable and may exhibit vertical corrugations (classically described as hairy, shaggy or furry). The lesions range in size as they may be less than one centimeter, or may extend onto the ventral and dorsal surfaces of the tongue where they are usually flat. They may be bilateral or unilateral. The lesions are usually long-standing.

**64852**

Clinical diagnosis only, testing technology not available to determine diagnosis.

## ORAL WARTS

**64048**

Clinical presentation of warts that are mucosal color or white, solitary or multiple (often clustered) raised lesions that range in texture as they may be smooth, spiky, or cauliflower-like, and located in any part of the oral cavity. They are usually asymptomatic. The lesion(s) is usually long-standing.

Note: Warts on the buccal or labial mucosa or tongue may get traumatized by biting, and may be painful.

## PARVOVIRUS B19

**66690**

Infection; Fifth disease or aplastic crisis; documented serologically or by PCR.

FSTRF maintains this code set on behalf of several clinical trials networks for which it is the data center.
Permission must be obtained through user.support@fstrf.org to use or copy it for any other purpose.

## PROGRESSIVE MULTIFOCAL LEUKOENCEPHALOPATHY (PML)

**64040 CONFIRMED**

PML diagnosed by histopathology or in situ hybridization from a brain biopsy or by PCR of cerebrospinal fluid (CSF) for James Canyon Virus (JCV).

**64041 PROBABLE**

1. Clinical presentation compatible with PML including a subacute or chronic progressive illness with hemiparesis, aphasia, hemianopsia, ataxia and other focal deficits.

*and*

2. MRI compatible with PML.

**64042**

Clinical diagnosis only;

1. Clinical presentation consistent with PML including subacute onset of progressive focal neurological abnormalities, including hemiparesis or field cut or ataxia or other abnormality referable to dysfunction of a specific brain region. Does not include cognitive impairment alone.
2. Focal lesions without mass effect or enhancement on CT or MRI of brain.

## RUBELLA

**66760 CONGENITAL, CONFIRMED**, diagnosed clinically with viral detection or serology.

**66761 CONGENITAL, PROBABLE,** clinical diagnosis**.**

**66765 POSTNATAL**, documented serologically or by antigen detection or virus isolation.

## VARICELLA ZOSTER (VZV, Chickenpox)

**64033 VZV, UNCOMPLICATED**, primary disease (chickenpox), uncomplicated.

**64034 VZV, DISSEMINATED**, clinical chickenpox; disseminated disease, including VZV pneumonia, encephalitis, or hepatitis, (with hepatic enzymes greater than 30 times normal).

FSTRF maintains this code set on behalf of several clinical trials networks for which it is the data center.
Permission must be obtained through user.support@fstrf.org to use or copy it for any other purpose.

**CUTANEOUS VARICELLA ZOSTER (**VZV, Shingles, Herpes Zoster**)**

**64030 CONFIRMED**
**LOCALIZED**

1. Painful or dysesthetic lesions appearing in a dermatomal distribution. May be either typical (macular/papular progressing to vesiculopustular) or atypical (ulcerative, necrotic, nodular and/or verrucous).

*and*

2. Demonstration of VZV in lesions by DFA, culture or PCR.

**DISSEMINATED**

1. Demonstration of VZV in lesions by DFA, culture or PCR.

*and*

2. Lesions extend beyond the primary and its adjacent (flanking) dermatomes.

**64031 PROBABLE**

1. Painful or dysesthetic lesions appearing in a dermatomal distribution.

*and*

2. Specific antiviral treatment initiated or recommended.

**64032**

Clinical diagnosis with characteristic rash.

FSTRF maintains this code set on behalf of several clinical trials networks for which it is the data center.
Permission must be obtained through user.support@fstrf.org to use or copy it for any other purpose.

## VARICELLA ZOSTER (VZV, SHINGLES, HERPES ZOSTER) WITH VISCERAL DISSEMINATION

**64038 CONFIRMED**

1. Painful or dysesthetic lesions appearing in a dermatomal distribution. May be either typical (macular/papular progressing to vesiculopustular) or atypical (ulcerative, necrotic, and/or nodular).

<u>*and*</u>

2. Clinical findings, laboratory test abnormalities and/or radiographic (i.e. X-ray, ultrasonography, CT and/or MRI findings) consistent with the diagnosis.

Examples:

   a. Pulmonary
      i. Bilateral interstitial infiltrates on chest X-ray and
      ii. Clinical signs and symptoms of pulmonary disease during the course of infection
   b. Hepatitis: Significant elevations of bilirubin, AST, ALT, attributable to VZV
   c. CNS: Encephalopathy and CSF pleocytosis with negative bacterial, acid-fast, fungal and viral cultures (other than HZV)
   d. Myelitis/Paralysis: pain in back or legs, with or without urinary retention, hyperesthesia and motor disturbances or paralysis. Loss or impairment of motor function involves area(s) not within the dermatomal distribution of the study participant's localized zoster.

<u>*and*</u>

3. Demonstration of VZV in cutaneous or visceral lesions by DFA, culture or PCR.

**64039 PROBABLE**

1. Painful or dysesthetic lesions appearing in a dermatomal distribution. May be either typical (macular/papular progressing to vesiculopustular) or atypical (ulcerative, necrotic, and/or nodular).

<u>*and*</u>

2. Clinical findings, laboratory test abnormalities and/or radiographic (i.e. X-ray, ultrasonography, CT and/or MRI findings) consistent with the diagnosis.

Examples:

   a. Pulmonary
      i. Bilateral interstitial infiltrates on chest X-ray and
      ii. Clinical signs and symptoms of pulmonary disease during the course of infection
   b. Hepatitis: Significant elevations of bilirubin, AST, ALT, attributable to VZV
   c. CNS: Encephalopathy and CSF pleocytosis with negative bacterial, acid-fast, fungal and viral cultures (other than HZV)
   d. Myelitis/Paralysis: pain in back or legs, with or without urinary retention, hyperesthesia and motor disturbances or paralysis. Loss or impairment of motor function involves area(s) not within the dermatomal distribution of the study participant's localized zoster.

FSTRF maintains this code set on behalf of several clinical trials networks for which it is the data center.
Permission must be obtained through user.support@fstrf.org to use or copy it for any other purpose.

# V. NEOPLASTIC DISEASES

## ADENOCARCINOMA, ENDOCERVICAL

**67045 CONFIRMED**
Diagnostic histopathology on biopsy or surgical pathology results showing endocervical adenocarcinoma.

**67030 PROBABLE**
Diagnostic cytology or PAP smear results showing endocervical adenocarcinoma.

## ADENOCARCINOMA, ENDOMETRIAL

**67046 CONFIRMED**
Diagnostic histopathology on biopsy or surgical pathology results showing endometrial adenocarcinoma.

**67031 PROBABLE**
Diagnostic cytology or PAP smear results showing endometrial adenocarcinoma.

## ADENOCARCINOMA, EXTRAUTERINE

**67047 CONFIRMED**
Diagnostic histopathology on biopsy or surgical pathology results showing extrauterine adenocarcinoma.

**67032 PROBABLE**
Diagnostic cytology or PAP smear results showing extrauterine adenocarcinoma.

## ADENOCARCINOMA, OTHER SITE

**67048 CONFIRMED**
Diagnostic histopathology on biopsy or surgical pathology results showing adenocarcinoma, specify site.

**67091 PROBABLE**
Diagnostic cytology or PAP smear results showing adenocarcinoma, specify site.

## ATROPHY, CERVICAL OR VAGINAL

**67036 CONFIRMED**
Diagnostic histopathology on biopsy or surgical pathology results showing reactive cellular changes associated with atrophy with inflammation ("atrophic vaginitis").

**67033 PROBABLE**
Diagnostic cytology or PAP smear results showing reactive cellular changes associated with atrophy with inflammation ("atrophic vaginitis").

FSTRF maintains this code set on behalf of several clinical trials networks for which it is the data center.
Permission must be obtained through user.support@fstrf.org to use or copy it for any other purpose.

## ATYPIA, GLANDULAR CELL (AGCUS), CERVICAL

**67034 CONFIRMED**
Diagnostic cytology or PAP smear results showing atypical glandular cells of uncertain significance without intraepithelial neoplasia.

## ATYPIA, SQUAMOUS CELL (ASCUS), CERVICAL

**67035 CONFIRMED**
Diagnostic cytology or PAP smear results showing atypical squamous cells of uncertain significance without intraepithelial neoplasia.

## INVASIVE CERVICAL CARCINOMA

**66025 CONFIRMED**
An abnormal cervical histopathology (biopsy) specimen.

**PROBABLE**
There is no acceptable definition to be used within the ACTG.

## CARCINOMA IN SITU

**67044 CONFIRMED** (Specify site: cervical, vaginal, vulvar, perianal)
Diagnostic histopathology on biopsy or surgical pathology results showing high-grade squamous intraepithelial lesion (HGSIL), carcinoma in situ (CIS).

**67080 PROBABLE** (Specify site: cervical, vaginal, vulvar, perianal)
Diagnostic cytology or PAP smear results showing high-grade squamous intraepithelial lesion (HGSIL), carcinoma in situ (CIS).

## CERVICAL INFLAMMATION

**67093 CONFIRMED**
Diagnostic histopathology on biopsy or surgical pathology results showing reactive cellular changes associated with inflammation (including typical repair).

**67082 PROBABLE**
Diagnostic cytology or PAP smear results showing reactive cellular changes associated with inflammation (including typical repair).

FSTRF maintains this code set on behalf of several clinical trials networks for which it is the data center.
Permission must be obtained through user.support@fstrf.org to use or copy it for any other purpose.

## RADIATION CELL CHANGES

**67058 CONFIRMED, SPECIFY SITE**
Diagnostic histopathology on biopsy or surgical pathology results showing reactive cellular change associated with radiation changes.

**67094 PROBABLE, SPECIFY SITE**
Diagnostic cytology or PAP smear results showing reactive cellular change associated with radiation changes.

## DYSPLASIA

**67095 CONFIRMED** (Specify site: cervical, vaginal, vulvar, perianal)
Diagnostic histopathology on biopsy or surgical pathology (grade unknown).

**67083 PROBABLE** (Specify site: cervical, vaginal, vulvar, perianal)
Diagnostic cytology or PAP smear (grade unknown).

## DYSPLASIA/INTRAEPITHELIAL NEOPLASIA, LOW GRADE/GRADE 1

**67096 CONFIRMED** (Specify site: cervical, vaginal, vulvar, perianal)
Diagnostic histopathology on biopsy or surgical pathology results showing low grade squamous intraepithelial neoplasia or dysplasia, specify type.

**67084 PROBABLE** (Specify site: cervical, vaginal, vulvar, perianal)
Diagnostic cytology or PAP smear results showing low grade squamous intraepithelial lesion (LGSIL).

## DYSPLASIA/INTRAEPITHELIAL NEOPLASIA, MODERATE/GRADE 2

**67097 CONFIRMED** (Specify site: cervical, vaginal, vulvar, perianal)
Diagnostic histopathology on biopsy or surgical pathology results showing moderate squamous intraepithelial dysplasia or neoplasia, specify type.

**67085 PROBABLE** (Specify site: cervical, vaginal, vulvar, perianal)
Diagnostic cytology or PAP smear results showing high grade squamous intraepithelial lesion (HGSIL), moderate dysplasia.

## DYSPLASIA/INTRAEPITHELIAL NEOPLASIA, SEVERE/GRADE 3

**67098 CONFIRMED** (Specify site: cervical, vaginal, vulvar, perianal)
Diagnostic histopathology on biopsy or surgical pathology results showing severe squamous intraepithelial dysplasia or neoplasia.

**67086 PROBABLE** (Specify site: cervical, vaginal, vulvar, perianal)
Diagnostic cytology or PAP smear results showing high grade squamous intraepithelial lesion (HGSIL), severe dysplasia.

FSTRF maintains this code set on behalf of several clinical trials networks for which it is the data center.
Permission must be obtained through user.support@fstrf.org to use or copy it for any other purpose.

## DYSPLASIA/INTRAEPITHELIAL NEOPLASIA/CANCER, VAGINAL

**67042 CONFIRMED**
Diagnostic histopathology on biopsy or surgical pathology, specify type.

**67087 PROBABLE**
Diagnostic cytology or PAP smear, specify type.

## DYSPLASIA/INTRAEPITHELIAL NEOPLASIA/CANCER, VULVAR

**67043 CONFIRMED**
Diagnostic histopathology on biopsy or surgical pathology, specify type.

**67088 PROBABLE**
Diagnostic cytology or PAP smear, specify type.

## IUD REACTIVE CHANGES

**67040 CONFIRMED**
Diagnostic histopathology on biopsy or surgical pathology results showing reactive cellular changes associated with intrauterine contraceptive device (IUD).

**67089 PROBABLE**
Diagnostic cytology or PAP smear results showing reactive cellular changes associated with intrauterine contraceptive device (IUD).

## KAPOSI SARCOMA (KS) MUCOCUTANEOUS AND VISCERAL

**66011 CONFIRMED MUCOCUTANEOUS,** specify site
**66012 CONFIRMED VISCERAL,** specify site
Positive histopathology on tissue from any site/organ.

**66013 PROBABLE MUCOCUTANEOUS,** specify site
**66014 PROBABLE VISCERAL,** specify site
Characteristic lesion(s) on skin or mucous membrane noted by an experienced physician. The early lesions are typically flat (or macular) with color ranging from red to purple. At a later stage, lesions become nodular, raised and ulcerated. On the oral mucosa, the lesions are predominantly seen on the palate or gingival. At the early stage, the lesions are asymptomatic. Mild to moderate pain may develop as the lesions become nodular and ulcerated. Local trauma to the more advanced lesions may induce bleeding. The nodular lesions are long-standing.

FSTRF maintains this code set on behalf of several clinical trials networks for which it is the data center.
Permission must be obtained through user.support@fstrf.org to use or copy it for any other purpose.

## PRIMARY CNS LYMPHOMA (PCL)

**66020 CONFIRMED**

Positive histopathology/cytology on tissue biopsy of brain or cerebrospinal fluid analysis.

**66021 PROBABLE**

1. Neurologic signs with CD4 lymphocyte count <100/$mm^3$

*and*

2. Mass lesion(s) on head CT/MRI scan.

*and*

3. Failure of clinical response to antitoxoplasmosis chemotherapy or other anti-infective chemotherapy (e.g. tuberculosis, cryptococcosis).

*and*

4. Lesion(s) becomes markedly reduced or disappears following high-dose glucocorticoid and/or radiation therapy.

**66022**

Clinical diagnosis only, testing technology not available to determine diagnosis.

## NON-HODGKIN LYMPHOMA (NHL)

**CONFIRMED**
**66031 LYMPHOMA, N-H SMALL NON-CLEAVED (BURKITT OR BURKITT'S LIKE)**
**66032 LYMPHOMA, N-H IMMUNOBLASTIC**
**66033 LYMPHOMA, N-H LARGE CELL**
**66034 N-H INDETERMINATE**

Including all B cell or indeterminate cell, intermediate to high-grade malignant lymphomas (e.g. large cell, immunoblastic, small non-cleaved, Burkitt or Burkitt's-like lymphoma.) Pathological/biopsy confirmation of NHL is mandatory in all cases.

Positive histopathology/cytology/fine-needle aspiration on tissue biopsy from any site/organ, supported by appropriate immunocytochemical or molecular biological investigations. (Note: bone marrow sampling may confirm diagnosis despite non-diagnostic biopsies from other sites.)

Oral manifestations present as a firm elastic, often somewhat reddish or purplish swelling, with or without ulceration. The gingival, palatal mucosa, and fauces are sites of predilection.
Note: The fauces are regarded as the two pillars of mucous membrane, the palatoglossal arch on the anterior and the palatopharyngeal arch on the posterior, surrounding the palatine tonsils. At the early stage, the lesions are usually asymptomatic. Moderate to severe pain may develop as the lesions become ulcerated. The ulcerated lesions and swelling are long-standing.

**PROBABLE**

There is no acceptable definition to be used within the ACTG. Pathological/biopsy confirmation of NHL is mandatory in all cases.

FSTRF maintains this code set on behalf of several clinical trials networks for which it is the data center.
Permission must be obtained through user.support@fstrf.org to use or copy it for any other purpose.

## PREINVASIVE ANOGENITAL NEOPLASIA, SPECIFY SITE

**66026 CONFIRMED**

1. Characteristic lesion(s) evident on gross examination by cervical or anal microscopy of the vulva, vagina, cervix, perineum, perianal or anal area *or* cytopathology (PAP smear, cervical or anal) showing dysplastic cells.

*and*

2. Histopathologic (biopsy) examination confirming the presence of dysplasia

**66027 PROBABLE**

1. Characteristic lesion(s) evident on gross examination by cervical or anal microscopy of the vulva, vagina, cervix, perineum, perianal or anal area *or* cytopathology (PAP smear, cervical or anal) showing dysplastic cells.

*and*

2. The presence of human papilloma virus (HPV) confirmed by hybrid capture, polymerase chain reaction (PCR) or other testing methods for HPV.

**66028**

Clinical diagnosis only, testing technology not available to determine diagnosis. Specify site.

## NEOPLASM, GYNECOLOGICAL

**67049 CONFIRMED**

Diagnostic histopathology on biopsy or surgical pathology results showing other malignant neoplasms (specify neoplasm).

**67090 PROBABLE**

Diagnostic cytology or PAP smear results showing other malignant neoplasms (specify neoplasm).

## POLYP

**67057 PROBABLE, SPECIFY SITE**

Origin uncertain, diagnostic cytology or PAP smear.

## POLYP, CERVICAL

**67055 CONFIRMED**

Diagnostic histopathology on biopsy or surgical pathology.

## POLYP, ENDOMETRIAL

**67056 CONFIRMED**

Diagnostic histopathology on biopsy or surgical pathology.

## MALIGNANCY

**68649**

Any newly diagnosed malignancy, except squamous cell cancer of the skin, specify diagnosis

FSTRF maintains this code set on behalf of several clinical trials networks for which it is the data center.
Permission must be obtained through user.support@fstrf.org to use or copy it for any other purpose.

## CERVICAL/ANOGENITAL SQUAMOUS CELL CANCER, INVASIVE

**67092 CONFIRMED**

Diagnostic histopathology on biopsy or surgical pathology. Specify site.

**67081 PROBABLE**

Diagnostic cytology or PAP smear. Specify site.
Any newly diagnosed malignancy, except squamous cell cancer of the skin, specify diagnosis

## ORAL SQUAMOUS CELL CARCINOMA

**66015 CONFIRMED**

1. Characteristic histological appearance on biopsy.

**66016 PROBABLE**

1. Clinical presentation of non-healing ulcer with rolled borders or margins. An advanced stage ulcer may be indurated or located on a firm mass. At the early stage, the lesions are usually asymptomatic. Moderate to severe pain may develop as lesions enlarge and become ulcerated. The ulcerated lesions and swellings are long-standing.

FSTRF maintains this code set on behalf of several clinical trials networks for which it is the data center.
Permission must be obtained through user.support@fstrf.org to use or copy it for any other purpose.

# VI. PERINATAL/GYNECOLOGIC CONDITIONS

---

**THERAPEUTIC ABORTION, (ELECTIVE/INDUCED)**

**68138**
Termination of pregnancy prior to viability utilizing a medical or surgical procedure.

---

**SPONTANEOUS ABORTION/MISCARRIAGE**

**68137**
Loss of a pregnancy at < 20 weeks gestation either spontaneously or through medical or surgical procedure after documentation of no fetal heart activity.

---

**VAGINAL BLEEDING**

**68050 VAGINAL BLEEDING <28 WEEKS**
Any vaginal bleeding occurring during pregnancy prior to 28 weeks gestation and prior to the onset of labor.

**68052 VAGINAL BLEEDING ≥ 28 WEEKS**
Any vaginal bleeding occurring during pregnancy at or after 28 weeks gestation and prior to the onset of labor.

---

**CHORIOAMNIONITIS/AMNIOTIC FLUID INFECTION**

**61085 CONFIRMED**
Amniotic fluid with a positive gram stain or culture.

**61086 PROBABLE**

1. Clinical diagnosis by obstetrician alone

*or*

2. Maternal oral temperature greater than or equal to 100.4º F or 38 º C not attributable to other causes

*and* any two of the following:

a. Fetal heart rate which is persistently >160BPM
b. Maternal heart rate which is >120BPM in the absence of tocolytics or known maternal heart tachyarrhythmia
c. Uterine tenderness not associated with contractions
d. Purulent cervical discharge or amniotic fluid
e. Premature labor unresponsive to tocolytic therapy

---

**CORD PROLAPSE**

**68020**
Documentation of protrusion of the umbilical cord through the cervical os.

FSTRF maintains this code set on behalf of several clinical trials networks for which it is the data center.
Permission must be obtained through user.support@fstrf.org to use or copy it for any other purpose.

## GESTATIONAL DIABETES (MEDICATION DEPENDENT)

**68030**

1. Abnormal three-hour glucose tolerance test during pregnancy, specify gestational age at diagnosis. Criteria: two abnormal serum values from the following:
   a. Fasting > 95; 1 hour > 180; 2 hour > 155; 3 hour >140

*or*

   b. Abnormal 1 hour post 50 gram glucose load of >200 mg/dL

*or*

   c. 2 abnormal fasting blood sugars according to institutional standards, specify values

*or*

   d. Gestational diabetes diagnosed by another method, specify method of diagnosis

*and*

2. Hyperglycemia requiring the administration of insulin or oral agent and diabetic diet

## GESTATIONAL DIABETES (DIET)

**68032**

1. Abnormal three-hour glucose tolerance test during pregnancy, specify gestational age at diagnosis. Criteria: two abnormal serum values from the following:
   a. Fasting > 95; 1 hour > 180; 2 hour > 155; 3 hour > 140

*or*

   b. Abnormal 1 hour post 50 gram glucose load of >200 mg/dL

*or*

   c. 2 abnormal fasting blood sugars according to institutional standards, specify values

*or*

   d. Gestational diabetes diagnosed by another method, specify method of diagnosis

*and*

2. Control of hyperglycemia with diabetic diet alone and no history of elevated blood sugar prior to pregnancy.

## ECLAMPSIA

**68076**

1. Seizure during pregnancy in the absence of any underlying known etiology or without any known reason for seizure.

*and*

2. No suspicion of epilepsy or trauma.

## ENDOMETRITIS

**61172 CONFIRMED**

Etiology proven by positive test for specific organism in endocervical secretions or positive endometrial culture.

**61173 PROBABLE**

Diagnosed clinically, etiology unproven, maternal postpartum fever greater than or equal to 38° C not attributable to other causes and accompanied by uterine tenderness.

FSTRF maintains this code set on behalf of several clinical trials networks for which it is the data center.
Permission must be obtained through user.support@fstrf.org to use or copy it for any other purpose.

## EPISIOTOMY INFECTION, MAJOR

**68172**

1. Oral temperature ≥100.4˚F or 38˚C in the absence of other sources of fever

*and*

2. Pus draining/drained from wound *or* wound dehiscence (episiotomy breakdown) requiring debridement.

## EPISIOTOMY INFECTION, MINOR

**68173**

Erythema, edema and tenderness *or* health care provider diagnosis.

## FEBRILE MORBIDITY

**68039 INTRAPARTUM**

Oral, aural/tympanic or forehead temperature ≥100.4º F or 38º C or rectal temperature ≥100º F Or 38.3º C.

**68040 POSTPARTUM**

Oral, aural/tympanic or forehead temperature ≥100.4º F or 38º C on any two occasions 4 hours apart from >24 hours post delivery through 10 days postpartum.

## HELLP SYNDROME (HEMOLYSIS, ELEVATED LIVER ENZYMES, LOW PLATELETS)

**68045**

This diagnosis should be reviewed by an obstetrician for confirmation before being reported.

1. The diagnosis must be made after 20 weeks gestation.

*and*

2.
   a. For women with no hypertension or proteinuria before 20 weeks gestation: Pregnancy associated hypertension consisting of a diastolic blood pressure of 90 mmHg or greater on two occasions, 4 hours to 1 week (or 168 hours) apart.

   b. For women with hypertension but no proteinuria before 20 weeks gestation: no hypertension requirement.

   c. For women with proteinuria but no hypertension before 20 weeks gestation: no hypertension requirement.

*and*

3. All of the following:
   a. Thrombocytopenia: at least one platelet count <100,000 per cubic millimeter ($mm^3$)
   b. AST/SGOT ≥ 70 U per liter (U/L).
   c. Hemolysis: LDH ≥600 U per liter (U/L) *or* total bilirubin concentration ≥ 2 mg per deciliter (mg/dL) *or* a peripheral smear with nucleated RBCs or schistocytes.

## HEMATOMA, VAGINAL OR VULVAR

**68060**
Documentation of a collection of blood, specify site.

## HEMORRHAGE, INTRAPARTUM

**68054 HEMORRHAGE WITH HEMODYNAMIC INSTABILITY INTRAPARTUM**
1. Bleeding

*and*
2. Blood pressure <90/60

*or*
3. Maternal heart rate>120 BPM.

*and*
4. Includes only those episodes treated with fluid/volume expanders.

**68056 HEMORRHAGE REQUIRING SURGICAL PROCEDURE INTRAPARTUM**
Bleeding that necessitates surgical intervention, such as dilation and curettage, hysterectomy or uterine artery ligation or embolizaton

**68058 HEMORRHAGE REQUIRING TRANSFUSION, INTRAPARTUM**
Bleeding with estimated maternal blood loss of > 750 mL in vaginal delivery or >1200 mL in caesarean delivery that necessitates transfusion intrapartum

## HEMORRHAGE, POSTPARTUM

**68055 HEMORRHAGE WITH HEMODYNAMIC INSTABILITY POSTPARTUM**
1. Postpartum maternal hemorrhage with estimated maternal blood loss of >750 mL in vaginal delivery or >1200 mL in caesarean delivery,

*and*
2. Hemodynamic instability

*and*
3. BP < 90/60 *or* HR >120 BPM

*and*
4. Treated with fluid/volume expanders.

**68057 HEMORRHAGE REQUIRING SURGICAL PROCEDURE POSTPARTUM**
Bleeding with estimated maternal blood loss of >750 mL in vaginal delivery or >1200 mL in caesarean delivery, which requires additional surgery such as dilation and curettage, hysterectomy or uterine artery ligation or embolization to control bleeding. Examples include retained placenta requiring curettage, placenta accreta requiring hysterectomy, and vaginal lacerations requiring repair in an operating room.

**68059 HEMORRHAGE REQUIRING TRANSFUSION, POSTPARTUM**
Bleeding with estimated maternal blood loss of >750 mL in vaginal delivery or >1200 mL in caesarean delivery that necessitates transfusion to maintain hemodynamic stability as defined by one of the following: to correct BP<90/60 or HR >120 BPM; or to maintain hematocrit >20.

## HYPERTENSION, CHRONIC, IN PREGNANCY

**68072**

Blood pressure persistently ≥140/90 mm Hg that began prior to pregnancy or in the first 20 weeks of pregnancy *or* study participant is on anti-hypertension medication at the onset of pregnancy.

## HYPERTENSION, PREGNANCY-INDUCED

**68070**

Blood pressure persistently ≥ 140/90mm Hg WITHOUT proteinuria and onset after first 20 weeks gestation with no hypertension prior to pregnancy.

## INCOMPETENT CERVIX

**68082**

History consistent with incompetent cervix or current exam by physical diagnosis or imaging study as determined by obstetrician.

## INCOMPETENT CERVIX, PROPHYLACTIC CERCLAGE

**68080**

History consistent with incompetent cervix, resulting in prophylactic cerclage placement.

## INCOMPETENT CERVIX, EMERGENCY CERCLAGE

**68081**

History consistent with incompetent cervix, resulting in emergency cerclage placement.

## INTRAUTERINE FETAL DEMISE

**68182**

Intrauterine death at ≥20 weeks gestational age.  Specify gestational age at diagnosis of general death.

## INTRAUTERINE GROWTH RESTRICTION (IUGR) FETAL

**68090**

Based on ultrasound with estimated fetal weight ≤ 10th percentile for gestational age.

## INTRAUTERINE GROWTH RESTRICTION (IUGR) FETAL, SEVERE

**68091**

Based on ultrasound with estimated fetal weight ≤ 3rd percentile for gestational age.

FSTRF maintains this code set on behalf of several clinical trials networks for which it is the data center.
Permission must be obtained through user.support@fstrf.org to use or copy it for any other purpose.

## PREMATURE LABOR

**68140**

Uterine contractions after 20 weeks and before 37 weeks necessitating tocolytic therapy and/or resulting in delivery.

## MASTITIS

**68100 CONFIRMED**

In postpartum study participant.

1. Oral temperature >100.4° Fahrenheit or 38° Celsius.

*and*

2. Any two of the following:
   a. Unilateral breast (not nipple) pain
   b. Erythema and induration in one area of the breast
   c. Fluctuation of one area of the breast

**68101**

Clinical diagnosis only by the health care provider.

## OLIGOHYDRAMNIOS

**68110**

Amniotic fluid index (AFI) less than 5 cm or largest vertical pocket <2 cm *or* diagnosis by ultrasound without AFI information.

## ABRUPTIO PLACENTA

**68010**

Examination of the placenta at delivery reveals retroplacental clot *or* clinical diagnosis in study participant with two of the following: vaginal bleeding; uterine tenderness without other evidence of chorioamnionitis; hypercontractility and/or hypertonus.

## PLACENTA ACCRETA (TOTAL OR PARTIAL)

**68121**

Placental villi invasion of the myometrium at the site of implantation and leading to obliteration of the nomal cleavage plane.

## PLACENTA INCRETA

**68123**

Abnormal placental implantation with the villi extending into the myometrium.

## PLACENTA PERCRETA

**68122**

Invasion of villi through the full thickness of the myometrium.

FSTRF maintains this code set on behalf of several clinical trials networks for which it is the data center.
Permission must be obtained through user.support@fstrf.org to use or copy it for any other purpose.

## PLACENTA PREVIA

**68120**

Documentation that the placenta overlies the cervical os by one of the following: by ultrasound; at delivery or at time of caesarean section.

## POLYHYDRAMNIOS

**68130**

Amniotic Fluid Index (AFI) ≥25 cm or maximum vertical pocket > 8 cm *or* diagnosis by ultrasound without AFI information.

## PRE-ECLAMPSIA

**68074**

1. Must occur after 20 weeks of gestation:

*and*

2. Blood pressure persistently ≥140/90 mm Hg

and at least one of the following:

a. Proteinuria of ≥ 1+ by dipstick, on two occasions

*and/or*

b. ≥300mg protein in 24 hour collection

## PREGNANCY

**68135 ECTOPIC PREGNANCY**

Implantation of the fertilized ovum outside the uterine cavity.

**68136 INTRAUTERINE PREGNANCY**

## PREGNANCY, POSTDATES/POST-TERM

**68153**

Pregnancy at ≥ 42 weeks gestation.

## PREMATURE RUPTURE OF MEMBRANES, PRETERM, CONFIRMED

**68150**

Spontaneous rupture of membranes <37 weeks. Must be documented by one of the following:

a. Visualizing a pool of amniotic fluid in the vagina; or
b. Gross leakage of amniotic fluid from the vagina;
c. Positive peri-pad test after installation of indigo carmine dye;
d. Elevated pH;
e. Ferning of dried fluid on a microscope slide;
f. History consistent with premature rupture of membranes or
g. Decreased amniotic fluid volume on ultrasound with no other explanation for the oligohydramnios.

FSTRF maintains this code set on behalf of several clinical trials networks for which it is the data center.
Permission must be obtained through user.support@fstrf.org to use or copy it for any other purpose.

## PREMATURE RUPTURE OF MEMBRANES, PROBABLE

**68151**

Suspected but not confirmed.

## PRETERM DELIVERY

**68152**

Delivery before 37 completed weeks gestation.

## UTERINE ATONY

**68185**

Failure of the uterus to contract postpartum, requiring intervention.

## UTERINE INVERSION

**68186**

Clinical diagnosis by obstetrical provider.

## UTERINE RUPTURE

**68184**

Spontaneous rupture of pregnant uterus resulting in fetal distress, maternal hemorrhage, or extrusion of all or part of the fetus. Does not include asymptomatic uterine dehiscence.

## UTERINE SCAR DEHISCENCE

**68196**

Separation of scar from prior uterine surgery without meeting any of the criteria for uterine rupture, asymptomatic.

## ABDOMINAL WOUND INFECTION, MAJOR (CAESAREAN)

**68170**

1. Oral temperature ≥100.4° F or 38° C in the absence of other source of fever

*and*

2. Pus draining/drained from wound *or* wound dehiscence requiring debridement.

## ABDOMINAL WOUND INFECTION, MINOR (CAESAREAN)

**68171**

Erythema, edema and tenderness *or* health care provider diagnosis.

FSTRF maintains this code set on behalf of several clinical trials networks for which it is the data center.
Permission must be obtained through user.support@fstrf.org to use or copy it for any other purpose.

# VII. NEONATAL DISORDERS (Infants)

**HEMOLYTIC DISEASE OF THE NEWBORN**

**67248 ABO HEMOLYTIC DISEASE OF THE NEWBORN**
**67249 RH HEMOLYTIC DISEASE OF THE NEWBORN**

Mediated by maternal antibody, Coomb's positive.

**NEONATAL HEPATITIS**

**61008**
Characterized by elevated transaminases 1.5 times the upper limit of normal with or without clinical findings such as jaundice, hepatomegaly, and hepatic failure.

**INTRAVENTRICULAR HEMORRHAGE, GRADE 3, NEONATAL**

**68190**
Radiologic diagnosis of hemorrhage into the germinal matrix tissues of the developing brain with possible rupture into the ventricular system and parenchyma.

**INTRAVENTRICULAR HEMORRHAGE, GRADE 4, NEONATAL**

**68189**
Radiologic diagnosis of hemorrhage into the germinal matrix tissues of the developing brain with possible rupture into the ventricular system and parenchyma.

**KERNICTERUS**

**61010**
Clinical neonatal syndrome in the presence of severe indirect hyperbilirubinemia (>20mg/dL) associated with CNS symptoms, such as lethargy, hypotonia, irritability, poor Moro response, and poor feeding. Clinical findings include bulging fontanel, opisthotonic posturing, pulmonary hemorrhage, fever, hypertonicity, paralysis, and/or seizures.

**MECONIUM ASPIRATION SYNDROME**

**68192**
Aspiration of meconium mixed with amniotic fluid in utero or during delivery causing a partial or complete blockage of the airways associated with poor gas exchange in the lungs and chemical pneumonitis.

FSTRF maintains this code set on behalf of several clinical trials networks for which it is the data center.
Permission must be obtained through user.support@fstrf.org to use or copy it for any other purpose.

## NECROTIZING ENTEROCOLITIS, NEONATAL

**68193 CONFIRMED**

Inflammation causing destruction of part of the bowel, may involve only the innermost lining or the entire thickness of the bowel, variable amounts of the bowel, proven by either surgery or radiographic study.

**68194 PROBABLE**

Inflammation causing destruction of part of the bowel, may involve only the innermost lining or the entire thickness of the bowel, variable amounts of the bowel: radiographic study non-diagnostic.

## RESPIRATORY DISTRESS SYNDROME, NEWBORN

**68197**

Clinical presentation of respiratory distress in a premature infant due to surfactant deficiency.

## NEONATAL SEPSIS

**61009**

Clinical and/or laboratory findings indicating the presence of disseminated bacterial infection in the infant (0-6 weeks of age).

## CONGENITAL SYPHILIS, EARLY

**61500 CONFIRMED**

< 1 year old, characteristic symptoms; demonstration of T. pallidum in specimens from infant or stillbirth.

**61501 PROBABLE**

< 1 year old, based on maternal history, infant or maternal serologic findings, and clinical presentation of infant; organism not detected.

## CONGENITAL SYPHILIS, LATE, SYMPTOMATIC

**61510**

Aged > 1 year old; seropositive with clinical evidence of late sequelae of congenital syphilis.

## CONGENITAL SYPHILIS, LATE, ASYMPTOMATIC

**61511**

Asymptomatic, aged > 1 year old; seropositive without clinical evidence of late sequelae of congenital syphilis.

FSTRF maintains this code set on behalf of several clinical trials networks for which it is the data center.
Permission must be obtained through user.support@fstrf.org to use or copy it for any other purpose.

## TORCH SYNDROME

**63100**

Clinical and/or laboratory findings in the neonate indicating one of the following congenital infections: toxoplasmosis, rubella, cytomegalovirus (CMV), or congenital herpes simplex infection (HSV).

## TOXOPLASMOSIS, CONGENITAL

**65030 CONFIRMED, SYMPTOMATIC**

Diagnosed by IgM/IgA serology or histopathology within 1st month of life, with clinical evidence of disease by CT scan, ophthalmologic exam, or physical exam.

**65031 PROBABLE, SYMPTOMATIC**

Diagnosed by IgM/IgA serology or histopathology at 1-5 months of life, with clinical evidence of disease by CT scan, ophthalmologic exam, or physical exam.

**65040 CONFIRMED, ASYMPTOMATIC**

Diagnosed by IgM serology or histopathology within 1st month of life, with no symptoms or signs.

**65041 PROBABLE, ASYMPTOMATIC**

Diagnosed by IgM serology or histopathology within 1-5 months of life, with no symptoms or signs.

## TRANSIENT TACHYPNEA, NEWBORN

**68198**

Noninfectious acute respiratory disease in newborn infants which results in admission to a critical care unit. TTN is the result of a delay in clearance of fetal lung liquid. Signs of respiratory distress (e.g., tachypnea, nasal flaring, grunting, retractions, cyanosis in extreme cases) become evident shortly after birth. The disorder is indeed transient, with resolution occurring usually by age 72 hours.

FSTRF maintains this code set on behalf of several clinical trials networks for which it is the data center.
Permission must be obtained through user.support@fstrf.org to use or copy it for any other purpose.

# VIII. BIRTH DEFECTS

**69233 ANOMALIES OF THE EAR,** congenital, specify anomaly.

**69232 ANOMALIES OF THE EYE,** congenital, specify anomaly.

**69234 ANOMALIES OF THE NOSE,** congenital, specify anomaly.

**69204 CLEFT LIP**
**CLEFT PALATE**
(If the study participant has both a cleft lip and a cleft palate, report each of these as a separate diagnosis using the same diagnosis code.)

**69205 CNS ANATOMICAL DEFECT,** other, specify.

**69228 CUTANEOUS DEFECTS,** specify (e.g., skin dimples, brachial cleft and thyroglossal, supernumery nipples).

**69226 DIAPHRAGMATIC HERNIA,** hemidiaphragm/absence of diaphragm, congenital**.**

**69202 DOWN SYNDROME,** Trisomy 21.

**69208 ENDOCRINE BIRTH DEFECT,** other, specify.

**69209 FETAL ALCOHOL SYNDROME**

**69210 GASTROINTESTINAL,** anatomical defect, specify

**69212 GENITOURINARY, MALE**, anatomical defect, specify**.**

**69213 GENITOURINARY, FEMALE,** anatomical defect, specify**.**

**69214 GENITOURINARY DEFECT,** other, specify.

**69222 GLYCOGEN STORAGE DISEASE,** congenital, specify.

**69200 HEART DEFECTS,** (anatomical), specify.

**69207 INFANT OF DIABETIC MOTHER**

**69221 INBORN ERRORS OF METABOLISM**

**69223 MUSCULOSKELETAL ABNORMALITY,** congenital, specify**.**

FSTRF maintains this code set on behalf of several clinical trials networks for which it is the data center.
Permission must be obtained through user.support@fstrf.org to use or copy it for any other purpose.

**69225 MUSCULOSKELETAL, ABSENCE OF,** congenital, specify**.**

**69224 MUSCULOSKELETAL DUPLICATION,** specify.

**69206 NEURAL TUBE DEFECT**, includes Spina Bifida, specify defect.

**69230 PIGMENT DISORDERS** (e.g., albinism, café au lait spots), specify size and location.

**69211 PYLORIC STENOSIS,** congenital, proven.

**69227 RESPIRATORY BIRTH DEFECT,** other, specify.

**69231 SKIN BIRTH DEFECT,** other, specify.

**69201 TRISOMIES-TRISOMY,** specify.

**69203 TURNER SYNDROME**

**69229 VASCULAR LESIONS** (e.g., port wine, nevi and hemangiomas).

**69239 OTHER BIRTH DEFECT,** specify.

FSTRF maintains this code set on behalf of several clinical trials networks for which it is the data center.
Permission must be obtained through user.support@fstrf.org to use or copy it for any other purpose.

# IX. MITOCHONDRIAL DISORDERS

Mitochondrial diseases result from failures of the mitochondria (specialized compartments present in every cell of the body except red blood cells), which are responsible for creating more than 90% of the energy needed by the body to sustain life and support growth. When they fail, less and less energy is generated within the cell. Cell injury and even cell death follow. If this process is repeated throughout the body, whole systems begin to fail, and the life of the person in whom this is happening is severely compromised. These inherited diseases primarily affect children but adult onset may occur.

Diseases of the mitochondria appear to cause the most damage to cells of the brain, heart, liver, skeletal muscles, kidney and the endocrine and respiratory systems.

Depending on which cells are affected, symptoms may include loss of motor control, muscle weakness and pain, gastro-intestinal disorders and swallowing difficulties, poor growth, cardiac disease, liver disease, diabetes, respiratory complications, seizures, visual/hearing problems, lactic acidosis, developmental delays and susceptibility to infection.

Please note that great care is required when applying one of the following diagnoses due to the overlapping of symptoms associated with this particular class of diseases.

---

**ALPER'S DISEASE**

**69300**

Progressive neurodegenerative disease of the brain characterized by developmental delay, progressive mental retardation, hypotonia, spasticity and dementia, seizures often intractable, including epilepsia partialis continua, optic atrophy, and chronic liver dysfunction leading to liver failure.

---

**CYCLIC VOMITING SYNDROME**

**69301**

Childhood disorder; bouts of vomiting that last from a few hours to several days, occurring regularly at intervals of days, weeks or months.

---

**KEARNS-SAYRE SYNDROME (KSS)**

**69302**

Progressive external ophthalmoplegia, retinal pigmentary degeneration, cardiac conduction block, short stature, hearing loss, increased cerebrospinal fluid protein, ataxia, cognitive dysfunction, diabetes, and other endocrine disorders. Caused by large deletions in mitochondrial DNA.

FSTRF maintains this code set on behalf of several clinical trials networks for which it is the data center.
Permission must be obtained through user.support@fstrf.org to use or copy it for any other purpose.

## LEIGH SYNDROME (SUBACUTE NECROTIZING ENCEPHALOMYELOPATHY)

**69305**

Degeneration of the central nervous system. Erratic breathing patterns (cyclic or Cheyne-Stokes) or respiratory failure are common. Brain MRI may show a characteristic pattern of lesions in basal ganglia, thalamus and brainstem, but may also be normal. Autopsy shows characteristic neuropathological changes in similar regions.

**Mitochondrial Myopathies:**

Muscle weakness or exercise intolerance due to underlying mitochondrial cytopathy. May be accompanied by other organ system disturbance, commonly heart failure or rhythm disturbances, dementia, movement disorders, stroke-like episodes, deafness, blindness, vomiting, and seizures. Ragged red fibers on muscle biopsy, abnormal mitochondria on electron microscopy, and/or documented muscle oxidative phosphoralation defects are necessary to confirm diagnosis.

## LEBER PROGRESSIVE OPTIC NEUROPATHY

**69304**

Delayed bilateral loss of vision which could lead to total blindness due to degeneration of the optic nerve. Early signs include localized collection of distended blood capillary vessels around the start of the optic nerve.

## MITOCHONDRIAL DNA DEPLETION/CONGENITAL MYOPATHY

**69307**

Neonatal weakness, hypotonia requiring assisted ventilation, possible renal dysfunction, severe lactic acidosis, and prominent ragged-red fibers in muscle biopsy.

## MITOCHONDRIAL DNA DEPLETION/INFANTILE MYOPATHY

**69308**

Following normal early development until one year of age, weakness appears and worsens rapidly, causing respiratory failure and death typically within a few years.

## MITOCHONDRIAL DNA DEPLETION/HEPATOPATHY

**69309**

Enlarged liver and intractable liver function, myopathy, and severe lactic acidosis. Death is typical within the first year.

## MITOCHONDRIAL ENCEPHALOPATHY LACTIC ACIDOSIS *AND* STROKE-LIKE EPISODES (MELAS)

**69310**

Stroke-like episodes with focal neurological deficits, lactic acidosis; may also include short stature, seizures, recurrent headaches, cognitive regression.

FSTRF maintains this code set on behalf of several clinical trials networks for which it is the data center.
Permission must be obtained through user.support@fstrf.org to use or copy it for any other purpose.

## MYOCLONIC EPILEPSY ***AND*** RAGGED-RED FIBER MYOPATHY (MERRF)

**69311**
Myoclonic epilepsy, progressive ataxia, muscle weakness and degeneration, ragged red fibers on biopsy, deafness, and dementia.

## NEUROGENIC PROGRESSIVE EXTERNAL OPHTHALMOPLEGIA

**69312**
Progressive external ophthalmoplegia (abnormal eye movements), progressive proximal muscle weakness, cataracts, ataxia, episodic ketoacidotic coma and episodic ketoacidosis.

## NEUROPATHY, ATAXIA ***AND*** RETINITIS PIGMENTOSA (NARP)

**69313**
Sensory neuropathy, cerebellar ataxia, retinitis pigmentosa, dementia, seizures, developmental delay, and proximal weakness.

## PEARSON'S SYNDROME (PS)

**69314**
Bone marrow involvement (pancytopenia), and exocrine pancreatic insufficiency. This syndrome is caused by large deletions in mitochondrial DNA.

## RENAL FANCONI SYNDROME

**69315**
Proximal tubular dysfunction, causing excretion of glucose, amino acids, uric acid and phosphate. Secondary growth failure, rickets, and osteomalacia may occur.

**69316 MITOCHONDRIAL SYNDROME,** not listed above, specify.

FSTRF maintains this code set on behalf of several clinical trials networks for which it is the data center.
Permission must be obtained through user.support@fstrf.org to use or copy it for any other purpose.

# X. METABOLIC/ENDOCRINE DISORDERS

## ADDISON'S DISEASE

**67210**

Primary adrenal insufficiency manifested by lassitude, malaise, salt-craving and frequently hyponatremia with hyperkalemia are present on laboratory testing. The diagnosis can be confirmed by the finding of low serum cortisol levels particularly in the early morning, accompanied by high levels of ACTH.

## CUSHING'S SYNDROME

**67205**

Primary overactivity of the adrenal glands or secondary adrenal overactivity due to pituitary hypersecretion of ACTH. The clinical picture can also be seen in study participants on high doses of glucocorticoid medication. The picture is one of central obesity with relatively thin limbs. Fat may accumulate at the base of the neck in a buffalo hump. Characteristic striae or stretch marks may be seen on the upper arms, abdomen or flanks. The face is full. The diagnostic workup is complex and is based initially on a high level of cortisol in a 24 hour urine and high serum cortisol levels especially in the evening and night.

## FAILURE TO THRIVE

**67200**

Based on consecutive weight and height measurements at the same site, documenting measurements from a child who downwardly crosses two major percentile lines on a standard growth chart, or who is less than the 5th percentile and fails to parallel the growth curve at the 5th percentile.

## HYPERTHYROIDISM

**67212**

An autoimmune disorder more common in females than males, which causes excessive amounts of thyroid hormone to be secreted. Symptoms include hyperactivity with a large appetite, feeling hot, with the skin warm to the touch. There may be tremors, excessive sweating, and diarrhea. There is usually goiter or thyroid enlargement. The eyes may be protuberant or staring. Thyroxine values are elevated usually above 12 micrograms/dL. TSH is suppressed.

## HYPOTHYROIDISM

**67213**

Underactivity of the thyroid more common in females than males. Symptoms are weight gain, growth failure, lassitude, constipation and feeling cold. The skin may feel dry to the touch and cold. There may be enlargement of the thyroid. Blood tests showed a reduced thyroxine level usually below 4 micrograms/dL and an elevated TSH.

FSTRF maintains this code set on behalf of several clinical trials networks for which it is the data center.
Permission must be obtained through user.support@fstrf.org to use or copy it for any other purpose.

## PRECOCIOUS PUBERTY

**67223**
The premature development of pubertal changes in a young child.

## PREMATURE ADRENARCHE

**67224**
Appearance of sexual hair before the age of 8 in girls or 9 in boys without evidence of maturation.

## PREMATURE THELARCHE

**67217**
Transient condition of isolated breast development.

## SALIVARY GLAND ENLARGEMENT (PAROTID)

**67225 CONFIRMED**
Clinical presentation of enlarged parotid glands, usually bilateral. The symptoms include asymptomatic enlargement and dry mouth (xerostomia) may be present.

## SALIVARY HYPOFUNCTION (HYPOSECRETION)

**67226 CONFIRMED**
Defined as unstimulated whole salivary flow rate less than (<)2.5 mL per 5 minutes (0.5mL/min).

**67219 METABOLIC/ENDOCRINE DISORDER,** other, specify.

FSTRF maintains this code set on behalf of several clinical trials networks for which it is the data center.
Permission must be obtained through user.support@fstrf.org to use or copy it for any other purpose.

# XI. NEUROLOGICAL DISORDERS

---

**AUTISM**

**67305**

Disorder characterized by the presence of markedly abnormal or impaired development in social interaction, qualitative impairment in communication and play and a markedly restricted repertoire of activity or interests. The qualitative impairments are distinctly deviant relative to the individual's developmental level or mental age. The disturbance in social interaction, language for social communication, and symbolic play are manifested prior to three years of age.

---

**CHILDHOOD DISINTEGRATIVE DISORDER**

**67309**

Characterized by a marked regression in multiple areas of functioning following a period of at least two years of normal development. After the first two years of life and before age 10, the child has a clinically significant loss of previously acquired skills in at least two of the following areas: expressive or receptive language, social skills or adaptive behavior, bowel or bladder control, play or motor skills. Individuals with this disorder exhibit the social and communicative deficits and behavioral features observed in autistic disorder; it does not occur in the context of a degenerative disease of the brain or schizophrenia.

---

**68514 CNS DISEASE/DISORDER,** other, specify diagnosis

---

**CNS MASS LESION OF UNDETERMINED ETIOLOGY (PROBABLE ONLY)**

**69920 PROBABLE**

1. Presence of mass lesion(s) on brain imaging study (CT or MRI).

*and*

2. Study participant does not meet all confirmed or probable criteria for other diagnosis with CNS mass lesion (e.g. CNS Toxoplasmosis, CNS Lymphoma).

**69921**

Clinical diagnosis only, testing technology not available to determine diagnosis.

---

**COMMUNICATION DISORDERS, SPECIFY**

**67336**

Characterized by significant difficulties or lack of development of age appropriate speech and/or language skills. These difficulties interfere with academic or occupational achievement or with social communication and are not due to sensory or motor deficit or environmental deprivation. Communication disorders may include expressive language disorder, mixed receptive-expressive language disorder, phonological disorder, and/or stuttering.

FSTRF maintains this code set on behalf of several clinical trials networks for which it is the data center.
Permission must be obtained through user.support@fstrf.org to use or copy it for any other purpose.

## HIV-ASSOCIATED DEMENTIA (ENCEPHALOPATHY)

**NOTE:** When recording this diagnosis specify either dementia or encephalopathy.

**67037 CONFIRMED**

1. Acquired cognitive/motor dysfunction for at least 1 month causing impairment of work or activities of daily living (verifiable by report of a key informant), not attributable solely to severe systemic illness or medication adverse effects.

*and*

2. Abnormalities from at least two of the following categories:
   a. Motor abnormality: For example, slowed rapid movements, release signs, abnormal gait, limb incoordination, diffuse hyperreflexia, hypertonia, or weakness.
   b. Behavioral abnormality: For example, change in personality with apathy, inertia, irritability, and/or emotional lability or new onset of impaired judgment characterized by socially inappropriate behavior or disinhibition.
   c. Cognitive abnormality (two or more): memory, judgment, flexibility, visual, constructional difficulties, reaction time, speed of mental processing, attention and/or concentration as determined by appropriate neuropsychological instruments, with interpretation of abnormality or decline by a neurologist/neuropsychologist.

*and*

3. No other etiology confirmed by MRI/CT scan, negative CSF cryptococcal antigen or CSF CMV PCR: exclude active CNS opportunistic infections or malignancy, active psychiatric disorders, active alcohol or substance use or substance withdrawal.

**67038 PROBABLE**

1. Acquired cognitive/motor dysfunction for at least 1 month causing impairment of work or activities of daily living (verifiable by report of a key informant), not attributable solely to severe systemic illness or medication adverse effects.

*and*

2. Abnormalities from at least two of the following categories:
   a. Motor abnormality: For example, slowed rapid movements, release signs, abnormal gait, limb incoordination, diffuse hyperreflexia, hypertonia, or weakness.
   b. Behavioral abnormality: For example, change in personality with apathy, inertia, irritability, and/or emotional lability or new onset of impaired judgment characterized by socially inappropriate behavior or disinhibition.
   c. Cognitive abnormality (two or more): memory, judgment, flexibility, visual, constructional difficulties, reaction time, speed of mental processing, attention and/or concentration as determined by appropriate neuropsychological instruments, with interpretation of abnormality or decline by a neurologist/neuropsychologist.

*and*

3. Tests for other possible etiology (active CNS opportunistic infections or malignancy, active psychiatric disorders, active alcohol or substance abuse or substance withdrawal) are not completed, results are not available or results do not exclude other CNS processes.

**67039**

Clinical diagnosis only, acquired cognitive/motor dysfunction for at least 1 month causing impairment of work or activities of daily living, not attributable solely to severe systemic illness or medication adverse effects and other possible etiologies do not exclude other CNS processes (active CNS opportunistic infections or malignancy, active psychiatric disorders, active alcohol or substance abuse or substance withdrawal).

---

**68519 ENCEPHALOPATHY,** other, specify

---

**68517 EPILEPSY**

---

**68685 GAIT OR BALANCE DISORDER**

---

**MOTOR DEVELOPMENTAL DELAY**

**69404**

Study participant does not have abnormalities of reflexes, tone or muscle bulk, cognitive development is reasonable but motor landmarks are delayed, for example late walking without weakness, CP, etc.

---

**HIV-ASSOCIATED MYOPATHY**

**67017 CONFIRMED**

1. Symptoms of weakness in proximal muscles (thighs, shoulders, or upper arms) with evidence of proximal weakness on physical examination.

*and*

2. CPK elevated to greater than twice (>2X) normal (no EMG, physical trauma, or IM injection within 2 weeks).

*and*

3. ZDV muscle toxicity excluded either by 1) no history of ZDV in the immediately preceding three months or 2) drug holiday from ZDV for at least one month with no improvement in signs, symptoms, or CPK elevation.

*and*

4. Neurodiagnostic confirmation by either:
   a. EMG documenting myopathic features.

*or*

   b. Muscle biopsy documenting myofiber degeneration or inflammation.

**67018 PROBABLE**

1. Symptoms of weakness in proximal muscles (thighs, shoulders, or upper arms) with evidence of proximal weakness on physical examination.

*and*

2. CPK elevated to greater than twice (>2X) normal (no EMG, physical trauma, or IM injection within 2 weeks).

*and*

3. ZDV muscle toxicity excluded either by 1) no history of ZDV in the immediately preceding three months or 2) drug holiday from ZDV for at least one month with no improvement in signs, symptoms or CPK elevation.

**67019**

Clinical diagnosis only, symptoms of weakness in proximal muscles (thighs, shoulders, or upper arms) with evidence of proximal weakness on physical examination and ZDV muscle toxicity excluded either by 1) no history of ZDV in the immediately preceding three months or 2) drug holiday from ZDV for at least one month with no improvement in signs, symptoms or CPK elevation.

FSTRF maintains this code set on behalf of several clinical trials networks for which it is the data center.
Permission must be obtained through user.support@fstrf.org to use or copy it for any other purpose.

**68684 NEUROLOGICAL DEFICIT, FOCAL**

**68652 NEUROLOGIC SYSTEM DISEASE/DISORDER,** other, specify diagnosis

**SENSORY PERIPHERAL NEUROPATHY**

**NOTE:** For sensory neuropathy diagnoses that are potentially related to medication, go to the sensory neuropathy diagnosis criteria in the Toxicity Evaluation section of this appendix.

**67027 CONFIRMED**

1. Symptoms of pain, burning, numbness, or tingling discomfort in both feet, or both feet and hands, for at least 2 weeks. No history of diabetes mellitus, chemotherapy, or vitamin B12 deficiency.

*and*

2. Examination shows at least two of the following abnormalities:
   a. Diminished or absent ankle reflexes.
   b. Diminished vibration sensation in the toes.
   c. Disturbance in pain or temperature sensation.

*and*

3. Exclusion of nerve toxicity from ddI, ddC, d4T either by history (no ddI, ddC, or d4T for immediately preceding three months) or by drug holiday off these medications for at least 1 month.

*and*

4. Neurodiagnostic confirmation by either:
   a. Nerve biopsy.

*or*

   b. Abnormal nerve conduction testing *and* abnormal quantitative sensory testing (Vibration CASE IV or equivalent.)

**67028 PROBABLE**

1. Symptoms of pain, burning, numbness, or tingling discomfort in both feet, or both feet and hands, for at least 2 weeks. No history of diabetes mellitus, chemotherapy, or vitamin B12 deficiency.

*and*

2. Examination shows at least two of the following abnormalities:
   a. Diminished or absent ankle reflexes.
   b. Diminished vibration sensation in the toes.
   c. Disturbance in pain or temperature sensation.

*and*

3. Exclusion of nerve toxicity from ddI, ddC, d4T either by history (no ddI, ddC, or d4T for immediately preceding three months) or by drug holiday off these medications for at least 1 month.

**67029**

Clinical diagnosis only, symptoms of pain, burning, numbness, or tingling discomfort in both feet, or both feet and hands, for at least 2 weeks. No history of diabetes mellitus, chemotherapy, or vitamin B12 deficiency and examination shows at least two of the following abnormalities: diminished or absent ankle reflexes, diminished vibration sensation in the toes and disturbance in pain or temperature sensation.

FSTRF maintains this code set on behalf of several clinical trials networks for which it is the data center.
Permission must be obtained through user.support@fstrf.org to use or copy it for any other purpose.

**68516 PERIPHERAL NERVE DISEASE/DISORDER,** other, specify diagnosis

**68683 SEIZURE DISORDER (NOT EPILEPSY)**

FSTRF maintains this code set on behalf of several clinical trials networks for which it is the data center.
Permission must be obtained through user.support@fstrf.org to use or copy it for any other purpose.

# XII. OTHER HIV ASSOCIATED DISEASES

**ANGULAR CHEILITIS**

This is a clinical diagnosis without definitive criteria.

**65022 PROBABLE**

1. Red or white fissures or linear ulcers located at the lip commissures or corners of the mouth. There may be no pain or possible mild pain when opening the mouth. The lesions/symptoms are usually intermittent, but may be long-standing.

**RECURRENT APHTHOUS STOMATITIS**

**64049**

Clinical presentation of single or multiple, white/yellow, well circumscribed, painful ulcer(s) on non-keratinized tissue. A red halo is usually present around each ulcer.

Minor aphthous ulcers may be, 0.2 to 0.5 cm in diameter and lasts 7 to 10 days.

Major aphthous ulcers are greater than (>) 0.5 cm in size (may be as large as 2 cm in diameter) and may last for weeks.

There may be moderate to severe pain, especially when eating. Patient reports a long-term history recurrent ulcers.

**SEBORRHEIC DERMATITIS**

This is a clinical diagnosis without definitive criteria.

**69027 PROBABLE**

1. Itchy, scaly skin condition, particularly affecting hairy areas (scalp, axillae, upper trunk and groin).

**UNEXPLAINED PERSISTENT FEVER**

**69026 CONFIRMED**

1. Documented fever of >37.5 º C with negative blood culture, negative Ziehl-Nielsen stain, malaria slide and normal or unchanged chest X-ray and no other obvious foci of infection.

**69025 PROBABLE**

2. Fever or night sweats for more than one month, either intermittent or constant, with reported lack of response to antibiotics or antimalarial agents, without other obvious foci of disease reported or found on examination. Malaria must be excluded in malarious areas.

FSTRF maintains this code set on behalf of several clinical trials networks for which it is the data center.
Permission must be obtained through user.support@fstrf.org to use or copy it for any other purpose.

**NECROTIZING ULCERATIVE GINGIVITIS OR PERIODONTITIS,** specify gingivitis or periodontitis

This is a clinical diagnosis without definitive criteria

**65014 PROBABLE**

1. Destruction of one or more interdental gingival papillae. In the acute stage of the process ulceration, necrosis, and sloughing may be seen with ready hemorrhage and characteristic fetid odor.
2. In the case of necrotizing ulcerative periodontitis, the condition is characterized by soft tissue loss as a result of ulceration or necrosis. Exposure, destruction or sequestration of alveolar bone may be seen, and the teeth may become loosened.
3. Usually of a sudden onset and rapidly worsens. Moderate to severe pain may be a prominent feature.

**IMMUNE RECONSTITUTION INFLAMMATORY SYNDROME (IRIS)**

**64900**

1. Initiation, reintroduction or change in antiretroviral therapy/regimen.

*and*

2. [1] Evidence of:
   a. an increase in CD4+ cell count as defined by ≥50 cells/mm$^3$ or a ≥2-fold rise in CD4+ cell count, **and/or**
   b. decrease in the HIV-1 viral load of >0.5 $\log_{10}$ **and/or**
   c. weight gain or other investigator-defined signs of clinical improvement in response to initiation, reintroduction or change of antiretroviral therapy/regimen.

*and*

3. Symptoms and/or signs that are consistent with an infectious/inflammatory condition

*and*

4. These symptoms and/or signs cannot be explained by a newly acquired infection, the expected clinical course of a previously recognized infectious agent, or the side effects of antiretroviral therapy itself.

*and*

5. For purposes of data collection, the infectious/inflammatory condition must be attributable to a specific pathogen or condition.

[1] If the study participant is being evaluated for an infectious/inflammatory condition at a time that is <4 weeks after initiation, reintroduction or change in antiretroviral therapy/regimen, items 2a-2c are not required.

**HIV-ASSOCIATED NEPHROPATHY**

**68631**

1. Renal biopsy

**PROBABLE PNEUMONIA AND/OR ETIOLOGY UNKNOWN**
**NOTE: This is not to be used for diagnoses of bacterial pneumonia. Refer to the bacterial pneumonia criteria in the Bacterial section of the appendix for the Bacterial Pneumonia diagnoses.**

**69028**
1. Compatible clinical syndrome of pneumonia (e.g., productive cough and fever)

*and*

2. Radiologic evidence of pulmonary infiltrate.

*and*

3. No pathogen identified.

**WASTING SYNDROME**

**NOTE:** Study participants should be carefully assessed for the concurrent existence of abnormal or altered fat distribution. Diagnostic criteria can be found in the document "Diagnostic Criteria for Abnormalities of Fat Redistribution." This document is located at the ACTG Web Site / Global Protocol Support Documents / Metabolic /Fat Redistribution Guidelines at the following link: http://ACTG.s-3.com/ . You must be a registered user to access the Web Site.

**69020 CONFIRMED**
**EITHER A**:
1. Involuntary weight loss of greater than 10% over at least 6 months.

*and*

2. No evidence of concurrent illness or condition (other than HIV infection) that explains or contributes to the ongoing weight loss (i.e., dehydration, edema, simple mechanical impediments to oral intake).

**OR B:**
1. Involuntary weight loss of greater than 5% over 3 consecutive months.

*and*

2. No evidence of concurrent illness or condition (other than HIV infection) that explains or contributes to the ongoing weight loss (i.e., dehydration, edema, simple mechanical impediments to oral intake).

*or*

3. The weight loss must persist for at least 3 consecutive months despite initiation of appropriate treatment for the known concurrent illness or condition.

**PROBABLE**

There is no acceptable definition to be used within the ACTG.

**69022**

Clinical involuntary weight loss >10% of baseline plus chronic diarrhea or chronic weakness and documented fever ≥30 days.

FSTRF maintains this code set on behalf of several clinical trials networks for which it is the data center.
Permission must be obtained through user.support@fstrf.org to use or copy it for any other purpose.

**ULCERATIONS NOS (NOT OTHERWISE SPECIFIED)/NECROTIZING ULCERATIVE STOMATITIS**, specify ulcerations NOS, oral or necrotizing ulcerative stomatitis

**65013 CONFIRMED**

Histologic features are those of non-specific ulceration. Microbiologic studies fail to identify a specific etiologic agent.

**65012 PROBABLE**

Large (>0.5 cm and sometimes up to 3 cm) ulceration(s) with white/yellow necrotic base that may be located on either keratinized or non-keratinized mucosa. NOTE: The clinical appearance is similar to that of major aphthous ulcer, but there is no history of recurrent lesions.

Necrotizing ulcerative stomatitis presents as localized, painful ulceronecrotic lesions of the oral mucosa that exposes underlying bone or penetrates or extends into contiguous tissues. Severe pain may be a prominent feature. It has a sudden onset, but may be long-standing and/or recurrent.

**OTHER CLINICAL EVENT DIAGNOSIS**

**69999 CONFIRMED, PROBABLE**

HIV associated clinical endpoints or events potentially related to medications not otherwise specified in this appendix. Refer to the CDC Case Definitions of AIDS, the local investigator and/or the protocol team for further guidance.

FSTRF maintains this code set on behalf of several clinical trials networks for which it is the data center.
Permission must be obtained through user.support@fstrf.org to use or copy it for any other purpose.

# XIII. CARDIOVASCULAR DISEASES

There is no distinction between confirmed or probable definitions for cardiovascular diseases.

---

**ANGINA PECTORIS**

**68222**

1. History of chest discomfort caused by exertion or excitement and alleviated with rest. May be described as pain but more frequently as heaviness, pressure, squeezing or choking sensation. May radiate to the left shoulder, down the arm, back, neck or jaw.

*or*

2. Atypical presentation with a report of at least one of the following:
   a. Electrocardiograph consistent with acute ischemia.
   b. Stress test findings consistent with ischemia.
   c. Angiogram of the coronary arteries demonstrating significant occlusion(s), and other etiologies of the presenting signs and symptoms are unlikely.

---

**AORTIC ANEURYSM**

**68270**

Radiographic or surgical evidence of an aortic aneurysm.

**68271**

Clinical diagnosis only, testing technology not available to determine diagnosis.

---

**ARRHYTHMIA, SIGNIFICANT,** specify arrhythmia

**68250**

A cardiac arrhythmia present on an ECG and/or rhythm strip causing or with the potential to cause clinically significant hemodynamic consequences.

**68251**

Clinical diagnosis only, testing technology not available to determine diagnosis.

---

**CARDIOMYOPATHY, HIV-ASSOCIATED, SYMPTOMATIC, (**WHO Stage 4 guidelines)

**68132 CONFIRMED**

Cardiomegaly and evidence of poor left ventricular function confirmed by echocardiography.
Note: It may not be possible to discern the etiology for cardiomyopathy.

**PROBABLE**

This is a definitive diagnosis without clinical criteria.

FSTRF maintains this code set on behalf of several clinical trials networks for which it is the data center.
Permission must be obtained through user.support@fstrf.org to use or copy it for any other purpose.

## DEEP VEIN THROMBOSIS (DVT)

**68224 CONFIRMED**

1. Clinical presentation of swelling and/or pain/tenderness of one or both lower extremities.

*and*

2. Findings consistent with DVT on ultrasound, Doppler, computerized tomography (CT) or other acceptable diagnostic method.

**68225 PROBABLE**

1. Clinical presentation of swelling and/or pain/tenderness of one or both lower extremities.

## HYPERTENSION

**NOTE**: The diagnosis of hypertension should be made by the study participant's clinician and not diagnosed solely on the blood pressure measurements obtained during research visits.

**68210**

1. A clinical diagnosis of hypertension is based on the average diastolic blood pressure >90 mmHg and/or systolic blood pressure of >140 mmHg in an adult not taking antihypertensive medications and not acutely ill. Based on the average of two or more readings taken at each of two or more visits after the first elevated blood pressure was obtained.

*or*

2. Antihypertensive treatment or a regimen of diet and exercise prior to starting antihypertensive medication recommended or initiated. This includes initial treatment with diuretics to control the hypertension.

## LEFT VENTRICULAR FAILURE

**68139**

1. One or more of the following: paroxysmal nocturnal dyspnea, or dyspnea at rest, or orthopnea, or New York Heart Classification III.

   (Note: NY Heart Class III* is defined as "Study participant with cardiac disease resulting in marked limitation of physical activity. They are comfortable at rest. Less than ordinary activity causes fatigue, palpitation, dyspnea, or anginal pain." *1994 Revisions to Classification of Functional Capacity and Objective Assessment of Study participants With Disease of the Heart, AHA.)

*and*

2. At least one of the following signs must be present: rales, 2+ or greater ankle edema, tachycardia of 120 beats/minute or more after 5 minutes at rest, cardiomegaly by chest X-ray, chest X-ray characteristic of congestive failure, S3 gallop, or jugular venous distention.

**68142**

Clinical diagnosis only, testing technology not available to determine diagnosis.

FSTRF maintains this code set on behalf of several clinical trials networks for which it is the data center.
Permission must be obtained through user.support@fstrf.org to use or copy it for any other purpose.

## LEFT VENTRICULAR HYPERTROPHY

**68131**

An interpretation of an electrocardiograph indicating findings consistent with diagnosis of left ventricular hypertrophy. (For example, Cornell criteria require the sum of the amplitude of R wave in avL lead and S wave in V3 lead is greater than 28 in males or 20 in females.)

## MYOCARDIAL INFARCTION, ACUTE (SYMPTOMATIC)

**68220 CONFIRMED**

1. Symptoms suggestive of myocardial infarction.

*and*

2. Cardiology report of electrocardiograph indicating findings consistent with myocardial infarction. (For example, new Q wave present in 2 or more contiguous leads and with either duration ≥ 40 msec or amplitude > ¼ of R wave.)

*or*

3. Significant elevation of serum enzymes as demonstrated by one of the following:
   a. CPK-MB present, or above upper limit of normal (depending on how local lab records) within 36 hours of onset of acute symptoms of MI.
   b. Reversal of LDH/LDH2 ratio within 5 days of the onset of acute symptoms of MI.
   c. CPK total at least 1.25 times the upper limit of normal for the laboratory that performed the test (in the absence of other possible causes for elevation of the CPK total and with CPK-MB missing, not done, or done more than 36 hours after onset of symptoms).
   d. SGOT, LDH or other cardiac enzymes at least 1.25 times the upper limit of normal for the laboratory that performed the test (in the absence of other possible causes for elevation of the enzymes and with CPK-MB missing, not done, or done more than 36 hours after the onset of symptoms.)
   e. Elevation (> 0.1ng/mL) in serum cardiac-specific troponin I (cTnI) and troponin T (cTnT). (Note: Serum levels of cTnI and cTnT increase 3-12 hours after onset of MI, reach a peak in 24-48 hours, and return to baseline over 5-14 days.)

**68221 PROBABLE**

Clinical diagnosis only, testing technology not available to determine diagnosis.

FSTRF maintains this code set on behalf of several clinical trials networks for which it is the data center.
Permission must be obtained through user.support@fstrf.org to use or copy it for any other purpose.

**MYOCARDIAL INFARCTION, SILENT** (found at routine ECG or on hospital ECGs**)**

**68223**

Cardiology report of electrocardiograph indicating findings consistent with myocardial infarction. (For example, new Q wave present in two (2) or more contiguous leads and with either duration ≥ 40 msec or amplitude > ¼ R wave.)

**PERIPHERAL VASCULAR DISEASE (PVD)**

**NOTE**: Intermittent claudication is a symptom of peripheral vascular disease. Report only diagnoses of PVD using this diagnosis code. Symptoms in the absence of a physician's diagnosis should be reported on a sign and symptom form.

**68141**

1. Recurring episodes of pain, ache, cramp, numbness or sense of fatigue in either leg (usually calf) occurring during exercise. (intermittent claudication)

*and*

2. Symptom(s) does not resolve during exercise but is relieved with rest.

**PULMONARY EMBOLUS**

**68160 CONFIRMED**

Radiographic evidence of an embolus in the pulmonary tree.

**68161 PROBABLE**

Signs and symptoms consistent with a thrombosis in the pulmonary tree, radiographic evidence non-diagnostic or not done.

FSTRF maintains this code set on behalf of several clinical trials networks for which it is the data center.
Permission must be obtained through user.support@fstrf.org to use or copy it for any other purpose.

## APPENDIX 60 - Diagnoses Appendix

---

**STROKE,** specify hemorrhagic, ischemic, or unknown

**68180**

1. Demonstrable lesion compatible with an acute stroke on a CT (or MRI).

<u>*or*</u>

2. Rapid onset of a neurologic deficit persisting for at least 24 hours which is:
   a. Attributed to an obstruction or rupture of the arterial system.

<u>*and*</u>

   b. Not known to be secondary to brain trauma, tumor, infection or other cause.

For study participants that satisfy the above criteria, select the type of stroke:

**A. HEMORRHAGIC STROKE**

1. Blood in subarachnoid space or intraparenchymal hemorrhage by CT scan. (Intraparenchymal blood must be dense and not mottled-mixed hyperdensity and hypodensity.)

<u>*or*</u>

2. Bloody spinal fluid by lumbar puncture. (Bloody CSF means >100 cells/cu mm. The LP is thought to be non-traumatic and counts in the last tube are similar to those in the first tube [no clearing] or xanthochromia when the specimen is spun down.)

<u>*or*</u>

3. Surgical evidence of hemorrhage as cause of clinical syndrome.

**B. ISCHEMIC INFARCTION**

1. Focal brain deficit without CT or LP evidence of blood, except mottled cerebral pattern. Either decreased density by CT in a compatible location or a negative CT or none done.

<u>*or*</u>

2. Surgical evidence of ischemic infarction.

**C. UNKNOWN TYPE OF STROKE**

Inadequate information to categorize as hemorrhagic or ischemic infarction. Satisfies criteria for stroke.

**68181**

Clinical diagnosis only, testing technology not available to determine diagnosis.

---

**TRANSIENT ISCHEMIC ATTACK**

**NOTE**: Discovery of an infarct by CT in a location compatible with the symptoms, even if the symptoms cleared in less than 24 hours, shall be diagnosed as a stroke.

**68183**

1. One or more episodes of focal neurologic deficit lasting more than 30 seconds and no longer than 24 hours with rapid evolution of the symptoms to the maximal deficit in less than 5 minutes with complete resolution and no immediately preceding head trauma.

<u>*and*</u>

2. There should be no evidence of clonic jerking, conjugate eye deviation, prolonged Jacksonian march, scintillating scotoma, headache with nausea and vomiting.

FSTRF maintains this code set on behalf of several clinical trials networks for which it is the data center.
Permission must be obtained through user.support@fstrf.org to use or copy it for any other purpose.

## OTHER CARDIOVASCULAR DISEASES

**68132 CARDIOMYOPATHY,** HIV-related

**68143 CARDIOMYOPATHY,** drug-induced

**68144 CARDIOMYOPATHY,** etiology unknown

**68145 PULMONARY HYPERTENSION**

**68146 SHOCK**

**68148 HYPOTENSION**

**68199 CARDIOVASCULAR SYSTEM DISEASE/DISORDER,** other, specify

FSTRF maintains this code set on behalf of several clinical trials networks for which it is the data center.
Permission must be obtained through user.support@fstrf.org to use or copy it for any other purpose.

# XIV. TOXICITY EVALUATION

The following diagnoses may or may not be related to medications that the study participant is receiving

---

**CORONARY HEART DISEASE (CHD)/ CORONARY ARTERY DISEASE (CAD)**
See ALSO Appendix Section XIII-Cardiovascular Diseases for specific cardiovascular diagnoses

**68147**

The Cardiovascular Diseases Study Group recommends that we use the term CHD (coronary heart disease) in order to include all clinically suspected and/or angiographically confirmed CAD (coronary artery disease).

**Disease State Description**

None

---

**DIABETES MELLITUS/IMPAIRED GLUCOSE**

**68021 IMPAIRED FASTING GLUCOSE**
**68022 IMPAIRED GLUCOSE TOLERANCE**
**68023 DIABETES MELLITUS**

| | |
|---|---|
| Normoglycemia: | Fasting plasma glucose of <110 mg/dL *or* 2-hour post glucose load plasma glucose <140 mg/dL. |
| Impaired Fasting Glucose: | Fasting plasma glucose of ≥110 mg/dL and <126 mg/dL. |
| Impaired Glucose Tolerance: | 2-hour post glucose load plasma glucose ≥140 mg/dL and <200 mg/dL. |
| Diabetes Mellitus: | Fasting plasma glucose ≥126 mg/dL *or* 2-hour post glucose load plasma glucose ≥200 mg/dL *or* non-fasting plasma glucose ≥200 mg/dL accompanied by symptoms of diabetes mellitus (polyuria, polydipsia, dehydration, blurred vision, new vaginal candidiasis). |

NOTE: If an ACTG study participant has a fasting plasma glucose value suggestive of a diagnosis of diabetes but no other symptoms of diabetes, the fasting glucose value must be confirmed.

**Disease State Description:**

Level A: Impaired fasting glucose
Level B: Impaired glucose tolerance
Level C: Diabetes Mellitus

FSTRF maintains this code set on behalf of several clinical trials networks for which it is the data center.
Permission must be obtained through user.support@fstrf.org to use or copy it for any other purpose.

## FAT ACCUMULATION (LIPODYSTROPHY)

**67221**

Symptoms due to fat accumulation in various places occur following the initiation or change of antiretroviral therapy. These include increasing abdominal girth with an increasing belt or waist size which may be accompanied by complaints of bloating or distension; fat accumulation in the back of the neck or increasing neck size; increasing breast size which may be accompanied by complaints of breast pain; and other new fat accumulations either circumscribed (lipomas) or general such as increase in chest size in absence of breast enlargement. In reporting fat accumulation, the body area involved needs to be specified. In males gynecomastia can present as unilateral breast enlargement, occasionally with nodular lesions.

**Abdominal and/or Truncal Obesity:**

**Possible:** Self-report of increasing abdominal girth; increasing belt or waist size (may be accompanied by complaints of bloating, distension)

**Definite:**

- Cross-sectional: Self-reported increase plus waist-to-hip ratio (WHR) > 0.95 (M); 0.85(F)
- Longitudinal: Measured increase in waist circumference of 2.5 cm (1") or 5% increase in WHR, sagittal diameter, or abdominal fat (by paired MRI, DEXA, or CT measurements obtained under identical, controlled conditions) in the past 12 months

**Dorsocervical Fat Pad Enlargement (Buffalo Hump):**

**Possible:** Self-report of increasing size of dorsocervical region; may be accompanied by increasing shirt neck size or inability to button shirts

**Definite:**

- Cross-sectional: Physical findings consistent with accumulations of fat in dorsocervical area
- Longitudinal: Measured increase in neck circumference of 1.5 cm (0.5") in the past 12 months

**Breast Enlargement (Both Genders):**

**Possible:** Self-report of increasing bra size or shirt/blouse size to accommodate increasing breast size; may be accompanied by complaints of breast pain

**Definite:**

- Cross-sectional: Self-reported increase plus physical findings consistent with enlarged breasts due to increase in fat deposition (note: gynecomastia is an increase in breast tissue, a distinct syndrome and finding)
- Longitudinal: Measured increase in chest circumference of 5% in past 12 months

**Other New Fat Accumulation (Must Specify Location):**

**Possible:** Self-report of new regional circumscribed accumulation of fat; increase in neck size in absence of dorsocervical fat pad enlargement; increase in chest size in absence of breast enlargement.

**Definite:**

- Cross-sectional: Self-report of new fat accumulation plus physical findings consistent with lipoma(s) or lipomatosis (multiple fat accumulations or one > 2 cm)

**Disease State Description:**

Level A: Study participant and/or only close friends/family notice the changes.
Level B: Physical evidence of fat accumulation noted by physician and confirmed by study participant.
Level C: Increase in regional fat is very concerning to the study participant and very obvious to others.

FSTRF maintains this code set on behalf of several clinical trials networks for which it is the data center.
Permission must be obtained through user.support@fstrf.org to use or copy it for any other purpose.

## FAT LOSS (LIPOATROPHY)

**67215**

Face — Study participant may report "sunken cheeks" or "drawn face" or indicate that family members or friends have noticed such changes since initiation or change of antiretroviral therapy. The loss of facial tissue should be just proximal to the nasolabial fold. (This is the area of the buccal fat pad, the largest fat deposit in the face.)

Extremities — Study participant reports that pants/slacks are progressively fitting more loosely through the thighs, new onset of looseness of watch or wristbands, and awareness that the extremities appear thinner since the initiation or change of antiretroviral therapy. The relationship is strengthened by reporting of awareness that veins in the extremities appear more prominent. On exam, extremities appear thin and veins prominent.

Buttocks — Self-reported change in the buttocks in which there is a perception of loss of volume in the subgluteal region, since the initiation or change of antiretroviral therapy. Loss of firmness is by itself not diagnostic as it could be due to muscle atrophy.

**Disease State Description:**

Level A: Study participant and/or only close friends/family notice the changes

Level B: Physical evidence of fat depletion noted by physician and confirmed by study participant

Level C: Loss of fat is very concerning to the study participant and very obvious to others, or 25% decrease in fat as documented by paired measurement of BIA, DEXA, ultrasound (for facial LA) or MRI.

FSTRF maintains this code set on behalf of several clinical trials networks for which it is the data center.
Permission must be obtained through user.support@fstrf.org to use or copy it for any other purpose.

## LACTIC ACIDEMIA/ LACTIC ACIDOSIS

**68657 HYPERLACTACIDEMIA (LACTIC ACIDEMIA),** specify Asymptomatic or Symptomatic

**68654 LACTIC ACIDOSIS**

Lactate level greater than the upper limit of normal(ULN) confirmed by repeat lactate level analysis may be part of a syndrome referred to as lactic acidemia or lactic acidosis.

Lactic acidemia refers to the presence of plasma lactate above ULN (confirmed) without evidence of a metabolic acidosis. In addition lactic acidemia may be symptomatic or asymptomatic. As lactate levels are highly dependent on collection techniques, careful attention to collection guidelines is necessary and high lactate levels should be repeated for verification. (See "ACTG Venous Lactate Specimen Collection and Storage Guidelines" at http://ACTG.s-3.com/member/psmet.htm)

Lactic acidosis is a potentially life-threatening condition and presents with elevated plasma lactate level AND an arterial pH less than 7.35, in general with low bicarbonate or increased anion gap. It is usually accompanied by symptoms which may be vague and/or subtle.

**Subcategorization:**

- Asymptomatic
- Symptomatic: New, otherwise unexplained occurrence of one or more of the following symptoms:
  - Nausea and/or vomiting
  - Abdominal pain or gastric discomfort
  - Abdominal distention
  - Increased hepatic transaminase levels
  - Unexplained fatigue
  - Dyspnea
  - Weight loss ≥ 5% body weight
  - Muscle weakness

**Disease State Description**

Level A: Asymptomatic lactic acidemia < 2x upper limit of normal (ULN) confirmed by repeat lactate level analysis

Level B: Asymptomatic lactic acidemia ≥ 2x upper limit of normal (ULN) confirmed by repeat lactate level analysis

Level C: Symptomatic lactic acidemia or lactic acidosis.

FSTRF maintains this code set on behalf of several clinical trials networks for which it is the data center.
Permission must be obtained through user.support@fstrf.org to use or copy it for any other purpose.

**LIVER DISEASE,** specify type (this includes but is not limited to the most common causes listed below)
If there is a diagnosis of Chronic Hepatitis B or Chronic Hepatitis C, refer to Appendix Section IV-Viral Infections, for criteria.

**68511**

Development of abnormal liver enzymes (ALT and/or AST and/or alkaline phosphatase) or elevation in bilirubin in a study participant with previously normal tests, or further increases (to grade ≥ 3) in a study participant with chronic abnormal levels. Most common causes in HIV-infected study participants are drug injury, viral hepatitis, steatosis, cholelithiasis, tumors, and other non-drug related conditions. In study participants with known chronic hepatitis B or C, certain drugs may worsen already present liver abnormalities, e.g., nevirapine (hepatocellular damage), indinavir and atazanavir (unconjugated bilirubinemia), lopinavir/ritonavir (hepatocellular damage), etc.

1. In study participants with normal liver tests prior to initiating or changing antiretroviral therapy, the following evaluations are recommended.

    a. History, physical examination, blood count, liver chemistries, ultrasound of the liver, liver biopsy (optional) and laboratory tests for viral hepatitis:
        i. HAVAb IgM (for acute disease; test HAVAb IgG if negative; vaccinate if HAVAb IgG negative)
        ii. HbsAg, HbcAb total (HBV DNA if either is positive)
        iii. HCVAb (HCV RNA if positive or CD4 <200)

2. In study participants with abnormal liver tests prior to initiating or changing antiretroviral therapy, assessment of the cause should be undertaken:
    a. As in previous section
    b. If chronic HBV, test HBV DNA
    c. If chronic HCV, test HCV RNA
    d. Liver biopsy strongly recommended to assess amount of inflammation and stage of fibrosis

3. All study participants with suspected drug-related liver disease must have:
    - No evidence of acute viral hepatitis
    - No evidence of tumor
    - No evidence of cholelithiasis
    - No evidence of non-drug related hepatic injury

**<u>Disease State Description</u>**

Level A: Elevation in hepatic transaminase and/or bilirubin only.
Level B: Elevation in hepatic transaminase and/or bilirubin plus clinical symptoms suggestive of liver disease.
Level C: Hospitalization, severe liver disease, or death due to liver disease.

FSTRF maintains this code set on behalf of several clinical trials networks for which it is the data center.
Permission must be obtained through user.support@fstrf.org to use or copy it for any other purpose.

## 68632 NEPHROPATHY, DRUG-INDUCED

## HIV-ASSOCIATED NEUROMUSCULAR WEAKNESS SYNDROME

**68007**

New onset of limb weakness in an HIV-infected individual, with or without sensory involvement; either acute (7-14 days) or subacute (>14 days) and affecting either lower or both lower and upper extremities.
**NOTE**: Abnormal lactic acid is not required for the definition.

Probability diagnosis based on:
Possible: Consistent clinical features
Probable: Confounding diagnosis excluded with work-up
Definite: NCV/EMG and/or nerve/muscle biopsy confirmation of neuromuscular disease

**Disease State Description**:
Level A: Muscle weakness appreciable, but not significantly limiting everyday functioning.
Level B: Muscle weakness significantly limiting everyday functioning (e.g., walking, climbing stairs, carrying groceries).
Level C: Muscle weakness resulting in requiring a wheelchair, being bed bound, or requiring respiratory support.

## SENSORY NEUROPATHY

**68009**

1. New or recurrent sensory symptoms bilaterally in the lower extremities for at least 14 consecutive days, including numbness or parasthesias (tingling) or dysesthesias (burning, shooting or stabbing pain), spontaneous or evoked.

*and*

2. Reduced or absent ankle reflexes

*and*

3. Abnormal sensory exam: reduced pinprick or reduced vibration.

**Disease State Description:**
Level A: Meets the case definitions. No therapy required.
Level B: Symptomatic therapy required. No limitation of ADLs.
Level C: Therapy required and ADLs are limited by the neuropathy.

Note: ADLs are activities of daily living such as ambulation, bathing, dressing, grooming, feeding, toileting.

FSTRF maintains this code set on behalf of several clinical trials networks for which it is the data center.
Permission must be obtained through user.support@fstrf.org to use or copy it for any other purpose.

## OSTEOPENIA/OSTEOPOROSIS

**68674 OSTEOPENIA**
**68675 OSTEOPOROSIS**

Osteopenia/osteoporosis is defined as a skeletal disorder characterized by compromised bone strength predisposing to an increased risk of fracture. The only difference between the terms is the grade, osteopenia being less severe as determined by dual-energy X-ray absorptiometry (DEXA) t-score.

Currently there is no accurate measure of overall bone strength. Bone mineral density (BMD) is frequently used as a proxy measure and accounts for approximately 70 percent of bone strength. The World Health Organization (WHO) operationally defines osteoporosis as bone density 2.5 standard deviations below the mean for young white adult women. It is not clear how to apply this diagnostic criterion to men, children, and across ethnic groups. *By extension these criteria are used in those groups (comparing to individuals of the same gender and race at age 30 in adults, and same-age children).*

Several different techniques have been developed to assess BMD at multiple skeletal sites including the peripheral skeleton, hip and spine. The World Health Organization (WHO) has selected BMD measurements to establish criteria for the diagnosis of osteoporosis. Although t-scores were based originally on assessment of BMD at the hip by DEXA, they have been applied to define diagnostic thresholds at other skeletal sites and for other technologies.

World Health Organization (WHO) criteria for diagnosis of osteoporosis based on BMD:
Normal t-score ≥ -1
Osteopenia t-score > -2.5 but < -1
Osteoporosis t-score ≤ -2.5
Severe Osteoporosis t-score ≤ -2.5 *and* evidence of fragility fracture

**NOTE**: If an ACTG study participant has clinical evidence of osteoporosis but has not received a DEXA scan, the primary care provider should obtain a DEXA scan to quantify the study participant's BMD.

**Disease Sate Description**
Level A: Osteopenia (t-score < -1 but > -2.5)
Level B: Osteoporosis (t-score ≤ -2.5)
Level C: Severe osteoporosis (t-score ≤ -2.5 and evidence of fragility fracture)

FSTRF maintains this code set on behalf of several clinical trials networks for which it is the data center.
Permission must be obtained through user.support@fstrf.org to use or copy it for any other purpose.

## PANCREATITIS DOCUMENTATION

### 68625 CLINICAL OR SYMPTOMATIC

Clinical or symptomatic pancreatitis is defined by the symptoms of nausea, vomiting, and/or abdominal pain of any duration associated with ≥ Grade 3 elevations of lipase (>2.0 x ULN) and without other non-pancreatic diagnoses to reasonably account for the presentation. Symptoms associated with persistent elevations of lipase < Grade 3 require radiographic evaluation, preferably by CT scan, to confirm the diagnosis. If radiographic evaluation does not confirm pancreatitis, symptoms and lipase evaluated every two weeks until resolution or progression to confirmed pancreatitis. The severity level of pancreatitis is determined by the highest severity of the symptoms.

### 68626 CHEMICAL ***OR*** ASYMPTOMATIC

Chemical or asymptomatic pancreatitis is defined as persistent (2 determinations, 2 weeks apart) elevations in lipase ≥ Grade 3. Severity for chemical pancreatitis in absence of symptoms is always rated as mild.

### 68628

Clinical diagnosis only, testing technology not available to determine diagnosis. Clinical symptoms < Grade 3 and no radiographic evaluation available.

## DRUG-RELATED RASH (not hypersensitivity reaction)

This is a clinical diagnosis without definitive criteria.

### 68529 PROBABLE

Papular pruritic lesions, often with marked post-inflammatory pigmentation.

## RENAL INSUFFICIENCY, ACUTE

### 68025

Increases in serum creatinine to values >1.5 mg/dL (or >1.0-1.3 x ULN) that return to normal values within 3 months or less.

**Disease State Description**

Score yes/no.

## RENAL INSUFFICIENCY, CHRONIC

### 68026

Increases in serum creatinine to values >1.5 mg/dL (or >1.0-1.3 x ULN) that persist for > 3 months.

**Disease State Description**

Level A: Medication dosage adjustments not required.
Level B: Medication dosage adjustments required.
Level C: Requires dialysis.

FSTRF maintains this code set on behalf of several clinical trials networks for which it is the data center.
Permission must be obtained through user.support@fstrf.org to use or copy it for any other purpose.

## NEUROPSYCHIATRIC CONDITIONS TOXICITY EVALUATION

### 68345 ANXIETY

Anxiety is defined as a new onset of persistent and excessive anxiety and worry for at least 10 out of 14 consecutive days.

**Disease State Description**:

Level A: Meets case definition.
Level B: Symptoms require treatment or interfere with usual activities or ADLs.
Level C: ADLs limited by the mood disorder *or* hospitalization required.

Usual Activities: All life activities (job, hobbies, social life, etc.) excluding ADLs.
ADLs: Activities of daily living such as ambulation, bathing, dressing, grooming, feeding, and toileting.

### 68682 DEPRESSION

1. Depression is defined as new onset of depressed mood and/or loss of interest or pleasure for at least 10 out of 14 consecutive days.

*and*

2. Five or more of the following symptoms (occurring nearly every day for symptoms 1-5):
   a. Depressed mood (e.g., sadness, tearfulness) most of the day
   b. Markedly diminished interest in most activities
   c. Insomnia or drowsiness
   d. Psychomotor agitation or retardation
   e. Feelings of worthlessness or excessive guilt
   f. Fatigue or loss of energy
   g. Indecisiveness or diminished concentration
   h. Recurrent thoughts of death or recurrent suicidal ideation
   i. Weight loss or gain (5% body weight change in 1 month)

NOTE: If symptoms listed above are used to make the diagnosis of depression, do not fill out separate Tox-EG forms for individual neuropsychiatric conditions.

**Disease State Description**:

Level A: Meets case definition.
Level B: Symptoms require treatment or interfere with usual activities but not ADLs.
Level C: ADLs limited by the mood disorder *or* hospitalization required.

Usual Activities: All life activities (job, hobbies, social life, etc.) excluding ADLs.
ADLs: Activities of daily living such as ambulation, bathing, dressing, grooming, feeding, and toileting.

FSTRF maintains this code set on behalf of several clinical trials networks for which it is the data center.
Permission must be obtained through user.support@fstrf.org to use or copy it for any other purpose.

**68043 DREAM ABNORMALITY**

Dream abnormality is defined as new onset of abnormal dreams which are described by the study participant as vivid, bizarre or frightening and which have been present for at least 4 days out of 7 consecutive days.

**Disease State Description**:

Level A: Meets case definition.
Level B: Symptoms require treatment or interfere with usual activities but not ADLs.
Level C: ADLs limited by the dream disorder *or* hospitalization required.

Usual Activities: All life activities (job, hobbies, social life, etc.) excluding ADLs.
ADLs: Activities of daily living such as ambulation, bathing, dressing, grooming, feeding, and toileting.

**68042 DROWSINESS**

Drowsiness is defined as new or worsening pathologic increase in absolute sleep hours by 25%, present for at least 7 of 14 consecutive days.

**Disease State Description:**

Level A: Meets case definition.
Level B: Symptoms require treatment or interfere with usual activities but not ADLs.
Level C: ADLs limited by drowsiness *or* hospitalization required.

Usual Activities: All life activities (job, hobbies, social life, etc.) excluding ADLs.
ADLs: Activities of daily living such as ambulation, bathing, dressing, grooming, feeding, and toileting.

**68038 HALLUCINATIONS**

Hallucinations is defined as new onset and presence of false visual, auditory, tactile, olfactory or gustatory perceptions that have no basis in external stimulation.

**Disease State Description:**

Level A: Meets case definition.
Level B: Symptoms require treatment or interfere with usual activities but not ADLs.
Level C: ADLs limited by the hallucinations *or* hospitalization required.

Usual Activities: All life activities (job, hobbies, social life, etc.) excluding ADLs.
ADLs: Activities of daily living such as ambulation, bathing, dressing, grooming, feeding, and toileting.

FSTRF maintains this code set on behalf of several clinical trials networks for which it is the data center.
Permission must be obtained through user.support@fstrf.org to use or copy it for any other purpose.

## 68035 HYPOMANIA/MANIA

1. A hypomanic episode is defined as a distinct period of abnormal and persistently elevated, expansive mood lasting at least 3 out of 4 consecutive days. A manic episode is defined as a distinct period of abnormal and persistently elevated, expansive mood lasting at least 6 out of 7 consecutive days.

*and*

2. Three or more of the following: Inflated self-esteem, decreased need for sleep (feels rested after only 3 hours of sleep), pressure to keep talking, flight of ideas or racing thoughts, distractibility, increase in goal-directed activity, excessive and risky pleasure-seeking (e.g., unrestrained buying sprees, sexual indiscretions, foolish investments, etc.).

**Disease State Description**:

Level A: Meets case definition.
Level B: Symptoms require treatment or interfere with usual activities but not ADLs.
Level C: ADLS limited by the mood disorder *or* hospitalization required.

Usual Activities: All life activities (job, hobbies, social life, etc.) excluding ADLs.
ADLs: Activities of daily living such as ambulation, bathing, dressing, grooming, feeding, and toileting.

## 68041 INSOMNIA

Insomnia is defined as new or worsening insomnia at least 7 days out of 14 consecutive days and characterized by difficulty in falling asleep or in staying asleep or by disturbed sleep patterns resulting in insufficient sleep.

**Disease State Description:**

Level A: Meets case definition.
Level B: Symptoms require treatment or interfere with usual activities but not ADLs.
Level C: ADLs limited by the insomnia *or* hospitalization required.

Usual Activities: All life activities (job, hobbies, social life, etc.) excluding ADLs.
ADLs: Activities of daily living such as ambulation, bathing, dressing, grooming, feeding, and toileting.

## 68036 PSYCHOSIS

Psychosis is defined as a new onset and presence for at least 2 days out of 14 consecutive days of delusional thought patterns (e.g., thoughts of persecution, thought broadcasting, thought insertion, or thoughts of reference) or disorganized speech or grossly disorganized or catatonic behavior.

**Disease State Description**:

Level A: Meets case definition.
Level B: Symptoms require treatment or interfere with usual activities but not ADLs.
Level C: ADLs limited by the psychosis *or* hospitalization required.

Usual Activities: All life activities (job, hobbies, social life, etc.) excluding ADLs.
ADLs: Activities of daily living such as ambulation, bathing, dressing, grooming, feeding, and toileting.

FSTRF maintains this code set on behalf of several clinical trials networks for which it is the data center.
Permission must be obtained through user.support@fstrf.org to use or copy it for any other purpose.

**68037 SUICIDAL IDEATION**

Suicidal Ideation is defined as new onset of suicidal thoughts.

**Disease State Description**:

Level A: Suicidal thoughts only.
Level B: Study participant has suicidal thoughts *and* a suicide plan.
Level C: Study participant has attempted suicide.

FSTRF maintains this code set on behalf of several clinical trials networks for which it is the data center.
Permission must be obtained through user.support@fstrf.org to use or copy it for any other purpose.

# XV. OTHER DISEASE CODES

**These codes are to be used to report diagnoses not specified in other sections of this appendix.**

**DERMATOLOGIC**

**68521 SKIN DISEASE/DISORDER, OTHER,** specify diagnosis

**68525 STEVENS-JOHNSON SYNDROME**

**68524 STUDY DRUG RELATED HYPERSENSITIVITY REACTION**

**68601 OTHER DRUG-INDUCED HYPERSENSITIVITY REACTION** (i.e. Abacavir-like)

**GASTROINTESTINAL**

**68510 GASTROINTESTINAL SYSTEM DISEASE/DISORDER, OTHER,** specify diagnosis

**GENITOURINARY**

**68526 NEPHROLITHIASIS**

**68527 PROXIMAL RENAL TUBE DYSFUNCTION (PRTD)** (Fanconi-like syndrome)

**68512 RENAL SYSTEM DISEASE/DISORDER, OTHER,** specify diagnosis

**METABOLIC/ENDOCRINE**

**68650 DIABETIC KETOACIDOSIS**

**68653 HYPOGONADISM**

**68655 LIPID ABNORMALITY**, specify abnormality

**68656 WEIGHT LOSS** (See Appendix Section XII - Other HIV Associated Diseases for Wasting Syndrome)

**MUSCULOSKELETAL**

**68670 ARTHRITIS**, specify type

**68671 AVASCULAR NECROSIS**

**68672 FRACTURE**

**68528 MYOSITIS**

**68518 MUSCULOSKELETAL SYSTEM DISEASE/DISORDER, OTHER,** specify diagnosis

FSTRF maintains this code set on behalf of several clinical trials networks for which it is the data center.
Permission must be obtained through user.support@fstrf.org to use or copy it for any other purpose.

## NEUROLOGIC AND/OR PSYCHIATRIC

**68680 ALCOHOL ABUSE**

**68681 SUBSTANCE ABUSE,** specify

**68686 MENTAL STATUS IMPAIRMENT**

**68687 MOOD DISORDERS**

**68515 PSYCHIATRIC DISEASE/DISORDER, OTHER,** specify diagnosis

## PULMONARY

**68690 ASTHMA**

**68691 PNEUMOTHORAX**

**68692 RESPIRATORY FAILURE**

**68507 RESPIRATORY DISEASE/DISORDER, OTHER,** specify diagnosis

## STD

**69611 CHANCROID**, specify site

**69605 CHLAMYDIA TRACHOMATIS**

**69604 NEISSERIA GONORRHEA**

**69610 SYPHILITIC ULCER**, specify site

**68700 SYPHILIS**

**69603 TRICHOMONAS VAGINALIS**

**69612 WARTS (EXCLUDING ORAL)**, specify site

**68522 VENEREAL DISEASE, OTHER,** specify diagnosis

FSTRF maintains this code set on behalf of several clinical trials networks for which it is the data center.
Permission must be obtained through user.support@fstrf.org to use or copy it for any other purpose.

## OTHER GENERAL DIAGNOSES CODES

**68501** **ALLERGY**, specify diagnosis

**68502** **EYE, EAR, NOSE DISEASE**, specify diagnosis

**68520** **HEMATOLOGIC DISEASE** (other than clotting disorder), specify diagnosis

**68523** **HEMOPHILIA**

**68503** **MOUTH OR THROAT DISEASE**, specify diagnosis

**68513** **REPRODUCTIVE SYSTEM DISEASE/DISORDER, MALE OR FEMALE**, specify diagnosis

**68599** **OTHER EVENT NOT LISTED IN APPENDIX 60**, non HIV-associated

FSTRF maintains this code set on behalf of several clinical trials networks for which it is the data center.
Permission must be obtained through user.support@fstrf.org to use or copy it for any other purpose.

# DIAGNOSES INDEX


Abdominal Wound Infection (Caesarean)
- Major ... 56
- Minor ... 56

Abortion
- Therapeutic (elective/induced) ... 49

Abortion/spontaneous (Miscarriage) ... 49
Addison's disease ... 65
Adenocarcinoma
- Endocervical ... 42
- Endometrial ... 42
- Extrauterine ... 42
- Other site ... 42

Adrenarche, Premature ... 66
Alcohol Abuse ... 95
Allergy ... 96
Alper's Disease ... 62
Amniotic Fluid Infection ... 49
Aneurysm, Aortic ... 76
Angina Pectoris ... 76
Anogenital Neoplasia, Preinvasive ... 47
Anomalies of the Ear, congenital ... 60
Anomalies of the Eye, congenital ... 60
Anomalies of the Nose, congenital ... 60
Anxiety ... 90
Aphthous Stomatitis, Recurrent ... 72
Arrhythmia, significant ... 76
Arthritis ... 94
Asthma ... 95
Atrophy
- Cervical ... 42
- Vaginal ... 42

Atypia
- Glandular cell (AGCUS), cervical ... 43
- Squamous cell (ASCUS), cervical ... 43

Autism ... 67
Avascular Necrosis ... 94
Bacteremia ... 23
- Catheter Related ... 23

Balance Disorder ... 69
Birth Defect, other ... 61
Blastomycosis
- Disseminated ... 8
- Pulmonary ... 13

Bleeding, vaginal ... 49
- <28 weeks ... 49
- >=28 weeks ... 49

Body Cavity Infection, Bacterial ... 16
Cancer, Squamous Cell, Invasive . see Squamous Cell Cancer,Iinvasive
Cancer/Dysplasia/Intraepithelial Neoplasia
- Vaginal ... 45
- Vulvar ... 45

Candidiasis
- Bronchi, Trachea or Lungs ... 8
- Esophageal ... 8
- Oral/Oropharyngeal ... 9
- Other Candidiasis ... 10
- Vulvovaginal ... 9

Carcinoma in situ ... 43
Carcinoma, Cervical, Invasive ... 43
Carcinoma, Squamous cell ... 48
Cardiomyopathy
- Drug-induced ... 81
- Etiology Unknown ... 81
- HIV-Related ... 81

Cardiomyopathy,
- HIV-Associated, Symptomatic ... 76

Cardiovascular System
- Disease/Disorder, other ... 81

Catheter Exit Site Infection, Bacterial ... 16
Cervical Carcinoma, Invasive ... 43
Cervical Inflammation ... 43
Chagas' Disease
- Central Nervous System Involvement ... 3
- Myocarditis ... 3

Chancroid ... 95
Cheilitis, Angular ... 72
Chickenpox ... *see* Varicella Zoster (VZV)
Childhood Disintegrative Disorder ... 67
Chlamydia trachomatis ... 95
Chorioamnionitis ... 49
Cleft Lip ... 60
Cleft Palate ... 60
Clinical Event diagnosis, other ... 75
CNS Anatomical Defect, other ... 60
CNS Disease/Disorder, other ... 67
CNS Lymphoma, Primary (PCL) ... 46
CNS Mass Lesion, Undetermined Etiology ... 67


FSTRF maintains this code set on behalf of several clinical trials networks for which it is the data center.
Permission must be obtained through user.support@fstrf.org to use or copy it for any other purpose.

Coccidioidal Meningitis....................................10

Coccidioidomycosis
  Disseminated....................................10
  Pulmonary....................................13
Communication Disorders ....................................67
Cord Prolapse....................................49
Coronary Artery Disease (CAD)....................................82
Coronary Heart Disease (CHD) ....................................82
Cryptococcal Meningitis....................................11
Cryptococcosis
  Disseminated....................................11
  Pulmonary....................................13
Cryptosporidiosis....................................4
Cushing's Syndrome ....................................65
Cutaneous Defects ....................................60
Cyclic Vomiting Syndrome ....................................62
Cyclospora Gastroenteritis....................................4
Cytomegalovirus (CMV
  Colitis, Adult ACTG Criteria....................................29
  Colitis, international....................................29
  Encephalitis....................................30
  Esophagitis....................................31
  Gastroenteritis....................................31
  Mucocutaneous ulcers....................................32
  Other CMV syndromes ....................................34
  Pneumonitis....................................32
  Proctitis ....................................33
  Retinitis....................................33
Deep Tissue Infection, Bacterial....................................16
Deep Vein Thrombosis ....................................77
Dementia, HIV-associated ....................................68
Depression....................................90
Dermatitis, Seborrheic ....................................72
Diabetes Mellitus ....................................82
Diabetes, Gestational
  Diet....................................50
  Medication dependent....................................50
Diabetic Ketoacidosis ....................................94
Diaphragmatic Hernia, congenital ....................................60
Diarrhea
  Chronic....................................17
  Persistent....................................17
Diarrheal Syndrome, acute....................................17
DNA Depletion, Mitochondria ....................................
  *see* Mitochondrial DNA Depletion
Down Syndrome ....................................60
Dream Abnormality ....................................91
Drowsiness....................................91
Dysentery, Acute ....................................18
Dysplasia, grade unknown....................................44
Dysplasia/Intraepithelial Neoplasia
  Low grade/grade 1 ....................................44
  Moderate, grade 2 ....................................44
  Severe, grade 3 ....................................44
Dysplasia/Intraepithelial Neoplasia/Cancer
  Vaginal....................................45
  Vulvar ....................................45
Ear Disease ....................................96
Ear, Anomalies of, congenital ....................................60
Eclampsia....................................50
Embolus, Pulmonary ....................................79
Encephalomyelopathy, Necrotizing,
  Subacute/Leigh Syndrome....................................63
Encephalopathy, other ....................................69
Endocarditis, Bacterial....................................19
Endocrine Birth Defect, other....................................60
Endometritis....................................50
Enterocolitis, Necrotizing, Neonatal....................................58
Epilepsy ....................................69
Episiotomy Infection
  Major ....................................51
  Minor ....................................51
Epstein Barr Vvirus (EBV)....................................34
Eye Disease....................................96
Eye, Anomalies of, congenital....................................60
Failure To Thrive....................................65
Fat Accumulation (Lipodystrophy) ....................................83
Fat Loss (Lipoatrophy) ....................................84
Febrile Morbidity
  Intrapartum ....................................51
  Postpartum....................................51
Fetal Alcohol  Syndrome....................................60
Fever, Persistent, Unexplained ....................................72
Focal Neurological Deficit ....................................70
Fracture....................................94
Fungi, other....................................15
Gait Disorder ....................................69
Gastrointestinal Syndrome, Acute ....................................17
Gastrointestinal System
  Disease/Disorder, other....................................94
Gastrointestinal, Anatomical Defect....................................60
Genitourinary, Anatomical Defect
  Female ....................................60
  Male....................................60
Genitourinary Defect, other....................................60
Gestational Diabetes
  Diet ....................................50

Medication dependent....50
Glucose
Impaired tolerance....82
Impaired fasting....82
Glycogen Storage Disease, congenital....60
Hallucinations....91
Hansen's Disease/Leprosy....20
Heart Defects (birth, anatomical)....60
HELLP Syndrome....51
Hematologic Disease, other than Clotting Disorder....96
Hematoma
Vaginal....52
Vulvar....52
Hemolytic Disease of the Newborn
ABO....57
Rh....57
Hemophilia....96
Hemorrhage, intrapartum
Requiring surgical procedure....52
Requiring transfusion....52
With hemodynamic instability....52
Hemorrhage, neonatal
Intraventricular, grade 4....57
Intrventricular, grade 3....57
Hemorrhage, postpartum
Requiring surgical procedure....52
Requiring transfusion....52
With hemodynamic instability....52
Hepatitis
Neonatal....57
Hepatitis B, chronic....35
Hepatitis C, chronic....35
Hepatitis, acute....34
Hernia, Diaphragmatic, congenital....60
Herpes Labialis....37
Herpes Simplex Virus (HSV)
Esophagitis....36
Mucocutaneous....36
Pneumonitis....37
Herpes Simplex, Intra-Oral....37
Herpes Zoster.... *see* Varicella Zoster (VZV)
HHV6, Roseola Infantum....35
Histoplasmosis
Disseminated....12
Pulmonary....13
HIV-Associated Dementia (Encephalopathy)..68
HIV-Associated Myopathy....69
HIV-Associated Nephropathy....73
Hypersensitivity Reaction
Other drug induced (i.e. Abacavir-like)....94
Study drug related....94
Hypertension....77
Chronic, in pregnancy....53
Pregnancy induced....53
Pulmonary....81
Hyperthyroidism....65
Hypogonadism....94
Hypomania....92
Hypotension....81
Hypothyroidism....65
Immune Reconstitution Inflammatory Syndrome (IRIS)....73
Impaired Fasting Glucose....82
Impaired Glucose Tolerance....82
Inborn Errors of Metabolism....60
Incompetent Cervix....53
Emergency Cerclage....53
Prophylactic Cerclage....53
Infant of diabetic mother....60
Insomnia....92
Intraepithelial Neoplasia/Dysplasia
Low grade, grade 1....44
Moderate, grade 2....44
Intraepithelial Neoplasia/Dysplasia
Severe, grade 3....44
Intraepithelial Neoplasia/Dysplasia/Cancer
Vaginal....45
Vulvar....45
Intrauterine Fetal Demise....53
Intrauterine Growth Restriction (IUGR)
Fetal....53
Severe....53
IRIS (Immune Reconstitution Inflammatory Syndrome)....73
Isosporiasis....4
IUD Reactive Changes....45
Kaposi sarcoma (KS)
Mucocutaneous....45
Visceral....45
Kearns-Sayre Syndrome (KSS)....62
Kernicterus....57
Lactic Acidemia....85
Lactic Acidosis....85
Leber Progressive Optic Neuropathy....63
Leigh Syndrome....63
Leishmaniasis....5
Lipid Abnormality....94

FSTRF maintains this code set on behalf of several clinical trials networks for which it is the data center.
Permission must be obtained through user.support@fstrf.org to use or copy it for any other purpose.

Liver Disease ........ 86
Lymphoma, Non-Hodgkin
  Immunoblastic ........ 46
  Large cell ........ 46
  Small non-cleaved
    (Burkitt or Burkitt's type) ........ 46
Lymphoma, Primary CNS (PCL) ........ 46
Malaria ........ 5
Malignancy, newly diagnosed, except Squamous
  Cell cancer of the skin ........ 47
Mania ........ 92
Mass lesion, CNS, undetermined etiology ........ 67
Mastitis ........ 54
Measles, Rubeola ........ 37
Meconium Aspiration Syndrome ........ 57
Mental Status Impairment ........ 95
Metabolic/Endocrine Disorder, other ........ 66
Metabolism, Inborn Errors of ........ 60
Microsporidiosis ........ 5
Miscarriage ........ *see* Abortion, spontaneous
Mitochondrial DNA Depletion
  Congenital Myopathy ........ 63
  Hepatopathy ........ 63
  Infantile Myopathy ........ 63
Mitochondrial Encephalopathy Lactic Acidosis
  and Stroke Like Episodes (MELAS) ........ 63
Mitochondrial Syndrome, not listed ........ 64
Mold Infections
  Aspergillus species ........ 14
  Mucormycosis ........ 14
  Other Mold Infections ........ 14
Mood Disorders ........ 95
Motor Developmental Delay ........ 69
Mouth Disease ........ 96
Mumps, Parotitis ........ 38
Musculoskeletal
  Abnormality, congenital ........ 60
  Absence of, congenital ........ 60
  Duplication ........ 61
Musculoskeletal System Disease/Disorder,
  Other ........ 94
Mycobacterial infection, non-Tuberculous,
  non-MAC
  M. Genovensii ........ 21
  M. Kansasii ........ 21
  Non-MAC, non-TB Mycobacteria, other ........ 21
Mycobacterium Avium Complex (MAC) ........ 20
Myocardial Infarction
  Acute ........ 78
  Silent ........ 79
Myoclonic Epilepsy and Ragged Red Fiber
  Myopathy (MERRF) ........ 64
Myopathy
  HIV-Associated ........ 69
Myositis ........ 94
Nail infections, fungal ........ 15
Necrotizing Encephalomyelopathy,
  Subacute/Leigh Syndrome ........ 63
Necrotizing Enterocolitis, Neonatal ........ 58
Necrotizing Ulcerative Gingivitis or
  Periodontitis ........ 73
Neisseria gonorrhea ........ 95
Neoplasia, Preinvasive Anogenital ........ 47
Neoplasm, gynecological ........ 47
Nephrolithiasis ........ 94
Nephropathy
  Drug induced ........ 87
Neural Tube Defect ........ 61
Neurogenic Progressive External
  Ophthalmoplegia ........ 64
Neurologic System Disease/Disorder, other ........ 70
Neurological Deficit, Focal ........ 70
Neuromuscular Weakness Syndrome,
  HIV-Associated ........ 87
Neuropathy
  Peripheral, Sensory ........ 70
  Sensory ........ 87
Neuropathy, Ataxia and Retinitis
  Pigmentosa (NARP) ........ 64
Non-Hodgkin Lymphoma ........
  *see* Lymphoma, Non-Hodgkin
Nose Disease ........ 96
Nose, Anomalies of, congenital ........ 60
Oligohydramnios ........ 54
Oral Hairy Leukoplakia ........ 38
Oral Warts ........ 38
Osteomyelitis ........ 21
Osteopenia ........ 88
Osteoporosis ........ 88
Other event not listed in appendix 60 ........ 96
Pancreatitis ........ 89
Paracoccidioidomycosis ........ 12
Parotitis, Mumps ........ 38
Parvovirus B19 ........ 38
Pearson's Syndrome (PS) ........ 64
Pelvic Inflammatory Disease (PID) ........ 22
Penicilliosis Marneffei, disseminated ........ 15
Peripheral Nerve Disease/Disorder, other ........ 71


FSTRF maintains this code set on behalf of several clinical trials networks for which it is the data center.
Permission must be obtained through user.support@fstrf.org to use or copy it for any other purpose.

## APPENDIX 60 - Diagnoses Appendix


Peripheral Neuropathy, Sensory ......................70
Peripheral Vascular Disease (PVD) .................79
Pigment Disorders, birth defect .......................61
Placenta
Accreta ........................................................54
Increta .........................................................54
Percreta .......................................................54
Previa ..........................................................55
Placenta, Abruptio...........................................54
Pneumocystis Carinii Pneumonia (PCP)...........6
Pneumocystosis, Extra-pulmonary....................6
Pneumonia, bacterial.......................................22
Pneumonia, Probable and/or Etiology
Unknown.....................................................74
Pneumothorax .................................................95
Polyhydramnios ..............................................55
Polyp, cervical ................................................47
Polyp, endometrial ..........................................47
Polyp, specify site ..........................................47
Postdates/Post-term Pregnancy .......................55
Precocious Puberty..........................................66
Pre-eclampsia ..................................................55
Pregnancy
Ectopic ........................................................55
Intrauterine..................................................55
Premature Adrenarche.....................................66
Premature Labor..............................................54
Premature Rupture of Membranes ..................56
Preterm .......................................................55
Premature Thelarche .......................................66
Preterm Delivery .............................................56
Progressive Multifocal Leukoencephalopathy
(PML).........................................................39
Proximal Renal Tube Dysfunction (PRTD).....94
Psychiatric Disease/Disorder, other ................95
Psychosis........................................................92
Pulmonary Embolus........................................79
Pyloric Stenosis, congenital ............................61
Radiation Cell Changes...................................44
Rash, Drug-Related.........................................89
Renal Fanconi Syndrome................................64
Renal Insufficiency
Acute ..........................................................89
Chronic.......................................................89
Renal System Disease/Disorder, other............94
Reproductive System Disease/Disorder
Female........................................................96
Male ...........................................................96
Respiratory Birth Defect, other.......................61
Respiratory Disease/Disorder, other................95
Respiratory Distress Syndrome, newborn .......58
Respiratory Failure .........................................95
Roseola Infantum, HHV6 ...............................35
Rubella...........................................................39
Rubeola, Measles............................................37
Salivary Gland Enlargement (parotid)............66
Salivary Hypofunction....................................66
Seizure disorder (not Epilepsy) ......................72
Sepsis
Bacterial.....................................................23
Catheter Related..........................................23
Neonatal......................................................58
Salmonella (non-Typhoid)..........................24
Shingles .................. *see* Varicella Zoster (VZV)
Shock .............................................................81
Sinusitis, bacterial...........................................25
Skin
Birth Defect, other ......................................61
Skin disease/disorder, other ............................94
Squamous Cell Cancer, Invasive
Anogenital ..................................................48
Cervical......................................................48
Squamous Cell Carcinoma, ............................48
Sterile Site Infection, Bacterial.......................16
Stevens-Johnson Syndrome ............................94
Stroke
Hemorrhagic ...............................................80
Ischemic Infarction .....................................80
Unknown type.............................................80
Substance Abuse.............................................95
Suicidal Ideation .............................................93
Syphilis ..........................................................95
Congenital, early.........................................58
Congenital, late, asymptomatic...................58
Congenital, late, symptomatic ....................58
Syphilitic Ulcer..............................................95
Tachypnea, Transient, Newborn.....................59
Thelarche, Premature ......................................66
Throat Disease ................................................96
Torch Syndrome .............................................59
Toxoplasmic Encephalitis.................................7
Toxoplasmosis
Congenital...................................................59
Non-CNS ......................................................7
Transient Ischemic Attack ..............................80
Trichomonas Vaginalis...................................95
Trisomies- Trisomy ........................................61
Trisomy 21.........................see Down Syndrome



FSTRF maintains this code set on behalf of several clinical trials networks for which it is the data center.
Permission must be obtained through user.support@fstrf.org to use or copy it for any other purpose.

## APPENDIX 60 - Diagnoses Appendix


Tuberculosis
- Extra Pulmonary (includes Miliary) ............28
- Latent ..........................................................26
- Pulmonary ....................................................27

Tunnel Infection, Bacterial ..............................16
Turner Syndrome .............................................61
Ulcerations NOS ..............................................75
Ulcerative Stomatitis, Necrotizing...................75
Upper Respiratory Tract Infections,
- Recurrent......................................................26

Uterine
- Atony ..........................................................56
- Inversion ......................................................56
- Rupture.........................................................56
- Scar Dehiscence ...........................................56

Varicella Zoster (VZV)
- Chickenpox .................................................39
- Disseminated Cutaneous Herpes Zoster, Shingles ..................................................40
- Localized Cutaneous Herpes Zoster, Shingles ..................................................40
- Visceral Dissemination Herpes Zoster, Shingles ..................................................41

Vascular Lesions, birth defect .........................61
Venereal Disease, other ..................................95
Ventricular Failure, left ..................................77
Ventricular Hypertrophy, left ..........................78
Warts...............................................................95
Wasting Syndrome ..........................................74
Weight Loss.....................................................94



FSTRF maintains this code set on behalf of several clinical trials networks for which it is the data center.
Permission must be obtained through user.support@fstrf.org to use or copy it for any other purpose.
